# Cardiac arrest: An interdisciplinary scoping review of the literature from 2019^☆^

**DOI:** 10.1016/j.resplu.2020.100037

**Published:** 2020-11-04

**Authors:** Travis W. Murphy, Scott A. Cohen, K. Leslie Avery, Meenakshi P. Balakrishnan, Ramani Balu, Muhammad Abdul Baker Chowdhury, David B. Crabb, Karl W. Huesgen, Charles W. Hwang, Carolina B. Maciel, Sarah S. Gul, Francis Han, Torben K. Becker, on behalf of the Interdisciplinary Cardiac Arrest Research Review (ICARE) group

**Affiliations:** a Division of Critical Care Medicine, Department of Emergency Medicine, University of Florida, Gainesville, FL, USA; b Department of Emergency Medicine, University of Florida, Gainesville, FL, USA; c Division of Pediatric Critical Care, Department of Pediatrics, University of Florida, Gainesville, FL, USA; d Division of Neurocritical Care, Department of Neurology, University of Pennsylvania, Philadelphia, PA, USA; e Department of Neurology, Yale University, New Haven, CT, USA; f Division of Neurocritical Care, Department of Neurology, University of Florida, Gainesville, FL, USA; g Department of Surgery, Yale University, New Haven, CT, USA

**Keywords:** Heart arrest, Out-of-hospital cardiac arrest, Cardiopulmonary resuscitation, Epidemiology, Emergency medical services, Sudden cardiac death

## Abstract

**Objectives:**

The Interdisciplinary Cardiac Arrest Research Review (ICARE) group was formed in 2018 to conduct a systematic annual search of peer-reviewed literature relevant to cardiac arrest. Now in its second year, the goals of the review are to illustrate best practices in research and help reduce compartmentalization of knowledge by disseminating clinically relevant advances in the field of cardiac arrest across disciplines.

**Methods:**

An electronic search of PubMed using keywords related to cardiac arrest was conducted. Title and abstracts retrieved by these searches were screened for relevance, classified by article type (original research or review), and sorted into 7 categories. Screened manuscripts underwent standardized scoring of overall methodological quality and impact on the categorized fields of study by reviewer teams lead by a subject-matter expert editor. Articles scoring higher than 99 percentiles by category-type were selected for full critique. Systematic differences between editors’ and reviewers’ scores were assessed using Wilcoxon signed-rank test.

**Results:**

A total of 3348 articles were identified on initial search; of these, 1364 were scored after screening for relevance and deduplication, and forty-five underwent full critique. Epidemiology & Public Health represented 24% of fully reviewed articles with Prehospital Resuscitation, Technology & Care, and In-Hospital Resuscitation & Post-Arrest Care Categories both representing 20% of fully reviewed articles. There were no significant differences between editor and reviewer scoring.

**Conclusions:**

The sheer number of articles screened is a testament to the need for an accessible source calling attention to high-quality and impactful research and serving as a high-yield reference for clinicians and scientists seeking to follow the ever-growing body of cardiac arrest-related literature. This will promote further development of the unique and interdisciplinary field of cardiac arrest medicine.

## Introduction

The most recent estimates of the global burden of out-of-hospital cardiac arrest report a range from 40 to 51 per 100,000-person years, or just over 350,000 per year in the United States.^1^, ^2^, ^3^ Despite advances in prevention and timely care for these patients, significant regional variability and slow improvement in overall outcomes persists both within and between countries.^2^ Continual improvements in coordinated data collection and novel approaches to care of these patients are significant advances in addressing cardiac arrest on social, clinical, and molecular levels. With such wide-ranging literature across several disciplines, a need for a centralized source for the highest quality cardiac arrest research was recognized. The Interdisciplinary Cardiac Arrest Research Review (ICARE) Group was created in 2018 with its first results published in 2019.^4^ This review, now in its second iteration, systematically gathers and summarizes articles from multiple sources and presents those chosen as having specific relevance or value to different categories of cardiac arrest research. Unlike a formal systematic review or meta-analysis that appraises the literature based on a specific research question, the scope of this review is to provide and summarize yearly updates on high-quality cardiac arrest research. The intent of the ICARE review is to serve as a starting resource both for practitioners caring for cardiac arrest patients as well as academic researchers referencing the most impactful developments over the previous year.

## Methods

The methods for the 2019 ICARE edition are consistent with the 2018 review published previously and adopted from the Global Emergency Medicine Literature Review group’s methodology that has been published annually for more than fifteen years.^4^^,^^5^ This year, the 2019 ICARE working group is comprised of 43 members, including 30 reviewers, 9 editors, and 4 editorial board members. The working group consists of physicians, scientists, medical students, and graduate students from multiple disciplines and educational backgrounds in the field of cardiac arrest. All members of the working group are unpaid and selected through an application process prior to literature search. The 2019 ICARE procedures manual is available as Supplement 1.

### Literature search

Publications pertaining to cardiac arrest were searched on PubMed in two phases: the first included publications between January and August 2019 that was conducted in October 2019, and the second phase between September and December 2019 that was conducted in January 2020. To filter by article publication dates, both literature searches were performed using the “[PDAT]” PubMed/MEDLINE field description tag. Therefore, articles with either an ‘Electronic Date of Publication’ and/or ‘Print Date of Publication’ in 2019 were included. Inclusion and exclusion criteria were consistent with 2018 ICARE review. Only articles that were available in English were included. Publications that were commentaries, editorials, case reports, study design protocols, data releases, and letters to the editor were excluded. The full PubMed/MEDLINE search query is presented in Supplement 2.

### Article screening

The titles and abstracts of articles identified were screened by the technical and section editors independently based on detailed inclusion and exclusion criteria (Supplement 1). The kappa statistic for agreement on article inclusion at this stage was calculated to determine consistency in the screening process. Full texts of selected articles were classified as either original research (OR) or review (RE) according to the study design. The articles were classified into seven categories: Epidemiology & Public Health (EPH), Prehospital Resuscitation, Technology & Care Processes (PRE), In-Hospital Resuscitation & Post-Arrest Care Processes (IN), Prognostication & Outcomes (PRO), Pediatrics (PED), Basic Science & Pharmacology (BSP), and Interdisciplinary Guidelines & Reviews (GL).

### Article scoring

Using a predefined scale according to the study type (original research or review), each article was scored by reviewers after article screening with scores verified by each section editor. Total scores ranged between 0–22. Scoring scales for OR and RE articles are presented in Table 1, Table 2. To ensure scoring reliability amongst the working group, approximately 200 random articles were selected and the reviewer and editor scores of each were compared. Systematic differences between editor and reviewer scores for each of these 200 articles were assessed using Wilcoxon signed-rank test.

**Table 1:** Scoring of original research (OR) articles.

Quality measure	Question		Points
**Design** A	*Select* *One*	Descriptive studies (including case studies and case series, natural observation studies and descriptive surveys)	1 *-or-*
		Correlation studies (case control studies, prospective observational studies, retrospective studies)	2 *-or-*
		Non-randomized or non-blinded experimental studies	3 *-or-*
		Randomized, blinded experimental studies	4
B	Study design is appropriate to answer the authors’ hypothesis.		1
C	Correct statistical tests are used to analyze the data.		1
D	Results are presented accurately and without bias.		1
E	Limitations are clearly described, and the conclusions are supported by data.		1

Design Total	/ Out of max score 8		

**Ethics** A	The study was approved by an institutional review board (IRB)/institutional animal use and care committee, ethics committee, community group, as required by local laws.		2
B	Informed consent was obtained, or consent was waived by the IRB (*give point if not applicable, e.g., animal study*).		1
C	The authors declare their conflicts of interest or declare that none exist.		1

Ethics Total	/ Out of a max of 4		

**Importance** A	The study results are not specific to one certain patient population but are broadly generalizable to a variety of settings.		2
B	The topic being studied is an important one, in that it advances the field of cardiac arrest research or care.		2
C	The study is clearly relevant to the realm of cardiac arrest research or care.		1

Importance Total	/ Out of a max of 5		

**Impact** A	The findings or recommendations of this study may be feasibly implemented by practitioners* of cardiac arrest care.		2
B	Practitioners* would likely change their practice if they were aware of this study.		2
C	The authors of this study raise interesting questions that may stimulate further research.		1

Impact Total	/ Out of a max of 5 *Practitioner: reader practicing in the category of the article (physician, epidemiologist, pharmacist etc.)

**Table 2:** Scoring of review (RE) articles.

Quality Measure	Question	Points
**Clarity** A	The review has a clearly stated hypothesis or purpose.	2
B	The authors provide sufficient background to put the results of the review into context.	1
C	The review can be understood by someone with general medical or public health training.	1
D	The authors use clear language and appropriate graphs, tables, and figures throughout the article.	1

Clarity Total	/ Out of max score 5	

**Design** A	This is a formal meta-analysis or a systematic review that only includes studies with a control group.	3
B	There is a clear, reproducible method for the selection of studies included in this review.	2
C	Articles for this review were selected by at least two authors blinded to each other’s selection.	1
D	The data was aggregated and/or analyzed appropriately.	1

Design Total	/ Out of max score 7	

**Importance** A	The review is not specific to one certain patient population but is broadly generalizable to a variety of settings.	2
B	The topic being reviewed is an important one, in that it advances the field of cardiac arrest research or care.	2
C	This is clearly relevant to the realm of cardiac arrest research or care.	1

Importance Total	/ Out of max score 5	

**Impact** A	The findings or recommendations of this review appear to have applicability towards improving cardiac arrest research or care.	2
B	Practitioners* would likely change their practice if they were aware of this review.	2
C	The authors of this review raise interesting questions that may stimulate further research.	1

Impact Total	**3** / Out of max score 5 *Practitioner: reader practicing in the category of the article (physician, epidemiologist, pharmacist etc.)	

### Full article review

Articles scoring higher than 99 percentiles by category and type were chosen for a full review. Reviewers then summarized selected articles encompassing the objective, key findings, as well as strengths and limitations of each study. Subsequently, editors reviewed summaries for content, accuracy, and style according to each category.

## Results

**Fig. 1:**
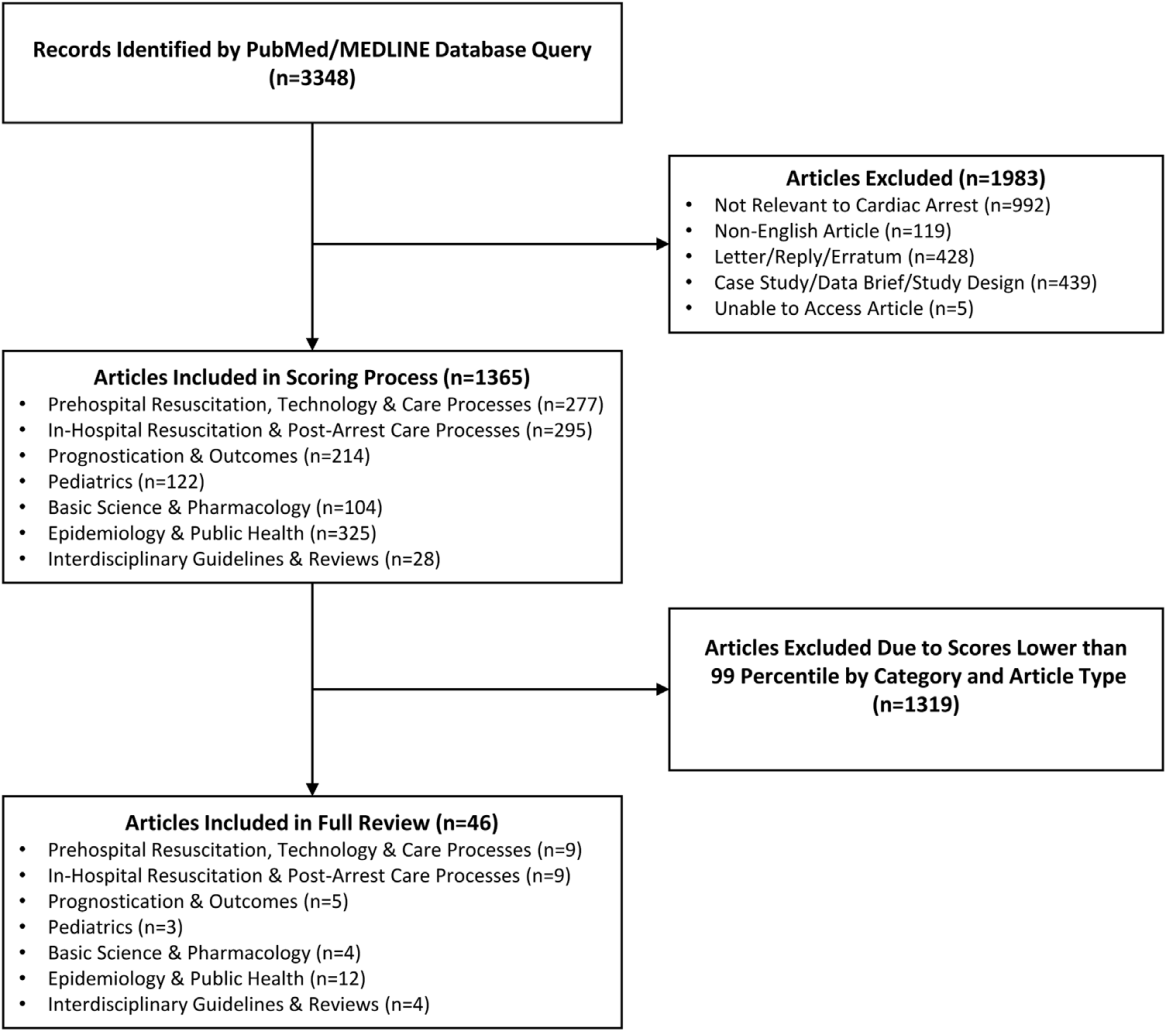
Flowchart of screening and scoring process.

The screening, scoring, and full article review process is presented in Fig. 1. A total of 3348 articles were identified in the initial search and after removal of duplicates and those not relevant to cardiac arrest; 1364 articles were categorized and underwent full review. Inter-rater reliability between the screening editors revealed a Cohen’s kappa score of 0.84 (95% CI: 0.77–0.92). Article scoring statistics for each article category are summarized in Table 3. Forty-five articles were selected for a full summary and critique; of these, 20 (44%) were OR articles. Twenty-four percent of summarized articles were in the EPH category and 20% each in PRE and IN. No significant differences between editor and reviewer scoring were found (p = 0.395).

**Table 3:** Summary statistics of reviewer scoring by category and article type.

Article category	Original research (OR)		Review (RE)	
	Count (%)	Median (IQR)	Count (%)	Median (IQR)
Basic Science & Pharmacology (BSP)	101 (8.7)	17 (15 – 18.5)	2 (1.0)	14.5 (14.3 – 14.8)
Epidemiology & Public Health (EPH)	262 (22.6)	15 (14 – 17)	62 (30.0)	14 (11 – 18)
Prehospital Resuscitation, Technology & Care Processes (PRE)	247 (21.3)	19 (16 – 20)	31 (15.0)	19 (17 – 21)
In-Hospital Resuscitation & Post-Arrest Care Processes (IN)	247 (21.3)	17 (15 – 18.5)	48 (23.2)	16.5 (13 – 20)
Prognostication & Outcomes (PRO)	199 (17.2)	14 (13 – 16)	15 (7.2)	12 (9.5 – 15)
Pediatrics (PED)	101 (8.7)	16 (14 – 18)	21 (10.1)	16 (11.5 – 18)
Interdisciplinary Guidelines & Reviews (GL)	---	--- ---	28 (13.5)	13 (11.4 – 18.6)

**Totals**	1157 (100.0)	16 (14 - 18)	207 (100.0)	16 (12 - 19)

## Discussion

Full summaries and critique of the top scoring cardiac arrest articles of 2019 identified by our review are available as Supplement 3. Below, we highlight the critical findings of our top-scoring articles (Table 4). This year, the search string was refined to be more specific and limit the initial number of articles and resulted in several thousand fewer articles extracted in the initial search but the total number of articles included for full review was not significantly different and in fact, a larger number of pertinent articles were able to be evaluated with this method (1364 from 2019 from 1214 in 2018).

**Table 4:** Interdisciplinary cardiac arrest research review 2019 articles.

Category	First Author	Title	Journal	Type	Summary
Basic Science & Pharmacology	Koziakova M	Noble gas neuroprotection: xenon and argon protect against hypoxic-ischaemic injury in rat hippocampus in vitro via distinct mechanisms.	Br J Anaesth	OR	Xenon and argon gas exhibit neuroprotective effect on hippocampal cells in vitro following oxygen-glucose deprivation model of cerebral ischemia. Helium, neon, and krypton are without neuroprotective effect.
	Chonde M	Intra-Arrest Administration of Cyclosporine and Methylprednisolone Does Not Reduce Postarrest Myocardial Dysfunction	Biomed Res Int	OR	Cyclosporin and methylprednisolone may have the potential to control the effects of post arrest myocardial dysfunction in patients who have been successfully resuscitated.
	Hoops HE	Selective aortic arch perfusion with fresh whole blood or HBOC-201 reverses hemorrhage-induced traumatic cardiac arrest in a lethal model of noncompressible torso hemorrhage.	J Trauma Acute Care Surg	OR	Selective aortic arch perfusion (Swith oxygenated fresh whole blood (FWB) has been previously shown to improve return of spontaneous circulation (ROSC). FWB is not always readily available in the pre-hospital setting, and it is not known if hemoglobin-based oxygen carriers (HBOC) are as efficacious. This study highlights how SAAP therapy with FWB or HBOC-201 in a swine model of HiTCA is (1) efficacious at achieving ROSC and (2) the possibility of converting to low-flow extracorporeal life support during management of HiTCA.
	Gazmuri RJ	Sodium-Hydrogen Exchanger Isoform-1 Inhibition: A Promising Pharmacological Intervention for Resuscitation from Cardiac Arrest.	Molecules	RE	The introduction of oxygenated blood to ischemic myocardial tissue results in further reperfusion injury. Cessation of coronary blood flow during CA leads to myocardial ischemia and accumulation of Ca2 + . Excess cytosolic and mitochondrial Ca2+ results in cell injury due to oxidative phosphorylation, release of pro-apoptotic factors, and increased mitochondrial inner membrane porosity. Attenuation of myocardial reperfusion injury can improve CA outcomes.
Epidemiology & Public Health	Czarnecki A	Association Between Hospital Teaching Status and Outcomes After Out-of-Hospital Cardiac Arrest.	Circ Cardiovasc Qual Outcomes	OR	Teaching hospital patients had more positive Out-of-hospital cardiac arrest (OHCA) outcomes then those treated at a non-teaching hospital.
	Nas J	Effect of Face-to-Face vs Virtual Reality Training on Cardiopulmonary Resuscitation Quality: A Randomized Clinical Trial	JAMA Cardiol	OR	This randomized noninferiority trial compared virtual reality (VR) cardiopulmonary resuscitation training to face-to-face training. VR training was shown to yield lower quality CPR than face-to-face training, with similar chest compression rate but inferior chest compression depth.
	Berger C	Combination of problem-based learning with high-fidelity simulation in CPR training improves short and long-term CPR skills: a randomised single blinded trial.	BMC Med Educ	OR	This prospective, randomized single-blind interventional study finds that a problem-based learning cardiopulmonary resuscitation (CPR) training leads to significantly improved CPR performance as compared with classical CPR training immediately following instruction. However, after six months the differences in performance are far less pronounced.
	Neves Briard J	Automated External Defibrillator Geolocalization with a Mobile Application, Verbal Assistance or No Assistance: A Pilot Randomized Simulation (AED G- MAP)	Prehosp Emerg Care	OR	Verbally providing the location of the nearest public automated external defibrillator to out of hospital cardiac arrest bystanders in a simulated environment seems effective in reducing the time to defibrillation compared to no assistance and to an automated external defibrillator geolocalizing mobile app.
	Bylow H	Self-learning training versus instructor-led training for basic life support: A cluster randomised trial.	Resuscitation	OR	There is no statistically significant difference in practical skills or willingness to act in a real-life OHCA situation when comparing self-learning training with instructor-led training, six months after training in BLS.
	Xu Y	An audio-visual review model enhanced one-year retention of cardiopulmonary resuscitation skills and knowledge: A randomized controlled trial	Int J Nurs Stud	OR	Audio-visual and audio-visual-practice review models revealed better 12-month retention on cardiopulmonary resuscitation skills and knowledge for family members of people at higher risk of out-of-hospital cardiac arrest.
	Haukilahti MAE	Sudden Cardiac Death in Women.	Circulation	OR	We demonstrated that women were more likely to experience nonischemic SCD than men, with PMF a remarkably more common cause of death among women than in men.
	Heard DG	Hands-Only Cardiopulmonary Resuscitation Education: A Comparison of On-Screen With Compression Feedback, Classroom, and Video Education.	Ann Emerg Med	OR	Hands-only CPR training using a kiosk results in skill acquisition and performance similar to that of traditional classroom training; both superior to video training. Hands-only CPR can be used for regular training and retraining of laypeople to increase bystander CPR.
	Hsieh YC	Hypoglycaemic episodes increase the risk of ventricular arrhythmia and sudden cardiac arrest in patients with type 2 diabetes-A nationwide cohort study.	Diabetes Metab Res Rev	OR	Hypoglycemic episodes (HEs) in patients with type 2 diabetes increase the risk of ventricular arrythmia and sudden cardiac arrest compared to those who do not experience HEs.
	Gonzalez-Salvado V	Training Adult Laypeople in Basic Life Support. A Systematic Review	Rev Esp Cardiol (Engl Ed)	RE	There is no single a gold standard for BLS, findings from a systematic review article.
	Chen KY	Interventions to improve the quality of bystander cardiopulmonary resuscitation: A systematic review	PLoS One	RE	This systematic review finds bystander cardiopulmonary resuscitation quality is improved with assistance by telephone dispatchers, compression only instructions, mobile apps, and real-time feedback devices. Mobile device and video use may delay initiation of chest compressions, however.
Prehospital Resuscitation, Technology & Care Processes	Harari Y	Paramedic equipment bags: How their position during out-of-hospital cardiopulmonary resuscitation (CPR) affect paramedic ergonomics and performance.	Appl Ergon	OR	Effective equipment handling and positioning have the potential for both reducing work-related injuries and improving performance efficiency.
	Teo MHN	The use of dispatcher assistance in improving the quality of cardiopulmonary resuscitation: A randomised controlled trial.	Resuscitation	OR	Dispatcher assisted CPR has potential to improve CPR quality in medical professionals and lay persons.
	Grunau B	North American validation of the Bokutoh criteria for withholding professional resuscitation in non-traumatic out-of-hospital cardiac arrest	Resuscitation	OR	The Bokutoh criteria allow reliable identification of the approximately 1/5 of patients in out-of-hospital cardiac arrest unlikely to benefit from EMS initiated resuscitation.
	Nas J	Diagnostic performance of the basic and advanced life support termination of resuscitation rules: A systematic review and diagnostic meta-analysis.	Resuscitation	RE	A meta-analysis that looks at different termination of resuscitation (TOR-rules) for BLS and ALS in different regions with focus on key arrest characteristics for the particular TOR-rules.
	Lalande E	Is point of care ultrasound a reliable predictor of outcome during atraumatic, non-shockable cardiac arrest? A systematic review and meta-analysis from the SHoC investigators	Resuscitation	RE	PoCUS has become a useful tool in predicting ROSC, survival to admission, and survival to discharge from the hospital in adult patients who are suffering from out of hospital non-traumatic cardiac arrest with arrhythmias consistent with pulseless PEA and asystole.
	Miraglia D	The evolving role of novel treatment techniques in the management of patients with refractory VF/pVT out-of-hospital cardiac arrest.	Am J Emerg Med	RE	Although the early results are promising, the current evidence is not strong for large-scale adoption of ECMO, esmolol, double sequential defibrillation, and stellate ganglion block as treatments for refractory ventricular fibrillation/pulseless ventricular tachycardia.
	Liu M	Mechanical chest compression with LUCAS device does not improve clinical outcome in out of hospital cardiac arrest patients	Medicine (Baltimore)	RE	Mechanical compression devices such as the LUCAS may provide consistent chest compressions, however, controversy exists over the quality of compressions automatic mechanical devices can deliver.
	Perkins GD	The effects of adrenaline in out of hospital cardiac arrest with shockable and non-shockable rhythms: Findings from the PACA and PARAMEDIC-2 randomised controlled trials.	Resuscitation	RE	Administration of adrenaline during OHCA significantly increases ROSC in non-shockable rhythms.
	Ng KT	The effect of prehospital epinephrine in out of hospital cardiac arrest: a systematic review and meta-analysis	Prehosp Disaster Med	RE	The use of epinephrine by prehospital providers increases return of spontaneous circulation and survival to discharge from the hospital, but at time of discharge no significant neurologic improvement is seen.
In-Hospital Resuscitation & Post-Arrest Care Processes	Beck S	Basic life support training using shared mental models improves team performance of first responders on normal wards: a randomised controlled simulation trial	Resuscitation	OR	A randomized controlled simulation trial demonstrated that Basic Life Support training focusing on team-based learning and group interaction reduces hands-off time.
	Goharani R	Real-time compression feedback for patients with in-hospital cardiac arrest: a multi-center randomized controlled clinical trial	J Intensive Care	OR	Use of the Cardio First Angel™, a hand-held chest compression feedback device, during in-hospital cardiac arrest improves patient survival and hospital discharge.
	Bartlett ES	Systematic review and meta-analysis of intravascular temperature management vs. surface cooling in comatose patients resuscitated from cardiac arrest.	Resuscitation	RE	Favorable neurologic outcome was significantly greater in post-cardiac arrest patients with the use of intravascular cooling methods, as compared to surface cooling methods, for induced hypothermia.
	Gottlieb M	Beta-blockade for the treatment of cardiac arrest due to ventricular fibrillation or pulseless ventricular tachycardia: A systematic review and meta-analysis.	Resuscitation	RE	Three studies demonstrated that when initial advanced cardiac life support measures fail in cardiac arrest patients with ventricular fibrillation or pulseless ventricular tachycardia, the use of beta-blockers may increase the likelihood for temporary or sustained return of spontaneous circulation, survival-to-discharge, survival-to-admission, and favorable neurologic outcome. Further randomized controlled trials are needed to evaluate these findings.
	Barbarawi M	Optimal timing of coronary intervention in patients resuscitated from cardiac arrest without ST-segment elevation myocardial infarction (NSTEMI): A systematic review and meta-analysis.	Resuscitation	RE	Coronary angiography, whether immediate or delayed, was found to increase both short- and long-term survival, as well as provide increased favorable neurological outcomes, among non-ST-elevation myocardial infarction cardiac arrest patients.
	Kyriazopoulou E	Sinus Bradycardia During Targeted Temperature Management: A Systematic Review and Meta-Analysis.	Ther Hypothermia Temp Manag	RE	Sinus bradycardia was associated with significant decreases in patient mortality and favorably affects neurological function in post-cardiac arrest patients.
	Calabro L	Effect of different methods of cooling for targeted temperature management on outcome after cardiac arrest: a systematic review and meta-analysis.	Crit Care	RE	This meta-analysis of targeted temperature management techniques demonstrated lower probability of unfavorable neurological outcome for core, invasive, and temperature feedback devices, when compared to their respective counterparts.
	Chen Z	Clinical Efficacy of Extracorporeal Cardiopulmonary Resuscitation for Adults with Cardiac Arrest: Meta-Analysis with Trial Sequential Analysis.	Biomed Res Int	RE	A meta-analysis comparing efficacy of extracorporeal cardiopulmonary resuscitation (ECPR) with traditional CPR found that cardiac arrest patients who received ECPR had improved survival and neurological outcome.
	Couper K	Prophylactic antibiotic use following cardiac arrest: A systematic review and meta-analysis.	Resuscitation	RE	Many patients who are successfully resuscitated following cardiac arrest often suffer from infective complications in the intensive care unit. The use of prophylactic or early antibiotics has therapeutic potential for reducing infective complications in post cardiac arrest patients.
Prognostication & Outcomes	Jentzer JC	Cardiogenic Shock Classification to Predict Mortality in the Cardiac Intensive Care Unit.	J Am Coll Cardiol	OR	The Society for Cardiovascular Angiography and Intervention (SCAI) classification scheme may be useful in the risk stratification of patients with cardiogenic shock, as SCAI shock stage is independently associated with all-cause hospital mortality.
	Petek BJ	Reexamination of the UN10 Rule to Discontinue Resuscitation During In-Hospital Cardiac Arrest	JAMA Netw Open	OR	The UN10 rule, a clinical decision rule for termination of resuscitation following in-hospital cardiac arrest, may have less predictive utility than suggested in previous studies.
	Fernando SM	Pre-arrest and intra-arrest prognostic factors associated with survival after in-hospital cardiac arrest: systematic review and meta-analysis.	BMJ	RE	In patients with in-hospital cardiac arrest, male sex, age greater than 60, active malignancy, and chronic kidney disease are highly predictive of early mortality. Cardiac arrests that are witnessed, in monitored patients, occurred during daytime hours, with a shockable rhythm and those that do not require intubation during arrest are associated with higher early survival.
	Shin H	Procalcitonin as a prognostic marker for outcomes in post cardiac arrest patients: a systematic review and meta-analysis	Resuscitation	RE	This systematic review and meta-analysis compared 10 studies with a total of 1065 patients and found that there was a positive correlation with procalcitonin levels drawn within 0-48 hours of hospital admission and in-hospital mortality as well as poor neurologic outcome.
	Lopez Soto C	Imaging for Neuroprognostication After Cardiac Arrest: Systematic Review and Meta-analysis	Neurocrit Care	RE	Lower gray-white ratio on noncontrasted head CT is highly specific (though carries a suboptimal sensitivity) for predicting poor neurologic outcomes post-cardiac arrest, whereas Diffusion Weighted Imaging on brain MRI has a higher sensitivity (at an expense of higher false positive rates or inaccurate predictions of outcome) for severe hypoxic-ischemic brain injury.
Pediatrics	Kim CW	Effect of metronome guidance on infant cardiopulmonary resuscitation	Eur J Pediatr	OR	In an infant CPR model, use of a metronome significantly increased the percentage of adequate chest compression rates, in both two-finger and two-thumb techniques, while maintaining adequate chest compression depth.
	Duff JP	2019 American Heart Association Focused Update on Pediatric Advanced Life Support: An Update to the American Heart Association Guidelines for Cardiopulmonary Resuscitation and Emergency Cardiovascular Care	Pediatrics	RE	This review summarizes findings and publishes updated recommendations based on systemic reviews of 1) advanced airway management in pediatric out of hospital cardiac arrest, 2) extracorporeal cardiopulmonary resuscitation following pediatric cardiac arrest and 3) targeted temperature management during post-cardiac arrest care.
	Soar J	2019 International Consensus on Cardiopulmonary Resuscitation and Emergency Cardiovascular Care Science With Treatment Recommendations: Summary From the Basic Life Support; Advanced Life Support; Pediatric Life Support; Neonatal Life Support; Education, Implementation, and Teams; and First Aid Task …	Circulation	RE	The Pediatric Task Force of the ILCOR systemically reviews and grades recent, peer-reviewed, published cardiopulmonary resuscitation science. This 3rd focused update summarizes findings supporting dispatcher-assisted CPR guidance to improve survival for pediatric patients suffering OHCA.
Interdisciplinary Guidelines & Reviews	Nas J	Meta-Analysis Comparing Cardiac Arrest Outcomes Before and After Resuscitation Guideline Updates	Am J Cardiol	RE	Updates to cardiopulmonary resuscitation guidelines in 2005 and 2010 are associated with improved rates of return of spontaneous circulation, survival to admission, survival to discharge, and favorable neurologic outcome.
	Granfeldt A	Advanced Airway Management during Adult Cardiac Arrest: A Systematic Review	Resuscitation	RE	Despite a need for clarity on the most effective airway management strategy, current studies have high risk of bias and significant heterogeneity which precludes a systematic examination and conclusion on best practice.
	Panchal AR	2019 American Heart Association Focused Update on Systems of Care: Dispatcher-Assisted Cardiopulmonary Resuscitation and Cardiac Arrest Centers	Circulation	RE	Dispatcher-assisted CPR is associated with improved survival and neurologic outcomes after out-of-hospital cardiac arrest. Post-arrest care in specialized cardiac arrest centers may also improve survival and outcomes.
	Holmberg MJ	Vasopressors during Adult Cardiac Arrest: A Systematic Review and Meta-Analysis	Resuscitation	RE	Standard dose epinephrine boluses improve survival after sudden cardiac arrest, especially in patients with non-shockable initial rhythms. Vasopressin and vasopressin plus epinephrine do not appear to improve survival compared to epinephrine only.

### Epidemiology & Public Health initiatives (EPH)

The importance of timely and effective bystander cardiopulmonary resuscitation (CPR) has long been recognized as a key link in the chain of survival from cardiac arrest and efforts to affect change have been the topic of much study. As evidence of this, seven of the twelve highest quality articles in the EPH section pertain specifically to training initiatives to increase rates and quality of bystander CPR among various learner groups. Five were randomized trials of teaching interventions and two were systematic reviews of interventions to improve the quality of bystander CPR.^6^, ^7^, ^8^, ^9^, ^10^, ^11^, ^12^ The other studies in this section addressed specific populations and the unique risk factors including one each dedicated to women, patients with type 2 diabetes and another comparing cardiac arrest outcomes between community and academic hospitals.^13^, ^14^, ^15^

Systematic reviews of interventions to improve the quality and rate of bystander CPR by Gonzalez-Salvado and Chen found that the methods for teaching, including the use of dispatcher assistance, mobile phone apps, compression-only instructions and real-time feedback devices, affect skills retention and brought attention to the lack of a current ‘gold-standard’ in teaching basic life support skills to adult laypeople.^11^^,^^12^ Heard et al. compared three different methods for teaching hands-only CPR to adults in the Denver, Colorado area and found that a 4-min kiosk with manikin and 30-min traditional classroom both had better rates of effective CPR than a 1-min video during a 30-s compressions test.^7^ Skill retention at three months was similar among all groups. This is in keeping with the findings of Nas et al. who also found that virtual reality methods were not superior to in-person training.^6^ Berger et al. found that a problem-based-learning approach to in-person trainings improved short term performance but the underlying need for retraining was also identified in all of these training studies with skill deterioration noted in all follow-up periods after initial training.^8^ Bylow et al. specifically assessed six-month willingness and skill retention and found no significant difference between learners randomized to self-learning versus instructor-led models.^9^ Xu et al. went further to assess skill retention at 12 months after randomization to either receipt of regular reminders via phone, video and skills sessions, as compared to receipt of an instruction booklet and placebo DVD without reminders as a control.^10^ Skill retention at 12 months was higher in both the audio-visual and audio-visual-practical intervention groups. In addition to promoting high quality chest compressions, a Canadian study by Briad et al. evaluated the impact of dispatcher-assisted AED localization in a randomized simulation model and found that time to first defibrillation was significantly shorter in groups that received assistance from the dispatcher compared to those that did not.^16^

Several EPH studies reported on specific at-risk patient populations. Hsieh et al. found in their cohort study utilizing the Taiwanese National Health Insurance Research Database that diabetic patients with episodes of hypoglycemia were at an increased the risk of ventricular arrythmias and sudden cardiac death in diabetic patients and those with no hypoglycemic episodes.^13^ Haukilathi et al. found in their cohort of residents of northern Finland that the rate of nonischemic heart disease and primary myocardial fibrosis was found to be higher in women though men were more likely than women to die of sudden cardiac death at younger ages in keeping with prior studies on the subject.^14^

In comparing the rates of survival and utilization of best practices in cardiac arrest including targeted temperature management (TTM) and cardiac catheterization, Czarnecki et al. found that patients with out-of-hospital cardiac arrest taken to teaching facilities in Ontario were more likely to receive these essential elements of care, correlating with a higher rate of survival of the event and ICU discharge.^15^

### Prehospital Resuscitation, Technology & Care Processes (PRE)

In treating out-of-hospital cardiac arrest, early recognition and contact with emergency medical services is essential. Nine of the top-scoring articles in this year’s review fell into the PRE category. Two studies examined the effect of pre-hospital medications and interventions, namely adrenaline (epinephrine) in the prehospital setting, and one reviewed the use of esmolol, extracorporeal support, double-sequential defibrillation, and stellate ganglion block in the management of ventricular fibrillation and pulseless ventricular tachycardia. The others focused on improved processes by which prehospital providers can help ensure high quality chest compressions and best practices in resuscitation.

A randomized controlled trial of simulated dispatcher assisted CPR in Singapore found that compression rate improved with dispatcher assistance, most markedly in persons without a medical background or current CPR training. This finding is in keeping with other studies of dispatcher assistance and supportive of current guidelines to provide dispatcher assistance when possible.^11^^,^^17^

The PARAMEDIC-2 and PACS randomized controlled trials were the subject of two different meta-analyses which both found that the positive impact of epinephrine was most pronounced in patients with non-shockable rhythms, in keeping with current guidelines, but that no improvement in neurologic outcome can be shown.^18^, ^19^, ^20^

Refractory ventricular fibrillation and pulseless ventricular tachycardia can be especially difficult to treat. Miraglia et al. reviewed the evidence for proposed therapies for this condition including esmolol, double sequential defibrillation, extracorporeal membrane oxygenation and stellate ganglion block.^21^ No articles supporting the use of stellate ganglion block were found. Promising findings with improved neurologic outcomes with the use of extracorporeal support were noted though the authors stop short of recommending the widespread adoption of this technique, in keeping with current guidelines to consider this therapy in centers well equipped and experienced in performing the procedure.^18^^,^^21^ No consistently convincing evidence to support the routine use of double sequential defibrillation or esmolol in refractory ventricular fibrillation or pulseless ventricular tachycardia was found.

A common technologic component of prehospital response to cardiac arrest is the use of mechanical CPR devices. A systematic review and meta-analysis by Mao et al. evaluated the impact of the LUCAS device on clinical outcomes.^22^ The authors did not find evidence that mechanical CPR devices performed higher quality chest compressions or had higher rates of return of spontaneous circulation (ROSC) or survival, but did advocate for use of these devices to prevent provider fatigue and reduce interruptions in chest compressions. Regarding the use of new technologies during cardiac arrest, the results of the PRINCESS trial of pre-hospital, trans-nasal cooling were also released in 2019 though no significant improvement in 90-day survival or neurologic outcome was noted and the interventions of this trial were not found to be readily generalizable.^23^

Another prehospital technology article was a systematic review and meta-analysis of point-of-care ultrasound as a predictor of outcomes in out-of-hospital arrest by Lalande et al. and one of the first to specifically address this question.^24^ This group found that patients with cardiac activity on ultrasound had higher rates of ROSC, survival to admission, and ultimately discharge, although there was significant heterogeneity in the studies included and a lack of uniform definitions, evaluation parameters, and time intervals.

Regarding paramedics and the physical loads they bear while attending to patients in cardiac arrest, an investigation into the ergonomics of equipment positioning and work efficiencies by Harari et al. found that adjustments to the positioning of equipment around the patients led to improved CPR quality and ergonomics which could mean fewer work-related injuries.^25^

Two articles in this category addressed when termination of resuscitation should be considered. A systematic review and meta-analysis of different termination of resuscitation rules from across the world by Nas et al. found key differences in basic and advanced life support termination rules between Western and non-Western societies that reinforced the need for validation of rules against local practices to make them more applicable to practitioners. A secondary analysis of the data collected for the Trial of Continuous or Interrupted Chest Compressions during CPR which had data from over 25,000 patients sought to validate the Bokutoh criteria for termination of resuscitation in the North American context.^26^ The authors found the Bokutoh criteria to be 98% specific in their retrospective analysis though a number of confounders and limitations were noted.^26^

### In-Hospital Resuscitation & Post-Arrest Care Processes (IN)

Studies in cardiac arrest often focus on out-of-hospital arrests, but high-quality evidence to support in-hospital arrest management and post-arrest care is an integral part in the continuum of care for these patients. Nine of the top-scoring articles this year fell into this category with five post-arrest management studies making up the majority. Three of these five focused on targeted temperature management considerations. The remaining articles focused on clinician training in preparation for cardiac arrest events and intra-arrest therapy and performance metrics.

A randomized training simulation model for in-hospital providers aimed at improving team dynamics was the focus of one study by Beck et al. and found that developing shared mental models through specific training during their instruction resulted in minimized time without compressions.^27^ In a prospective, randomized study evaluating the effect of a real-time compression feedback device, Gohrani et al. found that use of the device studied was associated with improved rates of ROSC and survival to ICU and hospital discharge although neurologic or functional outcomes were not reported.^28^

When standard measures fail during cardiac arrest, other medications not explicitly recommended in the standard algorithms may be necessary. Beta-blockade for ventricular fibrillation or pulseless ventricular tachycardia has been evaluated through prior systematic reviews, but a meta-analysis of the evidence to support the practice had been lacking. Gottlieb et al., specifically reviewed human studies addressing this question and found that rates of survival and neurologic outcome were higher in the beta-blocker groups. This stands in contrast to the conclusion reported by Miraglia et al. as noted in the previous section, though there was significant heterogeneity in the inclusion criteria and post-arrest care among the studies included.^21^

A meta-analysis and trial sequential analysis by Chen et al. sought to address clinical efficacy of extracorporeal support for adults in cardiac arrest.^29^ This project specifically sought to determine the strength of existing evidence and confirmed that patients with cardiac arrest in the hospital who underwent extracorporeal resuscitation had better neurologic outcomes at 30 days and up to one year. This study did not find the same level of support among the existing evidence for patients with out-of-hospital arrest and further research in this realm was advocated, in keeping with previous studies of this subject.^21^

Following ROSC, providers make several critical decisions to prepare patients for the best possible outcomes. Barbarawi et al. conducted a systematic review and meta-analysis to examine the optimal timing of coronary intervention following cardiac arrest in patients without ST-segment elevation.^30^ They found that although the existing evidence- including a study by Lemkes et al. released just months prior to this review- had variability in what constituted ‘immediate’ versus ‘delayed’ catheterization, both of these groups had improved mortality and neurologic outcomes compared to those who did not undergo angiography.^30^^,^^31^ Couper et al. performed a systematic review and meta-analysis of prophylactic antibiotic use following cardiac arrest and while the authors found that prophylactic or early antibiotics did not improve outcome, the evidence quality among all studies included was low, making the case for further study of this important question.^32^ The three studies relating to targeted temperature management after a cardiac arrest addressed the significance of sinus bradycardia, as well as methods of temperature induction and systematic review with meta-analysis of the effects on outcomes of different methods of targeted temperature management. Kyriazopoulou et al. sought to determine the association between patient heart rate while undergoing temperature management and neurologic outcome through a systematic review.^33^ In this group’s meta-analysis of four retrospective studies, heart rates under 50 beats per minute were associated with improved mortality and neurologic outcome via possible cardioprotective effects. The two remaining articles addressing targeted temperature management were both systematic reviews and meta-analyses looking to compare neurologic outcomes and survival between different cooling methods. Both studies concluded that induction to goal temperature by core cooling or invasive cooling methods, such as intravascular approaches, and devices with temperature feedback provided better neurologic outcomes than surface cooling alone.^34^^,^^35^ Though not formally included with a full summary due to scoring just below the cut-off for inclusion for lack of blinding, the findings of the HYPERION study were also released in 2019 and provided convincing evidence for the use of targeted temperature management in patients with non-shockable cardiac arrest, marking a significant advance in the field.^36^

### Basic Science & Pharmacology (BSP)

Four top-scoring studies were selected in the BSP category. Apart from targeted temperature management, there are few therapeutic options to limit the extent of ischemia-reperfusion injury following a cardiac arrest. Three OR articles studied pharmacologic interventions that could improve outcomes post-arrest in both a medical and traumatic model. The fourth, a RE article discussed the roles of a molecular ion exchanger in models of altered cardiac perfusion.

The first, by Koziakova et al. conducted an in vitro model of hypoxic-ischemic injury of rat hippocampus to assess the neuroprotective effects of the noble gasses.^37^ The noble gases helium, neon, argon, xenon, and krypton have been topics of interest in recent years as neuroprotective agents following a neurologic insult – cardiac arrest included. This study exposed rat hippocampal cell cultures to an oxygen-glucose deprivation environment and found that both xenon and argon appear to have neuroprotective effects but via different mechanisms. While this study is in keeping with past findings regarding the neuroprotective effects of these noble gases and the model included clinically relevant injury and temperature control it remains difficult to assess the clinical implications by virtue of its in vitro design.

The second study by Manning et al. focused on a traumatic arrest model in swine with non-compressible thoracic hemorrhage using selective aortic arch perfusion.^38^ While HBOC-201 has been shown previously to improve rates of ROSC in previous traumatic arrest studies, the potential efficacy of selective aortic arch perfusion with HBOC-201 in direct comparison to perfusion with fresh whole blood was the motivation for this study.^38^^,^^39^ The three main objectives were comparing HBOC-201 to fresh whole blood in efficacy, feasibility in ECLS conversion, and reversal of systemic derangements using selective aortic-arch perfusion. ROSC was able to be obtained at a similar rate with both blood and HBOC-201, even in asystolic animals which is encouraging as HBOC-201 is more easily stored and transported than blood. Transitioning to extracorporeal support was deemed equally feasible. However, despite the rates of recovery, similar physiologic derangements in both groups from the ischemia-reperfusion injury were noted, with pulmonary hypertension particularly common in the HBOC-201 group. This paper showed an interesting and effective method for obtaining ROSC, though the known complication of pulmonary hypertension with HBOC-201 and complexity of administering this therapy may limit is broader adoption until more studies can be completed and reperfusion injury more reliably prevented.

The third basic science study to evaluate cyclosporin and methylprednisolone as possible intra-arrest therapies to limit the extent of post-arrest myocardial dysfunction pot-ROSC and found that swine in the intervention arm had higher mean arterial pressure and subsequent lower cardiac output but no improvement in post-arrest myocardial dysfunction.^40^ This is in keeping with previous Phase III trials of cyclosporin alone.^41^

A RE article authored by Gazmuri et al. regarding Sodium-Hydrogen Exchanger Isoform-1 (NHE-1) inhibition described a proposed pathophysiology of myocardial cell death and this ion exchanger’s potential as a target to limit progression of this process.^42^ While this specific target has only been studies in myocardial infarction and coronary bypass grafting in studies sponsored by pharmaceutical companies, its potential cardioprotective effects via interruption of the accumulation of cytosolic and mitochondrial calcium certainly warrants further study.

### Prognostication & Outcomes (PRO)

Five of the top-scoring articles fell into the Prognostication & Outcomes category. In the uncertain and critical context of treating such a resource intensive condition as cardiac arrest, methods for predicting neurologic outcomes remain a topic of significant research interest.

A predictor of cardiac arrest among patients with underlying cardiac disease conducted by Jentzer et al. found an increasing rate of in-hospital cardiac arrest among patients admitted to the cardiac intensive care unit with increasing classification level using the Society for Cardiovascular Angiography and Intervention criteria.^43^ For evaluating a broader population of in-hospital arrest patients, Fernando et al. conducted a systematic review and meta-analysis of 23 studies evaluating pre- and intra-arrest factors and found that age >60, male sex, active malignancy, and chronic kidney disease were highly predictive of mortality.^44^ Notably, patients with endotracheal intubation were also found to have lower chances of survival though this relationship bears further study given heterogeneity of the studies included, the risk of confounding factors and the retrospective nature of this investigation. Another metric to predict mortality of an in-hospital arrest is the UN10 Rule, originally proposed in 1999.^45^ Petek et al. sought to reexamine the utility of this rule in determination of mortality by retrospectively applying it to a cohort of over 95,000 patients using the AHA Get With the Guidelines Registry.^46^ This group found that the three-component rule was simple and easily implemented, but ultimately did not have the predictive power to justify its use given it’s nearly six percent false-positive rate, with many of those patients surviving with good neurologic outcomes.^46^

Several biomarkers for evaluating the likelihood of favorable neurologic recovery after ROSC have been proposed. A systematic review and meta-analysis noted this year sought to determine the prognostic utility of procalcitonin given its association with severity of systemic inflammation in sepsis. In pooling ten studies, Shin et al. found a positive correlation with serum procalcitonin levels drawn within 0–48 h after admission and in-hospital mortality as well as poor neurologic outcome.^47^

Recent studies of post-arrest imaging as a contributor to the multi-modal prediction of outcome after arrest was the topic of a systematic review and meta-analysis of 44 studies by Lopez Soto et al. Evidence of anoxic injury on MRI and CT grey-white ratio were noted to have consistent positive predictive value of poor neurologic outcomes.^48^ In the context of the other research on this topic and most recent guidelines, these results indicated that neuroimaging with CT or MRI after cardiac arrest can be another valuable prognostic tool for neurologic outcome of patients experiencing cardiac arrest; however, both standard times for imaging acquisition and methodology for hypoxic-brain injury burden quantification remain knowledge gaps.^49^

### Pediatrics (PED)

Three high-scoring articles were chosen from the pediatrics category, with two of the highest quality articles being Updates from the ILCOR Pediatric Task Force covering dispatcher assisted CPR, extracorporeal support, and targeted temperature management.^50^^,^^51^

Early action in cases of pediatric out-of-hospital cardiac arrest has been associated with improved rates of ROSC and positive neurologic outcomes.^52^ In keeping with adult guidelines, the ILCOR recommended that emergency medical dispatch centers provide dispatcher assistance for presumed pediatric cardiac arrests including instructions to start CPR when no bystander CPR is in progress based on the results of registry studies involving over 11,000 patients. The precise content, age-based recommendations and accommodations for ongoing bystander CPR or skill level and local EMS standards remain areas in need of further research. Proposed ventilation rates were the topic of another study by the Collaborative Pediatric Critical Care Research Network, but this was limited to in-hospital cardiac arrest.^53^ Committee guidelines on placement of advanced airways during cardiac arrest, extracorporeal support, and targeted temperature management were the subjects of a separate update. The 2019 Update reaffirmed 2010 guidance regarding the use of effective bag-mask ventilation over advanced airway placement in pediatric out-of-hospital arrests based on a lack of supporting evidence for improved outcome with endotracheal or supraglottic devices.^50^ No advanced airway recommendations were made for in-hospital arrests. In experienced ECMO centers, extracorporeal support for in-hospital arrests for pediatric patients with a cardiac diagnosis garnered additional support based on three new studies.^51^ Finally, the 2019 Update expanded on 2015 Pediatric Advanced Life Support guidance TTM in infants and children who are comatose after cardiac arrest to include in-hospital arrests with goal temperatures either 32 °C to 34 °C followed by a TTM of 36 °C to 37.5 °C or alternatively, a TTM of 36 °C to 37.5 °C.

In the last article, Kim et al. demonstrated in a simulated an infant CPR model that use of a metronome significantly increased the percentage of adequate chest compression rates, in both two-finger and two-thumb techniques, while maintaining adequate chest compression depth, unlike similar adult studies showing diminished chest compression depth.^54^

### Interdisciplinary Guidelines & Reviews (GL)

Guidelines help to promote known best-practices and identify important areas that need further research or quality improvement. Four articles scored highly in the interdisciplinary guidelines and reviews category, largely relating to intra-arrest management. The first, an AHA Focused Update on Systems of Care, had strong recommendations for dispatcher-assisted CPR based on observational studies including both instruction to initiate and real-time guidance in the performance of CPR implementation.^17^ Support for post-arrest care at specialized Cardiac Arrest Centers was given a moderate recommendation based on observational studies which showed improved rates of discharge with favorable neurologic outcomes though no difference at the 30-day mark.^17^ The next two studies focused on airway management and vasopressor use during cardiac arrest. Granfeldt et al. conducted a systematic review of studies comparing the effectiveness of airway management strategies but found significant risk of bias and heterogeneity between studies, thereby precluding the ability to perform a meta-analysis of the findings.^55^ As mentioned by the authors, this highlights the need for a more systematic approaches to assess best practices in airway management during cardiac arrest, thus leaving an important question unanswered. Holmberg et al. found through a systematic review and meta-analysis commissioned by the International Liaison Committee in Resuscitation (ILCOR) that standard 1 mg doses epinephrine improved survival after cardiac arrest, especially in patients with non-shockable rhythms and that neither vasopressin alone nor vasopressin in addition to epinephrine conferred additional benefit.^56^ A meta-analysis by Nas et al. that showed improved rates of ROSC, survival to admission, survival to discharge and favorable neurologic outcome following both the 2005 and 2010 guideline updates, illustrates the importance of continuous process improvement efforts in the field of cardiac arrest medicine, though there were few studies examining the effect of the most recent update in 2015 at the time of this study.^57^

## Limitations

The 2019 Interdisciplinary Cardiac Arrest Research Review has several limitations. First, the conclusions presented in this review are not designed to change professional practice. The aim of this literature review is to highlight the breadth of articles published in the field of cardiac arrest medicine and demonstrate the current state of this growing area of critical care research on an annual basis. An intrinsic shortcoming of this approach is that it does not provide extensive context, such as historical comparison as seen in a systematic review, or account for differences in systems of care. To address this concern, we provide article summaries and commentaries for additional context in Supplement 3. Second, while the methodology used to screen, score and select articles is designed to capture the most relevant published research in the field of cardiac arrest medicine, it is likely that this process still omits some high-quality publications that deserve attention in each category. Lastly, to remain objective in this literature search, this review did not include letters to the editor, commentaries, and other editorials that may provide important context to the articles selected.

## Conclusion

In its second year, the Interdisciplinary Cardiac Arrest Research Review screened more than a thousand articles related to cardiac arrest and after a rigorous scoring process, fully summarized 45 articles in seven different categories (Table 4). This year’s top-scoring articles were focused on the Epidemiology, Prehospital and In-Hospital Management of cardiac arrest. The total number of articles relevant to cardiac arrest demonstrate the need for an accessible guide that summarizes findings of quality research articles to serve as a reference for clinicians and scientists, as it is challenging to stay abreast of the growing body of cardiac arrest literature. ICARE’s goal is to further the development of the field of cardiac arrest medicine by highlighting the most methodologically sound and practice-changing articles each year.

## Conflict of interest

The authors have no relevant sources of funding to disclose.

## CRediT authorship contribution statement

**Travis W. Murphy:** Conceptualization, Data curation, Formal analysis, Methodology, Supervision, Visualization, Project administration, Writing - original draft, Writing - review & editing. **Scott A. Cohen:** Conceptualization, Data curation, Formal analysis, Methodology, Supervision, Visualization, Project administration, Writing - original draft, Writing - review & editing. **K. Leslie Avery:** Project administration, Writing - original draft, Writing - review & editing. **Meenakshi P. Balakrishnan:** Project administration, Writing - original draft, Writing - review & editing. **Ramani Balu:** Project administration, Writing - original draft, Writing - review & editing. **Muhammad Abdul Baker Chowdhury:** Project administration, Writing - original draft, Writing - review & editing. **David B. Crabb:** Project administration, Writing - original draft, Writing - review & editing. **Karl W. Huesgen:** Project administration, Writing - original draft, Writing - review & editing. **Charles W. Hwang:** Project administration, Writing - original draft, Writing - review & editing. **Carolina B. Maciel:** Project administration, Writing - original draft, Writing - review & editing. **Sarah S. Gul:** Project administration, Writing - original draft, Writing - review & editing. **Francis Han:** Conceptualization, Data curation, Formal analysis, Methodology. **Torben K. Becker:** Conceptualization, Methodology, Supervision, Writing - review & editing.
