# Supplementary material for: Cardiac arrest: An interdisciplinary scoping review of the literature from 2019
Source: Resusc Plus. 2020 Nov 4;4:100037. doi: 10.1016/j.resplu.2020.100037 (PMC8244427; doi:10.1016/j.resplu.2020.100037)
Supplement: Supplementary file 1 [file mmc1.docx]

**Interdisciplinary Cardiac Arrest Research Review**

**2019-2020 Reviewer Procedure Manual**

Revised *October 15, 2018*

**TABLE OF CONTENTS**

**Mission Statement Page 3**

**Introduction Page 4**

**Roles, Positions and Authorship Page 5**

**Expectations of Reviewers Page 7**

**Conflict of Interest Policy Page 8**

**Plagiarism Policy Page 10**

**Search Strategy Page 12**

**Screening Guidelines Page 13**

**Scoring Guidelines Page 20**

**Reviewing Articles Page 25**

**Appendix 1 Page 27**

**MISSION STATEMENT**

*The Interdisciplinary Cardiac Arrest Research Review (ICARE) aims to highlight and disseminate high quality cardiac arrest research. The goals of the review are to illustrate best practices, stimulate additional research, and promote further professionalization of field involving cardiac arrests.*

**INTRODUCTION**

Recognizing the need for a clear and accessible literature base to guide the growing research of cardiac arrest (CA), the Interdisciplinary Cardiac Arrest Research Review (ICARE) Group was formed in 2018. This review gathers together a number of articles from disparate sources, presenting the ones chosen as having specific relevance or value to one of the previously described domains of CA. We hope that it will continue to be a valuable resource, both for practitioners caring for cardiac arrest patients and academic researchers, for many years to come.

**ROLES, POSITIONS AND AUTHORSHIP**

**Editor-in-Chief:** Maintains overall responsibility for the review as a whole and represents the “public face” of the review to outside organizations. The editor-in-chief will chair all meetings of the editorial board and will be responsible for communicating regularly with the full editorial board regarding any new developments or major decisions regarding the review. The editor-in-chief works with the managing editor to select new reviewers each year based on the applications they submit. The editor-in-chief works closely with the managing editor and technical editor to produce the review each year and will have responsibility for filling both those roles if either becomes temporarily or permanently unavailable until such time as a replacement can be found. The editor-in-chief will be last author on the print version of the review.

**Managing Editor**: Maintains primary responsibility for ensuring that the review adheres to its predetermined timeline, working with individual editors to ensure that material is submitted correctly and on time. The managing editor will not directly supervise reviewers but will be responsible for taking on the role of an editor in supervising their reviewers if an editor becomes temporarily or permanently unavailable until a replacement can be found. The managing editor will work with the editor-in-chief to select new reviewers each year based on their applications. The managing editor will work closely with the technical editor to ensure that the mechanics of the review run smoothly at all times. In addition, the managing editor will have primary authorship of the print version of the review and will be listed as first author of the review.

**Assistant Managing Editor:** The Assistant Managing Editor work alongside the Managing Editor. He or she is elected from among the current group of editors and advisors. The Assistant Managing Editor will support and receive training from the current Managing Editor and will replace the Managing Editor at the end of his or her term. The Assistant Managing Editor will be listed as the second author on the review. A prerequisite for serving as assistant managing editor will include at least one year of superior prior service as an editor.

**Technical Editor**: Assists the managing editor in the technical aspects of review production, including performing the initial search, dividing up citations for screening, dividing up articles for scoring, creating tables and figures, etc. The technical editor will be listed as the third to last author on the review.

**Assistant Technical Editor**: Assists the Technical Editor in the review production including conducting the article search using defined search queries, reviewing citations for possible review inclusion, performing quality control procedures to ensure data integrity, assisting the development/management of databases, coordinating article assignments for screening and scoring, creating tables and figures for manuscript

**Editor**: Primary responsibility for supervising a small group of junior and senior reviewers, including ensuring the quality and timeliness of all material submitted by reviewers. ***If at any point a reviewer becomes temporarily or permanently unavailable, the editor will take over their responsibilities until such time as a replacement can be found.*** All editors will also participate as active members of the editorial board. All editors, including the Alternate Editor, will be listed as authors on the print version of the review, and the author positions will generally be in alphabetical order. Editors will be elected initially by a vote of the editorial board and will be given the opportunity each year thereafter to continue in their role, unless they are found by the editorial board to be derelict in their duties. The prerequisite for serving as an editor will include at least one (preferably two) years of superior service as a reviewer.

**Alternate Editor:** The Alternate Editor will be elected from among the senior reviewers. During their term, however, the Alternate Editor will serve as an assistant to a current editor, not as a reviewer. They will be ready to take over for any editor who is unable to complete their term and will receive priority when editor positions open up in future years.

**Advisor**: After serving for a full year as editor, an editor can choose to become an inactive editor, referred to as advisor. They will continue to be full members of the editorial board with the ability to vote in all editorial board decisions, but they will not be responsible for supervising reviewers during that year. They will be acknowledged in the print version of the review as members of the ICARE but will not be listed as authors. Each year, advisors will be given the chance to “reactivate” as editors prior to electing new editors from among the reviewers.

**Reviewer**: Reviewers will be primarily responsible for performing the screening, scoring, and writing up of individual reviews. They will be selected initially by the managing editor and editor-in-chief based on their applications and will be given the opportunity each year thereafter to continue in their role, unless they are found by their editor or managing editor to be derelict in their duties. Reviewers will be designated as junior reviewers during their first year on the review, and as senior reviewers each year thereafter. They will be acknowledged in the print version of the review as members of the ICARE but will not be listed as authors.

**Editorial Board**: The editorial board will have primary responsibility for making decisions for the ICARE. It will be chaired by the editor-in-chief, and will generally attempt to make decisions by consensus, though the editor-in-chief may choose to hold an official vote when necessary. All editors, including editor-in-chief, managing editor, and technical editor, and advisors will be full voting members of the editorial board.

**EXPECTATIONS OF REVIEWERS**

- This review is a collaborative effort between a teams of editors and reviewers. This requires a great deal of ongoing communication in order to ensure that everyone is working in concert. As such, **it is expected that reviewers will check their email at least daily** during the course of the review to ensure they are kept abreast of any new developments.
- **It is expected that reviewers will submit screening lists, scoring lists, and reviews on or before each of the deadlines assigned**. We cannot emphasize enough how important this is for the review. If one person is late with any of their assignments, it holds up the entire review. Even relatively brief delays can add up quickly, and too many delays may mean missing our deadlines. A detailed Google calendar will be sent to you. You can import this calendar into iCal as well. We recommend that you have all the deadlines in your calendar so that you can prepare for them in advance.
- If you anticipate being out of the country or on a busy rotation, it is important to plan ahead and complete your assignments ahead of schedule. **If you know in advance that you may have limited email access at any time, please contact your editor well in advance to work out a plan**. **If an emergency arises and you realize that you will not be able to make one of the deadlines, alert your editor right away so that alternative arrangements may be made.**
- The editors will be tracking when reviewers turn in assignments throughout the year. A consistent pattern of late assignments will count against a reviewer being invited back the following year. In fact, **late assignments are the number one reason why reviewers have not been invited back in years past.**
- We expect reviewers to ask for help early when they don't understand a step in the process or for any other reason, and to let their team editor know early if they will not be able to complete their assignment by a given deadline. **Asking for help is not a sign of weakness, and is strongly encouraged by this review.** While most questions can be resolved by email, editors will also be available by phone or skype to answer more complicated questions. If a reviewer feels they need more assistance than is available from their editor, they should next contact the Assistant Managing Editor, Managing Editor, and Editor-in-Chief (in that order).
- A brief note about authorship. The first author will be the managing editor, the second author will be the assistant managing editor, the last author will be the editor-in-chief, and the second and third to last authors will be the technical editors. The remaining author positions will generally be assigned to the other editors in alphabetical order. The managing editor and editor-in-chief *will have flexibility to promote an editor to a higher autho*r order if, and only if, they take on additional objective responsibility during the year, such as filling in for a reviewer who leaves mid-year. As members of the Interdisciplinary Cardiac Arrest Research Review Group, all reviewers will be listed in the acknowledgment section of the article. *Though limitations of most journals prevent us from listing all reviewers individually as authors, they will also typically be listed as collaborators in PubMed.* It is still appropriate for all reviewers to include this review as an official publication on their CV by stating that were a member of the study group.

## CONFLICT OF INTEREST POLICY FOR EDITORS AND REVIEWERS

*Adapted from the Annals of Emergency Medicine COI Policy*

**Purpose:**

To describe the policy and practice for management of editor and reviewer conflicts of interest involving *ICARE.*

**Policy:**

*ICARE* believes that all editorial board members, advisors, and reviewers acting on behalf of *ICARE* have a fiduciary duty to *ICARE*. A conflict of interest may occur when an editorial board member or reviewer has personal or outside financial, business, professional interests, or other responsibilities that conflict with their duties to *ICARE*, including decisions and reviews of manuscripts submitted to *ICARE*. Editorial board members and reviewers are required to declare to *ICARE* any actual or potential conflicts of interest.

**Practice:**

1. *Definitions*
   1. Conflict of Interest: Any situation or transaction in which a person has a direct or indirect interest such that he/she may realize a personal benefit from the situation or transaction. This includes decisions to publish or not publish a manuscript.
   2. Financial conflict of interest includes more than nominal compensation from an entity that has a financial interest or stake in the subject of a publication or other activity of *ICARE*. Nominal in value means no more than twenty-five dollars ($25.00). Direct interest includes ownership by you or a member of your immediate family, or an investment in a concept, product or another party developing those concepts or products. Indirect interests involve consulting or accepting any type of compensation for work involving the concept or product or any company involved in the concept or product. Indirect interest also includes receipt of external funding or grants from either commercial or governmental or nonprofit funding agencies, such as NIH and others.
   3. Non-financial conflict of interest includes personal (friendship, family members, co-workers, political, religious), and intellectual (competing research, fiduciary responsibilities) interests that would make a reasonable reader, author, reviewer, or editor feel misled or deceived if not disclosed. These include friendship with an author, interest in competing organizations, competing research interests, or political beliefs or religious beliefs that could be perceived by a reasonable reader of *ICARE* to interfere with your objectivity.
2. *Procedures*
   1. General: Annually, members of the *ICARE* editorial board participating in the review will provide information regarding their potential conflicts of interest. This will include a financial conflicts of interest statement, as defined above.
   2. Reviewers: If a reviewer is assigned an article to score or review in which there may be a potential conflict of interest as outlined above, they should notify their editor immediately. The editor will promptly review the potential conflict and determine whether the reviewer may continue to score or review the article or whether it should be reassigned to another reviewer. The editor will notify the managing editor in writing of their decision on this matter.
   3. Editors: If an article is assigned to an editor or one of their reviewers for scoring or review in which the editor believes they might have a potential conflict of interest, they should notify the managing editor immediately who will review the conflict and determine if the article should be reassigned to another editor’s team. Articles co-authored by the managing editor or editor-in-chief will not be included in the review.
   4. Complaints: A complaint regarding potential conflict of interest of a reviewer will be referred to the managing editor who will review the circumstances and send a written assessment of the merits of the complaint to the editor-in-chief. A complaint regarding potential conflict of interest of an editor will be referred to the managing editor who will review the circumstances and send a written assessment of the merits of the complaint to the editor-in-chief. The editor-in-chief will make a final decision on management of the conflict and a written summary will be sent to the *ICARE* Editorial Board for record keeping.

**PLAGIARISM POLICY FOR EDITORS AND REVIEWERS**

*Adapted from the John Hopkins Plagiarism Policy*

**Purpose:**

To describe the policy and practice for management of plagiarism committed by editors and reviewers of *ICARE.*

**Policy:**

*ICARE* believes that all editorial board members, editors and reviewers acting on behalf of *ICARE* have a duty to *ICARE* to prevent plagiarism. Plagiarism may occur when an editorial board member or reviewer takes another individual’s work and attributes the work as his or her own. Editorial board members and reviewers are required to declare to *ICARE* any occurrences of plagiarism and to uphold the ethical integrity of the review. The definition of plagiarism *ICARE* uses comes from the John Hopkins Bloomberg School of Public Health. The original source for the definition can be found at the following link:

*http://www.jhsph.edu/academics/degree-programs/master-of-public-health/current-students/JHSPH-ReferencingHandbook.pdf*

**Practice:**

1. *Definition*
   1. Plagiarism is defined as “…taking for one’s own use the words, ideas, concepts or data of another without proper attribution. Plagiarism includes both direct use or paraphrasing of the words, thoughts, or concepts of another without proper attribution. Proper attribution includes: (1) use of quotation marks or single-spacing and indentation for words or phrase directly taken from another source accompanied by proper reference to that source and (2) proper reference to any source from which ideas concepts or data are taken even if the exact words are not reproduced.” (The John Hopkins Bloomberg School of Public Health Policy and Procedures Memorandum Students-1 Academic Ethics: October 2006).
2. *Procedures*
   1. General: Members of the *ICARE* editorial board participating in the review will annually agree to *ICARE*’s plagiarism policy.
   2. Reviewers: All reviewers will receive *ICARE*’s policy on plagiarism prior to the start of the review and will be asked to affirm they have read and agreed to follow the policies regarding plagiarism.
   3. Editors: All editors will receive *ICARE*’s policy on plagiarism prior to the start of the review and will be asked to affirm they have read and agreed to follow the policies regarding plagiarism. Editors will be responsible for ensuring reviews are not only well written and accurate, but also adhere to our plagiarism policy. At a minimum, editors should compare the review to the original article to make sure that portions of the original article have not been directly copied. Editors will also be encouraged to run the reviews through an online plagiarism checking software, such as Viper (available free at [http://www.scanmyessay.com](http://www.scanmyessay.com/)) or another similar program of their choice.
   4. Reporting: Any incident of plagiarism committed by a reviewer should be reported to the managing editor who will review the circumstances and send a written assessment of the merits of the report to the editor-in-chief. The editor-in-chief will make a final decision on management of the situation and a written summary will be sent to *ICARE* staff for record keeping.

**SEARCH STRATEGY**

Each year, we search the medical literature for all publications in the past year pertaining to Cardiac Arrest. We do this search in two phases: the first phase includes January – May, and the second phase includes June – December. We use PubMed for our main search, specifying a series of “cardiac arrest” terms and “international” terms that we have refined over the years (see Table 1 below), along with specification on publication dates and article types.

We limit our PubMed search to articles published in English.

All the citations identified by our main PubMed search are divided into a series of Word documents. Each Word document will have a file name that is based either on the language of the citations in it (for our main PubMed search) or on the journal it came from (for our hand search). These Word documents are then distributed evenly among the reviewers, so that each reviewer has a similar number of citations to screen.

**Table 1: Current Search Terms for PubMed Search**

| **Cardiac Terms** | |
| --- | --- |
| Cardiac Arrest | Ventricular Tachycardia |
| Heart Arrest | Ventricular Fibrillation |
| Arrest | Sudden Cardiac Death |
| Cardiac | Cerebral Hypoxia |
| Asystole | Anoxic Brain Injury |
| Heart Attack | Ischemic Brain Injury |
| Cardiopulmonary Arrest | Cerebral Hypoxia |
| Resuscitation | Bystander CPR |

*((Heart Arrest[MeSH Terms]) OR (("heart arrest"[Title/Abstract] OR "cardiac arrest"[Title/Abstract] OR "heart attack") AND Title/Abstract OR Cardiac Arrest[Title/Abstract] OR Arrest, Cardiac[Title/Abstract] OR Asystol*[Title/Abstract] OR cardiac arrest[Title/Abstract] OR cardiopulmonary arrest[Title/Abstract])) AND (("2019/01/01"[PDAT] : "2019/12/31"[PDAT]))*

**SCREENING GUIDELINES**

The first task for the technical editors will be to screen the citation lists generated by our search in order to find articles that are truly relevant to Cardiac Arrest. The screening process is often the most confusing aspect of the review, partly because the boundaries of the field of CA are not clearly defined. For the purposes of this review, we will split up the field of CA into seven categories: **Epidemiology & Public Health (EPH),** **Prehospital Resuscitation, Technology & Care Processes (PRE), In-Hospital Resuscitation & Post-Arrest Care Processes (IN), Prognostication & Outcomes (PRO), Pediatrics (PED), Basic Science & Pharmacology (BSP),** and **Interdisciplinary Guidelines & Reviews (GL).** The types of articles to be included in each of these categories will be described below. If a citation does not clearly fall into one of these categories, then it does not belong in this review.

Once you receive your citation lists, you should divide them into two separate files and send these back to your editor **(by emailing them AND by uploading them to the appropriate folder in the Dropbox)**. The first file should include the original file name with the word KEEP at the end, and the other file should include the original file name with the word TOSS at the end. For instance, if the original file was named “H1 English8.doc”, the reviewer should return two separate files to their editor. The first would be called “H1 English8 KEEP.doc” and the second would be called “H1 English8 TOSS.doc”.

You should cut and paste citations from your original file into each of these two new files based on the screening criteria below. In general, the TOSS file should have 10-20 times as many articles as the KEEP file. This is because most citations will not be relevant to CA and should be placed in the TOSS file. The entire citation, including the PMID number at the very bottom should be included, as this will be important for the next phase of the review.

Below are a set of general rules for screening citations, followed by specific rules for each of our three categories of articles. Afterwards are examples of how to categorize two different citations. Please read the instructions below carefully and email your editor with specific questions.

General Rules for Screening Citations

1) The citations included in our review should fall into one of the following seven categories: **Epidemiology & Public Health (EPH),** **Prehospital Resuscitation, Technology & Care Processes (PRE), In-Hospital Resuscitation & Post-Arrest Care Processes (IN), Prognostication & Outcomes (PRO), Pediatrics (PED), Basic Science & Pharmacology (BSP),** and **Interdisciplinary Guidelines & Reviews (GL).** Below are listed specific examples of articles that would fit or not fit into each of these categories.

2) In general, we are looking for original research or review articles that contribute something new to the cardiac arrest literature base. Most letters to the editor, commentaries, editorials, news articles, case studies, or general descriptive reports should not be included in the review. When screening citations, a good rule of thumb is that *if the citation does not have an abstract, it probably does not belong*, no matter how interesting the title **(this does not apply to the grey literature screening!)**. Furthermore, if the abstract does not seem to describe an original research study or a review of original research studies, it also should probably be cut.

Specific Criteria for Each Category

**Epidemiology & Public Health (EPH)**

Keep:

- Citations on analyses and resources provided to the public for the better understanding of cardiac arrest incidence, etiology, epidemiology, preventions, and intervention.
- Public programs targeted at increasing the awareness of cardiac arrest and awareness of interventions for cardiac arrest.
- General perceptions of survivors and cardiac arrest (in general)
- Quality of life assessment of causes of cardiac arrest
- Diseases and disorders that increase risk of cardiac arrest
- Clinical interventions (e.g. surgical, ICD placement) that adversely induce cardiac arrest are included.
- Training of the public in CPR or cardiac arrest interventions
- Evaluations of specific drugs or drug classifications that increase risk for cardiac arrest

Toss:

- Programs directly involving the impact or improvement of bystander involvement in cardiac arrest are excluded (*PRE*)
- Quality of life assessment of survivors after cardiac arrest (*PRO)*
- Training of healthcare professions in prehospital cardiac intervention (*PRE)*

**Prehospital Resuscitation, Technology & Care Processes (PRE)**

Keep:

- Citations assessing the prehospital healthcare management from cardiac arrest onset to hospital admission.
- Assessments of bystander involvement (e.g. CPR, witnesses, dispatch-assistance) in the cardiac arrest treatment process
- Articles involving the interventions performed by emergency medical services
- Interventions that lead to improvement of return of spontaneous circulation (ROSC)
- Guidelines involving CPR and Basic Life Support
- Perceptions of emergency medical services, witnesses, and bystanders
- Evaluations of specific drugs or drug classifications during prehospital or intra-arrest care process

Toss:

- Interventions performed after post-arrest in the hospital setting *(IN)*
- Early indicators of cardiac arrest survival in the prehospital setting *(PRO)*

**In-Hospital Resuscitation & Post-Arrest Care Processes (IN)**

Keep:

- Citations assessing the in-hospital cardiac arrest healthcare management from hospital admission to discharge.
- Technological advances, interventions, and analyses that are primarily used in the in-hospital setting
- Surgical and procedural interventions in the in-hospital setting
- Articles involving experimental in-hospital technology in animal models
- Evaluations of specific drugs or drug classifications during post-arrest care process

Toss:

- Interventions performed in the prehospital setting are excluded (*PRE)*
- Indicators of cardiac arrest outcomes in the in-hospital setting are excluded *(PRO)*
- Results obtained from interventions or healthcare processes (e.g. labs, imaging results, biomarkers) to predict outcomes are excluded (*PRO)*
- Assessment of CPR in out-of-hospital cardiac arrest patients are excluded (*PRE)*
- Assessment of pharmacological interventions in non-human models (*BSP)*

**Prognostication & Outcomes (PRO)**

Keep:

- Citations assessing the ability to project survival, neurological, cardiac, or pulmonary outcomes in cardiac arrest patients
- Articles investigating the application of results (e.g. labs, imaging results, biomarkers) obtained from technology or interventions
- Articles characterizing patient demographics associated with particular outcomes
- Articles assessing long-term outcomes in cardiac arrest survivors
- Articles assessing health disparities in cardiac arrest outcomes
- Assessment of complications that results from cardiac arrest treatment

Toss:

- Articles investigating factors that predict cardiac arrest onset (*EPH*)
- Articles understanding cardiac arrest survivors’ public perceptions (*EPH)*
- Articles assessing the impact of an intervention on patient outcomes (*PRE or IN*)
- Novel biomarkers measured in non-human models (*BSP)*

**Pediatrics (PED)**

Keep:

- Citations on the causes, outcome, and interventions of cardiac arrests on individuals under the age of eighteen.

Toss:

- Citations involving primarily studying adult cardiac arrest.

**Basic Science & Pharmacology (BSP)**

Keep:

- Articles involving the pharmacodynamics or pharmacokinetics in non-human models
- Use of animal models or other basic science research relating to pharmacology
- Basic science research regarding metabolic changes pertaining to cardiac arrest in non-human models

Toss:

- Research involving the measurement of biomarkers without pharmacological intervention is excluded *(PRO).*
- Translational research involving the testing or feasibility of medical equipment in non-human models (*PRE or IN)*

**Interdisciplinary Guidelines & Reviews (GL)**

Keep:

- Citations presenting guidelines or review articles on the management or scientific understanding of cardiac arrest patients at an interdisciplinary level.
- Guidelines for methodology and study design for researching cardiac arrest topics
- Guidelines pertaining to pharmacological management that are not specialty specific

Toss:

- Guidelines presenting protocols specific in the prehospital setting (e.g. EMS systems) *(PRE)*
- Guidelines presenting protocol suggestions specific in the in-hospital setting (e.g. TTM) *(IN)*
- Guidelines presenting protocol suggestions specific to pediatric patients (*PED*)

EXAMPLES OF HOW TO SCREEN CITATIONS

EXAMPLE #1

Resuscitation. 2018 Apr;125:70-78. doi: 10.1016/j.resuscitation.2018.01.025. Epub 2018 Feb 3.

Gender aspects in cardiopulmonary resuscitation by schoolchildren: A systematic review.

Finke SR(1), Schroeder DC(2), Ecker H(2), Wingen S(2), Hinkelbein J(2), Wetsch

WA(2), Köhler D(2), Böttiger BW(3).

AIM: Bystander CPR-rates are embarrassingly low in some European countries. To increase bystander CPR-rates, many different approaches are used; one of them is training of schoolchildren in CPR. Multiple authors investigated practical and theoretical CPR performance and demonstrated gender differences related to schoolchildren CPR. The objective was to elaborate gender aspects in practical and theoretical CPR performance from the current literature to better address female and male students.

METHODS: A systematic search in PubMed-database with different search terms was performed for controlled and uncontrolled prospective investigations. Altogether, n = 2360 articles were identified and checked for aptitude. From n = 97 appropriated articles, n = 24 met the inclusion criteria and were finally included for full review and incorporated in the manuscript.

RESULTS: Female students demonstrated higher motivation to attend CPR-training

(p < 0.001), to respond to cardiac arrest (CA) (p < 0.01), scored higher in a CPR-questionnaire (p < 0.025), revealed better remembrance of the national emergency phone-number (p < 0.05) and showed a higher multiplier effect (p < 0.0001). Male students showed higher confidence in CPR-proficiency (p < 0.05), revealed deeper chest compressions (CC) (p < 0.001; p < 0.0015; p < 0.01), a higher CC-fraction (p < 0.01) and a higher arbitrary cardiac output simulated equivalent index (p < 0.05). Male gender could not be detected to be a predictor for higher tidal volume (p = 0.70; p = 0.0212).

CONCLUSION: In context of schoolchildren CPR, gender aspects are underestimated. Female students seem to be more motivated to attend CPR-training, reach more people in the role of a multiplier and need to be individually addressed in intensified practical training. Male students achieve a more sufficient chest compression depth and -fraction and could benefit from individual motivation.

Copyright © 2018 Elsevier B.V. All rights reserved.

DOI: 10.1016/j.resuscitation.2018.01.025

PMID: 29408490

EXAMPLE #2

Resuscitation. 2018 May;126:7-13. doi: 10.1016/j.resuscitation.2018.02.009. Epub 2018 Feb 12.

Late heartbeat-evoked potentials are associated with survival after cardiac arrest.

Schulz A(1), Stammet P(2), Dierolf AM(3), Vögele C(3), Beyenburg S(4), Werer C(5), Devaux Y(6).

RATIONALE: Cardiac arrest (CA) is a serious condition characterized by high mortality rates, even after initial successful resuscitation, mainly due to neurological damage. Whether brain-heart communication is associated with outcome after CA is unknown. Heartbeat-evoked brain potentials (HEPs) represent neurophysiological indicators of brain-heart communication. The aim of this study was to address the association between HEPs and survival after CA.

METHODS: HEPs were calculated from resting EEG/ECG in 55 CA patients 24 h after resuscitation. All patients were treated with targeted temperature management and a standardized sedation protocol during assessment. We investigated the association between HEP amplitude (180-320 ms, 455-595 ms, 860-1000 ms) and 6-month survival.

RESULTS: Twenty-five of 55 patients (45%) were still alive at 6-month follow-up. Survivors showed a higher HEP amplitude at frontopolar and frontal electrodes in the late HEP interval than non-survivors. This effect remained significant after controlling for between-group differences in terms of age, Fentanyl dose, and time lag between resuscitation and EEG assessment. There were no group differences in heart rate or heart rate variability.

CONCLUSION: Brain-heart communication, as reflected by HEPs, is associated with survival after CA. Future studies should address the brain-heart axis in CA.

Copyright © 2018 Elsevier B.V. All rights reserved.

DOI: 10.1016/j.resuscitation.2018.02.009

APPROACH TO EXAMPLE 2

By Scott Cohen, ICARE Technical Editor

Relevant to cardiac arrest?

*YES, Title indicates that of the article will focus on survival of cardiac arrest*

Is this article OR or RE?

*OR, methods indicate authors studied a 55 cardiac arrest cases. This article cannot be categorized in GL*

Does the article primarily focus on pediatric cardiac arrests?

*NO, there is no mention that the article specifically focuses on pediatric cases. This article is not PED category.*

If not PED, what category does this article screen?

*PRO. Although the article focuses on post-arrest patients and the use of medical equipment in the inhospital setting, it specifically involves the use of EEG/ECG results to predict survival in these patients. If the article focused on patients who had an EEG/ECG vs. those who did not, then it would be classified as IN.*

APPROACH TO EXAMPLE 1

By Scott Cohen, ICARE Technical Editor

Relevant to cardiac arrest?

*YES, Title indicates that of the article will focus on bystander CPR with regards to schoolchildren*

Is this article OR or RE?

*RE, Title indicates that it is a systematic review.*

Does the article primarily focus on pediatric cardiac arrests?

*NO, although articles highlights schoolchildren’s role in CPR, it is not specifically studying cardiac arrest in pediatric patients. This article is not PED category.*

If not PED, what category does this article screen?

*EPH. Although bystander CPR is clearly a prehospital intervention, it involves the training of the public in bystander interventions without the involvement of real cardiac events. If the article focused on gender aspects in EMS professionals, then it would be classified as PRE.*

**SCORING GUIDELINES**

At this stage, each reviewer will receive one list of citations that have been selected by the screening process for formal scoring.

The first task will be to find the full text PDF for each of the citations on the **assignment** list. **Reviewers will be responsible for uploading their articles into the appropriate Dropbox folder within the deadline of receiving their assigned lists of articles (see Timeline).** The majority of full text PDFs can be accessed using this PubMed link via an university account. Many of you will be familiar with accessing PubMed. For those who are not, it will be easiest to complete this process on an on-campus computer to be granted immediate access, otherwise you can use a VPN to gain access from an off-campus personal computer (vpn.ufl.edu for more information). **If reviewers have any trouble locating an article by one of these means, they should notify their editor immediately – well before the deadline for uploading articles**.

Once your articles are downloaded and saved, upload your files using your respective category-specific link. **Links will be provided when articles are assigned.** You are able to upload multiple files at once and you can start this at any time. These links are also located in the file named “Category_PDF_Upload_Links.xlsx” in Dropbox. You do not need a Dropbox account to upload your files and please make sure you use the correct link.

Editors will be responsible for scanning through the articles in the Dropbox folders uploaded by their reviewers and ensuring that they have: 1) actually uploaded all their articles on time, and 2) put them in the correct Dropbox folder. Then the reviewers will have several weeks to actually read their assignment lists.

Once articles are located and uploaded, begin the scoring process as soon as possible. You are asked to score every article on your assignment list, using the Scoring Criteria outlined in the Procedures Manual. To begin the scoring process, access the scoring template spreadsheets that are located in the “SCORING TEMPLATES” folder in the Dropbox.

Scoring of articles will be separated into original research articles and review articles. As the goal of the literature is to identify important work done in cardiac arrest over the preceding year, it is critical that we develop a system to assess and rank articles in an objective manner. Ideally, two separate reviewers should be able to read the same article and give it the exact same score, based on our scoring criteria. While there are many different scoring systems in existence for evaluating research articles, the heterogeneity of the articles contained in our search and the unique logistical concerns of CA have led us to develop our own scoring system.

**Original Research vs Review Articles**: To clarify, an original research (**OR**) article is a primary source. Typically, it is a scientific article written by the researchers who performed the study. The researchers describe their research question, purpose, methods, discussion, and conclusions (often under those headings). To contrast, a review article (**RE**) might include literature reviews, systematic reviews, meta-analyses, clinical guidelines, as well as articles about the ethics or conduct of research or education in an international setting. Both **OR** and **RE** articles can be placed in any of the 7 categories (EPH, PRE, IN, PRO, PED, BSP, GL) depending on their focus.

Each article will be scored across 4 separate categories and awarded points according to the subsections within each category. Subsections for each category are labeled as A, B, C, D, E. Please use the Scoring Guides within spreadsheet templates as reference. **Table 4** provides the specific elements to be used for scoring original research articles, while **Table 5** focuses on review articles. Do not award partial points: It’s either all in or all out. For example, if a category is weighted with two points, you will either give those two points if you think that the article meets the criteria listed. If it doesn’t, award zero points.

Example 1: If an RE article was a “formal meta-analysis that includes studies with a control group”, I would enter a “3” in subsection A in the “Design” category (Column K in excel) on the RE template.

Example 2: If an OR article lacked an informed consent process, I would enter a “0” in subsection B in the “Ethics” category (Column M in excel) on the OR template.

Please note that for articles in which there is a large discrepancy between reviewer scores, the editor will rescore the article independently. Afterwards, the score closest to the editor's score will be averaged with the editor's score to produce the final score, with the other reviewer’s score dropped (unless the editor score is perfectly in the middle, in which case all three will be averaged). The editor should provide feedback to reviewers as needed on the accuracy of their scoring during this process.

Guide for Evaluating Original Research Articles

For articles describing original research studies (including randomized controlled trials, observational studies, case series, cross-sectional surveys, and diagnostic studies) consider the following 4 measures of quality and impact listed in **Table 4**. The maximum possible score is 22 points. The best articles will have the highest scores. **If unsure about the answer to one of the questions, do not award the point(s).**

**Table 4: Scoring of Original Research (OR) Articles** (refer to PDF file labeled “PM_OR article”)

| **Quality Measure** | **Question** | | **Points** |
| --- | --- | --- | --- |
| **Design**  A | *Select*  *One* | Descriptive studies (including case studies and case series, natural observation studies and descriptive surveys) | **1** *-or-* |
|  |  | Correlation studies (case control studies, prospective observational studies, retrospective studies) | 2 *-or-* |
|  |  | Non-randomized or non-blinded experimental studies | 3 *-or-* |
|  |  | Randomized, blinded experimental studies | 4 |
| B | Study design is appropriate to answer the authors’ hypothesis. | | **1** |
| C | Correct statistical tests are used to analyze the data. | | **1** |
| D | Results are presented accurately and without bias. | | **1** |
| E | Limitations are clearly described, and the conclusions are supported by data. | | 1 |
| Design Total | **4** / Out of max score 8 | | |
| **Ethics**  A | The study was approved by an institutional review board (IRB)/institutional animal use and care committee, ethics committee, community group, as required by local laws. | | **2** |
| B | Informed consent was obtained or consent was waived by the IRB (*give point if not applicable, e.g., animal study*). | | **1** |
| C | The authors declare their conflicts of interest or declare that none exist. | | **1** |
| Ethics Total | **4** / Out of a max of 4 | | |
| **Importance**  A | The study results are not specific to one certain patient population but are broadly generalizable to a variety of settings. | | 2 |
| B | The topic being studied is an important one, in that it advances the field of cardiac arrest research or care. | | **2** |
| C | The study is clearly relevant to the realm of cardiac arrest research or care. | | **1** |
| Importance Total | **3** / Out of a max of 5 | | |
| **Impact**  A | The findings or recommendations of this study may be feasibly implemented by practitioners* of cardiac arrest care. | | 2 |
| B | Practitioners* would likely change their practice if they were aware of this study. | | 2 |
| C | The authors of this study raise interesting questions that may stimulate further research. | | 1 |
| Impact Total | **3** / Out of a max of 5  ***Practitioner: reader practicing in the category of the article (physician, epidemiologist, pharmacist etc.)** | | |

Guide for Evaluating Reviews

For review articles (including formal meta-analyses, systematic reviews, and descriptive reviews), consider the following 4 measures of quality and impact listed in **Table 5**. The maximum possible score is 22 points. The best articles will have the highest scores. **If unsure about the answer to one of the questions, do not award the point(s).**

**Table 5: Scoring of Review (RE) Articles** (refer to PDF file labeled “PM_RE article”)

| **Quality Measure** | **Question** | **Points** |
| --- | --- | --- |
| **Clarity**  A | The review has a clearly stated hypothesis or purpose. | 2 |
| B | The authors provide sufficient background to put the results of the review into context. | **1** |
| C | The review can be understood by someone with general medical or public health training. | **1** |
| D | The authors use clear language and appropriate graphs, tables, and figures throughout the article. | **1** |
| Clarity Total | **3** / Out of max score 5 | |
| **Design**  A | This is a formal meta-analysis or a systematic review that only includes studies with a control group. | 3 |
| B | There is a clear, reproducible method for the selection of studies included in this review. | 2 |
| C | Articles for this review were selected by at least two authors blinded to each other’s selection. | 1 |
| D | The data was aggregated and/or analyzed appropriately. | **1** |
| Design Total | **1** / Out of max score 7 | |
| **Importance**  A | The review is not specific to one certain patient population but is broadly generalizable to a variety of settings. | **2** |
| B | The topic being reviewed is an important one, in that it advances the field of cardiac arrest research or care. | **2** |
| C | This is clearly relevant to the realm of cardiac arrest research or care. | **1** |
| Importance Total | **5** / Out of max score 5 | |
| **Impact**  A | The findings or recommendations of this review appear to have applicability towards improving cardiac arrest research or care. | **2** |
| B | Practitioners* would likely change their practice if they were aware of this review. | 2 |
| C | The authors of this review raise interesting questions that may stimulate further research. | **1** |
| Impact Total | **3** / Out of max score 5  ***Practitioner: reader practicing in the category of the article (physician, epidemiologist, pharmacist etc.)** | |

**REVIEWING ARTICLES**

After the scoring is completed for both the first and second half of the year, the articles with the highest average score will be chosen for full review (usually about 20-30 articles). Each reviewer will be assigned between one to three of these articles, for which they will write a review. Each review should include a summary and comment section.

The summary section should include:

1. Objective of the article
2. Brief summary of the methods
3. Results of the article

The comment section should address:

1. Strengths of the article's design
2. Limitations of the article's findings
3. Importance of the findings to the field of CA
4. How the article fits into the context of prior research on the topic

Because each article will present its own challenges and raise specific questions during the review process, it is difficult to give precise instructions other than the general guidelines above on how to complete the reviews. We have included sample reviews below for two Review articles and two Original Research articles. New reviewers should also refer to the online supplement section for our prior published reviews for more examples. Editors should be contacted EARLY if reviewers have any questions at all about how to proceed or what to include in the write-up!

It is very important to follow our formatting template *exactly* as outlined below:

| OR vs RE  EPH vs PRE vs IN vs PRO vs PED vs BSP vs GL  <single blank line>  <bold> Author Last Name First Initials, et al *(include first author’s name only, no comma between last name and first name, but before “et al.”)*. Title. Journal Name *(abbreviated)*. Year; Issue: Page. (*last page abbreviated: 105 to 109 written as 105-9; 105 to 115 written as 105-15)* </bold>  <single blank line>  <italic> One-to-two-sentence summary.</italic>  <single blank line>  <bold>Summary:</bold> Summary of the article.  <single blank line>  <bold>Comment:</bold> Editorial assessment of the articles strength and  weaknesses, comment on context and importance  <single blank line>  <italic> Name of reviewer, name of supervising editor </italic> |
| --- |

Sample Reviews

REVIEW ARTICLES:

**RE**

**PRE**

**Lindsay PJ, et al. The efficacy and safety of pre-hospital cooling after out-of-hospital cardiac arrest: a systematic review and meta-analysis. Critical Care. 2018; 22:66.**

*One-to-two sentence summary*

**Summary:**

**Comment**:

*Name of reviewer, name of supervising editor*

ORIGINAL RESEARCH ARTICLES:

**OR**

**PRO**

**Weiser C, et al. Initial electrical frequency predicts survival and neurological outcome in out of hospital cardiac arrest patients with pulseless electrical activity. Resuscitation. 2018. 125:34-8.**

*One-to-two sentence summary*

**Summary:**

**Comment:**

*Name of reviewer, name of supervising editor*

**APPENDIX 1: EDITOR TERM LIMITS, EVALUATION AND TERMINATION**

I. Editor term limits

a) Editor – 3 years (1 year = 1 review cycle). This can be extended by taking on a leadership position such as chairing a committee for a maximum of 2 additional years.

b) Assistant Editor – 1 year (unless no Editor positions are available the following year, in which case the Assistant Editor will be allowed to renew again until an Editor position opens)

c) Assistant Managing Editor/Managing Editor – 4-year combined term, with two years spent in each role (must have served previously as an Editor).

d) Advisor – 8 years (must have served previously as an Editor). This can be extended by taking on a leadership position such as chairing a committee for a maximum of 2 additional years.

e) Editor-in-Chief – 10 years.

f) Assistant Technical Editor/Technical Editor – No term limit.

II. Exceptions to editor term limits

a) If the ICARE is unable to recruit enough editors, the term of the most junior editor(s) will be extended by 1 year. If there is only 1 position that needs to be extended beyond the regular term, but more than 1 editor eligible for this extension, the executive group consisting of the editor-in-chief, managing editor, assistant managing editor and technical editor will decide which editor’s term will be extended, taking editor interest and past performance into consideration.

b) Exceptions to this policy due to unforeseen or special circumstances may be made by simple majority vote of the Editorial Board.

III. Editor evaluation and termination

a) After the H1 and H2 phase, the Technical Editor will collect anonymous feedback on the editors’ performance from the reviewers. Submission of feedback will be mandatory. The Technical Editor will compile the feedback and share it with the Editor-in-Chief, the Managing Editor, and the Assistant Managing Editor who will review the feedback and share it with editors when appropriate/indicated.

b) The Managing Editor will monitor the Editors’ compliance with the ICARE procedures as described in the Procedures Manual as well as with regards to meeting the established deadlines. Editors who receive unfavorable reviewer feedback or fail to meet the ICARE procedures or deadlines (as defined by submission more than 1 day late) will be issued a warning letter via email by the Editor-in-Chief.

c) Editors are required to reply to the warning letter within 5 days. The Editor should explain what steps she/he will take to avoid any future occurrences of this problem. If the Editor refuses to respond to the complaint, she/he will be terminated from the position immediately. Any editor who has received two warning letters will be terminated from her/his position immediately upon receipt of an additional (third) complaint.

d) After the H1 and H2 phase, the Editor-in-Chief will collect feedback from the committee chairs regarding each Editor and Advisor’s contribution to the committee’s work throughout the year. Editors and advisors not fulfilling their duties on their assigned committee will receive a warning letter.

e) At the discretion of the Editor-in-Chief and Managing Editor, Editors and Advisors who receive any warning letters during the year may not be invited to return for the following year.
