# Supplementary material for: Cardiac arrest: An interdisciplinary scoping review of the literature from 2019
Source: Resusc Plus. 2020 Nov 4;4:100037. doi: 10.1016/j.resplu.2020.100037 (PMC8244427; doi:10.1016/j.resplu.2020.100037)
Supplement: Supplementary file 2 [file mmc2.docx]

**Supplement 2:**

**†**PubMed/MEDLINE search query 2018:

Heart Arrest[Mesh] OR ("heart arrest" OR "cardiac arrest" OR "heart attack") OR Cardiac Arrest OR Arrest, Cardiac OR Asystol* OR cardiac arrest OR cardiopulmonary arrest OR ventricular tachycardia AND(("2018/01/01"[PDAT] : "2018/12/31"[PDAT])) NOT (editorial OR "case report" OR "case reports" OR commentary OR comments OR "letter to the editor")

PubMed/MEDLINE search query 2019:

((Heart Arrest[MeSH Terms]) OR (("heart arrest"[Title/Abstract] OR "cardiac arrest"[Title/Abstract] OR "heart attack") OR Cardiac Arrest[Title/Abstract] OR Arrest, Cardiac[Title/Abstract] OR Asystol*[Title/Abstract] OR cardiac arrest[Title/Abstract] OR cardiopulmonary arrest[Title/Abstract])) AND (("2019/01/01"[PDAT] : "2019/12/01"[PDAT]))

**†**In the initial design of the first iteration of this project in 2018, ventricular tachycardia was included though this yielded many more articles not directly related to cardiac arrest. This nearly tripled the number of articles returned and lengthened the title/abstract screening process considerably without yielding any additional articles that scored at or even the near the cut-off for inclusion. For 2019, this term was not included, leading to a higher proportion of relevant articles on initial screening as the number of articles included for formal review was actually higher despite returning fewer on initial search (1214 included for review in 2018 and 1365 in 2019).
