# Supplementary material for: Cardiac arrest: An interdisciplinary scoping review of the literature from 2019
Source: Resusc Plus. 2020 Nov 4;4:100037. doi: 10.1016/j.resplu.2020.100037 (PMC8244427; doi:10.1016/j.resplu.2020.100037)
Supplement: Supplementary file 3 [file mmc3.docx]

OR

BSP

**Koziakova M, et al. Noble gas neuroprotection: xenon and argon protect against hypoxic–ischaemic injury in rat hippocampus *in vitro* via distinct mechanisms. Br J Anaesth. 2019; 123(5): 601–9.**

*Xenon and argon gas exhibit neuroprotective effect on hippocampal cells in vitro following oxygen-glucose deprivation model of cerebral ischemia. Helium, neon, and krypton are without neuroprotective effect.*

**Summary:**  Noble gases (NG) have garnered interest in recent years as neuroprotective agents following a primary cerebral insult (CA, TBI, HIE, etc). This is an *in vitro* experimental study evaluating the neuroprotective effect of helium, neon, xenon, argon, and krypton on murine hippocampal cell cultures exposed to oxygen-glucose deprivation (OGD). C57BL/6 mouse hippocampal slices were prepared and incubated for 14 days on a growth medium. Cell cultures were transferred to an experimental medium containing propidium iodide (PI) and assigned to sham, OGD control, and OGD + NG groups. PI fluorescence was measured at baseline. OGD was conducted for 30 minutes at 95% N_2_:5% CO_2_. Samples were subjected to normobaric helium (0.75 atm He) or mildly hyperbaric (+0.5 atm NG) xenon, argon, krypton, or neon. All groups were held at 37 ℃ and 20% O_2_:5% CO_2_. Helium was used to control for the effect of hyperbaric pressure in the OGD control group. 100 µM glycine was added to separate xenon and argon gas cohorts to evaluate for effect on N-methyl-D-aspartate receptor xenon/argon antagonism. Following 24 hours of NG treatment, hippocampal cell injury was quantified by PI fluorescence. Magnitude of cell injury was determined by maximal cell death simulated by overnight incubation in 70% ethanol at 4 ℃. Helium, neon and krypton gas demonstrated no neuroprotective effect. Mildly hyperbaric xenon and argon (0.5 atm partial pressure) were equally effective in neuroprotection. Glycine reversed neuroprotective effect of xenon, whereas argon was unaffected, suggesting that argon exerts its neuroprotective effect by an alternate mechanism.

**Comment**: Beyond targeted temperature management, there are few therapeutic options to mitigate neurologic damage following CA. NG inhalation provides a novel therapeutic approach to neuroprotection that appears promising in the CA population. In this study, murine hippocampal cells were deprived of oxygen and then exposed to NG. The relative injury reduction of xenon and argon gas on hippocampal cells is marked, however, the clinical effect is unclear. Both gases appear to have different mechanism of action in exerting neuroprotection. These findings are generally consistent with past research on xenon and argon, where both gases have been shown to exert variable neuroprotective effects depending on the mechanism of cerebral injury. The strengths of this study include clinically relevant parameters such as oxygen-glucose deprivation time, temperature control, and controlling for the effect of hyperbaric pressure. Limitations include a single time point for evaluation of the primary outcome, limited extrapolation to humans, and sample size.

*Colton Amaral, Sarah Gul*

OR

BSP

**Hoops et al. Selective Aortic Arch Perfusion With Fresh Whole Blood or HBOC-201 Reverses Hemorrhage-Induced Traumatic Cardiac Arrest in a Lethal Model of Noncompressible Torso Hemorrhage. J Trauma Acute Care Surg. 2019; 87(2): 263-73**

*Selective aortic arch perfusion (SAAP) with oxygenated fresh whole blood (FWB) has been previously shown to improve return of spontaneous circulation (ROSC). FWB is not always readily available in the pre-hospital setting, and it is not known if hemoglobin-based oxygen carriers (HBOC) are as efficacious. HBOC-201 is an isocalcemic substance that is stable at room temperature for up to three years. This study highlights how SAAP therapy with FWB or HBOC-201 in a swine model of HiTCA is (1) efficacious at achieving ROSC and (2) the possibility of converting to low-flow extracorporeal life support (ECLS) during management of HiTCA.*

**Summary:** There were three main objectives of the study: to determine whether SAAP with HBOC-201 demonstrated similar efficacy compared to FWB; to evaluate the feasibility of SAAP to ECLS conversion; and, to assess if SAAP therapy was able to reverse systemic physiologic derangements after CA. Twenty-six swine were subjected to HiTCA where CA was induced via a controlled, non-compressible torso hemorrhage surgery model. Animals were randomly divided into two groups (FWB or HBOC-201) and investigators were blinded to the randomization. Using Seldinger technique, animals were cannulated which allowed for monitoring and, later, cardiopulmonary resuscitation. The pharmacologic intervention of SAAP with either FWB or HBOC-201 was maintained until ROSC is achieved. End-tidal CO2 was employed as a surrogate marker for overall resuscitation effectiveness. Primary outcomes were rate of ROSC, survival post-intervention, conversion to ECLS, and correction of physiology. The rate of ROSC and short-term survival were comparable between SAAP therapy with FWB versus with HBOC-201. Most interestingly, ROSC was achieved in animals with asystole. All animals surviving the DCS phase were transitioned to ECLS. Both groups had physiological derangements, and the only significant physiological variance was pulmonary hypertension, a documented side effect of HBOC-201. In swine models post-HiTCA, ROSC was effectively achieved with SAAP, with use of either FWB or HBOC-201, but it did not prevent physiological derangements due to ischemic-reperfusion injury (IRI).

**Comment**: This study demonstrates the efficacy of SAAP at achieving ROSC, including in animals with asystole, and the feasibility of transitioning to ECLS after definitive haemorrhage control in swine models of HiTCA. Although human and swine anatomy and physiology are similar, there may be limitations in the translational nature of this study. The complexity of administering intra-aortic oxygenated FWB or HBOC-201 may also limit prehospital use of SAAP. Furthermore, SAAP did not effectively prevent physiological derangements caused by IRI. IRI negatively impacts neurological outcome and overall long-term survival. Further research using SAAP is warranted to find the balance between adequate haemorrhage control and preventing IRI.

*Morgan Carson-Marino, Sarah Gul*

OR

BSP

**Chonde M, et al. Intra-Arrest Administration of Cyclosporine and Methylprednisolone Does Not Reduce Post Arrest Myocardial Dysfunction. Biomed Res Int. 2019.** **2019:6539050.**

*Cyclosporin (CCY) and methylprednisolone (MP) may have the potential to control the effects of post arrest myocardial dysfunction in patients who have been successfully resuscitated.*

**Summary:** Following the return of spontaneous circulation (ROSC), many patients commonly experience complications with ventricular function and inflammatory responses that result in a post-arrest myocardial dysfunction (PAMD). It is thought that PAMD may be treated with a combination of drugs that target mitochondrial permeability transition pores, and immune responses, called cyclosporine (CCY) and methylprednisolone (MP) respectively. This study aimed to evaluate whether the combination of CCY+MP would reduce PAMD following ROSC compared to placebo, as well as its effectiveness in reducing inflammatory responses. During this experiment, 11 female swine were sedated and induced into ventricular fibrillation (VF) through the administration of an electric shock, who were then given mechanical chest compressions and administered either a combination of cyclosporine with methylprednisolone (n=5) or placebo (normal saline, n=6). The authors of this study found that subjects given CCY+MP had a higher mean arterial pressure following ROSC accompanied by a reduced cardiac output. However, the study did not find that the combination of CCY+MP was effective at reducing post arrest myocardial dysfunction.

**Comment**: This investigation was strengthened by a detailed method of inducing cardiac arrest and collecting several quantitative variables that assisted in the analyses of the post arrest care the swine subjects received. However, the study, with its intentions to be a pilot design, is still limited by small sample size and the reduced realism of ischemia during the induced arrest. It may be improved in the future by a larger sample size and a more severe model of injury that may result in a larger inflammation response for testing the efficacy of CCY+MP. Ultimately, PAMD is a common issue in cardiac arrest care, and can result in higher morbidity in those patients who need to be cared for following ROSC. Successfully management of PAMD following ROSC has the potential to significantly improve cardiac arrest survival outcomes, and previous studies have suggested that the combination of CCY and MP may be the solution. This study differs in that it was not able to support decreased PAMD in subjects that received CCY and MP, and such results are supported by Phase III trials of just cyclosporine.

*Kayvon Yazdanbakhsh, David Crabb, Karl Huesgen*

RE

BSP

**Gazmuri R, et al. Sodium-Hydrogen Exchanger Isoform-1 Inhibition: A Promising Pharmacological Intervention for Resuscitation from Cardiac Arrest. Molecules. 2019;24(9):1765**

*Coronary blood flow during CPR is poor which results in myocardial ischemia. The introduction of oxygenated blood to ischemic myocardial tissue results in further reperfusion injury. Cessation of coronary blood flow during CA leads to myocardial ischemia and accumulation of Ca^2+^. Excess cytosolic and mitochondrial Ca^2+^ results in cell injury due to oxidative phosphorylation, release of pro-apoptotic factors, and increased mitochondrial inner membrane porosity. Attenuation of myocardial reperfusion injury can improve CA outcomes.*

**Summary:** Cessation of coronary blood flow during CA leads to myocardial ischemia and activation of the sarcolemmal Na^+^-H^+^ exchanger isoform-1 (NHE-1), which drives accumulation of the Ca^2+^ via the Na^++^-Ca^2+^ exchanger, which leads to worsening myocardial injury. Experimental NHE-1 inhibitors in animal models of ventricular fibrillation (VF) have been shown to 1) preserve left ventricular distensibility which in turn preserves preload and forward flow, 2) reduce post-resuscitation episodes of VF which could prevent potential re-arrests, 3) lessen post-resuscitation myocardial dysfunction resulting in improved hemodynamic stability and thus improved survival in the early post-resuscitation period, and 4) attenuate adverse cardiac effects of increased myocardial oxygen consumption of epinephrine.

**Comment**: NHE-1 inhibitors may be beneficial for post-CA resuscitation in humans. So far, it has only been studied in patients with myocardial infarction (MI) undergoing intervention and during coronary artery bypass (CABG) in studies sponsored by pharmaceutical companies. Only one study in the MI group demonstrated cardioprotective effect, whereas in one of the CABG studies, reduction of post-operative MI was observed, but that study also had a higher rate of occlusive strokes, which was never previously demonstrated in animal models or other clinical studies, raising the thought that this adverse finding may be related to the mode of administration instead of NHE-1’s mode of action.

*Sarah Gul*

OR

EPH

**Heard D, et al.** **Hands-Only Cardiopulmonary Resuscitation Education: A Comparison of On-Screen With Compression Feedback, Classroom, and Video Education. Annals of Emergency Medicine. 2019; 6: 599-609.**

*Hands-only CPR training using a kiosk results in skill acquisition and performance similar to that of traditional classroom training; both superior to video training. Hands-only CPR can be used for regular training and retraining of laypeople to increase bystander CPR.*

**Summary:** This randomized, controlled study compared 3 different methods of hands-only CPR education, using immediate CPR performance scores from a Laerdal Mini Anne Feedback Manikin. Participants were employees and community members in the Denver, CO region who volunteered for the study. Subjects were randomized into 1 of 3 groups by colored cards: Facilitator-led, 30-minute, practice-while-watching, hands-only CPR classroom session (classroom session); On-screen 4-minute hands-only CPR tutorial with feedback manikin practice (kiosk session); 1-minute hands-only CPR video (video-only session). Primary outcome was the percentage (0-100) of compressions in which all measured components (depth, rate, and hand position) were correct during a 30-second compression test. Secondary outcomes were the percentage (0-100) of compressions in which depth was correct, rate was correct, and hand position was correct. 738 skills assessments were recorded across the 3 groups at the initial session and 326 skills assessments (44% of initial participants) were recorded at the 3-month follow-up. The video-only group had a significantly lower total score (–9.7) than the facilitator-led classroom group. No difference was detected between the classroom group and the kiosk group on total score. 3-month follow-up scores did not show superiority of any training method.

**Comment**: Early bystander CPR is imperative for increased neuro-intact survival chances of out-of-hospital cardiac arrest (OHCA) patients. Providing the public with cost-friendly, easily accessible, and effective CPR training is therefore of the utmost importance. This study supports the novel concept of using CPR training kiosks to increase bystander CPR training with the goal of increased bystander CPR being initiated on-scene. While the results of this study are promising, only 44% of the subjects returned for the 3-month follow-up, therefore the results showing that none of the training methods were superior should be interpreted with caution given the significant number of participants lost to follow-up. This could also suggest the need for retraining at 3 months to maintain the positive results from the initial training. Kiosk users had poorer performance on compression depth compared to classroom learners, indicating a potential area for kiosk improvement. However, kiosk users had more correct hand placement than classroom learners demonstrating a potential area of curricular improvement in the classroom. While not yet widely available, the data from this study as well as that gathered from already existing kiosks at airports and other locations nationwide support the novel kiosk hands-only CPR training approach as a potentially feasible and effective one for communities nationwide. This could have a great effect on the initiation of bystander CPR especially in rural communities where EMS response times may be longer and ultimately lead to an increase in neurologically intact OHCA survival.

*Yasmeen Elmelige, Meenakshi P. Balakrishnan, Muhammad Abdul Baker Chowdhury*

OR

EPH

**Hsieh Y, et al. Hypoglycaemic episodes increase the risk of ventricular arrhythmia and sudden cardiac arrest in patients with type 2 diabetes—A nationwide cohort study. Diabetes Metab Res Rev. 2020; 2: e3226.**

*Hypoglycemic episodes (HEs) in patients with type 2 diabetes increase the risk of ventricular arrythmia (VA) and sudden cardiac arrest (SCA) compared to those who do not experience HEs.*

**Summary:** This is a retrospective database study analyzing the risk of developing VAs (ventricular tachycardia (VT)/ventricular fibrillation (VF)) and SCAs in patients with newly diagnosed type 2 diabetes (T2D) and HEs. Patients aged 20 years or older and newly diagnosed with T2D type 2 diabetes from 2001 to 2010 or identified according to ICD 9 code. Study population was selected from the Taiwanese National Health Insurance Research Database. T2D was defined as those having experienced three outpatient visits or more with a diabetes diagnosis code over a 1-year period or at least one hospitalization with a diagnostic code for T2D. The hypoglycemic group included patients with T2D who experienced at least one HE. HE was defined as the presentation of symptomatic hypoglycemic coma or specified/unspecified hypoglycemia Requiring medical assistance in a hospital during the study period. The control group consisted of patients with T2D who had no HE during the study and were matched to the hypoglycemic cohort at a 4:1 ratio based on age and diabetes duration. 1037 patients were included in the HE group and 4148 in the control group. During the mean follow-up period of 3.3 ± 2.5 years, a total of 29 events occurred. Patients with one to two HEs experienced higher incidences of VA/SCA than those of controls (adjusted HR: 2.42; 95% CI, 1.02‐5.72; P = .04). The patients with more than two HEs showed even higher event rates than the controls (adjusted HR: 5.64; 95% CI, 1.57‐20.2; P = .008). In addition, the study found that patients using insulin had a significantly increased VA/SCA risk compared with those not using insulin (adjusted HR: 3.05; 95% CI, 1.31‐7.10; P = .01).

**Comment**: Cardiovascular disease (CVD) is the leading cause of death in patients with diabetes, with two to four times the risk of mortality compared with those without diabetes. Recent clinical trials have sought to reduce CVD mortality by implementing tighter control in these patients, but these studies have failed to demonstrate any significant benefit. This study showed that in a large sample, hypoglycemic episodes as well as insulin use were strong risk factors for VA/SCA. However, several limitations must be considered such as the sample size being from only Taiwan. Because the duration from HE to the first occurrence of VA/SCA was long in the hypoglycemic group, 3.4 ± 2.9 years, the patient's HE and subsequent VA/SCA may not have a temporal relationship. The hypoglycemic group patients had higher prevalence of cardiovascular (CAD and CHF) and cerebrovascular disease than the control group, which may indicate the that hypoglycemic group was a more complex population that was more prone to VA/SCA due to these comorbid conditions. Further studies are needed in various populations to validate these findings and delve deeper into the pathophysiology of the disease process and comorbidities in association with VAs/SCAs.

*Yasmeen Elmelige, Meenakshi P. Balakrishnan, Muhammad Abdul Baker Chowdhury*

OR

EPH

**Czarnecki, A, et al. Association Between Hospital Teaching Status and Outcomes After Out-of-Hospital Cardiac Arrest. Circulation: Cardiovascular Quality and Outcomes. 2019;12**

*Teaching hospital patients had more positive out-of-hospital cardiac arrest outcomes then those treated at a non-teaching hospital.*

**Summary:** Out-of-hospital cardiac arrest is a medical event that requires many resources. In this retrospective study, the main objective was to determine whether OHCA outcomes differ based on hospital teaching status. Inclusion for the study was nontraumatic OHCA’s who presented to an Ontario, Canada hospital. All patients had to be alive or were excluded. OHCA’s that occurred between April 7, 2007- March 31, 2014, were reviewed for inclusion. Patients were stratified by age; less than 65, 66 to 80, and 80 and over. In total, 29,972 charts were reviewed. Following inclusion and exclusion, a total of 25,346 were analyzed. 19,933 were from a community hospital while 5,413 were from a teaching hospital. The results of the study suggest that teaching hospital patients had more positive outcomes than those treated at a non-teaching hospital. The data suggests that teaching hospitals performed targeted temperature management (TTM) and cardiac catheterizations at a higher rate than non-teaching hospitals. Patients at teaching hospitals were also more likely to survive after being admitted to an intensive care unit and remain alive at 72 hours.

**Comment:** This study explored the impact of differences in utilization of resources between teaching hospitals as compared to community hospitals. The data from this study suggests that teaching hospitals are more likely to employ strategies shown to increase survival, namely TTM and cardiac catheterization. By extension, and shown in this study, the use of these measures was associated with increased survival though not necessarily causative. What was not considered in this study is the potential for differences in time to cooling, depth of temperature changes and rates of rewarming. Each of these variables regarding TTM have been or are currently being examined separately by larger trials though this study does provide more evidence that TTM is associated with a benefit. Another factor that bears consideration is the ‘halo effect’ of treatment at centers that have the resources and experience to provide TTM and regular cardiac catheterization. Other factors not included in the original data set such as levels of nursing care or presence of family to discuss goals of care early in the hospitalization could have had significant effects on survival in the first 72 hours after arrest.

*Emmett Martin, Meenakshi P. Balakrishnan, Muhammad Abdul Baker Chowdhury*

OR

EPH

**Nas J, et al. Effect of Face-To-Face vs Virtual Reality Training on Cardiopulmonary Resuscitation Quality: A Randomized Clinical Trial. JAMA Cardiol. 2020; 5(3): 328–35.**

*This randomized noninferiority trial compared virtual reality (VR) cardiopulmonary resuscitation (CPR) training to face-to-face training. VR training was shown to yield lower quality CPR than face-to-face training, with similar chest compression rate but inferior chest compression depth.*

**Summary:** Performance of CPR in the setting of sudden cardiac arrest (SCA) has been shown to be important in improving survival, but most individuals with SCA do not receive bystander CPR probably due to lack of CPR training in the general public. This randomized noninferiority trial aimed to examine the usefulness of the Lifesaver VR smartphone app, which could be used to increase the accessibility of CPR training. Participants were adult volunteers at the Lowlands Music Festival in the Netherlands. Trainees used VR goggles and headphones for an interactive experience that included performing chest compressions on a pillow while receiving feedback from the app. Face-to-face trainees received standardized training by an experienced and certified independent instructor using CPR manikins and training automated external defibrillators (AEDs). The primary outcome was CPR quality determined by compression depth and rate and the secondary outcome included scoring by blinded reviewers. 381 subjects were randomized to 20-minute VR (190) or face-to-face (191) training and 352 subjects (175 VR and 177 face-to-face) completed the training. At baseline there were a greater proportion of health care professionals in the VR group (P = 0.03). Analysis of primary outcome showed inferiority (P =0.99) of chest compression depth in the VR group, with a mean difference of -7.7 (95% CI: -9.4 to -6.0) mm and predefined noninferiority margin of -5 mm, and noninferiority of chest compression rate in the VR group (P < .001), with a mean difference of 5.7 (3.3 – 8.2) min^-1^ and predefined noninferiority margin of -17 min^-1^. Secondary outcomes also showed statistically significant inferiority of VR group in CPR scoring (P <0 .001).

**Comment**: Because most of the general population is not trained in CPR, studying VR and other technologies that can extend the accessibility of training to a larger audience are worth studying. This study shows that VR currently is still inferior to face-to-face training, however those trained with VR provided compression quality that could be improve survival in an SCA patient. The difference in performance between VR and face-to-face groups was also smaller in those with prior CPR training or healthcare experience, indicating the usefulness for retraining. The study however, had a population which was young and highly educated, which may not be representative of the general population. Because of the setting, 16% of participants had an alcohol level ≥0.5%, but passed a tandem gait test, but subgroup analysis showed no significant effect of alcohol between VR and face-to-face training groups. Given the limitations it is not possible to measure the impact of these training methods on SCA patient outcome. While VR CPR training is still inferior to face-to-face training, further research and development may find useful applications of VR.

*Jon Wiese, Meenakshi P. Balakrishnan, Muhammad Abdul Baker Chowdhury*

OR

EPH

**Berger C, et al. Combination of problem-based learning with high-fidelity simulation in CPR training improves short and long-term CPR skills: a randomised single blinded trial. BMC Med Educ. 2019; 19, 180.**

*This prospective, randomized single-blind interventional study finds that a problem-based learning (PBL) cardiopulmonary resuscitation (CPR) training leads to significantly improved CPR performance as compared with classical CPR training immediately following instruction. However, after six months the differences in performance are far less pronounced.*

**Summary:** High-quality CPR is essential to improving outcomes for sudden cardiac arrest (SCA) patients, but the optimal CPR training method is not clear. This interventional study aimed to determine if PBL with high fidelity simulation could lead to improved long-term CPR skills. Participants were fourth year medical students at the University of Muenster, Germany. Both intervention and control groups (blinded) received the same 30minute lecture on CPR followed by four days of small group teaching with tutors. The control group received classical CPR training using a CPR manikin in a non-simulated environment in which tutors interrupted and corrected students. The intervention group received a PBL-module and hands on CPR training in a simulated environment with no interruption or guidance. After training, randomly selected pairs of students from the same group completed a cardiac arrest simulation and evaluated by video. Six months later, an unannounced follow-up of the same scenario was completed by different random pairs of students from the initial group. 112 of 127 students enrolled in the study, 54 of whom were randomized to the control group and 58 to the intervention group. Fifty of the original subjects participated in six-month follow-up (22 control, 28 intervention). “Guideline conform CPR” was the primary endpoint, and performed significantly more by the intervention group during the initial evaluation (51.9% vs. 12.5%, p= 0.007), but improved performance by that group was not significant at six month follow-up (71.4% vs. 54.5%, p = 0.55). Percentage of sufficient compressions showed no significant difference at initial evaluation (intervention: 42.9 ± 34% vs. control: 41.2 ± 33% p = 0.98) or at follow up (intervention: 41.5 ± 31% vs. control: 45.1 ± 37% p = 0.93). After training, there was a significant increase in self-assessment scores of competence for both groups and a higher self-estimation of threshold reduction to perform CPR and improvement in CPR ability in the control compared to the intervention group.

**Comment**: PBL is a strategy that is prevalent in modern medical education. This study shows that a PBL CPR training method in medical students improves short term performance more than classical CPR training, while long term improvements are less significant. The decrease in participation (45% of original) at six-month follow up is a notable limitation of this study. Inability to perform all trainings and evaluations simultaneously may also have led to miscommunication, possibly impacting subject blinding. Because high-quality CPR is an important factor in improving SCA survival, optimal training methods are an important research subject, and PBL may be a strategy to improve training.

*Jon Wiese, Meenakshi P. Balakrishnan, Muhammad Abdul Baker Chowdhury*

OR

EPH

**Briard JN, et al. Automated External Defibrillator Geolocalization With a Mobile Application, Verbal Assistance or No Assistance: A Pilot Randomized Simulation (AED G-MAP). Prehosp Emerg Care. 2019; 3: 420-29**

*Verbally providing the location of the nearest public automated external defibrillator (PAED) to out-of-hospital cardiac arrest (OHCA) bystanders in a simulated environment seems effective in reducing the time to defibrillation compared to no assistance and to an automated external defibrillator (AED) geolocalizing mobile app.*

**Summary:** This was a Canadian based study designed as a three-armed, open, controlled randomized simulation trial whose objective was to evaluate the efficacy of verbal assistance from the emergency medical dispatcher (EMD) and through new mobile apps. Adult participants, English or French speaking, owning a mobile smartphone and without physical disabilities were stratified and block randomized by age, date of availability, and likelihood of coming across cardiac arrest frequently. The simulation site was a University campus location about 175 meters from the closest real PAED and stored in the entrance area of a nearby building. The primary outcome was time elapsed to shock and the secondary outcome was the total distance traveled by the participant. Of the 52 participants, 17 were randomized to the no assistance, 17 to verbal, and 18 to the mobile app groups. Time elapsed to shock was shorter in the verbal group (med 5:23 min, IQR 4:11–9:08) compared to the no assistance (med 10:00 min, IQR 7:49–10:00) and mobile app groups (median 9:44 min, IQR 6:30–10:00). A statistically significant difference in time elapsed between the verbal group and the no assistance and mobile app groups (p<=0.01) was noted. No differences were found between the groups in the distance traveled. Participants in the no assistance group reported greater difficulty in finding the PAED than participants from the other groups.

**Comment**: In response to low PAED utilization, communities have developed novel approaches to assist OHCA bystanders in locating life-saving devices, particularly through verbal assistance from the EMD and through new mobile apps but their efficacy has not been evaluated by comparative randomized simulation making this one of the first studies to do so. A limitation of the study is that participants from the verbal assistance group had a higher knowledge of the simulation study site geography, making this a confounding factor. However, the authors performed a post-hoc subgroup analysis showing verbal assistance provided an advantage to participants unaware of campus geography (not statistically significant). Among those familiar with campus geography, neither group outperformed another. EMD telephone/mobile app-assisted PAED localization could help optimize bystander response to OHCA, and this study highlights the need for further research. It also highlights logistical concerns such as the need for an additional available bystander, improving the external validity of findings and assessing practical aspects of such public defibrillator geolocalization strategies.

*Meenakshi P. Balakrishnan*

OR

EPH

**Bylow H et al. Self-learning training versus instructor-led training for basic life support: A cluster randomised trial. Resuscitation. 2019; 03 (026)**

*There is no statistically significant difference in practical skills or willingness to act in a real-life OHCA situation when comparing self-learning training with instructor-led training, six months after training in BLS.*

**Summary**: The primary objective of the study was to compare the effectiveness of basic two life support (BLS) training interventions by enrolling 1301 lay individuals in BLS training. The participants were randomized to either self-learning training or to traditional instructor-led training. Both groups used the Mini-Anne Kit and standardized film instructions. Study participants’ practical skills were measured on a Resusci Anne manikin and an AED trainer with the PC Skill Reporting system six months after the training. The Cardiff Test was used to determine adherence to the BLS algorithm as the main outcome of the study. The study did not find any statistically significant difference in the two groups.

**Comment:** This was a randomized control trial of BLS teaching strategies to determine whether there was a significant difference in skill retention between learners that attended self-led or instructor-led courses The study did not find any significant difference in the two groups at the six-month mark in terms of ability to perform CPR. The instructor-led course did have higher rates of self-assessed knowledge and willingness to act. Overall, this study found that while immediate skills were higher in the instructor-led group, this was not sustained at six-months. The higher level of self-assessed knowledge and willingness to act may serve to encourage instructor-led courses.

*Sabuj Chandra Bhowmick, Meenakshi P. Balakrishnan, Muhammad Abdul Baker Chowdhury*

OR

EPH

**Xu Y et al. An audio-visual review model enhanced one-year retention of cardiopulmonary resuscitation skills and knowledge: A randomised controlled trial. Int J Nurs Stud**. **2019.103451**

*Audio-visual and audio-visual-practice review models revealed better 12-month retention on cardiopulmonary resuscitation skills and knowledge for family members of people at higher risk of out-of-hospital cardiac arrest.*

**Summary:** The main objective of the study was to evaluate the effectiveness of audio-visual review model and audio-visual-practice review model on cardiopulmonary resuscitation skill after 12 months training. A randomized, double-blind, placebo controlled, and three-arm parallel study design was used to evaluate the effectiveness of the two models A total of 641 family members of patients at high risk of out-of-hospital cardiac arrest enrolled in the study and 448 participants completed the follow-up. All participants had initial cardiopulmonary resuscitation training and their cardiopulmonary resuscitation skill and knowledge were assessed immediately after training. Trainees who were rated “adequate skill and knowledge” were assigned randomly into one of three groups. The control group was given a cardiopulmonary resuscitation instruction booklet and a placebo-DVD without any reminders and other two groups were reminded in every 3 months over the phone. At 12 months trainees’ cardiopulmonary resuscitation skills and knowledge were re-assessed. The result showed that both intervention models outperformed the control group at 12 months after the training.

**Comment:** The study was a double-blind, randomized, placebo-controlled trial of different methods for teaching bystander CPR. This study design is compelling in that it simultaneously compares three different approaches to bystander education and included follow-up assessments after one year as opposed to the three- or six-month follow-up periods in similar studies. Both the ‘audio-visual’ and ‘audio-visual-practice’ reminder models outperformed the control which consisted of an educational booklet given to families of cardiac arrest victims upon discharge.

*Sabuj Chandra Bhowmick, Meenakshi P. Balakrishnan, Muhammad Abdul Baker Chowdhury*

OR

EPH

**Haukilahti MA et al. Sudden Cardiac Death in women. Circulation. 2019; 139(8):1012-1021.**

*The study demonstrated that women, while less likely to suffer sudden cardiac death (SCD) in the pre-menopausal and early postmenopausal years, still have a significant risk of SCD, though less likely ischemic in etiology.*

**Summary:**

The objective of the study was to explore the autopsy findings and causes of sudden cardiac death (SCD) among the Finnish population in the selected area with SCD deaths. This is a cohort-based study with SCD in northern Finland consist of 5869 study subjects. The study found that the average age of SCD was significantly higher among women than men. Ischemic heart disease was the prime cause of death for both sexes, though patients that died from nonischemic heart disease were more likely to be women.

**Comment**: Sudden cardiac death is the major cause of death among women and men. This study found that while heart disease and SCD affected women and men both significantly, the rate of ischemic heart disease was lower in women. This study also found that while women were more likely to have markers of left ventricular hypertrophy on ECGs obtained prior to SCD, about one-third were normal, suggesting a need to risk stratify cardiac risk with more than just ischemic disease taken into account.

*Sabuj Chandra Bhowmick, Meenakshi P. Balakrishnan, Muhammad Abdul Baker Chowdhury*

RE

EPH

**Gonzalez-Salvado, V, et al. Training adult laypeople in basic life support. A systematic review. Revista Española de Cardiología (English Edition). 2020; 73(1): 53-68**

*According to the findings of this systematic review, there is no single, gold standard for teaching BLS to adult learners.*

**Summary:** Training non-medical professionals in basic life support (BLS) has been crucial in expecting positive outcomes for those who have experienced out-of-hospital cardiac arrest. Methods for teaching BLS vary by time to train, techniques used, and resources needed. In an effort to accurately measure the effectiveness and success of multiple BLS strategies for non-medical professionals, a systematic review of relevant literature was conducted. The aim of this review was to incorporate key factors of BLS training to create a gold standard. To be included in this systematic review research studies and/or interventions with non-medical professionals were analyzed. The primary outcome measures were 1) performance of BLS skills, and/or 2) BLS skills retention, measured at least once by means of a practical test with objective quality measurement. In total, 1263 titles were found from various sources. The final analysis included 27 titles. 37% were randomized control trials. The conclusion of this review revealed that the method of training could play a role in BLS skill acquisition and retention. A limitation of the study was that the literature search only included one database. The results of the systematic review were not sufficient to identify a gold standard for BLS education. For effective BLS teaching should focus on skill retention, feedback from learners, and hands-on methods.

**Comment:** Although the principles of basic life support (BLS) are standardized, training strategies vary. With this variation, there has not been a single method that has been shown to result in better skill retention than the others. Based on the findings of this review, courses led by instructors, with hands-on components and feedback devices with a frequent retraining schedule seemed to yield better results. These findings are instructive for designers of BLS curricula and can serve as a recommendation for what has been shows to be effective in the past even though a true ‘gold-standard’ has not been identified.

*Emmet Martin, Meenakshi P. Balakrishnan, Muhammad Abdul Baker Chowdhury*

RE

EPH

**Chen K-Y, et al. Interventions to improve the quality of bystander cardiopulmonary resuscitation: A systematic review. PLoS ONE. 2019; 14(2): e0211792.**

*This systematic review finds bystander cardiopulmonary resuscitation (CPR) quality is improved with assistance by telephone dispatchers, compression only instructions, mobile apps, and real-time feedback devices. Mobile device and video use may delay initiation of chest compressions, however.*

**Summary:** High quality bystander CPR has been shown to improve outcomes in patients with sudden cardiac arrest (SCA). This systematic review following PRISMA guidelines aimed to determine interventions associated with improved layperson CPR quality. The authors searched Ovid MEDLINE, Ovid EMBASE, EBSCO CINAHL, Ovid PsycInfo, Reuters SCI-EXPANDED, and the Cochrane CEN-TRAL for articles published from 1966 – October 5, 2018 using the key concepts: cardiac arrest, bystander/layperson, and CPR quality. Two reviewers conducted an initial title and abstract screen of 2703 unique articles followed by a full text review of 152 articles, which led to 42 articles included in the study. Interventions in included studies were grouped into three categories: modifications to dispatcher-assisted CPR (DA-CPR), different methods to perform CPR, and additional aids to bystander CPR. The following interventions were shown to improve CPR quality in at least five studies: telephone DA-CPR, video-assisted DA-CPR, simplified telephone DA-CPR instructions, compression-only CPR, and use of real time-feedback devices. Mobile phone software showed improved CPR quality in two studies, and opposite side two-rescuer CPR, use of heel instead of hands, providing rescuer with a basic life support (BLS) flowchart, and use of M730 ventilator showed improved metrics of CPR in one study each. Video-assisted DA-CPR and mobile phone software each showed increased time to compression in more than one study.

**Comment**: SCA is a significant cause of morbidity and mortality in the general population and can often occur in settings where there are no medical professionals. This systematic review identifies multiple interventions that improve CPR quality by non-medical personnel that can be used in the event of emergency SCA. This study presents numerous low-cost and feasible interventions which could be easily implemented in the community, such as telephone DA-CPR and use of compression-only CPR. It is also important to note the limitations of this study. None of the included studies were performed in real-life resuscitation situations, limiting direct linkage to patient outcomes. There was also variability in measurement of CPR quality in each study. Each study included was also found to have had a high risk of bias in the blinding process. Further research is needed to determine which interventions can be used most effectively in practice.

*Jon Wiese, Meenakshi P. Balakrishnan, Muhammad Abdul Baker Chowdhury*

RE

GL

**Nas J, et al. Meta-Analysis Comparing Cardiac Arrest Outcomes Before and After Resuscitation Guideline Updates. Am J Cardiol, 2020;125: 618-29.**

*Updates to cardiopulmonary resuscitation (CPR) guidelines in 2005 and 2010 are associated with improved rates of return of spontaneous circulation (ROSC), survival to admission, survival to discharge, and favorable neurologic outcome.*

**Summary:** Cardiopulmonary resuscitation (CPR) is essential to the management of sudden cardiac arrest, and guidelines for best CPR practices have evolved over the years. Although several studies have examined changes in outcomes based on changes in guidelines, no study provides a comprehensive analysis of all available data. The current study overcomes this limitation by providing a meta-analysis of studies on CPR outcomes across a near 20-year period. Authors searched PubMed, Web-of-Science, Embase, and the Cochrane Libraries for original studies published since 2000 on differences in outcomes based on updates in guidelines in 2005, 2010, and 2015. Studies were limited to full manuscripts or published abstracts and those that included adult participants. Outcomes included return of spontaneous circulation (ROSC), survival to admission, survival to discharge, and favorable neurologic outcome. Thirty-four studies met criteria for review. Studies on outcomes comparing use of the 2000 v. 2005 guidelines (*n* = 23) showed significantly higher ROSC, survival to admission, survival to discharge, and favorable neurologic outcome when using the updated guidelines. Studies on outcomes comparing the use of 2010 v. 2005 guidelines (*n* = 11) showed improved survival to discharge but no difference in ROSC when using the updated guidelines. There were insufficient data to compare use of the 2015 v. 2010 guidelines.

**Comment**: As cardiopulmonary resuscitation (CPR) is an essential therapy for both in- and out-of-hospital cardiac arrests, regular monitoring of effectiveness and corresponding updates to guidelines are warranted. Overall, authors found improved outcomes after the 2005 and 2010 resuscitation guideline updates. Limitations include lack of randomization in examined studies, inclusion of published abstracts which do not allow for thorough assessment of methodology and risk of bias, and few studies available to examine changes in outcomes based on the most recent guideline update (2015).

*Daniel Ralston, Ramani Balu*

RE

GL

**Granfeldt A, et al. Advanced airway management during adult cardiac arrest: A systematic review. Resuscitation. 2019; 139: 133-43.**

*Despite a need for clarity on the most effective airway management strategy, current studies have high risk of bias and significant heterogeneity which precludes a systematic examination and conclusion on best practice.*

**Summary:** Airway management is key for treatment of cardiac arrests and although multiple strategies are accepted as effective, there is limited empirical evidence to support one over another. Bag-mask ventilation is often used as a first-line approach but may be limited in providing appropriate ventilation and oxygenation. Advanced management techniques, specifically tracheal intubation and supraglottic airway devices are used widely but also have demonstrated limitations in effectiveness. With the goal of creating the first evidence-based recommendation for clinical practice, the authors of this study conducted a systematic review of studies comparing effectiveness of airway management strategies. Authors searched Medline, Embase, and Evidence-Based Medicine Reviews for studies published on use of at least one specific airway management method on adults who experienced cardiac arrest in- or out-of-hospital. There were no limitations on publication year or language. Both observational and controlled (randomized or non-randomized) studies were included. Studies must have compared two advanced airway management techniques (i.e., tracheal intubation v. supraglottic airway device) or one advanced method compared to bag-mask ventilation. Outcomes for observational studies included short-, mid-, and long-term survival. Controlled trials included these outcomes as well as 72-h survival, neurological outcome up to 1-month post-discharge, success rates for advanced airways, ventilation success rates, regurgitation/ aspiration, and cardiopulmonary resuscitation (CPR) quality. Searches and a series of systematic screening strategies resulted in 11 controlled trials and 78 observational studies for review. Risk of bias for each study was assessed using the revised Cochrane risk-of-bias tool and the ROBINS-I tool. Examination of observational studies revealed high levels of risk of bias and heterogeneity between studies. Examined of controlled trials similarly revealed high levels of risk of bias and heterogeneity between studies. Thus, no meta-analysis was performed for either group of studies.

**Comment:** A limitation of the current study was low inter-rater agreement in initial screening of articles by title and abstract. Identified studies in this systematic review also had significant risk of bias and heterogeneity which precluded authors from drawing meaningful conclusions on best practice for airway management. As the authors note, future studies of airway management should aim to be systematic in randomization of condition, limit deviations from the intended intervention, and be cautious of selective reporting outcomes

*Daniel Ralston, Ramani Balu*

RE

GL

**Panchal AR, et al. 2019 American Heart Association focused update on systems of care: dispatcher-assisted cardiopulmonary resuscitation and cardiac arrests centers. Circulation. 2019; 140: e895-e903**

*Dispatcher-assisted CPR is associated with improved survival and neurologic outcomes after out-of hospital cardiac arrest. Post-arrest care in specialized cardiac arrest centers may also improve survival and outcomes.*

**Summary:** This update on systems of care from the American Heart Association reviews the evidence regarding the association of dispatch-assisted cardiopulmonary resuscitation (DA-CPR) and specialized cardiac arrest centers (CACs) for post-arrest care with outcomes after OHCA. DA-CPR is defined as the use of real-time coaching by an individual at an emergency call center to a bystander providing CPR at the scene of an OHCA. A CAC is defined as a specialized center that can provide comprehensive post-resuscitation care after OHCA, including cardiac catheterization, targeted temperature management, and post-arrest neuroprognostication.

The panel provided a Class IC-LD (Strong recommendation with limited supporting data from observational studies) in favor of the use of DA-CPR. Specifically, the panel recommended that emergency dispatchers both instruct bystanders to initiate CPR and provide real-time guidance on CPR implementation. Survival to hospital discharge and neurologic outcome at both hospital discharge and 1-month post-discharge were improved when the dispatch center offered DA-CPR compared with dispatch centers that did not offer DA-CPR. Patients who actually received DA-CPR had improved survival and neurologic outcome when compared with patients who did not receive DA-CPR. There was no difference in survival to good neurologic outcome in patients who received non-assisted bystander CPR compared to those that received DA-CPR; however, offering DA-CPR was associated with a 5-fold increase in the rate of bystander CPR.

A Class 2aC-LD (moderate recommendation with limited supporting data from observational studies) was given in support of the use of specialized CACs. Observational studies showed at treatment at a CAC was associated with improved survival to hospital discharge with favorable neurologic outcome; however, there was no difference in survival with favorable neurologic outcome at 30 days.

**Comment:** Coordinated systems of care are essential for maximizing survival and favorable outcomes after OHCA. This update provides evidence to support the use of DA-CPR and provide post-arrest care at specialized CACs; however, the evidence is based on limited data from observational studies.

*Ramani Balu*

RE

GL

**Holmberg MJ, et al. 2019. Vasopressors during adult cardiac arrest: A systematic review and meta-analysis. Resuscitation. 2019; 139: 106-121**

*Standard dose (1 mg) epinephrine boluses improve survival after sudden cardiac arrest, especially in patients with non-shockable initial rhythms. Vasopressin and vasopressin plus epinephrine do not appear to improve survival compared to epinephrine only.*

**Summary:** Bolus dose vasopressors are commonly used during resuscitation after sudden cardiac arrest, especially in patients with non-shockable rhythms. However, the evidence supporting this practice is limited and evolving. This systematic review and meta-analysis was commissioned by the International Liaison Committee on Resuscitation (ILCOR) to evaluate the evidence supporting the use of vasopressors in cardiac arrest and provide an update to previously published 2015 guidelines. The authors evaluated studies comparing (1) epinephrine to placebo, (2) vasopressin compared to epinephrine, and (3) vasopressin plus epinephrine to epinephrine only. Studies comparing high dose (≥ 0.2 mg/kg or 5 mg) to standard dose (1 mg) epinephrine were not reviewed, since no new controlled trials comparing epinephrine doses had been performed since the 2015 guidelines.

Fifteen controlled trials were reviewed. Two were used for a meta-analysis comparing epinephrine to placebo, 3 were used for a meta-analysis comparing vasopressin to epinephrine, and 3 were used for a meta-analysis comparing vasopressin plus epinephrine to epinephrine only. Epinephrine was associated with improved return to spontaneous circulation (ROSC), survival to hospital admission, and survival to hospital discharge when compared to placebo. Improvements in ROSC and survival to hospital discharge with epinephrine were most pronounced in patients with non-shockable initial rhythms. There was no difference in the proportion of patients with favorable neurologic outcome at discharge between patients treated with epinephrine versus placebo. However, in one study, the proportion of survivors with unfavorable neurologic outcome was higher with epinephrine versus placebo.

Vasopressin did not improve ROSC, survival to hospital admission, survival to hospital discharge, or discharge neurologic outcomes when compared to epinephrine. Finally, the combination of vasopressin plus epinephrine did not improve ROSC, survival to hospital admission, or survival to hospital discharge when compared with epinephrine only.

**Comment:** This systematic review and meta-analysis provides support for the use of standard dose epinephrine during resuscitation after sudden cardiac arrest to improve survival. The effects of epinephrine on neurologic outcomes; however, are unclear and require further study.

*Ramani Balu*

OR

IN

**Beck S, et al. Basic life support training using shared mental models improves team performance of first responders on normal wards: A randomised controlled simulation trial. Resuscitation. 2019;144:33-9.**

*A randomized controlled simulation trial demonstrated that Basic Life Support (BLS) training focusing on team-based learning and group interaction reduces hands-off time.*

**Summary:**  The objective of this trial was to evaluate BLS training that focused on developing shared mental models as a method for improving in-hospital cardiac arrest resuscitation. Investigators randomized participants into a control or intervention group and blinded both participants and assessors. The intervention group received specialized training that focused on teamwork and developing a shared mental model. This included items such as group discussions and feedback from other participants. Furthermore, the intervention group instructor focused on encouraging self-directed learning as opposed to traditional teaching. A team assessment scale demonstrated no significant difference between the two groups. However, the hands-off time for the intervention group was statistically significant.

**Comment**: This trial boasts multiple strengths including randomization and blinding. Furthermore, the studied training methods demonstrate evidence of improved cardiac arrest care without increasing training time or costs. However, both groups scored very highly on the chosen team assessment tool, limiting the usefulness of the respective data. Improving cardiac arrest care involves not only developing and evaluating interventions, but also how that care is delivered. Overall, this trial takes strides in identifying ways to improve cardiac arrest care by studying the training of healthcare professionals.

*Garrett M Snipes, Charles W Hwang*

OR

IN

**Goharani R, et al. Real-Time Compression Feedback for Patients With In-Hospital Cardiac Arrest: A Multi-Center Randomized Controlled Clinical Trial. J Intensive Care. 2019;7:5.**

*Use of the Cardio First Angel™ (CFA), a hand-held chest compression feedback device, during in-hospital cardiac arrest improves patient survival and hospital discharge.*

**Summary:**  This multicenter, prospective, randomized controlled trial investigated if use of the CFA device during CPR for in-hospital cardiac arrest would lead to improved patient outcomes when compared to standard CPR. Improved patient outcomes were measured by a primary outcome of sustained ROSC (> 30 min) and secondary outcomes of survival to ICU and hospital discharge. Inclusion criteria included age ≥ 18 years, admission to the ICU from the emergency department (ED), full code status, and informed consent. Patients were excluded if pregnant. Randomization occurred in the ED, and enrolled patients were randomized to receive either standard CPR or CPR performed with real-time feedback using the CFA device. Subjects were excluded from the final study due to any out-of-hospital cardiac arrest or ED cardiac arrest prior to study enrollment, change in code status, revoked consent, or lost or incomplete data. Both groups were found to be similar with respect to demographics and resuscitation variables (e.g. ROSC, first shock success, initial rhythm, CPR duration), as well as resuscitation medications.

The results of the study showed the CFA group had improved sustained ROSC (66.7% vs. 42.4%, *p* < 0.001), improved survival to ICU discharge (59.8% vs. 33.6%, *p* < 0.001), and improved survival to hospital discharge (54% vs. 28.4%, *p* < 0.001).

**Comment**: It is largely accepted that proper compression technique is the most important factor for successful CPR. Using Audio Visual Feedback (AVF) devices during CPR to ensure proper compressions are delivered has thus been identified as a strategy which could improve cardiac arrest outcomes. Several AVF devices are now available for this reason. A prior meta-analysis reported improved quality of CPR, but not ROSC, with automated external defibrillator (AED) -associated AVF devices. At that time, there were no studies available for non-AED AVF devices. Subsequently, a trial by Vahedian-Azimi, et al. determined that the use of the CFA during in-hospital arrest improved rates of ROSC, CPR guideline adherence, and CPR quality.

This article evaluates whether the use of the CFA would lead to improved patient outcomes. The study was carefully designed and is larger in scale than many other similar articles regarding use of AVF devices. It is also the first of its kind in looking specifically at patient outcomes with a hand-held non-AED device. The study found that the CFA improved patient outcomes when a patient suffered from an in-hospital cardiac arrest. Moreover, the CFA is an affordable AVF device. One limitation discussed by the authors is that patients with primary cardiac conditions were admitted to the cardiac ICU and thus were not enrolled. A second limitation is that the study was not designed to follow neurologic or functional outcome. As stated in this paper, additional research should aim to compare the CFA to other AVF devices to clarify performance characteristics and potential benefits of these devices.

*Kathryn Dasburg, Charles W Hwang*

RE

IN

**Gottlieb M, et al. Beta-blockade for the treatment of cardiac arrest due to ventricular fibrillation or pulseless ventricular tachycardia: A systematic review and meta-analysis. Resuscitation. 2020;146:118-25.**

*Three studies demonstrated that when initial advanced cardiac life support (ACLS) measures fail in cardiac arrest patients with ventricular fibrillation or pulseless ventricular tachycardia, the use of beta-blockers may increase the likelihood for temporary or sustained return of spontaneous circulation (ROSC), survival-to-discharge, survival-to-admission, and favorable neurologic outcome. Further randomized controlled trials are needed to evaluate these findings.*

**Summary:** The primary objective of this article was to assess for a relationship between beta-blockers and the rate of ROSC in patients with ventricular fibrillation or pulseless ventricular tachycardia after failure of initial ACLS measures. Other endpoints included survival-to-admission, survival-to-discharge, survival with a favorable neurologic outcome, and adverse events. Criteria for included studies were retrospective observational, prospective observational, or randomized controlled trials that included beta-blocker therapy compared to a control group. Two reviewers independently searched for articles that matched these criteria with the appropriate primary and secondary outcomes. Thirty full-text articles were assessed, and three studies were included in the review; one study was a prospective observational study and two studies were retrospective observational studies. Temporary ROSC, defined as ROSC lasting between 30 seconds and 20 minutes, occurred in 19 of 22 patients (86.4%) in the beta-blocker group, as opposed to 14 of 44 patients (31.8%) in the control group. Sustained ROSC was seen in 13 of 22 patients (59.1%) in the beta-blocker group, as compared to 10 of 44 patients (22.7%) in the control group. Survival-to-admission, survival-to-discharge, and favorable neurologic outcome were also found to be significantly higher in the beta-blocker group.

**Comment**: The strength of the article is that it is the first review and meta-analysis to date to assess this topic using human studies. Previously, only animal studies and case reports were done. Two previous systematic reviews regarding this topic have been completed but lacked meta-analysis. This study also evaluated both survival and neurologic function. Several limitations are present, such as the lack of randomized controlled trials. One study included a left stellate ganglion block, which may have introduced heterogeneity. There was also variation in cardiac arrest care (e.g. not all patients received targeted temperature management). This review is important in advancing the field of cardiac arrest care, as patients that are refractory to ACLS algorithm have a survival rate of 3-15%, and of those that survive, only 5% have good neurologic outcome.

*Daniel Buchalter, Charles W Hwang*

RE

IN

**Bartlett E, et al. Systematic review and meta-analysis of intravascular temperature management vs. surface cooling in comatose patients resuscitated from cardiac arrest. Resuscitation. 2020;146:82-95.**

*Favorable neurologic outcome was significantly greater in post-cardiac arrest patients with the use of intravascular cooling methods, as compared to surface cooling methods, for induced hypothermia.*

**Summary:** The objective of this article was to determine if there is a difference in post-cardiac arrest patient survival and neurologic outcome when using different cooling methods such as intravascular and surface cooling. A Boolean search was completed to find observational or interventional studies of cardiac arrest patients aged 18 years or older with induced hypothermia via intravascular cooling vs. surface cooling methods. Articles were reviewed independently for eligibility, methodologic quality, and risk of bias. Vital signs or survival to follow-up were used as a measure for survival, and Cerebral Performance Category of 1 or 2 or a modified Rankin score ≤ 3 were considered markers of favorable neurologic outcome. Analysis was performed on results, statistical heterogeneity, and publication bias. Twelve studies were included with a total of 5581 patients. Results showed that intravascular cooling was associated with a greater chance of survival and good neurologic outcome, as compared to surface cooling. No significant heterogeneity was found among studies and no publication bias was seen in funnel plots. There was also lower risk of arrhythmia and decreased risk of overcooling in the intravascular cooling group.

**Comment**: The article provides clarity to cardiac arrest providers, as prior studies on induced hypothermia compared to normothermia in cardiac arrest patients have shown mixed results. The results may clinically affect the practice of cardiac arrest resuscitation by enhancing favorable neurologic outcome among cardiac arrest patients by limiting reperfusion injury. Previous studies, including multiple retrospective studies, suggest that a temperature of 34ºC or less is associated with a better outcome compared to 36ºC. Small randomized trials have compared intravascular vs. surface cooling, but they lacked enough power to detect differences in outcomes. Some of the strengths of this article are that the studies included are considered moderate in quality and that no statistical heterogeneity or publication bias was found. Other strengths include sample size, use of a random-effects statistical model, and studies were separate in terms of geography, time, and induced hypothermia methods. Limitations are inclusion of only English written texts, strict inclusion criteria, data from mostly observational studies, and statistical analysis showing significant differences among neurologic outcome but no statistical difference in survival rates between the treatment groups.

*Daniel Buchalter, Charles W Hwang*

RE

IN

**Calabró L, et al. Effect of different methods of cooling for targeted temperature management on outcome after cardiac arrest: a systematic review and meta-analysis. Crit Care. 2019;23:285.**

*This meta-analysis of targeted temperature management (TTM) techniques demonstrated lower probability of unfavorable neurological outcome for core, invasive, and temperature feedback devices, when compared to their respective counterparts.*

**Summary:** The objective of this meta-analysis was to compare the effects of core versus surface cooling, invasive versus non-invasive cooling, and the use of temperature feedback devices on neurological outcome and mortality. Study screening and selection was performed by two authors with disagreements settled by a third. Only retrospective, prospective, or randomized controlled trials comparing at least two cooling methods were included. Primary and secondary outcomes were the occurrence of unfavorable neurological outcome and overall mortality, respectively. Core cooling devices, invasive cooling devices, and temperature feedback devices all demonstrated a lower probability of unfavorable neurological outcome.

**Comment**: This meta-analysis boasts excellent design. However, the results are limited by the quality of evidence available. Of the twenty-two included studies, only four were randomized controlled trials, and four were prospective studies. Furthermore, the isolated randomized controlled trials did not show quite as significant outcomes. Overall, this study expands on prior research demonstrating the effectiveness of targeted temperature management. The authors take a significant step for cardiac arrest research as the first meta-analysis to evaluate different cooling methods.

*Garrett M Snipes, Charles W Hwang*

RE

IN

**Barbarawi M, et al. Optimal timing of coronary intervention in patients resuscitated from cardiac arrest without ST-segment elevation myocardial infarction (NSTEMI): A systematic review and meta-analysis. Resuscitation. 2019;144:137-44**

*Coronary angiography, whether immediate or delayed, was found to increase both short- and long-term survival, as well as provide increased favorable neurological outcomes, among non-ST-elevation myocardial infarction (NSTEMI) cardiac arrest patients.*

**Summary:** Cardiac arrests due to NSTEMIs are extremely common, comprising up to 33% of arrest cases. While coronary angiography remains the standard of care, data involving the impact of this intervention is not well characterized. Thus, this systematic review and meta-analysis evaluates the current literature to analyze the impact of coronary angiography on patient outcomes. This was achieved by utilizing a systematic search of PubMed, Cochrane, and Embase databases from their establishment until March 28, 2019. Outcomes of interest included mortality ≤ 30 days, long-term mortality, and neurological outcome measured by Cerebral Performance Category (CPC). Eleven studies (3702 patients) were evaluated, including a trial from earlier the same year by Lemkes et al.. Coronary angiography was found to result in significantly lower short-term mortality compared to the control group (OR 0.17; 95% CI 0.04–0.64) vs (OR 0.07; 95% CI 0.01–0.29), as well as less long-term mortality (OR 0.21; 95% CI 0.05–0.82) vs (OR 0.11; 95% CI 0.03–0.43) when compared to the control group. Additionally, the number of favorable neurological outcomes, measured via a CPC score of 1-2, was increased in patients treated with coronary angiography. There was no significant impact between immediate versus delayed intervention on any of these outcomes of interest.

**Comment**: The results of this analysis support the current standard of care throughout the medical community, emphasizing the important impact that coronary angiography has on patient outcomes. This is true whether treatment is immediate or delayed. It is important to note that confounding variables may contribute to the lack of statistical significance in the impact of timing of coronary angiography upon patient outcomes. Limitations of the study include biases as a result of including several retrospective observational studies compared to the fewer and less-powered randomized controlled trials. Furthermore, heterogeneity was present that may be attributed to varying definitions of “immediate” vs “delayed” intervention. With future randomized controlled trials, the ramifications of treatment would be better understood. Nonetheless, this study highlights the many positive aspects of coronary angiography in NSTEMI cardiac arrest patients.

*Dru Curtis, Charles W Hwang*

RE

IN

**Kyriazopoulou E, et al. Sinus Bradycardia During Targeted Temperature Management: A Systematic Review and Meta-Analysis. Ther Hypothermia Temp Manag. 2020;1:17-26.**

*Sinus bradycardia was associated with significant decreases in patient mortality and favorably affects neurological function in post-cardiac arrest patients.*

**Summary:** Post-cardiac arrest care has been the subject of the medical community for some time, as the prognosis remains poor with limited therapeutic options. One therapeutic treatment that has been shown to be effective is targeted temperature management (TTM), although scientists have suggested that there is still a great amount of variability in patient heart rate during the first 24-48 hours post-arrest. Thus, this systematic review and meta-analysis studied the association of sinus bradycardia during TTM with patient outcomes. Searching through PubMed, Cochrane, ClinicalTrials.gov, and Google Scholar databases from their establishment until January 2019, four studies were included, each being retrospective studies. The outcomes of interest were the mortality rate and neurological function as measured by Cerebral Performance Category (CPC), with favorable neurological outcomes defined as a CPC score of 1-2. Bradycardia, defined as heart rate below 50 beats per minute, during TTM was discovered to significantly decrease mortality compared to the control group (OR = 0.42; 95% CI: 0.29–0.59). Additionally, in two studies of the meta-analysis, bradycardia was associated with an insignificant increase in favorable neurological outcomes, although other studies in the past have reached a statistically significant increase in this outcome measure.

**Comment**: The results of this systematic review and meta-analysis adds another viable option for treating critical post-cardiac arrest patients via bradycardia during TTM. With TTM providing neuroprotection, bradycardia may additionally prevent arrythmias and QTc interval prolongation. These findings were supported with low heterogeneity due to independent researchers performing data extraction. Limitations of this study include varying definitions of bradycardia between studies, as well as the retrospective nature of each study which could introduce biases. However, this analysis is consistent with prior research in the field and discusses the possibility that a patient heart rate below 50 beats per minute during TTM may be cardioprotective. Finally, it calls for future projects aimed at studying the pathophysiology behind this mechanism.

*Dru Curtis, Charles W Hwang*

RE

IN

**Chen Z, et al. Clinical Efficacy of Extracorporeal Cardiopulmonary Resuscitation for Adults With Cardiac Arrest: Meta-Analysis With Trial Sequential Analysis. Biomed Res Int. 2019;2019:6414673.**

*A meta-analysis comparing efficacy of extracorporeal cardiopulmonary resuscitation*

*(ECPR) with traditional CPR found that cardiac arrest patients who received ECPR had improved survival and neurological outcome.*

**Summary**: The objective of this paper was to use meta-analysis and trial-sequential analysis (TSA) to compare survival and neurologic outcomes in patients who receive ECPR versus traditional CPR. In-hospital cardiac arrest (IHCA) and out-of-hospital cardiac arrest (OHCA) patients were included. Primary outcome was 30-day survival. Secondary outcomes were 30-day neurologic outcomes, 3-6 months’ survival, 3-6 months’ neurologic outcome, 1-year survival, and 1-year neurologic outcome. Neurologic status was considered favorable based on Cerebral Performance Category (CPC) of 1 or 2, or when Modified Glasgow Outcome Score (MGOS) was 4 or less. They identified 13 eligible observational studies for their final analysis. Studies were included based on pre-determined quality assessment scales. Quality of evidence for each outcome was assessed using the Grading of Recommendations Assessment, Development, and Evaluation (GRADE). TSA was used to evaluate whether there was sufficient evidence for the anticipated intervention effect and that no further trials were needed. This meta-analysis found that IHCA patients who received ECPR had improved 30-day survival and 30-day neurologic outcome compared to traditional CPR (RR 1.90) and TSA confirmed the evidence as sufficient and conclusive that further trials are not required. In addition, for patients with IHCA this analysis showed ECPR provided benefit at 3-6 months and 1 year after cardiac arrest.

**Comment:**. The authors used rigorous scales and scoring systems to assess the papers included in this meta-analysis. They also used appropriate and informative analysis tools to analyze their data. One limitation is that all of the trials included were observational cohort studies as no randomized controlled trials were available. This indicates that unbalanced confounders may exist. Some of the trials that were included had differences in baseline characteristics between those who received ECPR vs. traditional CPR, and indications for ECPR varied greatly among the 13 studies. Although ECPR was not found to provide survival benefit in the OHCA group, it is important to recognize the TSA analysis indicated that the evidence to reach this conclusion was insufficient. Similarly, although it showed ECPR was associated with better neurologic outcome in OHCA patients, it is important to realize the TSA analysis indicated the evidence to reach this conclusion was also insufficient. The authors thus state future large and adequately powered prospective clinical trials would be needed to confirm whether OHCA patients benefit from ECPR or not. The findings with respect to IHCA patients, however, are promising.

*Kathryn Dasburg, Charles W Hwang*

RE

IN

**Couper K, et al. Prophylactic antibiotic use following cardiac arrest: A systematic review and meta-analysis. *Resuscitation*. 2019;141:166-73.**

*Many patients who are successfully resuscitated following cardiac arrest often suffer from infective complications in the intensive care unit. The use of prophylactic or early antibiotics has therapeutic potential for reducing infective complications in post cardiac arrest patients.*

**Summary:** Pneumonia is a common infectious complication that arises in patients resuscitated from cardiac arrest. These infections can significantly lengthen ICU hospitalization and increase mortality. This is a systemic review and meta-analysis evaluating the effect of prophylactic antibiotic use in adult patients who were successfully resuscitated following cardiac arrest. Observational and interventional research studies were selected through Medline (1946-current), EMBASE (1947-current), and the Cochran Library. The 11 eligible studies consisted of three randomized controlled trials and eight observational studies. The overall treatment effect showed no improvement in survival, neurological outcome, critical care length of stay, incidence of pneumonia diagnosis, or duration of invasive mechanical ventilation. Two studies did report reduced incidence of positive respiratory microbiology in the first three days of treatment, but this effect did not persist past the fourth day.

**Comment**: Although pneumonia can have detrimental repercussions on key clinical outcomes in adult patients who are successfully resuscitated following cardiac arrest, this review found no benefit in the use of prophylactic or early antibiotics. This review included a relatively small number of studies which had a total of 6149 patients. Methodology and data collection varied among the studies which caused many trials to be excluded when evaluating individual clinical outcomes. The population reviewed was diverse with studies from countries across Europe, Asia, and North America being included. The choice of antibiotic given was decided by local factors and the study recognizes that patients chosen for prophylactic antibiotic treatment may be infected by a bacterium resistant to treatment. The study stressed that the evidence quality for all outcomes was low and further high-quality evidence in this area is needed.

*Robert R Leverence, Charles W Hwang*

OR

PED

**Kim CW, *et al.* Effect of metronome guidance on infant cardiopulmonary resuscitation. European Journal of Pediatrics. 2019; 178:795-801.**

*In an infant CPR model, use of a metronome significantly increased the percentage of adequate chest compression rates, in both two-finger and two-thumb techniques, while maintaining adequate chest compression depth.*

**Summary:** Various feedback devices have been investigated to promote high quality CPR components, including adequate chest compression rate and depth, full chest recoil, minimal interruptions, and avoidance of excessive ventilation during simulations. In the adult literature, use of a metronome to effectively set a rate has been validated, however, its use was associated with decreased chest compression depth. This study, comparing the addition of metronome guidance of 110 bpm on infant CPR simulation, was a prospective, randomized, simulation trial in which 36 medical doctors, members of their hospital’s CPR team, volunteered to perform CPR on a 3 month old manikin. They were randomly assigned to either a two-finger (n=20), or two-thumb (N=16), technique. After a baseline assessment without metronome guidance, each group performed CPR a second time either with or without metronome guidance. Variables reviewed included chest compression depth and rate, ratio of adequate chest compression depth and rate, ratio of complete recoil, ventilation, and hands-off time. The study demonstrated that metronome guidance increased the percent time that an adequate rate was provided without negatively impacting other components of high-quality CPR.

**Comment**: Previous studies have shown that CPR performance can be highly variable and of suboptimal quality. In this study, metronome tempo increased the percent adequate chest compression rate without diminishing chest compression depth, as was found in adult studies. While this simulation study supports the use of a simple tool to assist in the consistent performance of high quality CPR, this tool may not be feasibly extrapolated to a broader range of clinical providers and environments until additional studies prove that metronome efficacy persists for real-life scenarios.

*Maria Echavarria, K. Leslie Avery*

RE

PED

**2019 International Consensus on Cardiopulmonary Resuscitation and Emergency Cardiovascular Care Science With Treatment Recommendations: Summary from the Basic Life Support; Advanced Life Support; Pediatric Life Support; Neonatal Life Support; Education, Implementation, and Teams; and First Aid Task Forces. Circulation. 2019; 140:e826-e880.**

*The Pediatric Task Force of the ILCOR systemically reviews and grades recent, peer-reviewed, published cardiopulmonary resuscitation science. This 3^rd^ focused update summarizes findings supporting dispatcher-assisted CPR guidance (“DA-CPR”) to improve survival for pediatric patients suffering OHCA.*

**Summary:** It is established that the provision of DA-CPR increases the provision of bystander CPR, which in turn is associated with reduced mortality and morbidity for OHCA in pediatrics compared to no CPR. In this 3^rd^ annual focused review, ILCOR published new Consensus on Science With Treatment Recommendations (“CoSTR”) that emergency medical dispatch centers should offer DA-CPR instructions for presumed pediatric cardiac arrest, and also that emergency dispatchers should provide CPR instructions for pediatric cardiac arrest when no bystander CPR is in progress (Class 1; Level of Evidence C-LD). These new recommendations are based on five large, separate registry studies, two from Japan and three from Korea, including over 11,000 events. Outcome measures were survival and favorable neurologic outcome at discharge or one-month post-arrest. No recommendations are made for DA-CPR when bystander CPR has already commenced, however, as existing low-quality evidence does not clearly suggest improved outcomes in these scenarios.

**Comment:** These new recommendations from the AHA for DA-CPR validate common-sense interventions for improved management of pediatric OHCA, yet point out many knowledge gaps for future research, including what is the essential content for instructions, and how instructions should be tailored based on the patient age, circumstances and etiology of the arrest, bystander CPR skills, and local EMS standards.

*Ellen George, K. Leslie Avery*

RE

PED

**2019 American Heart Association Focused Update on Pediatric Advanced Life Support: An Update to the American Heart Association Guidelines for Cardiopulmonary Resuscitation and Emergency Cardiovascular Care. Circulation. 2019; 140:e904-e914.**

*This review summarizes findings and publishes updated recommendations based on systemic reviews of 1) advanced airway management in pediatric out-of-hospital cardiac arrest (“OHCA”), 2) extracorporeal cardiopulmonary resuscitation (“ECPR”) following pediatric cardiac arrest and 3) targeted temperature management (“TTM”) during post-cardiac arrest care.*

**Summary:** The Pediatric Life Support Task Force of the ILCOR systemically reviews and grades recent, peer-reviewed, published cardiopulmonary resuscitation science. Consensus on Science With Treatment Recommendations (“CoSTR”) are developed after analyzing the data from RCTs and observational studies with a GRADE approach to provide recommendations for specific questions.

The 2019 update reaffirms 2010 recommendations regarding the use of effective bag mask ventilation (“BMV”), compared to endotracheal intubation (“EI”) or supraglottic airway (“SGA”) placement in the management of pediatric patients with OHCA, based on a lack of evidence supporting improved survival or neurologic outcomes using advanced airways. Due to lack of evidence, no recommendations could be made for airway management for in-hospital cardiac arrest (“IHCA”). (Class 2a; Level of Evidence C-LD).

Three studies of ECPR were reviewed, reaffirming the 2015 guidelines supporting the use of ECPR for pediatric patients with a cardiac diagnosis who suffer IHCA in a setting with ECMO support and expertise. Due to lack of evidence, no recommendation is made for or against ECPR for IHCA of pediatric patients without a cardiac diagnosis or OHCA. (Class 2b; Level of Evidence C-LD).

Finally, the 2019 recommendations expand the 2015 PALS TTM guidelines for infants and children who remain comatose to include those post- IHCA, as well as OHCA. Continuous measurement of core temperature is advised for a TTM of 32$^{\circ}$C to 34$^{\circ}$C followed by a TTM of 36$^{\circ}$C to 37.5$^{\circ}$C or alternatively, a TTM of 36$^{\circ}$C to 37.5$^{\circ}$C. (Class 2a; Level of Evidence B-NR).

**Comments:** The updated recommendations in this year’s ILCOR/AHA comprehensive update affirm and refine existing pediatric resuscitation practices. Most of the recommendations, however, are supported by low quality evidence, demonstrating the need for further research into pediatric cardiac arrest research.

*Manjiri Tule, K. Leslie Avery*

RE

PRE

**Lalande E, et al. Is point-of-care ultrasound a reliable predictor of outcome during atraumatic, non-shockable cardiac arrest? A systemic review and meta-analysis from the SHoC investigators. Resuscitation. 2019; 139: 159-66.**

*PoCUS has become a useful tool in predicting ROSC, survival to admission, and survival to discharge from the hospital in adult patients who are suffering from out-of-hospital non-traumatic cardiac arrest with arrhythmias consistent with pulseless PEA and asystole.*

**Summary:** The role of Point-of-Care ultrasound (PoCUS) has steadily increased within emergency medicine and has been used frequently in cardiac arrest. Most of the literature on PoCUS involves traumatic arrests, therefore this study aimed to research PoCUS and its use in non-traumatic out-of-hospital arrests and their survival to ROSC, admission and discharge. The meta-analysis included 10 studies which composed 1486 patients. All traumatic arrests, VF, and pulseless VT were excluded from the study. It was found that patients with cardiac activity on PoCUS showed a 16.6% (195/1174) favorable outcome for ROSC, 10.8% (156/1437) for survival to admission, and 1.3% (15/1127) for survival to discharge in comparison with those that did not show cardiac activity on PoCUS which were as followed: 11.5% (135/1174) for ROSC, 4.2% (60/1437) for survival to admission, and 0.03% (4/1127) for survival to discharge. There were certain discrepancies between the different studies sensitivity and specificities of PoCUS for cardiac arrest but the overall sensitivity of cardiac activity on PoCUS for ROSC was found to be 60.3% and the specificity 91.5%.

**Comment:** This study was one of the first meta-analysis to show the prognostic value of PoCUS in regard to non-traumatic, out-of-hospital cardiac arrest and their ability to survive to ROSC, hospital admission, and discharge. However, this study was limited by only including studies in the French and English language which could lead to a selection bias. The study is also at risk of inherent bias as the 10 studies selected were only prospective and retrospective cohort studies which did not include randomization or blinding. In addition, 9 of the studies included confidence intervals for survival to hospital admission; however, only 6 studies included it for ROSC and 5 studies for survival to discharge—which could have caused the wide confidence intervals that were reported for ROSC and survival to discharge. There were many different study protocols that were throughout the 10 studies that could have led to significant differences in results reported. These differences included: different numbers of views required for inclusion, different numbers of PoCUS evaluations performed during a particular cardiac arrest, and different levels of experience that the clinicians preforming the exam had. Finally, there was not a unified definition for “cardiac arrest” which could have caused bias when selecting certain studies.

*Chanteil Ulatowski, David Crabb, Karl Huesgen*

RE

PRE

**Nas J, et al. Diagnostic performance of the basic and advanced life support termination of resuscitation rules: A systemic review and diagnostic meta-analysis. Resuscitation. 2020; 148:3-13.**

*A meta-analysis that looks at different termination of resuscitation (TOR-rules) for BLS and ALS in different regions with focus on key arrest characteristics for the particular TOR-rules.*

**Summary:** Termination-of-resuscitation rules (TOR-rules) are created in order to reduce futile out-of-hospital cardiac arrest transports to a hospital. These rules can differ between BLS, ALS and region. Typical BLS TOR-rules include 3 criteria: any ROSC (prior to transport), a shock was delivered, arrest witnessed by EMS personnel and typical ALS TOR-rules include 4 criteria: Any ROSC (prior to transport), a shock was delivered, arrest witnessed, bystander CPR performed. If any of these criteria are met, they will transport to a local emergency department. If none are met, EMS personnel can consider termination of resuscitation. A meta-analysis was performed that included 19 studies with the aim to measure the use of BLS vs ALS TOR-rules with a primary outcome of PPV and specificity of death and secondary outcome of PPV and specificity for unfavorable neurologic outcomes. For the diagnostic accuracy for death, the BLS TOR-rule had a PPV of 0.99, NPV of 0.14, specificity of 0.95, and sensitivity of 0.66 with an overall mean transport rate of 43% with a range of 25-79% within the individual studies. As for the ALS TOR-rule, the diagnostic accuracy of death had a PPV of 1.00, NPV of 0.09, specificity of 0.98, and sensitivity of 0.26 with an overall mean transport rate of 76% with a range from 66-94% within the individual studies. For the diagnostic accuracy of unfavorable neurologic outcomes, the BLS TOR-rule had a PPV of 1.00, NPV of 0.11, specificity of 0.96, and sensitivity of 0.65 and the ALS TOR-rule had a PPV of 1.00, NPV of 0.05, specificity of 0.98, and sensitivity of 0.27. The studies were further broken down within different regions which included Western vs non-Western regions and their major differences. Overall, there was a relatively high PPV; however, there were a lot of variations between BLS and ALS TOR-rules within the different regions. This led to a lower specificity and PPV in non-Western regions and certain populations where in field-defibrillation occurred at lower rates.

**Comment:** This study demonstrates key differences between BLS/ALS TOR-rules and Western vs non-Western regions that effected the PPV. It helped prove that local validation of TOR-rules is a necessity before their clinical use. The main limitation of the study was that all the studies were of observational design. The study did its best to perform several sub-analyses; however, there were a lot of different confounders that could have caused discrepancies in the data like quality of CPR and the quality of care received after resuscitation. In addition, not all studies reported the survival to discharge, which may have affected the results.

*Chanteil Ulatowski, David Crabb, Karl Huesgen*

OR

PRE

**Harari Y et al. Paramedic equipment bags: How their position during out-of-hospital cardiopulmonary resuscitation (CPR) affect paramedic ergonomics and performance. Appl Ergon. 2020; 82:102977.**

*Effective equipment handling and positioning have the potential for both reducing work-related injuries and improving performance efficiency.*

**Summary:** Paramedics are among the highest risk of work-related injuries. They are three times more likely to suffer occupational related injuries than all other occupations. This liability attributes to nearly 350 lost working days per 10,000 workers. 94% of these injuries are musculoskeletal: sprains, strains and back pain. The leading cause of injuries is poor body mechanics when lifting, carrying, or handing patients and/or equipment. In order to investigate whether the positions of equipment bags affect the quality of CPR and work efficiency the following dependent variables were selected for measurement during simulation events: quality of CPR, work efficiency, physiological effort and biomechanical load. The quality of CPR was measured by the depth and rate of compression and the overall fraction of compression performed during the simulation. Work efficiency looked at the time spent on tasks not related to CPR. The physiological effort was measured both subjectively and objectively looking at the heart rate. The Borg test evaluated the paramedic-perceived level of effort. Biomechanical loads had both a direct measure (number of times the bags were handled) and an ergonomic assessment using the REBA. It calculated peak compression forces acting on the paramedics while lifting equipment bags. Statistical analysis was carried out using the MANOVA test for both dependent and independent variables. A test was also performed in regard to the physiological and work efficiency to ensure there was no significant difference held between the senior and junior paramedics.

**Comment:** Both the quality of CPR and the ergonomic effect on the paramedics were positively influenced by placement of the equipment around the patient. Further studies regarding optimal equipment positioning with the objective of creating guidelines for optimal positions with respect to CPR quality, work efficacy, physiological effort and biomechanical loads should be explored. Paramedics hold an exorbitant burden when it comes to work-related injuries, therefore it would be beneficial to both paramedics and employers as well to further these studies.

*Danielle Roberts, David Crabb, Karl Huesgen*

OR

PRE

**Miragli D, et al. The evolving novel treatment techniques in the management of patients with refractory VF/pVT out-of-hospital cardiac arrest. Am J Emerg Med. 2020; 38(3):648-654.**

*Although the early results are promising, the current evidence is not strong for large-scale adoption of ECMO, esmolol, double sequential defibrillation (DSD), and stellate ganglion block (SGB) as treatments for refractory ventricular fibrillation/pulseless ventricular tachycardia (VF/pVT).*

**Summary:** Ventricular fibrillation and pulseless ventricular tachycardia (VF/pVT) are common, deadly cardiac arrythmias. There are currently no consensus guidelines established regarding management of refractory VF/pVT. This was a literature review of observational studies of different novel treatments for refractory VF/pVT: extracorporeal membrane oxygenation (ECMO), esmolol, double sequential defibrillation (DSD), and stellate ganglion block (GSB). The studies mainly focused on prevalence of refractory VF/pVT as initial rhythm, ROSC, survival to discharge and favorable neurologic outcome (Pittsburgh Cerebral Performance Category of 1 or 2 at time if hospital discharge). 5,331 articles were reviewed by two independent reviewers and a total of 23 were found to meet the inclusion criteria and were included in the study. There were promising findings in survival to discharge and favorable neurologic outcome in 16 studies for ECMO. Esmolol and double sequential defibrillation did not have any consistent positive findings for survival to discharge or favorable neurologic outcome. There were no identified studies on GSB for refractory VF/pVT.

**Comment:** While there were some early promising results, especially for ECMO, there is no consistent, strong evidence for the wide-spread adoption of these management techniques. The evidence for the interventions is only a limited of case reports, case series and observational studies. Large-scale, randomized clinical studies are necessary for better assessment of their potential.

*Danielle Roberts, David Crabb, Karl Huesgen*

RE

PRE

**Mao L, et al. Mechanical Chest Compression with LUCAS Device Does not Improve Clinical Outcome in Out-of-Hospital Cardiac Arrest Patients: A Systematic Review and Meta-Analysis. Medicine (Baltimore). 2019; 98: 44-50.**

*Mechanical compression devices such as the LUCAS may provide consistent chest compressions, however, controversy exists over the quality of compressions automatic mechanical devices can deliver.*

**Summary:** This article aims to evaluate how effective using the LUCAS device is in improving clinical outcomes in out-of-hospital cardiac arrests. This systematic review included randomized control trials, cohort, and case control studies where the LUCAS device and manual chest compression were subject groups. All animal studies, case reports, abstracts, reviews, drug trials, and studies conducted in languages other than English or Chinese were excluded. Selected articles were reviewed independently, and statistical analyses were conducted using the Q test and I2 test. The authors found that there was no significant difference between manual CPR and the LUCAS device in the areas of ROSC, survival to hospital admission, survival to discharge, or survival to 30 days.

**Comment**: This study benefited from a strong design and a large number of subjects – 8501 in the survival to hospital admission group. Additionally, the selected studies in this review maintained a balanced ratio of LUCAS cases to manual compression cases in their investigations. However, the validity of this review is limited by a small pool of original studies (6), low number of RCTs (4), and lack of blinding. None of the studies included data that could speak to the effectiveness of compressions such as oxygen saturation or end-tidal CO2. The delivery and effectiveness of high-quality chest compressions is a vital component of prehospital cardiac arrest care. There has been much debate over the effectiveness of automated CPR devices, such as the LUCAS. This study, although a small piece of the investigation, builds on more recent research that did not show a significant difference in the quality of chest compressions between subject groups, but rather supports automated devices as an addition to OHCA care in order to combat provider fatigue and reduce CPR interruption.

*Kayvon Yazdanbakhsh, David Crabb, Karl Huesgen*

OR

PRE

**Teo MHN, et al. The use of dispatcher assistance in improving the quality of cardiopulmonary resuscitation: A randomised controlled trial. Resuscitation. 2019; 138:153-59**

*Dispatcher assisted CPR has potential to improve CPR quality in medical professionals and lay persons.*

**Summary:** Rapid initiation and use of proper technique during cardiopulmonary resuscitation (CPR) is crucial to improve outcomes in cardiac arrest (CA) patients. Dispatcher-assisted CPR (DA) has been used to improve bystander CPR with varying success in different rescuer populations. This randomized control study investigated the use of CPR with DA versus CPR without DA in Southeast Asian medical professionals and lay persons. 408 subjects were randomized to either dispatcher assisted CPR (DA+) or no dispatcher assistance (DA-) groups in a 1:1 ratio. Subjects in each group were asked to perform CPR on a manikin while objective data was collected by the manikin and subjective data collected by three independent assessors. Subjects in the DA+ group were given CPR instructions via a phone speaker while the DA- group was given no instruction. Primary outcomes were measured objectively by assessing compression rate, compression depth, no-flow time, complete release between compressions, and hand placement; subjective data collected included correct kneeling position, rescuer compressing vertically over the patient, palm contact with chest, fingers interlaced, and locked elbows. Participants in the DA+ group compressed at a more accurate compression rate when compared to the control group with no improvements in other measures. When each group was stratified regarding occupation, healthcare professionals that were in the DA+ group significantly improved hand position. Additionally, when non-healthcare professionals in each group were stratified to whether they had a valid CPR certification or not, only the non-valid certification sub-group improved in no-flow time, compression rate, and complete release of compression. Subjective results indicated that only hand-over-hand placement and palm in contact improved in the DA+ group. These outcomes show that DA+ CPR may be beneficial to improving some quality of CPR in both medical professionals and lay persons.

**Comment:** Dispatcher assisted CPR is one method used to improve bystander CPR quality. This study demonstrated that compression rate improved when DA CPR was present in both healthcare professionals and lay persons. Limitations to this study include a possible sampling bias as all participants were recruited from a hospital’s common area. External validity may be limited because compressions were only conducted for two minutes whereas in single rescuer CPR, compressions for longer than two minutes may be necessary to allow for longer ambulance arrival time. However, because quality bystander CPR has been proven to improve OHCA outcomes, this study warrants future investigations into the efficacy of DA CPR with different lengths of time of compressions, varying scripts given by dispatcher, and other variables that can be optimized to provide quality CPR assistance to bystanders.

*Robert Cueto, David Crabb, Karl Huesgen*

RE

PRE

**Perkins GD, et al. The effects of adrenaline in out-of-hospital cardiac arrest with shockable and non-shockable rhythms: Findings from the PACA and PARAMEDIC-2 randomised controlled trials. Resuscitation. 2019: 140:55-63**

*Administration of adrenaline during OHCA significantly increases ROSC in non-shockable rhythms.*

**Summary:** Although use of adrenaline during out of hospital cardiac arrests (OHCA) has been in practice for a considerable amount of time, only recently has its efficacy been studied in randomized control studies in out-of-hospital settings. This meta-analysis investigated the results of two random control studies that compared the efficacy of adrenaline versus placebo in OHCA. The studies analyzed, PARAMEDIC-2 and PACA trial, enrolled a combined number of 7848 subjects and investigated administration of either 1mg doses of adrenaline or 0.9% saline in shockable and non-shockable rhythms. Adrenaline or placebo was administered immediately in subjects with non-shockable rhythms, and adrenaline or placebo was administered after three failed defibrillation attempts in subjects with shockable rhythms. Primary outcome measures included rate of survival at 30 days; secondary outcome measures included rate of survival at time of discharge, rate of survival at 3 months, rate of return of spontaneous circulation (ROSC) at hospital admission, ROSC at any time, and rate of favorable neurological outcome at 3 months all stratified by initial arrest rhythm type. Results indicated greater effects of adrenaline compared to placebo on ROSC for subjects with initially non-shockable rhythms than those with shockable rhythms. Although other outcomes resulted in similar patterns for favorable neurological outcomes and survival patterns, data did not show significance.

**Comments:** This meta-analysis found that the administration of adrenaline improves ROSC in non-shockable rhythms in OHCA. Some limitations to this study include that data was limited to OHCA populations and does not necessarily translate to in-hospital cardiac arrest patients. Additionally, adrenaline was administered in 1mg doses every 3-5 minutes which may vary to other dosing protocols in different regions/ hospitals. Further, larger studies are warranted to investigate the optimal dose, interval, and other variables.

*Robert Cueto, David Crabb, Karl Huesgen*

OR

PRE

**Grunau B, et al. North American validation of the Bokutoh criteria for withholding professional resuscitation in non-traumatic out-of-hospital cardiac arrest. *Resuscitation*. 2019; 135: 51-56.**

*The Bokutoh criteria allow reliable identification of the approximately 1/5 of patients in out-of-hospital cardiac arrest unlikely to benefit from EMS initiated resuscitation.*

**Summary:** This study attempted to validate the Bokutoh criteria in out-of-hospital cardiac arrest (OHCA) in the North American population. The Bokutoh criteria predicts non-survival of patients at initial evaluation if the arrest is unwitnessed, the initial rhythm is non-shockable, and the patient is ≥73 years old. This study utilized existing data from the Trial of Continuous or Interrupted Chest Compressions during CPR, which had prospectively collected information on 25,885 individuals. The researchers ran a secondary analysis of that data. Of those 25,885 patients with OHCA, 5,442 (21%) met the Bokutoh criteria, of whom 64 (1.2%) survived to discharge and 28 (0.51%) had a good neurologic outcome. Sensitivity of the Bokutoh criteria was 0.227 (95% CI 0.221-0.232), specificity was 0.986 (95% CI 0.979-0.990). This study strongly supports use of the Bokutoh criteria in North America.

**Comment:** The current study supported the use of the Bokutoh criteria for determining futility of resuscitation in a subset of individuals presenting with OHCA. The large sample size enhances the validity of the statistics of the study. This study is a secondary analysis of data from another study, where a prospective blinded study with treatment protocols would provide stronger results than re-use of data. Prognostication bias, where physicians believed a patient would not survive so prematurely stopped resuscitation efforts, could have played a significant role in the study results, causing non-survival of Bokutoh positive patients. Lastly, resuscitation treatment decisions and abilities could vary by hospital or by physician, leading to changes in survivability for patients. A goal of the Bokutoh study and others is to improve resource utilization. The cost and effort required to perform resuscitation is significant, however, societal and ethical beliefs on termination of resuscitation must be considered before the Bokutoh criteria should be broadly used.

*Terri Davis, David Crabb, Karl Huesgen*

RE

PRE

**Ng K and Teoh W. The Effect of Prehospital Epinephrine in Out-of-Hospital Cardiac Arrest: A Systematic Review and Meta-Analysis. Prehospital and Disaster Medicine. 2019 Oct;34(5):532-539.**

*The use of epinephrine by prehospital providers increases return of spontaneous circulation and survival to discharge from the hospital, but at time of discharge no significant neurologic improvement is seen.*

**Summary:** Epinephrine, although recommended in cardiac arrest by multiple guidelines, has no definitive proven efficacy or safety in out-of-hospital cardiac arrest (OHCA). This meta-analysis of two trials involving a total of 8,548 patients compared epinephrine versus placebo (0.9% NaCl) in out-of-hospital cardiac arrest. To find these studies, the authors utilized MEDLINE, EMBASE and CENTRAL as well as ClinicalTrials.gov and World Health Organization International Clinical Trials Registry Platform to identify published and unpublished randomized trials comparing epinephrine to placebo in prehospital cardiac arrest. They screened 859 article abstracts, but only 2 studies met criteria for inclusion in the meta-analysis. Patients receiving epinephrine had increased ROSC with an odds ratio of 4.25 (3.79-4.75, P<.001). Increased ROSC led to increased chance of transport to a hospital with an odds ratio of 2.31 (2.11-2.53, P<.001). The survival to hospital discharge also improved with an odds ratio of 1.43 (1.10-1.87, P=.008). There was no significant difference in neurological function at discharge with an odds ratio of 1.21 (0.90-1.64, P=.21). This study demonstrates that patients who receive epinephrine for cardiac arrest prior to arriving at the hospital have an increased rate of return of spontaneous circulation, have an increased incidence of reaching the hospital, and have a higher incidence of survival to hospital discharge. However, there was no significant difference in neurological function at discharge between patients who received epinephrine and those who received placebo.

**Comment:** This meta-analysis of prehospital epinephrine use has several significant limitations. Although this is a meta-analysis, the strongest type of study, it included only 2 studies, limiting its value as a meta-analysis. In addition, the results may be skewed because one study was fifteen times larger than the other. An additional concern is that there was no resuscitation protocol. Additional medications given during the resuscitation could alter the results since they were given at the provider’s discretion and were not controlled for in either study included in the meta-analysis. In cardiac arrest in the pre-hospital setting, epinephrine should be given to improve ROSC and survival, however no meaningful improvement in neurologic function can be expected.

*Terri Davis, David Krabb, Karl Huesgen*

OR

PRO

# Petek BJ, et al. Reexamination of the UN10 Rule to Discontinue Resuscitation During In-Hospital Cardiac Arrest. JAMA Netw Open. 2019; 2(5): e194941

*The UN10 rule, a clinical decision rule for termination of resuscitation following in-hospital cardiac arrest, may have less predictive utility than suggested in previous studies.*

**Summary:** In-hospital cardiac arrest (IHCA) is heavily associated with unfavorable outcomes, with high rates of mortality and poor neurological status. Various clinical decision rules (CDRs) have been developed to help practitioners determine when resuscitation efforts should be terminated. Among them is the UN10 rule, which relies on three factors: (1) unwitnessed arrest, (2) non-shockable rhythm, and (3) no return of spontaneous circulation (ROSC) within 10 minutes of resuscitative efforts. This retrospective cohort study used an US nationwide cohort from the AHA ‘Get With the Guidelines’ registry comprising 96,509 IHCA from 716 US hospitals between 2000 and 2016 to assess the performance of UN10 rule. Study outcomes included survival to discharge and favorable neurological status on discharge, defined as cerebral performance category (CPC) score of 1 (little to no major neurological disability) or 2 (moderate neurological disability). Exclusion criteria included patients who achieved ROSC within 2 minutes of cardiac arrest and patients who received less than 10 minutes of attempted resuscitation despite not achieving ROSC. Outcomes demonstrated that the UN10 rule identified patients with poor neurological outcome and high mortality with a positive predictive value of 93.7%, but did not have the predictive power to justify termination of resuscitation for patients with IHCA, with about 6% of those meeting the criteria for futility surviving to discharge and nearly5% with a good neurological outcome.

**Comment**: The UN10 rule is unique among CDRs in that it considers only intra-arrest variables, making it a simple and easily implemented technique to predict outcomes in cardiac arrest. Limitations include the potential for different outcomes among facilities not participating in the database used in this study, the lack of data about the quality of cardiopulmonary resuscitation (CPR) (e.g. depth of compressions, interruptions), inability to apply findings to patients with out-of-hospital cardiac arrest (OHCA) or to patients following discharge, and changes in standard of care for IHCA over the timeline of the study and its predecessors. UN10 rule was able to identify patients with a lower likelihood of good outcome; however, it was not infallible and should not be used alone when deciding to terminate resuscitation efforts. Further refinement of clinical decision rules in cardiac arrest are needed in pragmatic trials, more compatible with real-world practices.

*Thomas King, Carolina Maciel*

OR

PRO

**Jentzer JC, et al. Cardiogenic Shock Classification to Predict Mortality in the Cardiac Intensive Care Unit. J Am Coll Cardiol. 2019; 74(17): 2117–28.**

*The Society for Cardiovascular Angiography and Intervention (SCAI) classification scheme may be useful in the risk stratification of patients with cardiogenic shock, as SCAI shock stage is independently associated with all-cause hospital mortality.*

**Summary:** Patients with cardiogenic shock (CS) demonstrate significant heterogeneity in hemodynamic status, ranging from minimal hypotension or hypoperfusion to refractory shock. The Society for Cardiovascular Angiography and Intervention (SCAI) classification scheme for CS stratifies patients into one of five stages of progressive hemodynamic insufficiency, each of which may be further delineated by the presence or absence of cardiac arrest. In this single-center cohort study, SCAI staging was retrospectively applied to 10,004 patients admitted between 2007 and 2015, based on data obtained during admission to the cardiac intensive care unit. In a univariate analysis, SCAI stage was found to be associated with all-cause hospital mortality, with mortality rate increasing in a stepwise fashion from 3.0% in SCAI stage A (no hypotension/tachycardia nor hypoperfusion) to 67.0% in SCAI stage E (hypoperfusion with deterioration and refractory shock; p<0.001). After adjustment for age, sex, Charleston Comorbidity Index, Acute Physiology and Chronic Health Evaluation-IV score, presence of cardiac arrest, and use of critical care therapies, each successive SCAI stage was associated with increased odds of hospital mortality (adjusted odds ratios: 1.53 to 6.80; p<0.001 for all) when compared to SCAI stage A. The prevalence of cardiac arrest increased across successive SCAI stages from 7.3% in stage A to 55.8% in stage E, and presence of cardiac arrest was independently associated with hospital mortality (adjusted odds ratio: 3.99; p<0.001).

**Comment**: Current research on CS outcomes and therapies is marred by the substantial variation in cardiogenic shock states among study participants. This study demonstrates that the recent SCAI classification scheme for CS is effective in stratifying patients by mortality risk, as worsening SCAI stage is associated with increased odds of mortality. Additionally, Jentzer *et al.* found that cardiac arrest independently increased mortality risk with each successive SCAI stage, validating the inclusion of cardiac arrest as a SCAI modifier. Thus, SCAI staging may play a valuable role in triaging and therapy selection based on CS severity, as well as minimizing patient heterogeneity in CS research. The strength of these findings lies in the large and diverse cohort, inclusive of patients with and acute coronary syndrome as well as heart failure. Nevertheless, the study is limited by its retrospective nature and reliance on data available in electronic medical records. Additionally, CS of greater severity may have been underrepresented, as only 7.3% of the cohort was classified as SCAI shock stage D and less than 1% as stage E. The clinical findings that determined staging may also have biased clinical decision-making or selected therapies, the extent of which cannot be determined as data on resuscitation status and limitation of therapeutic efforts were not collected. Thus, the study’s overall generalizability remains limited, necessitating replication in a prospective, multicenter cohort.

*Sonya Zhou, Carolina Maciel*

RE

PRO

**Lopez Soto C, et al. Imaging for Neuroprognostication After Cardiac Arrest: Systematic Review and Meta-Analysis. Neurocrit Care. 2020; 32(1): 206-16**

*Lower gray-white ratio on non-contrasted head CT is highly specific (though carries a suboptimal sensitivity) for predicting poor neurologic outcomes post-cardiac arrest, whereas Diffusion Weighted Imaging on brain MRI has a higher sensitivity (at an expense of higher false positive rates or inaccurate predictions of outcome) for severe hypoxic-ischemic brain injury.*

**Summary:** This systematic review and meta-analysis investigates the predictive accuracy of neuroimaging with computed tomography (CT) and magnetic resonance imaging (MRI) for prediction of neurologic outcome after cardiac arrest. Studies published in the Medline from inception to 8/2018 were screened using keywords of cardiac arrest, anoxia, prognosis, and brain imaging. All title and abstracts were then independently screened by two reviewers for inclusion or exclusion; however, it is unclear if they were blinded from one another. Ultimately 44 studies including 4008 patients were analyzed in the systematic review. Studies identified brain edema on CT primarily sing gray-to-white-matter ratio (GWR) while structural changes to brain tissue were identified on MRI primarily using diffusion weighted imaging (DWI). Neurologic outcome was most commonly stratified with Cerebral Performance Category scale at variable times, most frequently at hospital discharge. Meta-analysis revealed that CT evidence of anoxic brain injury based on GWR had a high positive likelihood ratio (13.8, 95% CI 6.9-27.7) for predicting poor neurologic outcomes with very low false positive rates (0.03, 95% CI 0.01-0.07). Meanwhile, MRI evidence of anoxic brain injury based on DWI had a more modest positive likelihood ratio (9.2, 95% CI 5.2-16.4) for predicting poor neurologic outcomes with low false positive ratio (0.08, 95% CI 0.04-0.15). Sensitivity for predicting poor neurologic outcomes was less robust at 0.18-0.51 with CT and 0.65-0.83 with MRI. Results suggest neuroimaging after cardiac arrest can be a valuable tool for neuroprognostication with greater specificity than sensitivity.

**Comment**: This systematic review and meta-analysis was conducted following the rigorous standards of the PRISMA-DTA and was registered on PROSPERO. “While previous systematic reviews recommended against use of neuroimaging for prognostication due to limited evidence, the breadth of data included here strengthens this study’s findings. Limitations included: restrictive search strategy including only one database and in English, no potential bias from included cohort studies with neuroimaging performed at the discretion of the treating physician. There was also significant heterogeneity among studies in imaging characteristics, particularly in measurement of GWR on CT and in radiological technique, brain region examined, calculation methods, and test thresholds in MRI studies. Overall, results indicate neuroimaging with CT or MRI after cardiac arrest can be a valuable prognostic tool for neurologic outcome of patients experiencing cardiac arrest; however, standardization of times for imaging acquisition and methodology for hypoxic-brain injury burden quantification remain a knowledge gap in the cardiac arrest literature. These findings advance the field of cardiac arrest care by identifying specific questions that merit further large scale neuroprognostic studies.

*Shannon Williams, Carolina Maciel*

RE

PRO

**Fernando SM, et al. Pre-arrest and intra-arrest prognostic factors associated with survival after in-hospital cardiac arrest: systematic review and meta-analysis. BMJ. 2019 Dec 4;367:l6373**

*In patients with in-hospital cardiac arrest, male sex, age greater than 60, active malignancy, and chronic kidney disease are highly predictive of early mortality. Cardiac arrests that are witnessed, in monitored patients, occurred during daytime hours, with a shockable rhythm and those that do not require intubation during arrest are associated with higher early survival.*

**Summary:** In-hospital cardiac arrest (IHCA) has different survivability, epidemiology, and etiology when compared to out-of-hospital cardiac arrest (OHCA), yet evidence from OHCA is often inappropriately extrapolated to IHCA. This systematic review and meta-analysis was registered in PROSPERO. It sought to determine associations between pre-arrest and intra-arrest factors in IHCA with early survival using GRADE approach to level of certainty on analyses. Studies published in English were screened in Medline, Pubmed, Embase, Scopus, Web of Science and Cochrane databases from inception to 2/2019. Included studies comprised 23 adult cohorts of IHCA reporting mortality at 30 days and factors associated with mortality (mainly from North America, and mostly from retrospective cohorts). Two reviewers screened, assessed, and abstracted all studies independently; however, it is unclear if they were blinded from one another. In pre-arrest characteristics, male sex was associated with lower odds of survival (0.84, 0.73 – 0.95), as was age 60 and older (0.50, 0.40 – 0.62), history of malignancy (0.57, 0.45 – 0.71), and history of chronic kidney disease (0.56, 0.40 – 0.78). In intra-arrest factors, witnessed cardiac arrest was associated with increased odds of survival (2.71, 2.17 – 3.38), as was monitoring prior to cardiac arrest (2.23, 1.41 – 3.52), cardiac arrest during daytime (1.41, 1.20 – 1.66), and initial shockable rhythm (5.28, 3.78 – 7.39). Intubation during cardiac arrest was associated with worse outcome (0.54, 0.42 – 0.70). Of note, the heterogeneity was markedly high in most outcomes putting the magnitude of the clinical effect size into question; however, the direction of the effect was reliable. The overall risk of bias was low according to QUIPS tool.

**Comment**: This study provides evidence regarding multiple pre-arrest and intra-arrest factors that predict early survival from IHCA. This information may help clinicians incorporate these factors into comprehensive multimodal prognostic assessments. Patients who are monitored prior to cardiac arrest had improved outcomes, which may guide clinicians on utilizing telemetry in patients at high risk for cardiac arrest. Additionally, patients who were intubated during cardiac arrest had a decreased chance of survival. This may reflect length of resuscitation or differing levels of acuity between patients, but in light of literature suggesting the benefit of delaying tracheal intubation during cardiac arrest, raises a clinical question that should be addressed further. Limitations of the study include lack of neurologic prognosis and the high degree of both clinical and statistical heterogeneity between most outcomes. This study is useful for clinicians in gathering information to be incorporated in multimodal neuroprognostic assessments, decisions on monitoring status, and it challenges the practice of early intra-arrest intubation.

*Casey Carr, Carolina Maciel*

RE

PRO

**Shin H et al. Procalcitonin as a prognostic marker for outcomes in post-cardiac arrest patients: A systemic review and meta-analysis. Resuscitation. 2019 May;138:160-167**

*This systematic review and meta-analysis compared 10 studies with a total of 1065 patients and found that there was a positive correlation with procalcitonin levels drawn within 0-48 hours of hospital admission and in-hospital mortality as well as poor neurologic outcome.*

**Summary:** This is a systematic review and meta-analysis of studies that included the measurement of serum procalcitonin levels. The protocol used MOOSE and PRISMA guidelines, followed QUIPS tool and was registered in PROSPERO. Procalcitonin was chosen as a marker of the severity of the post-cardiac arrest syndrome given its association with sepsis, another inflammatory state associated with multi-organ failure. Studies published in MEDLINE and Embase up to 1/2019 were screened. Ten studies (mainly from Europe) were included in the review and the variables of study size, arrest type, number of patients, in-hospital mortality and poor neurologic outcomes, timing of follow-up and timing of procalcitonin draws were abstracted. Results of procalcitonin values measured within 24 and 48 hours of hospital admission were compared with neurologic outcome results based on Glasgow Outcome Scale and Cerebral Performance Category scales. In pooling the results of the ten studies, the authors found that within 24 hours of hospital admission, an elevated procalcitonin level was more common in non-survivors. Elevated procalcitonin in the first 24 hours was also associated with poor neurologic outcomes at hospital discharge, 30-days and both 1-, 6-month follow-up. Procalcitonin levels measured between 24 and 48 hours of hospital admission were also associated with poor neurologic outcome. Three out of 10 included studies were of low-quality due to selection, attrition and reporting biases. Significant heterogeneity was found regarding characteristics of arrest and patient population, as well as PCT sampling times.

**Comment**: This review pooled the results of ten studies of procalcitonin and found that procalcitonin levels within the first 24 and 48 hours of hospital admission may be useful as a marker of the severity of a cardiac arrest and predict ultimate neurologic outcome. Some of the limitations of this study include the relatively limited styles of practice represented as most were single center studies and almost all limited to within Europe, which limit extrapolation to other settings. The heterogeneity of procalcitonin sampling times and arrest types could also have been confounders as initial rhythms, location whether out-of-hospital or in-hospital arrests were not separated. Prospective studies designed with the intent to determine the predictive value of procalcitonin levels at various timepoints and interactions with other post-arrest interventions such as targeted temperature management would provide more convincing data about the use of this biomarker.

*Travis Murphy, Carolina Maciel*
