# Supplementary material for: Cardiac arrest: An interdisciplinary scoping review of the literature from 2019
Source: Resusc Plus. 2020 Nov 4;4:100037. doi: 10.1016/j.resplu.2020.100037 (PMC8244427; doi:10.1016/j.resplu.2020.100037)
Supplement: Supplementary file 4 [file mmc4.pdf]

Article & Reviewer Information						Design (8 max)					Ethics (4 max)			Importance (5 max)			Impact (5 max)			
Citation Title	Category (EPH, PRE, IN, PRO, PED, BSP, GL)	Type: Original Research (OR) or Review (RE)	PubMed ID	Editor Initials	Reviewer Initials	A	B	C	D	E	A	B	C	A	B	C	A	B	C	Total score (22 max)
A caspase-6-cleaved fra	BSP	OR	31693684	SG	JL	2	1	1	1	1	2	1	1	0	2	1	0	0	1	14
A caspase-6-cleaved fra	BSP	OR	31693684	SG	TM	4	1	1	1	1	2	1	1	2	0	0	0	0	1	15
Adenosine 2A Recepto	BSP	OR	31082918	SG	MC	4	1	1	1	1	2	1	1	2	2	1	0	0	1	18
Adenosine 5'-Monoph	BSP	OR	31460871	SG	SC	1	1	1	1	0	2	1	1	0	2	1	0	0	1	12
Adenoviral betaARKct	BSP	OR	30664574	SG	CA	3	1	1	1	1	2	1	1	2	2	1	0	0	1	17
Akt1-mediated CPR c	BSP	OR	31398213	SG	MC	4	1	1	1	1	2	1	1	2	2	1	0	0	1	18
Alterations in Respirat	BSP	OR	31549565	SG	TM	4	1	1	1	1	2	1	1	2	2	1	2	0	1	20
Alterations in Respirat	BSP	OR	31549565	SG	CA	2	1	1	1	1	2	1	1	2	2	1	2	2	1	20
Amiloride Alleviates N	BSP	OR	31272354	SG	MC	4	1	1	1	0	2	1	1	2	2	1	0	0	1	17
Amiodarone exacerbate	BSP	OR	31864286	SG	MC	3	0	0	1	1	2	1	1	2	2	1	2	2	1	19
Amplitude screening i	BSP	OR	30194021	SG	CA	1	1	1	1	1	2	1	1	2	2	1	0	0	1	15
Annexin A1 Bioactive	BSP	OR	31258464	SG	MC	4	1	1	1	1	2	1	1	2	2	1	2	0	1	20
Anti-Nogo-A antibody	BSP	OR	31746353	SG	MC	4	1	1	1	1	2	1	1	2	2	1	0	2	1	20
Beneficial Effects of Iv	BSP	OR	31274829	SG	CA	4	1	1	1	1	2	1	1	0	2	1	2	0	1	18
Biomechanical propert	BSP	OR	31726251	SG	CA	2	1	1	1	1	2	1	1	2	2	1	0	0	1	16
Calcium/Calmodulin-I	BSP	OR	31520314	SG	JL	4	1	1	1	1	2	1	0	2	2	1	0	0	1	17
Cerebral protection of	BSP	OR	31496652	SG	JL	3	1	1	1	0	2	1	1	2	2	1	0	0	1	16
Cerebral protection of	BSP	OR	31496652	SG	TM	3	1	1	1	0	2	1	1	2	2	1	0	0	1	16
Combined Treatment v	BSP	OR	31591692	SG	CA	3	1	1	1	1	2	1	1	2	2	1	0	2	1	19
Comparative Regimen	BSP	OR	30113398	SG	CA	3	1	1	1	1	2	1	1	0	2	1	0	2	1	17
Comparison of hemod	BSP	OR	31469891	SG	MC	4	0	1	1	1	2	1	1	2	2	1	2	0	1	19
Conjunctival Microcir	BSP	OR	31876330	SG	MC	3	0	0	1	1	2	1	1	2	2	1	2	2	0	18
CTL-derived granzyme	BSP	OR	31920929	SG	JL	4	1	1	1	1	2	1	0	2	2	1	0	0	1	17
CTL-Derived Granzym	BSP	OR	31920929	SG	TM	3	1	1	1	1	2	1	1	2	2	1	0	0	1	17
Cyclosporine A plus is	BSP	OR	31576483	SG	JL	4	1	1	1	0	2	1	1	2	2	1	2	0	1	19
Differential effects of h	BSP	OR	30461327	SG	MC	4	1	1	1	1	2	1	1	2	2	1	2	2	0	21
Differential effects on	BSP	OR	31504369	SG	MC	2	0	0	1	1	2	0	1	0	2	1	2	2	1	15
Dim light at night imp	BSP	OR	30822422	SG	JL	4	1	1	1	1	2	1	1	0	2	1	2	0	1	18
Dose-dependent Effects	BSP	OR	31258066	SG	SC	3	1	1	1	1	2	1	1	0	2	1	0	0	1	15
Duration of cardiac arr	BSP	OR	31183772	SG	SC	3	1	1	1	1	2	1	1	0	2	1	0	0	1	15
ECMO attenuates infla	BSP	OR	31253699	SG	CA	3	1	1	1	1	2	1	1	2	2	1	2	0	1	19
Effect of Electrical vag	BSP	OR	30382532	SG	JL	4	1	1	1	1	2	1	1	0	2	1	0	0	1	16
Effect of mild hyperca	BSP	OR	30612967	SG	CA	3	1	1	1	1	2	1	1	2	2	1	2	0	1	19
Effect of mild hypothe	BSP	OR	31690318	SG	CA	3	1	1	1	1	2	1	1	2	2	1	2	0	1	19
Effect of pralidoxime	BSP	OR	31036784	SG	SC	3	1	1	1	1	2	1	1	0	2	1	0	0	1	15
Electroacupuncture pre	BSP	OR	31476306	SG	MC	4	1	1	1	0	2	1	1	2	2	1	0	0	0	16
Endovascular hypother	BSP	OR	31283936	SG	SC	4	1	1	1	1	2	1	1	0	2	1	0	0	1	16
Esmolol for cardioprot	BSP	OR	31802327	SG	TM	4	1	1	1	1	2	1	1	2	2	1	2	0	1	20
Esmolol for cardioprot	BSP	OR	31802327	SG	CA	3	1	1	1	1	2	1	1	2	2	1	0	0	1	17
Exploration of the opti	BSP	OR	30554120	SG	JL	3	1	1	0	0	2	1	1	2	2	1	0	0	1	15
Extracorporeal resuscit	BSP	OR	31482730	SG	CA	3	1	1	1	1	2	1	1	2	2	1	0	0	1	17
Fingolimod Plays Rol	BSP	OR	31835656	SG	MC	4	1	1	1	1	2	1	1	2	2	1	2	0	1	20
First-in-Human study	BSP	OR	31625480	SG	MC	4	1	1	1	0	2	1	1	0	2	0	2	0	0	15
Functional Reclassifica	BSP	OR	30578647	TM	TM	3	1	1	1	0	0	0	0	0	0	1	0	0	1	8
Genetic inhibition of I	BSP	OR	30488977	SG	TM	4	1	1	1	1	2	1	1	0	2	1	0	0	1	16

Genetic inhibition of I	BSP	OR	30488977	SG	CA	2	1	1	1	1	2	1	1	2	1	1	0	0	1	15
Growth differentiation	BSP	OR	31394153	SG	SC	2	1	1	1	1	2	1	1	0	2	1	0	0	1	14
Heterotopic Ossificatic	BSP	OR	31108046	SG	CA	1	0	0	1	0	0	0	0	0	0	0	0	0	0	2
Hippocampus and basa	BSP	OR	31005590	SG	SC	2	1	1	1	1	2	1	1	2	2	1	0	0	1	16
Hypothermia Inhibits (	BSP	OR	31330290	SG	MC	4	0	1	1	1	2	1	1	2	2	1	0	0	1	17
Hypothermic propertie	BSP	OR	31258715	SG	CA	3	1	1	1	1	2	1	1	2	2	1	2	0	1	19
Imbalance of angiotens	BSP	OR	30816437	SG	CA	3	1	1	1	1	2	1	1	0	2	1	0	0	1	15
Improvement in Outco	BSP	OR	30586713	SG	CA	3	1	1	1	1	2	1	1	2	2	1	2	0	1	19
Increased Survival Tin	BSP	OR	29503242	SG	MC	4	0	0	1	1	2	1	1	2	2	1	2	0	1	18
Intra-Arrest Administr	BSP	OR	31309111	DC	KY	4	1	1	1	1	2	1	1	2	2	1	2	2	1	22
Intracerebroventricular	BSP	OR	31946798	SG	MC	4	0	0	1	0	2	1	0	2	2	1	0	0	1	14
Intravenous transplants	BSP	OR	30482485	SG	JL	4	1	1	1	0	2	1	1	0	2	1	0	0	0	14
Intravenous Transplant	BSP	OR	30482485	SG	TM	3	1	1	1	0	2	1	1	2	2	1	0	0	1	16
Involvement of sphing	BSP	OR	30604190	SG	MC	3	0	0	1	1	2	1	1	2	2	1	2	2	1	19
Low dose Iloprost effe	BSP	OR	31945586	SG	TM	4	1	1	1	1	2	1	1	2	0	1	2	0	1	18
Low-dose iloprost effe	BSP	OR	31945586	SG	JL	4	1	1	1	1	2	1	1	2	2	1	0	0	1	18
Mechanisms of early g	BSP	OR	30908518	SG	MC	2	0	1	1	1	2	1	1	0	2	1	0	0	1	13
Melatonin alleviates as	BSP	OR	31251935	SG	MC	4	0	1	1	0	2	1	1	2	2	1	0	0	0	15
Mesenchymal stem cel	BSP	OR	31201814	SG	SC	3	1	1	1	1	2	1	1	0	2	1	0	0	1	15
Mild hypothermia imp	BSP	OR	31428461	SG	MC	4	1	1	1	0	2	1	1	2	2	1	0	0	1	17
Minocycline fails to ir	BSP	OR	31853446	SG	TM	4	1	1	1	1	2	1	1	2	2	1	2	0	1	20
Minocycline fails to ir	BSP	OR	31853446	SG	CA	3	1	1	1	1	2	1	1	2	2	1	0	0	1	17
miR-26a prevents neur	BSP	OR	30992390	SG	CA	3	1	1	1	1	2	1	1	0	2	1	0	0	1	15
Mitochondrial dysfunc	BSP	OR	30854867	SG	CA	2	1	1	1	1	2	1	1	0	2	1	0	0	1	14
Mitophagy in the Hipp	BSP	OR	31773373	SG	CA	2	1	1	1	1	2	1	1	2	2	1	0	0	1	16
Monoacylglycerol Lip	BSP	OR	30431495	SG	CA	3	1	1	1	1	2	1	1	2	2	1	0	0	1	17
Neuroprotection of Gli	BSP	OR	31946797	SG	MC	4	1	1	1	0	2	1	0	2	2	1	2	2	0	19
Nitrite pharmacokineti	BSP	OR	31526855	SG	JL	4	1	1	1	1	2	1	1	2	2	1	0	0	1	18
Noble gas neuroprotect	BSP	OR	31470983	SG	MC	4	1	1	1	1	2	1	1	2	2	1	2	2	1	22
Novel Role of Carbon	BSP	OR	31030597	SG	MC	4	0	1	1	1	2	1	1	2	2	1	2	0	0	18
Oxygen saturation and	BSP	OR	30792759	SG	MC	2	0	1	0	1	2	1	1	0	2	1	0	0	1	12
Palmitic acid methyl e	BSP	OR	30514597	SG	JL	4	1	1	0	0	2	1	0	2	2	1	0	0	1	15
PD98059 protects the	BSP	OR	31265854	SG	SC	3	1	1	1	1	2	1	1	0	2	1	0	0	1	15
Perioperatively Inhalec	BSP	OR	31061920	SG	CA	3	1	1	1	1	2	1	1	2	2	1	2	0	1	19
Pharmacokinetic effect	BSP	OR	30853153	SG	MC	4	1	1	1	1	2	1	1	2	2	1	2	0	1	20
Pharmacokinetic effect	BSP	OR	30853153	SG	MC	4	1	1	1	1	2	1	1	2	2	1	2	0	0	19
Post-TTM Rebound P	BSP	OR	31871429	SG	CA	2	1	1	1	1	2	1	1	2	2	1	2	0	1	18
Pre-arrest hypothermia	BSP	OR	31530020	SG	JL	4	1	1	1	1	2	1	1	0	2	1	2	2	1	20
Propofol does not affe	BSP	OR	31163372	SG	CA	2	1	1	1	1	2	1	1	2	2	1	2	0	1	18
Protective effect of ext	BSP	OR	31777541	SG	CA	3	1	1	1	1	2	1	1	2	2	1	2	0	1	19
Protective effects of gl	BSP	OR	31155149	SG	MC	4	1	1	1	0	2	1	1	2	2	1	0	0	1	17
Relative Resilience of	BSP	OR	31396839	SG	MC	4	1	1	1	0	2	1	1	2	2	1	0	0	1	17
Repetitive anodal trans	BSP	OR	30611705	SG	MC	4	1	1	1	1	2	1	1	2	2	1	0	0	1	18
Search for Therapeutic	BSP	OR	31780928	SG	MC	2	0	0	1	1	2	1	1	2	2	1	2	2	1	18
Selective aortic arch pe	BSP	OR	31348400	SG	MC	4	1	1	1	1	2	1	1	2	2	1	2	2	1	22
Selective beta-blocker	BSP	OR	30321461	SG	MC	4	1	1	1	1	2	1	0	2	2	1	2	2	1	21
Semaphorin 3F Promc	BSP	OR	30877507	SG	SC	1	1	1	1	0	2	1	1	0	2	1	0	0	1	12
Simultaneous cardiac a	BSP	OR	31634345	SG	MC	3	0	0	1	0	2	1	1	2	2	1	0	0	1	14
Single dose of 17B-est	BSP	OR	30471325	SG	JL	4	1	1	1	1	2	1	0	0	0	1	0	0	0	12
Study of the effects of	BSP	OR	30747772	SG	JL	4	1	1	1	1	2	1	0	2	2	1	2	0	1	19
Study of the Effects of	BSP	OR	30747772	SG	SC	4	1	1	1	1	2	1	1	0	2	1	0	0	1	16
Suppression of Supero	BSP	OR	31725436	SG	CA	3	1	1	1	1	2	1	1	2	2	1	2	0	1	19
The Effects of Pharma	BSP	OR	30986796	SG	MC	4	1	1	1	1	2	1	1	2	2	1	0	0	1	18

The incidence of acute	BSP	OR	31014141	SG	TM	3	1	1	1	1	2	1	1	2	0	1	0	0	1	15
The influence of low-e	BSP	OR	31019122	SG	JL	3	1	1	1	0	2	1	1	2	2	1	0	0	0	15
The influences of morp	BSP	OR	31747898	SG	JL	4	1	1	1	1	2	1	1	0	2	1	0	0	1	16
The potassium channel	BSP	OR	29390872	SG	CA	2	1	1	1	1	2	1	1	0	2	0	0	0	1	13
The roles of oxidative	BSP	OR	30951836	SG	CA	3	1	1	1	1	2	1	0	0	2	1	0	0	1	14
Therapeutic hypotherm	BSP	OR	31331507	SG	CA	3	1	1	1	1	2	1	1	0	2	1	2	0	1	17
Therapeutic hypotherm	BSP	OR	29739265	SG	TM	4	1	1	1	1	2	1	1	2	0	1	2	0	1	18
Therapeutic hypotherm	BSP	OR	29739265	SG	JL	3	1	1	1	1	2	1	1	0	2	1	0	0	1	15
Three-Dimensional Sh	BSP	OR	31737367	SG	TM	3	1	1	1	1	2	1	1	2	0	1	0	0	1	15
Three-Dimensional Sh	BSP	OR	31737367	SG	CA	2	1	1	1	1	2	1	1	0	2	1	0	0	1	14
Tissue damage in the l	BSP	OR	31336381	SG	SC	2	1	1	1	0	2	1	1	0	2	1	0	0	0	12
Toll-like receptor 4 de	BSP	OR	31369614	SG	CA	3	1	1	1	1	2	1	1	0	2	1	0	0	1	15
Towards long-acting a	BSP	OR	30586629	SG	CA	3	1	1	1	1	2	1	1	2	2	1	0	0	1	17
Towards personalized	BSP	OR	31403405	SG	CA	3	1	1	1	1	2	1	0	0	2	1	0	0	1	14
Transient hyperthermia	BSP	OR	31627241	SG	MC	3	0	0	1	1	0	1	1	2	2	1	0	0	1	13
Two-site regional oxyg	BSP	OR	30820870	SG	SC	3	1	1	1	1	2	1	1	0	2	1	0	0	0	14
Ultrastructural Charact	BSP	OR	31631775	SG	JL	3	1	1	1	1	2	1	1	0	0	0	0	0	1	12
Use of resuscitative ba	BSP	OR	31121206	SG	MC	3	1	1	1	1	2	1	1	2	2	1	2	2	1	21
Validation of spectral	BSP	OR	31656658	SG	CA	2	1	1	1	1	2	1	1	2	2	1	0	0	1	16
Xuezhikang improves	BSP	OR	30953818	SG	SC	3	1	1	1	0	2	1	1	2	2	1	0	0	1	16
1A Lethal Blow to the	EPH	OR	31277792	MPB/MABC	EM	1	1	1	1	1	0	1	1	0	2	1	2	0	1	13
A 5-year change of kno	EPH	OR	30730932	MPB, MABC	SB	2	1	1	1	1	2	1	1	0	2	1	2	0	1	16
A common indel poly	EPH	OR	31220685	MPB/MABC	EM	2	1	1	1	1	2	1	1	0	2	1	2	0	0	15
A comparative study o	EPH	OR	31623585	MPB/MABC	YE	2	1	1	1	1	2	1	1	0	2	1	0	0	1	14
A five-year retrospectiv	EPH	OR	29932346	MPB/MABC	JW	2	1	1	1	1	2	0	1	0	2	1	2	0	0	14
A Hybrid Risk Assess	EPH	OR	31491657	MPB/MABC	MPB	1	1	1	1	0	0	0	1	0	2	1	0	0	1	9
A laboratory-derived e	EPH	OR	31424605	MPB/MABC	EM	2	1	1	1	1	2	1	0	0	0	1	0	0	1	11
A local neighborhood	EPH	OR	31580910	MABC	MABC	2	1	1	1	1	2	1	1	0	2	1	2	2	1	18
A mediation analysis	EPH	OR	31034486	MBC/MABC	SB	2	1	1	1	1	2	1	1	0	2	1	2	2	1	18
A multidisciplinary ap	EPH	OR	31166220	MPB/MABC	EM	2	1	1	1	1	2	1	1	0	2	1	2	2	1	18
A Nationwide Investig	EPH	OR	30385384	MPB/MABC	EM	2	1	1	1	1	2	1	1	0	0	1	0	0	1	12
A new approach to pre	EPH	OR	31320952	MPB/MABC	YE	2	1	1	1	1	2	1	1	0	2	1	0	0	1	14
A novel DPP6 variant	EPH	OR	31476289	MPB/MABC	JW	3	1	1	1	1	2	1	1	0	2	1	0	0	1	15
A novel familial trunca	EPH	OR	30118858	MPB/MABC	MPB	1	1	0	1	0	0	0	1	0	2	1	0	0	1	8
A randomised controll	EPH	OR	30988532	MPB/MABC	JW	3	1	1	1	1	0	1	1	0	2	1	2	2	0	16
A randomised controll	EPH	OR	31151481	MABC	MABC	4	1	1	1	1	2	1	1	0	2	1	2	2	1	20
A video-based training	EPH	OR	30209672	MBC/MABC	SB	1	1	1	1	1	2	1	1	0	2	1	0	0	0	12
Accuracy of Automatic	EPH	OR	31706968	MPB/MABC	EM	1	1	1	1	1	2	1	1	0	2	1	0	0	1	13
Actual resuscitation ac	EPH	OR	31201883	MPB/MABC	JW	1	1	1	1	1	2	1	1	0	2	1	2	2	1	17
Adult in-hospital cardi	EPH	OR	31075290	MPB/MABC	EM	1	1	1	1	1	2	1	1	0	2	1	0	0	1	13
Age-associated outcom	EPH	OR	31206587	MPB, MABC	SB	2	1	1	1	1	2	1	1	0	2	1	2	0	1	16
An association betwee	EPH	OR	31634617	MABC	MABC	2	1	1	1	1	2	1	1	0	2	1	2	2	1	18
An audio-visual review	EPH	OR	31734218	MABC	MABC	4	1	1	1	1	2	1	1	0	2	1	2	2	1	20
An intelligent warning	EPH	OR	31416562	MPB/MABC	YE	2	1	1	1	1	0	1	1	2	2	1	0	0	1	14
Analysis of a sustained	EPH	OR	31210452	MPB/MABC	EM	1	1	1	1	1	2	1	1	0	2	1	2	0	1	15
Antipsychotics and the	EPH	OR	31743435	MBC/MABC	SB	2	1	1	1	1	0	0	0	0	2	1	2	2	1	14
App-based Learning as	EPH	OR	30105946	MPB/MABC	EM	1	1	1	0	0	2	1	1	2	0	0	0	0	1	10
Association between a	EPH	OR	31578416	MPB/MABC	YE	2	1	1	1	1	2	1	1	0	2	1	0	0	1	14
Association between c	EPH	OR	30677440	MPB, MABC	SC	2	1	1	1	1	2	1	1	2	2	1	0	0	1	16
Association Between I	EPH	OR	31822122	MPB/MABC	JW	2	1	1	1	1	2	1	1	2	2	1	2	2	1	20
Association of ambien	EPH	OR	31527786	MPB/MABC	MPB	2	1	1	1	1	2	1	1	0	2	1	2	0	1	16
Association of health i	EPH	OR	30639790	MPB/MABC	YE	2	1	1	1	1	2	1	1	0	2	1	0	0	1	14
Association of hemogl	EPH	OR	31296079	MPB/MABC	EM	2	1	1	1	1	2	1	1	0	2	1	0	0	1	14

Associations between (	EPH	OR	31217990	MPB, MABC	SB	2	1	1	1	1	2	1	1	0	2	1	2	0	1	16
Attitudes to Cardiopul	EPH	OR	30917733	MPB/MABC	YE	1	1	1	1	1	2	1	1	2	2	1	2	2	1	19
Automated External D	EPH	OR	30111222	MBC/MABC	SB	4	1	1	1	1	2	1	1	0	2	1	2	2	1	20
Automated external de	EPH	OR	31658139	MABC	MABC	2	1	1	1	1	2	1	1	0	2	1	2	2	1	18
Automated extraction (	EPH	OR	31160009	MPB/MABC	EM	2	1	1	1	1	2	1	1	0	2	1	2	0	0	15
Avalanche victims in (	EPH	OR	31185258	MPB/MABC	EM	2	1	1	1	1	2	1	1	0	2	1	0	0	1	14
Basic life support by c	EPH	OR	30976436	MPB/MABC	EM	2	1	1	1	1	2	1	1	0	0	1	0	0	1	12
Basic life support trair	EPH	OR	31189497	MPB/MABC	YE	4	1	1	1	1	2	1	1	0	2	1	2	0	1	18
Basic life support trair	EPH	OR	31189497	MBC/MABC	SB	4	1	1	1	1	2	1	1	0	2	1	2	2	1	20
Bystander automated e	EPH	OR	30790694	MPB, MABC	SC	2	1	1	1	1	2	1	1	2	2	1	0	0	1	16
Bystander cardiopulmo	EPH	OR	30380021	MBC/MABC	SB	2	1	1	1	1	2	1	1	0	2	1	2	2	1	18
Bystander-initiated Co	EPH	OR	31639461	MPB/MABC	EM	2	1	1	1	1	2	1	1	0	2	1	0	0	1	14
Cardiac arrest as an ag	EPH	OR	29856229	MPB/MABC	YE	2	1	1	1	1	2	0	1	0	2	1	2	0	1	15
Cardiopulmonary resus	EPH	OR	30134008	MPB/MABC	YE	1	1	1	1	1	2	1	1	0	2	1	2	2	1	17
Cardiopulmonary Resu	EPH	OR	31840239	MPB/MABC	JW	1	1	1	1	1	2	0	1	0	2	1	0	0	1	12
Cardiopulmonary resus	EPH	OR	30639787	MPB/MABC	JW	2	1	1	1	1	2	1	1	0	2	1	0	0	0	13
Cardiorespiratory fitne	EPH	OR	31422363	MPB, MABC	SB	2	1	1	1	1	2	1	1	0	2	1	2	0	1	16
Cardiovascular effects	EPH	OR	30772188	MPB/MABC	JW	1	1	0	1	0	0	1	0	0	2	1	2	0	0	9
Cardiovascular Outcon	EPH	OR	31581860	MPB/MABC	MPB	2	1	1	1	1	2	1	1	0	2	1	0	2	1	16
Certified Basic Life Su	EPH	OR	31490866	MPB/MABC	MPB	1	1	1	1	1	2	0	1	0	2	1	0	2	1	14
Characteristics of neig	EPH	OR	29961881	MPB/MABC	MPB	2	1	1	1	1	2	1	1	0	2	1	0	2	1	16
Characteristics of Out	EPH	OR	31803370	MABC	MABC	2	1	1	1	1	2	1	1	0	2	1	2	2	1	18
Characteristics of Subj	EPH	OR	31551291	MPB/MABC	EM	2	1	1	1	1	2	1	0	0	2	1	0	0	1	13
Clinical characteristics	EPH	OR	30190239	MPB/MABC	YE	2	1	1	1	1	2	0	1	0	2	1	2	0	1	15
Clinical risk predictior	EPH	OR	31888507	MBC/MABC	SB	1	1	1	1	1	2	1	1	0	2	1	2	0	1	15
Clinician Perspectives	EPH	OR	30624280	MPB/MABC	YE	1	1	1	1	1	2	1	1	2	2	1	2	0	1	17
Combination of proble	EPH	OR	31151450	MPB/MABC	JW	4	1	1	1	1	2	1	1	0	2	1	2	2	1	20
Community-Wide Dis	EPH	OR	30612478	MPB/MABC	JW	2	1	1	1	1	2	0	1	0	2	1	2	2	1	17
Comparable Survival i	EPH	OR	30896453	MPB/MABC	EM	2	1	1	1	1	2	0	1	0	2	1	0	0	1	13
Comparative evaluatio	EPH	OR	30958836	MABC	MABC	4	1	1	1	1	2	1	1	0	2	1	2	2	1	20
Comparative evaluatio	EPH	OR	30958836	MPB, MABC	SB	4	1	1	1	1	2	1	1	0	2	1	2	0	1	18
Comparison of Long-T	EPH	OR	31103131	MPB/MABC	EM	2	1	1	1	1	2	1	1	2	2	1	0	0	1	16
Contactless cardiac arr	EPH	OR	31304398	MPB/MABC	YE	3	1	1	1	1	2	1	1	0	2	1	0	0	1	15
Cost effectiveness and	EPH	OR	30922936	MPB/MABC	YE	2	1	1	1	1	2	0	1	0	2	1	2	0	1	15
Cost-effectiveness of a	EPH	OR	31345972	MPB/MABC	EM	1	1	1	1	1	2	1	1	0	0	1	0	0	1	11
Cost-utility of extracor	EPH	OR	30716427	MPB/MABC	YE	2	1	1	1	1	2	0	1	2	2	1	0	0	1	15
CPRural	EPH	OR	30638192	MPB, MABC	SB	2	1	1	1	1	2	1	1	0	2	1	2	0	1	16
Culture and personal ir	EPH	OR	31878920	MBC/MABC	SB	1	1	1	1	1	2	1	1	0	2	1	2	0	1	15
Current state of knowl	EPH	OR	31752855	MABC	MABC	1	1	1	1	1	2	1	1	0	2	1	2	2	1	17
Data concerning AED	EPH	OR	31193077	MPB, MABC	SB	1	1	1	1	1	0	0	0	0	2	1	2	0	0	10
Depression and anxiety	EPH	OR	30745287	MPB/MABC	JW	2	1	1	1	1	2	1	1	0	2	1	0	0	1	14
Determining the Motiv	EPH	OR	30536860	MPB/MABC	EM	1	1	1	1	1	2	1	1	0	2	1	0	0	1	13
Development of a Mec	EPH	OR	31249769	MPB, MABC	SB	1	1	1	1	1	0	0	0	0	2	1	2	0	0	10
Development, validatio	EPH	OR	31870420	MPB/MABC	JW	3	1	1	1	1	2	1	1	0	2	1	2	0	1	17
Differences in duration	EPH	OR	30708073	MPB/MABC	EM	2	1	1	1	1	2	1	1	0	2	1	2	0	1	16
Differential outcomes f	EPH	OR	30965094	MPB/MABC	EM	2	1	1	1	1	2	1	1	0	2	1	0	0	1	14
Do Patients Suffering	EPH	OR	31584640	MPB/MABC	JW	2	1	1	1	1	2	0	1	2	2	1	2	2	1	19
Does occurrence during	EPH	OR	31238153	MPB, MABC	SC	2	1	1	1	1	2	1	1	0	2	1	0	0	1	14
Dyadic effects of type	EPH	OR	31752502	MPB/MABC	MPB	2	1	1	1	1	2	1	1	0	2	1	0	0	1	14
Early warning score ad	EPH	OR	30588704	MABC	MABC	2	1	1	1	1	2	1	1	0	2	1	2	2	1	18
ECG derived Cheyne-S	EPH	OR	30340809	MPB/MABC	MPB	2	1	1	1	1	2	1	1	0	2	1	0	0	1	14
ECG left ventricular hy	EPH	OR	30293667	MPB/MABC	MPB	2	1	1	1	1	2	1	1	0	2	1	0	2	1	16
Effect of cancer history	EPH	OR	30771449	MPB/MABC	YE	2	1	1	1	1	2	0	1	0	2	1	2	0	1	15

Effect of estimated glo	EPH	OR	31299221	MPB/MABC	JW	2	1	1	1	1	2	0	1	2	2	1	2	2	1	19
Effect of Face-to-Face	EPH	OR	31734702	MBC/MABC	SB	4	1	1	1	1	2	1	1	0	2	1	2	2	1	20
Effect of hypertension	EPH	OR	30025949	MBC/MABC	SB	1	1	1	1	1	2	1	1	0	2	1	2	2	1	17
Effect of Prophylactic	EPH	OR	30781870	MPB/MABC	EM	2	1	1	1	1	2	1	1	0	0	1	0	0	1	12
Effect of Public Repor	EPH	OR	30998398	MPB/MABC	YE	2	1	1	1	1	0	0	1	0	2	1	0	0	1	11
Effect of real-time visu	EPH	OR	31189674	MPB/MABC	YE	4	1	1	1	1	2	1	1	0	2	1	2	0	1	18
Effect of vertical locati	EPH	OR	30953711	MPB, MABC	SC	2	1	1	1	1	2	1	1	2	2	1	0	2	1	18
Effectiveness of Basic	EPH	OR	29361872	MPB/MABC	EM	1	1	1	1	1	2	1	1	2	2	1	0	0	1	15
Effects of native langu	EPH	OR	31061026	MPB/MABC	JW	3	1	1	1	1	2	1	1	0	2	1	0	0	1	15
Emergency response fa	EPH	OR	28615216	MABC	MABC	1	1	1	1	1	2	1	1	0	2	1	2	2	1	17
Epidemiological inves	EPH	OR	30691418	MBC/MABC	SB	2	1	1	1	1	2	1	1	0	2	1	2	2	1	18
Epidemiological inves	EPH	OR	30691418	MPB, MABC	SB	2	1	1	1	1	2	1	1	0	2	1	2	0	0	15
Epidemiology of Out-	EPH	OR	29684654	MPB/MABC	EM	1	1	1	1	1	2	1	1	0	2	1	0	0	1	13
Epidemiology of pre-h	EPH	OR	31352680	MPB/MABC	JW	2	1	1	1	1	2	1	1	0	2	1	2	2	1	18
Epidemiology, etiolog	EPH	OR	31502289	MPB/MABC	YE	2	1	1	1	1	2	0	1	0	2	1	2	0	1	15
Establishment of a pre	EPH	OR	30628955	MPB/MABC	YE	2	1	1	1	1	2	1	1	0	2	1	2	0	1	16
Ethnic and Neighborho	EPH	OR	30582395	MPB/MABC	JW	2	1	1	1	1	2	1	1	0	2	1	0	2	1	16
Ethnic disparities in th	EPH	OR	31585186	MPB/MABC	MPB	2	1	1	1	1	2	0	1	0	2	1	2	2	1	17
Evaluating the awarene	EPH	OR	31463249	MPB/MABC	YE	1	1	1	1	0	2	0	1	0	2	1	0	0	2	12
Evaluation of a Standa	EPH	OR	31539339	MPB/MABC	YE	2	1	1	1	1	2	1	1	0	2	1	2	0	1	16
Exercise Related Sudd	EPH	OR	31560990	MPB/MABC	EM	1	1	1	1	1	2	1	1	0	2	1	0	0	1	13
Exercise-related out-of-	EPH	OR	30981883	MPB/MABC	JW	2	1	1	1	1	2	0	1	2	2	1	2	0	1	17
External validation of	EPH	OR	31256916	MPB/MABC	JW	2	1	1	1	1	2	1	1	0	2	1	2	2	1	18
Factors associated with	EPH	OR	31539607	MBC/MABC	SB	2	1	1	1	1	2	1	1	0	2	1	2	2	1	18
Final-year medical stud	EPH	OR	31375334	MPB, MABC	SB	1	1	1	1	1	0	0	0	0	2	1	2	0	0	10
Future projections of t	EPH	OR	31125746	MPB/MABC	YE	2	1	1	1	1	2	1	1	0	2	1	0	0	1	14
Gender Differences in	EPH	OR	30545703	MPB/MABC	EM	1	1	1	1	1	0	0	1	0	2	1	0	0	1	10
Gender differences in u	EPH	OR	31330199	MPB, MABC	SB	2	1	1	1	1	2	1	1	0	2	1	2	0	0	15
General Public's Know	EPH	OR	30896391	MPB/MABC	JW	1	1	1	1	1	2	1	1	0	2	1	2	0	0	14
Geographical Differenc	EPH	OR	31888125	MPB/MABC	YE	2	1	1	1	1	2	1	1	0	2	1	0	0	1	14
Hands-Only Cardiopul	EPH	OR	30442510	MPB/MABC	YE	3	1	1	1	1	2	1	1	2	2	1	2	2	1	21
Healthcare professional	EPH	OR	29887282	MABC	MABC	1	1	1	1	1	2	1	1	0	2	1	0	0	1	13
High Incidence and M	EPH	OR	31165598	MPB/MABC	YE	2	1	1	1	1	2	1	1	0	2	1	0	0	1	14
Hong Kong needs a te	EPH	OR	31178443	MPB, MABC	SB	1	1	1	1	1	0	0	0	0	2	0	0	0	1	8
Human factors influen	EPH	OR	30759497	MPB/MABC	EM	1	1	0	1	1	2	1	1	0	2	1	0	0	1	12
Hypertrophic Cardiom	EPH	OR	31630535	MPB/MABC	JW	2	1	1	1	1	2	0	1	0	2	1	0	2	1	15
Hypoglycaemic episod	EPH	OR	31655001	MPB/MABC	JW	2	1	1	1	1	2	1	1	2	2	1	2	2	1	20
Identifying patients at	EPH	OR	31530553	MPB/MABC	MPB	1	1	1	1	1	2	1	1	0	2	1	0	2	1	15
Impact of population a	EPH	OR	31988755	MABC	MABC	2	1	1	1	1	2	1	1	0	2	1	2	2	1	18
Impact of Sex on Surv	EPH	OR	31791546	MBC/MABC	SB	2	1	1	1	1	0	0	0	0	2	1	2	0	1	12
Impact of the consump	EPH	OR	31209087	MPB/MABC	JW	2	1	1	1	1	2	1	1	0	2	1	2	2	1	18
Implementing automat	EPH	OR	31275605	MPB/MABC	JW	1	1	1	1	1	2	1	1	0	2	1	2	0	0	14
In Hospitals With Mo	EPH	OR	31260358	MPB/MABC	EM	2	1	1	1	1	2	1	0	0	2	1	0	0	1	13
In Silico Trial of Opti	EPH	OR	31537265	MABC	MABC	2	1	1	1	1	2	1	1	0	2	1	2	2	1	18
Incidence, Mortality, a	EPH	OR	30608874	MPB, MABC	SB	1	1	1	1	1	2	0	1	0	2	1	2	0	1	14
Informed consent and	EPH	OR	28443357	MPB/MABC	JW	1	0	0	0	0	0	0	1	2	2	1	2	0	1	10
Integration of 12-Lead	EPH	OR	30361038	MBC/MABC	SB	2	1	1	1	1	2	1	1	0	2	1	2	0	1	16
International variation	EPH	OR	30898569	MPB/MABC	YE	2	1	1	1	1	2	1	1	2	2	1	0	0	1	16
Interprofessional Educa	EPH	OR	28438106	MPB/MABC	EM	2	1	1	1	1	2	1	1	2	2	1	0	0	1	16
Keeping Bystanders A	EPH	OR	31316962	MPB/MABC	MPB	3	1	1	1	0	2	1	1	0	2	1	0	2	1	16
Knowledge and Attitu	EPH	OR	30941220	MPB/MABC	YE	1	1	1	1	1	2	1	1	0	2	1	0	0	1	13
Knowledge and Attitu	EPH	OR	31106082	MPB, MABC	SB	1	1	1	1	1	2	1	1	0	2	1	2	0	1	15
Knowledge of and will	EPH	OR	31861024	MABC	MABC	1	1	1	1	1	2	1	1	0	2	1	2	2	1	17

Knowledge of cardiopu	EPH	OR	31711281	MBC/MABC	SB	1	1	1	1	1	2	1	1	0	2	1	2	0	0	14
Knowledge of Nonmec	EPH	OR	30792927	MPB, MABC	SB	1	1	1	1	1	2	1	1	0	2	1	2	0	1	15
Laparoscopic Sleeve G	EPH	OR	31872338	MPB/MABC	JW	2	1	1	1	1	2	0	1	0	2	1	2	2	1	17
Large urban center imp	EPH	OR	31009693	MPB, MABC	SB	2	1	1	1	1	2	1	1	0	2	1	2	0	1	16
Lay People training in	EPH	OR	31405209	DC	TM	1	1	1	1	0	0	0	1	2	2	1	2	2	1	15
Lay People Training ir	EPH	OR	31405209	DC	RC	2	1	1	1	0	0	0	1	2	2	1	2	2	0	15
Learning by teaching b	EPH	OR	30823878	MPB/MABC	YE	3	1	1	1	1	2	1	1	2	2	1	0	0	1	17
Learning by teaching b	EPH	OR	30823878	MPB/MABC	MPB	3	1	1	1	1	2	1	1	0	2	1	2	0	1	17
Lived Experiences of S	EPH	OR	30329171	MPB/MABC	EM	1	1	1	1	1	2	1	1	0	2	1	0	0	1	13
Logistic early warning	EPH	OR	31270799	MPB/MABC	YE	2	1	1	1	0	2	1	0	2	0	0	0	0	0	10
LONG-TERM SURVI	EPH	OR	31262547	MPB, MABC	SB	2	1	1	1	1	2	1	1	0	2	1	2	0	1	16
Long-term Trends in tl	EPH	OR	31513862	MPB/MABC	EM	1	1	1	1	1	2	1	1	0	2	1	0	0	1	13
Lower Proportion of F	EPH	OR	31607503	MPB/MABC	MPB	2	1	1	1	1	2	1	1	0	2	1	0	0	1	14
Lower socioeconomic	EPH	OR	31165498	MPB, MABC	SC	2	1	1	1	1	2	1	1	2	2	1	0	0	1	16
Management and outco	EPH	OR	31129228	MPB/MABC	YE	2	1	1	1	1	2	1	1	0	2	1	0	0	1	14
Marriage Dissatisfactic	EPH	OR	30352663	MBC/MABC	SB	2	1	1	1	1	2	1	1	0	0	1	0	0	1	12
Maternal cardiac arrest	EPH	OR	31051417	MPB/MABC	EM	1	1	1	1	1	2	1	1	0	2	1	0	0	1	13
Maternal out-of-hospit	EPH	OR	30562597	MABC	MABC	2	1	1	1	1	2	1	1	0	2	1	2	2	1	18
Measuring Health Care	EPH	OR	31783966	MPB/MABC	EM	2	1	1	1	1	2	1	1	2	2	1	0	0	1	16
Medical encounters, ca	EPH	OR	31371337	MPB/MABC	EM	1	1	1	1	1	2	1	1	0	0	0	0	0	1	10
Neurological outcomes	EPH	OR	31714474	MBC/MABC	SB	2	1	1	1	1	2	1	1	0	2	1	2	2	1	18
Nurse Vigilance: A Th	EPH	OR	31206418	MPB/MABC	EM	1	1	0	1	1	2	1	1	0	2	1	0	0	1	12
Nursing knowledge of	EPH	OR	30746310	MPB/MABC	JW	2	1	1	1	1	2	0	1	0	2	1	2	2	1	17
Out of hospital cardiac	EPH	OR	31401136	MPB/MABC	JW	2	1	1	1	1	2	1	1	2	2	1	0	2	1	18
Out-of-hospital cardiac	EPH	OR	31517891	MPB/MABC	YE	2	1	1	1	1	0	0	1	0	2	1	2	0	1	13
Out-of-Hospital Cardia	EPH	OR	30654927	MPB/MABC	YE	2	1	1	1	1	2	1	1	0	2	1	0	0	1	14
Out-of-hospital cardiac	EPH	OR	30481998	MPB/MABC	JW	2	1	1	1	1	2	1	1	2	2	1	0	0	1	16
Out-of-hospital cardiac	EPH	OR	31504477	MPB/MABC	MPB	2	1	1	1	1	2	0	1	2	2	1	0	0	1	15
Out-of-hospital cardiac	EPH	OR	31325557	MABC	MABC	2	1	1	1	1	2	1	1	0	2	1	2	2	1	18
Out-of-hospital cardiac	EPH	OR	31325557	MPB, MABC	SB	2	1	1	1	1	2	1	1	0	2	1	2	0	1	16
Out-of-hospital cardiac	EPH	OR	31311316	MPB/MABC	JW	2	1	1	1	1	0	0	1	0	2	1	2	2	1	15
Outcome after pre-hosp	EPH	OR	30831217	MPB/MABC	JW	2	1	1	1	1	2	0	1	2	2	1	2	0	1	17
Outcome of exercise-re	EPH	OR	30707745	MPB/MABC	YE	2	1	1	1	1	2	0	1	2	2	1	0	0	1	15
Outcomes and modifia	EPH	OR	30855446	MPB, MABC	SC	2	1	1	1	1	2	1	1	2	2	1	0	0	1	16
Outcomes of Patients (	EPH	OR	30924892	MPB/MABC	EM	1	1	1	1	1	1	1	1	0	2	2	0	0	1	13
Outcomes of Patients (	EPH	OR	30924892	MPB, MABC	SB	2	1	1	1	1	2	1	1	0	2	1	2	0	1	16
Outcomes of patients a	EPH	OR	30236610	MPB/MABC	YE	2	1	1	1	1	0	0	1	2	2	1	2	0	1	15
Ozone and cardiac arres	EPH	OR	30399483	MBC/MABC	SB	1	1	1	1	1	2	1	1	0	2	1	2	2	1	17
Patient, Neighborhood	EPH	OR	31287347	MPB/MABC	YE	2	1	1	1	1	2	1	1	0	2	1	2	2	1	18
Patients' knowledge ar	EPH	OR	30969861	MPB/MABC	JW	2	1	1	1	1	2	0	1	0	2	1	2	2	1	17
Perceptions of Risk of	EPH	OR	31501048	MPB/MABC	YE	1	1	1	1	1	2	0	1	0	2	1	2	2	1	16
Plakophilin-2 Truncati	EPH	OR	30678776	MPB/MABC	MPB	1	1	1	1	1	2	1	1	0	2	1	0	0	1	13
Post-Mortem Imaging	EPH	OR	30340963	MPB/MABC	EM	1	1	1	1	1	0	0	0	0	2	1	0	0	1	9
Postconvulsive central	EPH	OR	30568003	MPB/MABC	MPB	2	1	1	1	1	2	1	1	0	2	1	0	0	1	14
Postmortem Interrogat	EPH	OR	30238160	MPB/MABC	EM	2	1	1	1	1	2	1	0	0	2	1	0	0	1	13
Postoperative high-sen	EPH	OR	30701514	MPB/MABC	JW	2	0	1	1	0	2	0	1	2	2	1	2	0	1	15
Pre-hospital outcomes	EPH	OR	31352690	MPB/MABC	YE	2	1	1	1	1	2	1	1	0	2	1	0	0	1	14
Predicting Cardiac Arr	EPH	OR	31470543	MPB/MABC	JW	2	1	1	1	1	2	0	1	0	2	1	2	0	1	15
Prediction and Prevent	EPH	OR	31586529	MABC	MABC	2	1	1	1	1	2	1	1	0	2	1	2	2	1	18
Predictors of in-hospit	EPH	OR	30887710	MPB, MABC	SB	2	1	1	1	1	2	1	1	0	2	1	2	0	0	15
Preferences of patients'	EPH	OR	29699912	MPB/MABC	JW	1	1	1	1	1	2	1	1	0	2	1	2	0	1	15
Prehospital Death Afte	EPH	OR	30898288	MPB/MABC	JW	1	1	1	1	1	2	0	0	0	2	1	2	0	0	12
Prevalence of spontane	EPH	OR	30569504	MPB/MABC	MPB	2	1	1	1	1	0	0	1	0	2	1	0	0	1	11

Primary Prevention of	EPH	OR	30678832	MBC/MABC	SB	1	1	1	1	1	2	1	1	0	2	1	2	2	1	17
Public knowledge, atti	EPH	OR	30190091	MPB/MABC	MPB	1	1	1	1	1	2	1	1	0	2	1	0	2	1	15
Public location and su	EPH	OR	31350130	MPB, MABC	SB	2	1	1	1	1	2	1	1	0	2	1	2	0	1	16
Public perception towa	EPH	OR	31473603	MABC	MABC	1	1	1	1	1	2	1	1	0	2	1	2	2	1	17
Public Perceptions on	EPH	OR	30779655	MPB/MABC	YE	1	1	1	1	1	2	0	1	2	2	1	2	2	1	18
Public-access automate	EPH	OR	30500911	MBC/MABC	SB	2	1	1	1	1	2	1	1	0	2	1	2	2	1	18
Public-access automate	EPH	OR	31400888	MPB/MABC	EM	2	1	1	1	1	2	1	1	0	2	1	0	0	1	14
Public-Access Defibril	EPH	OR	31204367	MPB/MABC	YE	2	1	1	1	1	2	1	1	2	2	1	2	0	1	18
Quantification of Vent	EPH	OR	29994685	MABC	MABC	1	1	1	1	1	2	1	1	0	2	1	2	2	1	17
Racial and ethnic diffe	EPH	OR	30753852	MPB, MABC	SB	2	1	1	1	1	2	1	1	0	2	1	2	0	0	15
Racial Differences in S	EPH	OR	30712378	MPB/MABC	EM	2	1	1	1	1	2	1	1	2	2	1	2	0	1	18
Randomised controlle	EPH	OR	31015214	MPB/MABC	JW	4	1	1	1	1	2	1	1	0	2	1	2	2	1	20
Randomized Controlle	EPH	OR	30643596	MPB/MABC	EM	2	1	1	1	1	2	1	1	2	2	1	2	2	1	20
Rationale and Design	EPH	OR	31753903	MPB/MABC	EM	1	1	1	1	1	2	1	0	2	2	1	0	0	1	14
Regional trends in In-l	EPH	OR	31408680	MPB, MABC	SB	2	1	1	1	1	2	1	1	0	2	1	2	0	1	16
Relationship between	EPH	OR	30802556	MPB/MABC	EM	1	1	1	1	1	2	1	1	0	2	1	2	0	1	15
Resuscitation for out-c	EPH	OR	30414749	MABC	MABC	1	1	1	1	1	2	1	1	0	2	1	2	2	1	17
Revised Cardiac Risk	EPH	OR	30005044	MABC	MABC	2	1	1	1	1	2	1	1	0	2	1	2	2	1	18
Risk factors associated	EPH	OR	31723930	MBC/MABC	SB	2	1	1	1	1	2	1	1	0	2	1	2	2	1	18
Risk of Out-of-Hospit	EPH	OR	31801927	MPB/MABC	EM	2	1	1	1	1	2	1	1	0	2	1	0	0	1	14
Risk prediction of futu	EPH	OR	31759070	MPB/MABC	YE	2	1	1	1	1	2	0	1	0	2	1	0	0	1	13
RNA sequencing revea	EPH	OR	31419596	MBC/MABC	SB	2	1	1	1	1	2	1	1	0	2	1	2	2	1	18
Role of Angiotensin-C	EPH	OR	30242890	MPB/MABC	EM	2	1	1	1	1	2	1	1	0	2	1	2	0	1	16
Self-learning training	EPH	OR	30926451	MPB/MABC	EM	4	1	1	1	1	2	1	1	2	2	1	2	2	1	22
Sex Differences in "Dc	EPH	OR	31047712	MPB/MABC	YE	2	1	1	1	1	2	1	1	2	2	1	2	0	1	18
Sex Differences in Ele	EPH	OR	31558356	MPB/MABC	JW	2	1	1	1	1	2	1	0	2	2	1	2	0	1	17
Sex differences in the	EPH	OR	30735739	MPB/MABC	EM	2	1	1	1	1	2	1	1	2	2	1	2	0	1	18
Sex Disparities in Rec	EPH	OR	31150086	MPB, MABC	SB	2	1	1	1	1	2	1	1	0	2	1	2	0	1	16
Sex Disparities in R	EPH	OR	31150086	MPB/MABC	MPB	2	1	1	1	1	2	1	1	0	2	1	2	2	1	18
Sex-Based Disparities	EPH	OR	30922691	MPB/MABC	YE	2	1	1	1	1	2	1	1	0	2	1	2	2	1	18
Sex-specific difference	EPH	OR	31345244	MABC	MABC	1	1	1	1	1	2	1	1	0	2	1	2	2	1	17
Sex-specific difference	EPH	OR	31345244	MPB/MABC	YE	2	1	1	1	1	2	1	1	0	2	1	0	0	1	14
Simulating blood pres	EPH	OR	31437806	MPB, MABC	SB	2	1	1	1	1	2	1	1	0	2	1	2	2	1	18
Socioeconomic status	EPH	OR	29303842	MBC/MABC	SB	1	1	1	1	1	2	1	1	0	2	1	0	0	1	13
Sudden Cardiac Arrest	EPH	OR	31618064	MPB/MABC	YE	2	1	1	1	1	2	1	1	2	2	1	2	0	1	18
Sudden Cardiac Arrest	EPH	OR	31588605	MPB/MABC	EM	2	1	1	1	1	2	1	1	0	2	1	2	0	1	16
Sudden cardiac arrest i	EPH	OR	30425046	MPB/MABC	JW	1	1	1	1	1	2	1	1	0	2	1	2	0	1	15
Sudden Cardiac Arrest	EPH	OR	31705910	MPB/MABC	EM	2	1	1	1	1	2	1	1	0	2	1	0	0	1	14
Sudden Cardiac Death	EPH	OR	30827144	MPB/MABC	JW	2	0	1	1	1	2	1	1	0	2	1	2	2	1	17
Sudden cardiac death i	EPH	OR	31852072	MPB/MABC	YE	2	1	1	1	1	2	0	1	0	2	1	2	0	1	15
Sudden Cardiac Death	EPH	OR	30779638	MPB/MABC	JW	2	1	1	1	1	2	1	1	2	2	1	2	2	1	20
Sudden Cardiac Death	EPH	OR	30808515	MPB/MABC	MPB	2	1	1	1	1	0	1	1	0	2	1	0	0	1	12
Survival after cardiopu	EPH	OR	30905924	MPB, MABC	SB	2	1	1	1	1	2	1	1	0	2	1	2	0	1	16
Survival After Exercise	EPH	OR	30204540	MPB/MABC	MPB	2	1	1	1	1	2	1	1	0	2	1	2	2	1	18
Survival after out-of-h	EPH	OR	30327393	MPB/MABC	JW	2	1	1	1	1	2	1	1	0	2	1	2	2	1	18
Survival of patients wi	EPH	OR	30632777	MPB/MABC	EM	2	1	1	1	1	2	1	1	0	2	1	2	0	1	16
Teaching Cardiopulmo	EPH	OR	30879607	MPB/MABC	EM	2	1	1	0	0	0	0	0	0	2	1	0	2	0	9
Temporal Trends in th	EPH	OR	31512958	MPB/MABC	JW	2	1	1	1	1	2	0	1	0	2	1	0	0	1	13
The association betwe	EPH	OR	31129227	MPB/MABC	JW	2	1	1	1	1	2	1	1	2	2	1	2	0	0	17
The congenital disorde	EPH	OR	31563034	MPB/MABC	JW	1	1	1	1	0	2	1	1	0	2	1	2	0	1	14
The Effect of Rurality	EPH	OR	28842929	MPB/MABC	MPB	2	1	1	1	1	0	0	1	0	2	1	0	2	1	13
The effectiveness of te	EPH	OR	30921176	MPB/MABC	EM	1	1	1	1	1	2	1	1	2	2	1	2	2	1	19
The epidemiology of i	EPH	OR	31462205	MPB/MABC	JW	2	1	1	1	1	2	0	1	2	2	1	2	0	0	16

The Feasibility of a N	EPH	OR	31757671	MPB/MABC	EM	1	1	1	1	0	2	0	0	2	2	1	2	0	1	14
The impact of psychol	EPH	OR	30586654	MABC	MABC	1	1	1	1	1	2	1	1	0	2	1	2	2	1	17
The influence of past e	EPH	OR	31830912	MABC	MABC	2	1	1	1	1	2	1	1	0	2	1	2	2	1	18
The narrow-sense and	EPH	OR	30297186	MPB/MABC	YE	2	1	1	1	1	2	0	1	0	2	1	0	0	1	13
The Relation of Emerg	EPH	OR	30567633	MPB/MABC	JW	2	0	1	1	1	2	1	1	0	2	1	2	2	1	17
The role of heart rate v	EPH	OR	30476644	MPB/MABC	MPB	2	1	1	1	1	2	1	1	0	2	1	0	0	1	14
The timing of testing i	EPH	OR	31801502	MABC	MABC	2	1	1	1	1	2	1	1	0	2	1	2	2	1	18
The utilization of auto	EPH	OR	29588095	MBC/MABC	SB	1	1	1	1	1	2	1	1	0	2	1	0	2	1	15
Thrombolysis During	EPH	OR	31381884	MPB/MABC	YE	2	1	1	1	1	2	1	1	2	2	1	0	0	1	16
Time of out-of-hospita	EPH	OR	31326405	MPB/MABC	EM	2	1	1	1	1	2	1	1	0	2	1	2	2	1	18
To Enhance the Qualit	EPH	OR	31590653	MPB/MABC	EM	2	1	1	1	1	2	1	1	2	2	1	0	0	1	16
TpTe and TpTe/QT: n	EPH	OR	30118535	MPB/MABC	JW	2	1	1	1	1	2	0	0	0	2	1	0	0	1	12
Trapped in a disrupted	EPH	OR	31887365	MPB/MABC	MPB	1	1	1	1	1	2	1	1	0	2	1	2	2	1	17
Trends in overdose-rel	EPH	OR	30352247	MBC/MABC	SB	1	1	1	1	1	2	1	1	0	2	1	2	2	1	17
Two major earthquake	EPH	OR	31051009	MABC	MABC	2	1	1	1	1	2	1	1	0	2	1	2	2	1	18
Unexpected High Prev	EPH	OR	30661423	MPB, MABC	SB	1	1	1	1	1	2	1	1	0	2	1	2	0	1	15
Unexpected shift in cir	EPH	OR	30193852	MPB/MABC	MPB	2	1	1	1	1	2	0	1	0	2	1	0	0	1	13
Use of a Virtual Realit	EPH	OR	31490865	MBC/MABC	SB	1	1	1	1	1	2	1	1	0	2	1	2	0	1	15
Usefulness of Focused	EPH	OR	30348435	MPB/MABC	EM	2	1	1	1	1	2	1	1	0	0	0	0	0	1	11
Usefulness of Genetic	EPH	OR	30975432	MPB/MABC	YE	2	1	1	1	1	2	0	1	0	2	1	0	0	1	13
Utilization of implant	EPH	OR	31561030	MPB/MABC	YE	2	1	1	1	1	2	1	1	0	2	1	0	0	1	14
Variability in survival	EPH	OR	30771450	MPB, MABC	SB	2	1	1	1	1	2	1	1	0	2	1	2	0	0	15
Variation in Bystander	EPH	OR	31887076	MPB/MABC	JW	2	1	1	1	1	2	1	1	0	2	1	2	0	1	16
Variation in Survival	EPH	OR	31767599	MPB/MABC	EM	1	1	1	1	1	2	1	1	0	2	1	2	0	1	15
Vulnerability to cardia	EPH	OR	31452398	MPB, MABC	SB	2	1	1	1	1	0	0	0	0	2	1	2	0	0	11
What Do Adolescents	EPH	OR	30909545	MBC/MABC	SB	3	1	1	1	1	2	1	1	0	2	1	2	2	1	19
What Do Adolescents	EPH	OR	30909545	MPB/MABC	JW	2	1	1	0	1	2	0	1	0	2	1	2	0	0	13
When is a bystander n	EPH	OR	30572073	MPB/MABC	MPB	1	1	1	1	1	2	0	1	2	2	1	0	2	1	16
When the Female Hear	EPH	OR	31053294	MPB, MABC	SB	2	1	1	1	1	2	1	1	0	2	1	2	0	1	16
Willingness and Obsta	EPH	OR	31494073	MPB/MABC	EM	1	1	1	1	1	2	1	1	2	0	1	0	0	1	13
Women have lower ch	EPH	OR	31112998	MPB, MABC	SC	2	1	1	1	1	2	1	1	2	2	1	0	0	1	16
Prevalence and Outcon	EPM	OR	31543736	MPB/MABC	JW	1	1	1	1	1	2	1	1	0	2	1	0	0	1	13
Amplitude-Integrated I	IN	OR	31381485	KD	CH	2	1	1	1	1	2	1	1	2	2	1	2	2	1	20
A comparison of intra	IN	OR	31348276	CH	DB	2	1	1	1	1	2	1	1	2	2	1	2	2	1	20
A comparison of the al	IN	OR	30287355	CH	KD	2	1	1	1	1	0	0	1	2	2	1	2	2	1	17
A new paradigm for lu	IN	OR	31447395	CH	RL	3	1	1	1	1	2	1	1	0	2	1	0	0	1	15
A Population-Based A	IN	OR	31651456	CH	DC	2	1	1	1	1	2	1	1	2	2	1	0	0	1	16
A review of Code Blu	IN	OR	31633855	CH	DB	1	0	0	1	1	2	0	1	2	2	1	2	0	1	14
A survey of ventilatio	IN	OR	31534596	CH	DB	1	0	1	1	1	2	1	1	2	2	1	0	0	1	14
Adaptation of global h	IN	OR	28695976	CH	GS	2	1	1	1	1	2	1	1	2	2	1	2	2	1	20
Admission Society fo	IN	OR	31710843	CH	KD	2	1	1	1	1	2	1	1	2	2	1	2	2	1	20
Adrenaline, ROSC an	IN	OR	31125581	KD	CH	2	1	1	1	1	2	0	1	2	2	1	2	0	1	17
After COACT trial-ne	IN	OR	31660312	CH	RL	4	1	1	0	1	0	0	1	2	2	1	2	2	1	18
An Analysis Using M	IN	OR	31198281	CH	DC	2	1	1	1	1	2	1	1	2	2	1	0	0	1	16
An outcome study of	IN	OR	31864867	CH	DC	2	1	1	1	1	2	1	1	2	2	1	0	0	1	16
Assessment of cardiop	IN	OR	31714118	CH	KD	1	1	1	1	1	2	1	0	0	0	0	2	0	1	11
Association between e	IN	OR	31466139	CH	DB	2	1	1	1	1	2	1	1	2	2	1	2	0	1	18
Association between h	IN	OR	30871337	CH	DB	2	0	1	1	1	2	1	1	2	2	1	2	2	1	19
Association Between I	IN	OR	30871337	CH	GS	2	1	1	1	1	2	1	1	2	2	1	0	0	1	16
Association between h	IN	OR	31303537	CH	RL	2	1	1	0	1	0	0	1	0	2	1	2	2	1	14
Association Between I	IN	OR	31526205	CH	GS	2	1	1	1	1	2	1	1	2	2	1	2	0	1	18
Association between r	IN	OR	31394154	CH	DC	2	1	1	1	1	2	1	1	2	2	1	2	2	1	20
Association between s	IN	OR	31377392	CH	GS	2	1	1	1	1	2	0	1	2	2	1	2	0	1	17

Association Between T	IN	OR	30276614	CH	KD	2	1	1	1	1	2	1	1	2	2	1	2	2	1	20
Association Between T	IN	OR	31200920	CH	RL	2	1	1	1	1	2	1	1	2	2	1	2	2	1	20
Association of acute m	IN	OR	30597584	CH	DC	2	1	1	1	1	2	0	1	2	2	1	0	0	1	15
Association of acute m	IN	OR	30597584	CH	GS	2	1	1	1	1	2	0	1	0	0	1	0	0	0	10
Association of antiepil	IN	OR	31325554	CH	DC	2	1	1	1	1	2	1	1	2	2	1	2	0	1	18
Association of extracor	IN	OR	30819234	CH	RL	2	1	1	0	1	2	1	1	0	2	1	0	2	1	15
Association of sudden	IN	OR	30862425	CH	DB	2	0	1	1	1	2	0	1	2	2	1	0	0	1	14
Association of Vitami	IN	OR	31490356	CH	GS	2	1	1	1	1	2	1	1	2	2	1	2	2	1	20
Automated detection o	IN	OR	31627019	CH	DB	1	0	1	1	0	0	0	0	2	2	1	2	2	1	13
Basic life support trair	IN	OR	31505232	CH	DB	4	1	1	1	1	2	1	1	2	2	1	2	2	1	22
Bedside Interpretation	IN	OR	31465539	CH	SC	1	1	1	1	1	2	1	1	2	2	1	0	0	1	15
Better Cardiac Arrest (	IN	OR	31567258	CH	KD	2	1	0	0	0	0	0	0	0	2	1	2	2	1	11
Bleeding Complicatio	IN	OR	30523732	CH	RL	2	1	1	1	1	2	1	1	2	2	1	2	2	1	20
Cangrelor in cardiogen	IN	OR	30790690	CH	DC	2	1	1	1	1	2	1	1	2	2	1	0	0	1	16
Capnography: A suppo	IN	OR	31005583	CH	DB	2	0	1	1	1	0	0	1	2	2	1	2	2	0	15
Cardiac arrest and subs	IN	OR	30889030	CH	DB	2	0	1	1	1	2	1	1	0	2	1	0	0	1	13
Cardiac Arrest in the C	IN	OR	31537284	CH	DC	4	1	1	1	1	2	1	1	0	2	1	2	0	1	18
Cardiac arrest in the in	IN	OR	31521775	CH	DB	1	1	1	1	1	2	0	1	2	2	1	0	0	1	14
Cardiopulmonary resus	IN	OR	31126275	CH	DB	1	0	1	1	1	2	1	1	2	2	1	0	0	1	14
Care After REsuscitati	IN	OR	31287385	KD	CH	2	1	1	1	1	0	0	1	2	2	1	2	0	1	15
Challenges in the deve	IN	OR	31887368	CH	GS	1	1	1	1	0	2	1	1	0	2	1	0	0	1	12
Change in myocardial	IN	OR	31022454	CH	GS	2	1	1	1	1	2	0	0	2	2	1	2	2	1	18
Characteristics and out	IN	OR	30401593	CH	KD	2	1	1	1	1	2	1	1	0	2	1	0	0	1	14
Characteristics and Ou	IN	OR	31819835	CH	RL	2	1	1	1	0	2	1	1	2	2	1	2	2	1	19
Clinical Factors Assoc	IN	OR	31172695	CH	GS	2	1	1	1	1	2	1	1	2	2	1	2	2	1	20
Cluster randomised co	IN	OR	30683057	KD	CH	2	1	1	1	1	2	1	1	0	2	1	2	0	1	16
Cluster randomised co	IN	OR	30683057	CH	CH	3	1	1	1	1	2	1	1	0	2	1	0	0	1	15
Coagulation Derangem	IN	OR	30655200	CH	RL	2	1	1	1	1	2	0	0	0	2	1	2	2	0	15
Code blue pit crew mo	IN	OR	31299222	CH	GS	1	1	1	1	1	2	1	1	2	2	1	2	2	1	19
Comparing in-patient (	IN	OR	31296118	CH	GS	2	1	1	1	1	2	0	1	0	2	1	0	0	1	13
Comparison of clinical	IN	OR	31371275	CH	RL	2	1	1	1	1	2	1	1	0	2	1	2	2	0	17
Comparison of in-hosp	IN	OR	31702660	CH	DC	2	1	1	0	1	2	1	1	2	2	1	2	0	1	17
Controlled automated	IN	OR	31293795	CH	DB	1	0	0	1	0	2	1	1	2	2	1	2	0	1	14
COOL-ARREST: Res	IN	OR	29883298	CH	DB	2	0	1	1	1	2	1	1	2	2	1	2	0	1	17
Coronary angiographic	IN	OR	31560991	CH	GS	2	1	1	1	1	2	1	1	2	2	1	2	2	1	20
Coronary angiography	IN	OR	30883057	CH	DB	4	1	1	1	1	2	1	1	2	2	1	0	0	0	17
Cost-effectiveness of e	IN	OR	31473264	CH	KD	0	1	1	1	1	0	1	1	2	2	1	2	0	1	14
Cut-off values of serur	IN	OR	31022496	CH	DC	2	1	1	1	1	2	1	1	0	2	1	2	0	1	16
Data and methods to c	IN	OR	31890782	CH	RL	2	1	1	0	1	0	0	1	0	2	1	0	2	1	12
Death after awakening	IN	OR	30944013	KD	CH	2	1	1	1	1	2	1	1	2	0	0	0	0	1	13
Decision tree model fo	IN	OR	31509271	CH	DC	2	1	1	1	1	2	1	1	2	2	1	2	2	1	20
Decision-making in ca	IN	OR	30588135	CH	DB	1	0	1	1	1	2	1	1	2	2	1	2	2	0	17
Diagnostic value of lea	IN	OR	31330201	CH	GS	2	1	1	1	1	2	1	1	2	2	1	2	2	1	20
Dimensional structure	IN	OR	30927582	CH	CH	2	1	1	1	1	2	1	1	2	2	1	0	2	1	18
Dimensional structure	IN	OR	30927582	CH	RL	2	1	1	1	1	2	1	1	2	2	1	0	0	1	16
Direct or subacute cor	IN	OR	31028826	CH	RL	4	1	1	1	1	2	1	1	0	2	1	2	2	1	20
Disability-Adjusted Li	IN	OR	30859852	KD	CH	2	1	1	1	1	2	1	1	2	2	1	0	0	1	16
Does Point-of-care Ult	IN	OR	31205842	CH	RL	2	1	1	1	1	2	1	1	0	2	1	2	2	1	18
Door-to-targeted temp	IN	OR	31955981	CH	DB	2	1	1	1	1	2	1	1	2	2	1	2	2	1	20
Door-to-Targeted Tem	IN	OR	31055981	CH	GS	2	1	1	1	1	2	1	1	2	2	1	2	0	1	18
Door-to-Targeted Tem	IN	OR	31055981	CH	KD	2	1	1	1	1	2	1	1	0	2	1	2	2	1	18
Duration of in-hospital	IN	OR	31779859	CH	RL	2	1	1	1	1	2	1	1	2	2	1	2	2	1	20
Dynamic determinatio	IN	OR	31828754	CH	RL	2	1	1	1	1	2	1	1	2	2	1	2	2	1	20

Early full-body compu	IN	OR	31811881	CH	DC	2	1	1	1	1	0	0	1	2	2	1	2	2	0	16
Early goal-directed hae	IN	OR	30895296	CH	DC	4	1	1	1	1	2	1	1	2	2	1	2	0	1	20
Early goal-directed hae	IN	OR	30895296	CH	DC	4	1	1	1	1	2	1	1	2	2	1	2	0	1	20
Early initiation of cont	IN	OR	30335673	CH	DB	4	1	1	1	1	2	1	1	2	2	1	2	2	0	21
Early termination of re	IN	OR	31328251	CH	GS	2	1	1	1	1	2	1	1	2	0	1	0	0	1	14
Early termination of re	IN	OR	31328251	CH	GS	2	1	1	1	1	2	1	1	0	2	1	0	0	1	14
Early vs. delayed in-ho	IN	OR	31759071	CH	KD	2	1	1	1	1	0	0	1	2	2	1	2	2	1	17
Early whole-body CT	IN	OR	31468207	CH	DC	2	1	1	0	0	2	0	1	2	2	1	2	0	0	14
Early withdrawal of lif	IN	OR	30836171	KD	CH	2	1	1	1	1	2	1	1	2	2	1	2	2	1	20
Early withdrawal of lif	IN	OR	30836171	CH	RL	2	1	1	1	1	2	0	1	2	2	1	2	2	1	19
Effect of interplay betv	IN	OR	30547322	CH	GS	2	1	1	1	1	2	1	1	2	2	1	2	2	1	20
Effect of Mean Blood	IN	OR	31080955	CH	DC	2	1	1	1	1	2	1	1	2	2	1	0	0	1	16
Effect of Public Repor	IN	OR	30998398	CH	DC	2	1	1	1	1	0	0	1	2	2	1	0	0	1	13
Effect of selenium on	IN	OR	29556681	CH	DB	2	1	1	1	1	2	1	1	2	2	1	2	0	1	18
Effect of selenium on	IN	OR	29556681	CH	CH	2	1	1	1	1	2	1	1	0	2	1	2	2	1	18
Effect of the floor leve	IN	OR	31737368	CH	DB	2	0	1	1	1	2	0	1	2	2	1	0	0	0	13
Effect of Trans-Nasal I	IN	OR	31063573	KD	CH	4	1	1	1	1	2	1	1	2	2	1	2	0	1	20
Effect of ulinastatin co	IN	OR	31616513	CH	GS	3	1	1	1	1	2	1	1	2	2	1	0	0	1	17
Effect of Using a Card	IN	OR	31772537	CH	KD	3	1	1	1	1	2	1	1	0	0	1	2	2	1	17
Effectiveness of the he	IN	OR	31988762	CH	GS	2	1	1	1	1	2	1	1	2	2	1	2	2	1	20
Elimination of glutam	IN	OR	31557520	CH	KD	4	1	1	1	1	2	1	1	0	2	1	2	0	1	18
EMERGENCY PHYS	IN	OR	31031069	KD	CH	2	1	1	1	1	2	1	1	2	2	1	2	0	1	18
Emergent airway mana	IN	OR	31795993	CH	KD	2	1	1	1	1	2	1	1	0	2	1	2	0	1	16
End stage renal disease	IN	OR	31796232	CH	RL	2	1	1	1	1	2	1	1	2	2	1	2	2	1	20
Endothelial Dysfunctio	IN	OR	31710844	CH	DC	4	1	1	1	1	2	1	1	2	2	1	0	0	1	18
Epinephrine, inodilato	IN	OR	31533785	CH	CH	2	1	1	1	1	2	1	1	2	2	1	2	2	1	20
Epinephrine, inodilato	IN	OR	31533785	CH	RL	2	1	1	1	1	2	1	1	2	2	1	2	2	1	20
Establishing extracorpo	IN	OR	30774958	CH	DC	2	1	1	1	1	2	0	1	2	0	0	0	0	1	12
Evaluation of out-of-ho	IN	OR	30779977	CH	DC	2	1	1	1	1	2	0	1	2	2	1	2	0	1	17
Evaluation of self-repo	IN	OR	31824739	CH	RL	2	1	1	1	0	2	1	1	0	0	1	0	0	1	11
Experience of extracorp	IN	OR	30820972	KD	CH	2	1	1	1	1	2	1	1	2	2	1	2	0	1	18
ExtraCorporeal life sup	IN	OR	30612896	CH	RL	2	1	1	1	1	2	0	1	0	2	1	2	0	1	15
Extracorporeal membra	IN	OR	31462264	CH	GS	4	1	1	1	1	2	1	1	2	2	1	0	0	1	18
Extracorporeal membra	IN	OR	31826642	CH	DB	2	0	1	1	1	0	0	1	2	2	1	0	0	1	12
Extracorporeal rewarmi	IN	OR	31219201	CH	DC	3	1	1	1	1	2	1	1	2	2	1	0	0	1	17
Extracorporeal rewarmi	IN	OR	31219201	CH	DC	3	1	1	1	1	2	1	1	2	2	1	0	0	1	17
Extubation of patients	IN	OR	30044174	CH	DB	2	0	1	1	1	0	1	1	2	2	1	2	0	1	15
Factors associated with	IN	OR	30845157	CH	RL	2	1	1	1	1	2	1	1	0	2	1	2	2	1	18
Factors associated with	IN	OR	30711387	CH	DB	1	1	1	1	1	2	1	1	2	2	1	0	0	1	15
Factors for modifying	IN	OR	31129440	CH	DC	2	1	1	1	1	2	0	1	2	2	1	2	2	1	19
Factors Predicting Bac	IN	OR	30575443	CH	GS	2	1	1	1	1	2	1	1	2	2	1	2	0	1	18
Female Physician Lea	IN	OR	30303843	CH	RL	2	1	1	1	1	0	0	1	0	2	1	0	0	1	11
Forehead electrodes su	IN	OR	30788811	CH	CH	2	1	1	1	1	2	1	1	2	2	1	2	0	1	18
Forehead electrodes su	IN	OR	30788811	CH	GS	2	1	1	1	1	2	1	1	2	2	1	2	0	1	18
Frailty is associated w	IN	OR	31369792	CH	DB	2	0	1	1	1	2	1	1	0	2	1	2	2	1	17
Functional outcomes a	IN	OR	31706964	CH	KD	2	1	1	1	1	2	1	1	2	2	1	2	0	1	18
Gender disparities in p	IN	OR	30563798	CH	RL	2	1	1	1	1	2	0	0	2	2	1	2	2	1	18
Guideline removal of a	IN	OR	30771452	KD	CH	2	1	1	1	1	0	0	1	2	2	1	2	2	1	17
Hospitals' extracorpore	IN	OR	30668963	CH	DB	2	0	1	1	1	2	1	1	2	2	1	2	2	1	19
How Feasible is Extra	IN	OR	31938615	CH	DC	1	1	1	1	1	2	1	1	2	2	1	0	0	1	15
Hypothermic to ischen	IN	OR	31830304	CH	DC	4	1	1	1	1	2	1	1	2	2	1	2	0	1	20
Identification of a pote	IN	OR	31684934	CH	DC	2	1	1	1	1	2	1	1	0	2	1	2	2	0	17
Impact of Extracorpore	IN	OR	31679644	CH	KD	2	1	1	1	1	2	1	1	2	2	1	2	2	1	20

Impact of Extracorpore	IN	OR	31679644	CH	CH	2	1	1	1	1	2	1	1	2	2	1	2	0	1	18
Impact of first docume	IN	OR	31108120	CH	GS	2	1	1	1	1	2	0	1	2	2	1	0	0	1	15
Impact of mild therapu	IN	OR	30799546	KD	CH	3	1	1	1	1	2	1	1	2	2	1	2	2	1	21
Impact of right ventric	IN	OR	30769125	CH	DB	2	0	1	1	1	2	1	1	2	2	1	0	0	0	14
Impact of targeted tem	IN	OR	30411236	CH	DB	2	1	1	1	1	2	1	1	0	2	1	2	2	1	18
Implantable cardiovert	IN	OR	31145795	CH	GS	2	1	1	1	1	2	1	1	0	2	1	2	0	1	16
Implementation of a L	IN	OR	31630977	CH	DB	1	0	1	1	1	2	0	1	2	2	1	2	2	1	17
Implementation of an	IN	OR	31335519	CH	DC	2	1	0	0	0	2	1	1	2	2	1	0	0	1	13
Implementation of an	IN	OR	31335519	CH	GS	1	1	1	1	1	0	0	1	0	0	1	2	0	0	9
Implications of Neurol	IN	OR	31320013	CH	KD	2	1	1	1	1	2	1	1	0	2	1	2	2	1	18
In-Hospital Cardiac Ar	IN	OR	31772514	CH	GS	2	1	1	1	1	0	0	1	0	0	1	2	0	0	10
In-hospital survival an	IN	OR	31080922	CH	DB	2	0	1	1	1	2	0	1	0	2	1	0	0	1	12
Independent risk factor	IN	OR	29762083	CH	DB	2	0	1	1	1	2	1	1	0	2	1	2	2	1	17
Influence of glycoprote	IN	OR	31300835	CH	RL	2	1	1	1	1	2	0	1	2	2	1	2	2	0	18
Initiation of Extracorp	IN	OR	31841997	CH	CH	1	1	1	1	1	2	0	1	0	2	1	2	2	1	16
Initiation of Extracorp	IN	OR	31841997	CH	GS	2	0	1	1	1	2	0	1	0	2	1	2	2	1	16
Interaction Effects Bet	IN	OR	31414970	CH	DC	2	1	1	1	1	0	0	1	2	2	1	2	2	0	16
Interhospital transfer ir	IN	OR	30974187	CH	GS	2	1	1	1	1	2	1	1	2	2	1	2	0	1	18
Interventions to reduce	IN	OR	30676710	CH	GS	1	1	1	1	1	2	1	1	2	2	1	0	0	1	15
Intra-aortic balloon pu	IN	OR	31302105	CH	RL	2	1	1	1	1	2	0	1	0	2	1	2	0	0	14
Intra-hospital transport	IN	OR	30835759	CH	DB	2	0	1	1	1	2	1	1	2	2	1	2	2	1	19
Intra-patient potassium	IN	OR	31842931	CH	DB	2	1	1	1	1	2	1	1	0	2	1	2	2	0	17
Intracranial pressure an	IN	OR	31185256	KD	CH	2	1	1	1	1	2	1	1	0	2	1	2	0	1	16
Intravascular Cooling	IN	OR	30418241	CH	DC	3	1	1	1	1	2	1	1	2	2	1	2	0	0	18
Intravascular Cooling	IN	OR	30418241	CH	CH	3	1	1	1	1	2	1	1	0	2	0	2	0	1	16
Intravascular versus su	IN	OR	30795782	CH	DB	2	0	1	1	1	2	1	1	2	2	1	2	0	1	17
Invasive Hemodynami	IN	OR	30770091	CH	DB	2	0	1	1	1	2	1	1	2	2	1	0	0	1	15
Laparotomy following	IN	OR	30949739	CH	CH	2	1	1	1	1	2	1	1	2	2	1	0	0	1	16
Laparotomy following	IN	OR	30949739	CH	GS	2	1	1	1	1	2	1	1	0	2	1	0	0	1	14
Left ventricular wall fi	IN	OR	31727121	CH	DB	2	0	1	1	1	2	1	1	2	2	1	2	0	1	17
Left ventricular wall fi	IN	OR	31727121	CH	CH	2	1	1	1	1	2	1	1	2	2	1	0	0	1	16
Long-term survival in	IN	OR	31470100	CH	DB	2	0	1	1	1	2	0	1	2	2	1	2	2	1	18
Longitudinal explorati	IN	OR	30555719	CH	KD	2	1	1	0	0	2	0	0	2	2	1	2	0	1	14
Low rates of immediat	IN	OR	31857140	CH	KD	2	1	1	1	1	2	1	1	0	2	1	2	2	1	18
Malignant Arrhythmia	IN	OR	31560612	CH	RL	3	1	0	1	1	2	1	1	0	2	1	2	2	1	18
Mechanical chest comp	IN	OR	31159737	KD	CH	2	1	1	1	1	2	1	1	2	2	1	0	0	1	16
Mechanism and extent	IN	OR	30826528	CH	RL	2	1	1	0	1	2	0	1	2	0	1	2	0	0	13
Mechanisms of early g	IN	OR	30908518	CH	DC	2	1	1	1	1	2	1	1	2	2	1	0	0	1	16
Mid-term clinical outc	IN	OR	30553557	CH	RL	2	1	1	1	1	2	1	1	2	2	1	2	2	1	20
Mild therapeutic hypot	IN	OR	30799547	CH	DC	2	1	1	1	1	2	0	1	2	2	1	0	0	1	15
Mild Therapeutic Hyp	IN	OR	30016204	CH	DC	2	1	1	1	1	2	1	1	2	2	1	2	0	1	18
Mortality and costs fol	IN	OR	31529353	CH	KD	2	1	1	1	1	2	1	1	0	2	1	2	2	1	18
Myocardial injury and	IN	OR	31286469	CH	RL	2	1	1	1	1	2	0	1	0	2	1	2	2	1	17
Near-infrared spectrosc	IN	OR	31088512	CH	RL	2	1	1	1	1	2	1	1	2	2	1	2	2	0	19
Near-infrared spectrosc	IN	OR	31088512	CH	CH	4	1	1	1	1	2	1	1	2	2	1	0	0	1	18
Neurologic outcomes i	IN	OR	30981847	CH	DB	2	0	1	1	1	2	1	0	2	2	1	2	2	0	17
Neurophysiological Fi	IN	OR	31823652	CH	RL	2	1	1	1	1	2	0	1	2	2	1	2	2	1	19
New Termination-of-R	IN	OR	31050224	CH	GS	2	1	1	1	1	2	1	1	2	2	1	2	2	1	20
Nursing roles for in-hc	IN	OR	31420410	CH	RL	2	1	0	0	1	2	1	1	2	2	1	2	2	1	18
Nutrition During Targ	IN	OR	31006879	KD	CH	2	1	1	1	1	2	1	1	2	2	1	2	1	1	19
Nutrition during target	IN	OR	31006879	CH	DB	2	1	1	1	1	2	1	1	2	2	1	2	0	1	18
One-year longitudinal	IN	OR	31272987	CH	DC	2	1	1	1	1	2	1	1	2	0	0	0	0	1	13
Optimal Arterial Bloo	IN	OR	31356478	CH	GS	2	1	1	1	1	2	1	1	2	2	1	2	2	1	20

Optimal combination (	IN	OR	31411632	CH	DB	2	1	1	1	1	2	1	1	2	2	1	2	0	1	18
Optimal extracorporeal	IN	OR	31988761	CH	DC	2	1	1	1	1	2	1	1	2	2	1	2	0	1	18
Organ blood flow and	IN	OR	30375081	CH	DC	3	1	1	1	0	2	1	1	0	2	1	0	0	1	14
Organ blood flow and	IN	OR	30375081	CH	CH	3	1	1	1	0	2	1	1	0	2	0	0	0	1	13
Out of hospital cardiac	IN	OR	31815873	CH	GS	2	1	1	1	1	2	1	1	2	0	0	0	0	0	12
Out-of-hospital transes	IN	OR	30957698	CH	DB	1	0	1	1	1	0	0	0	2	2	1	2	0	1	12
Outcomes associated v	IN	OR	31255419	CH	DB	2	0	1	1	1	2	1	1	0	2	1	2	0	1	15
Outcomes of Coronary	IN	OR	31089443	KD	CH	2	1	1	0	0	2	1	1	0	2	0	0	0	1	11
Outcomes of extracorp	IN	OR	31486571	CH	KD	2	1	1	1	1	2	0	1	0	2	1	2	2	1	17
Outcomes of Impella (	IN	OR	31891791	CH	GS	2	0	1	1	1	0	0	1	0	2	1	0	0	1	10
Partial pressure of arte	IN	OR	30452939	CH	GS	2	1	1	1	1	2	0	1	2	2	1	2	2	1	19
Partial pressure of arte	IN	OR	30452939	CH	CH	1	1	1	1	1	2	0	1	2	2	1	2	2	1	18
Patient and hospital fa	IN	OR	30885828	CH	KD	2	1	1	1	1	2	0	1	2	2	1	2	0	1	17
Patients With Refracto	IN	OR	30890669	CH	DC	2	1	1	1	1	2	0	1	2	2	1	2	2	1	19
Pilot study on a rewar	IN	OR	30781943	KD	CH	2	1	1	1	1	2	1	1	2	2	1	2	0	1	18
Plasma proteomic char	IN	OR	31316602	CH	DC	2	1	1	1	1	2	1	1	2	2	1	0	0	1	16
Platelet dysfunction af	IN	OR	30716426	CH	DC	2	1	1	1	1	2	1	1	0	0	0	0	0	1	11
Postarrest steroid use r	IN	OR	30308548	CH	DB	2	1	1	1	1	2	1	1	2	2	1	2	0	1	18
Predictors of Futility i	iN	OR	30376419	CH	RL	2	1	1	1	1	2	1	1	2	2	1	2	2	1	20
PREDICTORS OF SU	IN	OR	30080744	CH	KD	2	1	1	1	1	2	1	1	0	2	1	2	0	1	16
Predictors of Survival	IN	OR	30312208	CH	DC	2	1	1	1	1	2	1	0	2	2	1	0	0	1	15
Prevention of Early Ve	IN	OR	31693806	CH	RL	4	1	1	0	1	2	1	0	2	2	1	2	2	1	20
Prognostic Factors for	IN	OR	31480615	CH	DC	2	1	1	1	1	2	0	1	2	2	1	0	0	1	15
Prolonged targeted ten	IN	OR	30572070	CH	GS	2	1	1	1	1	2	1	1	2	2	1	2	2	1	20
Questionaire survey or	IN	OR	31178438	CH	DB	1	0	1	1	1	2	1	1	2	2	1	2	2	1	18
RBM3 and CIRP expr	IN	OR	31821351	CH	DB	2	0	1	1	1	2	1	1	2	2	1	2	0	1	17
Real-time compression	IN	OR	30693086	KD	CH	4	1	1	1	1	2	1	1	2	2	1	2	2	1	22
Real-time quantitative	IN	OR	29283290	CH	GS	4	1	1	1	1	2	1	1	2	2	1	2	0	1	20
Real-world clinical exp	IN	OR	31028475	CH	DC	2	1	1	0	1	2	1	1	2	0	0	0	0	1	12
Realistic Evaluation of	IN	OR	29768987	CH	GS	2	1	1	1	1	2	1	1	2	0	1	2	0	0	15
Relationship between t	IN	OR	30359876	CH	KD	2	1	1	1	1	2	1	1	2	2	1	2	0	0	17
Renal function after ou	IN	OR	31068215	CH	GS	3	1	1	1	1	2	1	1	2	2	1	2	0	1	19
Reported practice of te	IN	OR	30857515	CH	CH	1	1	1	1	0	2	0	1	0	0	1	0	0	0	8
Reported practice of te	IN	OR	30857515	CH	GS	1	1	1	1	0	2	0	1	0	0	1	0	0	0	8
Resistin and Cardiac A	IN	OR	31881807	CH	KD	2	1	1	1	0	0	1	1	0	2	1	0	0	1	11
Results of targeted ten	IN	OR	31736476	CH	RL	2	1	1	1	1	0	0	1	2	2	1	2	2	1	17
Retrospective cohort st	IN	OR	31060580	CH	RL	2	1	1	1	1	2	1	1	2	2	1	2	2	1	20
Revealed by degrees: I	IN	OR	30589946	CH	RL	2	1	0	1	1	2	1	0	2	2	1	2	2	1	18
Risk of mortality asso	IN	OR	31296365	CH	DB	2	0	1	1	1	2	1	0	0	2	1	2	2	1	16
Role of coronary angio	IN	OR	31237435	CH	DB	4	0	1	0	1	2	1	1	2	2	1	2	0	1	18
Selective retrograde ce	IN	OR	31950100	CH	DC	3	1	1	1	0	0	0	1	2	2	0	2	0	1	14
Short and long-term su	IN	OR	31470101	CH	RL	2	1	1	1	1	2	0	0	0	2	1	2	2	1	16
Short and long-term su	IN	OR	31470101	CH	CH	2	1	1	1	1	2	0	0	2	2	1	0	0	1	14
Shorter defibrillation i	IN	OR	31442471	CH	RL	3	1	1	1	1	0	1	1	2	2	1	2	2	1	19
Standardising commun	IN	OR	31891790	CH	DB	3	0	1	1	1	2	1	1	2	2	1	2	0	1	18
Study on the timing o	IN	OR	30612965	KD	CH	2	1	1	1	1	2	1	1	0	0	1	0	0	1	12
Subphenotypes of Card	IN	OR	31541172	CH	CH	1	1	1	1	1	2	1	1	2	2	1	0	0	1	15
Subphenotypes of Card	IN	OR	31541172	CH	RL	2	1	1	1	0	2	1	1	2	2	1	0	0	1	15
Surface Body Tempera	IN	OR	30566033	CH	RL	2	1	0	1	1	2	1	1	2	2	1	2	2	1	19
Survival and neurologi	IN	OR	30572065	CH	DC	2	1	1	1	1	2	1	1	0	2	1	2	0	1	16
Survival to hospital di	IN	OR	30708074	KD	CH	2	1	1	1	1	2	1	1	2	2	1	2	0	1	18
Target Temperature Ma	IN	OR	30270729	CH	KD	2	1	1	1	1	2	1	1	2	2	1	2	0	1	18
Targeted hypothermia	IN	OR	31473324	CH	DB	4	0	0	0	0	0	0	1	2	2	1	0	0	0	10

Targeted Temperature	IN	OR	31809279	CH	GS	2	0	1	1	1	2	0	1	2	2	1	2	0	0	15
Targeted Temperature	IN	OR	31577396	CH	GS	3	1	1	1	1	2	1	1	2	2	1	2	2	1	21
Targeted Temperature	IN	OR	31314693	KD	CH	2	1	1	1	1	2	1	1	2	2	1	2	0	1	18
Targeted temperature n	IN	OR	31745738	CH	KD	2	1	1	1	1	2	1	1	0	2	1	2	0	1	16
Targeted temperature n	IN	OR	31146629	CH	DB	1	0	0	1	1	0	1	1	2	2	1	0	0	1	11
Targeted Temperature	IN	OR	31390319	CH	GS	2	1	1	1	1	2	1	1	2	2	1	2	2	1	20
Temporal trends in the	IN	OR	31796127	CH	RL	2	1	1	1	1	2	1	1	2	2	1	2	2	1	20
Temporal trends of sur	IN	OR	30790415	CH	RL	2	1	1	1	1	0	0	1	2	2	1	2	2	1	17
The acute respiratory d	IN	OR	30654012	CH	GS	2	1	1	1	1	2	0	1	2	2	1	2	0	1	17
The advocacy role of n	IN	OR	31113269	CH	GS	1	1	1	1	1	2	1	1	2	0	1	0	0	0	12
The balance of thromb	IN	OR	31682901	CH	CH	2	1	1	1	1	2	1	1	2	2	1	0	0	1	16
The balance of thromb	IN	OR	31682901	CH	DC	2	1	1	1	1	2	1	1	2	2	1	0	0	1	16
The Burden of Brain	IN	OR	30889022	CH	DB	2	0	1	1	1	2	1	1	2	2	1	2	0	1	17
The cardiac arrest survi	IN	OR	31539610	CH	DC	2	1	1	1	1	2	0	1	2	2	1	2	0	1	17
The Early Initiation of	IN	OR	30713299	CH	DC	2	1	1	1	1	2	0	1	2	2	1	2	2	1	19
The effect of extracorp	IN	OR	31146644	CH	RL	2	1	1	1	1	2	1	1	2	2	1	2	2	1	20
The effects of thoracic	IN	OR	31238036	CH	SC	2	1	1	1	1	2	1	1	0	0	1	2	0	1	14
The Evolving Role of	IN	OR	30760026	KD	CH	1	1	1	1	1	2	1	1	2	2	1	2	2	1	19
The impact of double	IN	OR	31059670	KD	CH	2	1	1	1	1	2	1	1	2	2	1	2	2	1	20
The Impact of Emerge	IN	OR	31934452	CH	KD	2	1	1	1	1	2	1	1	2	2	1	2	2	1	20
The impact of nurse st	IN	OR	31752859	CH	RL	2	1	1	1	1	2	1	1	2	2	1	2	2	0	19
The impact of proactiv	IN	OR	30409047	CH	DC	2	1	1	1	1	0	0	1	2	2	1	2	2	1	17
The impact of real-tim	IN	OR	31446232	KD	CH	2	1	1	1	1	2	1	1	2	2	1	2	2	1	20
The National Early Wa	IN	OR	30516860	CH	DB	2	1	1	1	1	2	1	1	2	2	1	2	0	1	18
The performance of a r	IN	OR	31377393	CH	DC	2	1	1	1	1	2	1	1	2	2	1	2	2	1	20
The prevalence of psyc	IN	OR	30735740	CH	GS	2	1	1	1	1	2	1	1	2	2	1	2	2	1	20
The related factors for	IN	OR	30702602	CH	DB	2	0	1	1	1	2	0	1	2	2	1	0	0	0	13
The urine biomarkers	IN	OR	31216431	CH	RL	2	1	1	1	1	2	1	1	2	2	1	2	2	1	20
The Use of ECMO for	IN	OR	29303029	CH	KD	2	1	1	1	0	0	0	1	0	2	1	2	0	1	12
Therapeutic Hypotherm	IN	OR	30052584	CH	RL	2	1	1	1	0	2	1	1	0	2	1	0	0	1	13
Therapeutic hypotherm	IN	OR	31950096	CH	KD	2	1	1	1	0	0	0	1	2	2	1	2	0	1	14
Therapeutic hypotherm	IN	OR	31950096	CH	CH	2	1	1	1	0	0	0	1	2	2	1	2	0	1	14
Therapeutic hypotherm	IN	OR	31223012	CH	GS	2	1	1	1	0	2	1	1	2	2	1	2	0	0	16
Transesophageal Echo	IN	OR	30773413	CH	DC	2	1	1	1	1	2	0	1	2	2	1	2	0	1	17
Transesophageal echoc	IN	OR	30862393	CH	GS	1	0	0	0	0	0	0	0	0	2	1	0	2	1	7
Trends Over Time in I	IN	OR	30407950	CH	DC	2	1	1	1	1	2	0	1	2	2	1	0	0	1	15
Use of automated exte	IN	OR	29116359	CH	DB	2	1	1	1	1	2	0	1	2	2	1	0	0	1	15
Usefulness of Intra-aor	IN	OR	30577950	CH	GS	2	1	1	1	1	2	0	1	2	2	1	0	0	1	15
Usefulness of Therape	IN	OR	31371900	CH	RL	2	1	1	1	1	0	0	1	0	2	1	2	2	1	15
Validating the Electro	IN	OR	31082902	CH	RL	2	1	1	1	1	2	0	1	2	2	1	2	2	1	19
Validation of Early Wa	IN	OR	31567342	CH	KD	2	1	1	1	1	2	1	1	2	0	0	2	2	1	17
Variation in outcomes	IN	OR	30590071	CH	DC	2	1	1	1	1	2	0	1	2	2	1	2	2	1	19
Venous Pooling in Su	IN	OR	31843349	CH	RL	3	1	0	0	1	0	0	0	0	0	0	0	0	1	6
Venovenous Extracorp	IN	OR	31182376	CH	GS	2	1	1	1	1	2	1	1	0	2	1	0	0	1	14
Ventilator settings and	IN	OR	31719587	CH	DC	2	1	1	1	1	0	0	1	0	0	0	0	0	1	8
Vitamin K deficiency	IN	OR	30415179	CH	DB	2	0	1	1	1	2	0	1	2	2	1	0	0	1	14
Vitamin K deficiency	IN	OR	30415179	CH	CH	2	1	1	1	1	2	0	1	2	2	1	0	0	1	15
Yellow Means Caution	IN	OR	30312207	CH	GS	2	1	1	1	1	2	0	1	0	2	0	0	0	1	12
Patient and hospital fa	IN	OR	30885828	CH	RL	2	1	1	1	1	2	0	1	2	2	1	0	0	1	15
A descriptive analysis	PED	OR	31136809	LA	MT	2	1	1	1	1	2	0	1	2	2	1	2	2	1	19
A mobile device app t	PED	OR	30797722	LA	MT	4	1	1	1	1	2	0	1	2	2	1	2	2	1	21
A Pulse Check on Lea	PED	OR	31842200	LA	ME	2	1	1	1	1	2	1	1	0	0	1	2	2	1	16
Arterial blood pressure	PED	OR	31246109	LA	MT	2	1	1	1	1	2	1	1	2	2	1	2	2	1	20

Association of diastoli	PED	OR	31390531	LA	LA	3	1	1	1	1	2	1	1	2	2	1	0	0	1	17
Association of diastoli	PED	OR	31390531	LA	EG	2	1	1	1	1	2	1	1	2	2	1	0	0	1	16
Association of school	PED	OR	31565076	LA	SRK	2	1	1	1	1	2	1	1	0	2	1	0	0	1	14
Avalidation study of tl	PED	OR	31155643	LA	MT	2	1	1	1	1	2	0	1	0	2	1	2	0	1	15
Cardiac Arrest in the F	PED	OR	30785870	LA	ME	2	1	1	1	1	2	1	1	2	2	1	0	0	1	16
Cardiac Arrest Outcom	PED	OR	30807545	LA	ME	2	1	1	1	1	2	1	1	0	2	1	0	0	1	14
Cardiopulmonary Resu	PED	OR	31232852	LA	EG	2	1	1	1	1	2	1	1	2	0	1	0	0	1	14
Catecholaminergic Pol	PED	OR	30640888	LA	ME	1	0	0	0	0	2	1	1	2	2	1	2	2	1	15
Cerebral microcirculatc	PED	OR	29192562	LA	ME	3	1	1	1	1	2	1	1	0	2	1	0	0	1	15
Changing Risk of In-H	PED	OR	30591395	LA	MT	2	1	1	1	1	2	0	1	2	2	1	0	0	1	15
Characteristics and out	PED	OR	31785372	LA	MN	2	1	1	0	0	2	1	1	0	0	1	0	0	1	10
Clinical Survey and Pr	PED	OR	31065052	LA	ME	2	1	1	1	1	2	1	1	2	2	1	0	0	1	16
Combination of Early	PED	OR	31214555	LA	SK	2	1	1	1	1	2	1	1	0	0	1	2	0	1	14
Comparison between r	PED	OR	30854750	LA	EG	3	1	1	1	1	2	1	1	0	2	1	2	0	1	17
Comparison between s	PED	OR	31318890	LA	MB	4	1	0	1	1	2	1	1	0	2	1	0	0	1	15
Comparison of In-Hos	PED	OR	31703806	LA	MB	2	1	1	1	1	0	0	1	0	2	1	0	2	1	13
Contemporary Outcom	PED	OR	30542921	LA	SRK	2	1	1	1	1	2	1	1	0	0	0	2	0	1	13
Creation of an empiric	PED	OR	30342418	LA	EG	2	0	1	1	1	2	1	1	2	2	1	0	2	0	16
Decline in incidence of	PED	OR	30809645	LA	MB	2	1	1	1	1	0	1	0	2	2	1	0	0	1	13
Decline in incidence of	PED	OR	30809645	LA	LA	2	1	1	1	1	0	0	0	2	2	1	0	0	1	12
Defining benefit thresh	PED	OR	31888705	LA	ME	2	1	1	1	1	2	1	1	2	0	0	0	0	1	13
Development and Eval	PED	OR	31218374	LA	MB	4	1	1	1	1	2	1	0	0	2	1	2	2	1	19
Development of a Nov	PED	OR	31411652	LA	MT	2	1	1	1	1	2	1	1	0	2	1	2	2	1	18
Development of a Nov	PED	OR	31411652	LA	LA	2	1	1	1	1	2	1	1	2	2	1	2	0	1	18
Developmental Outcor	PED	OR	30850900	LA	MT	2	1	1	1	1	2	1	1	2	2	1	2	2	1	20
Dispatcher-assisted car	PED	OR	30999084	LA	MB	2	1	0	1	1	2	1	0	0	2	1	0	2	1	14
Dispatcher-assisted car	PED	OR	30999084	LA	MN	2	1	1	1	1	2	1	0	2	2	1	0	0	1	15
Dispatcher-Assisted Ca	PED	OR	29200138	LA	MB	2	1	1	1	1	2	0	0	0	2	1	0	0	1	12
Early and long-term ou	PED	OR	31608760	LA	SRK	2	1	1	1	1	0	0	1	2	2	0	2	0	1	14
Early EEG Features fo	PED	OR	31033654	LA	LA	2	1	1	1	1	2	1	1	2	2	1	2	2	1	20
Early EEG Features fo	PED	OR	31033654	LA	MT	2	1	1	1	1	2	1	0	2	2	1	2	2	1	19
Early Epinephrine Imp	PED	OR	31245334	LA	MN	2	1	1	0	1	2	1	1	0	2	1	0	0	1	13
Early identification of	PED	OR	31496467	LA	EG	2	1	1	1	1	2	1	1	0	2	1	0	0	1	14
Early On-Scene Manag	PED	OR	30412719	LA	ME	2	1	1	1	1	2	1	1	2	2	1	2	2	1	20
Effect of metronome g	PED	OR	30850868	LA	MT	4	1	1	1	1	2	1	1	2	2	1	2	2	1	22
Effectiveness of a Higl	PED	OR	27763955	LA	MB	3	1	1	1	1	2	1	1	0	2	1	2	2	1	19
Effectiveness of a Higl	PED	OR	27763955	LA	LA	3	1	1	1	1	2	1	1	2	2	1	0	0	1	17
Electroencephalographi	PED	OR	31560988	LA	SRK	2	1	1	1	1	2	1	1	0	2	1	2	2	1	18
Epidemiologic Trends	PED	OR	31246741	LA	ME	2	1	1	0	1	2	1	1	2	2	1	2	2	1	19
Epidemiology and aeti	PED	OR	30262513	LA	EG	2	1	1	1	1	2	1	1	0	2	1	0	0	1	14
Epidemiology of Brair	PED	OR	30882855	LA	MN	2	1	1	1	1	2	1	1	0	0	1	0	0	0	11
Epidemiology of Sudc	PED	OR	31013114	LA	SC	1	1	1	1	1	2	1	1	2	0	1	0	0	1	13
Exercise testing oversi	PED	OR	30763784	LA	MN	2	1	1	1	1	2	1	1	0	2	1	2	2	1	18
Extracorporeal Cardiop	PED	OR	31496406	LA	MT	2	1	1	1	1	2	1	1	2	2	1	2	2	1	20
Extracorporeal cardiop	PED	OR	30515882	LA	MN	2	1	1	1	1	2	1	1	0	2	1	2	2	1	18
Extracorporeal cardiop	PED	OR	30515882	LA	LA	2	1	1	1	1	0	0	1	0	2	1	0	0	1	11
Five-year survival, per	PED	OR	31296978	LA	SK	2	1	1	1	1	2	0	1	0	2	1	2	0	1	15
Functional outcomes a	PED	OR	31404636	LA	MB	2	1	1	1	1	2	0	1	0	2	1	0	0	1	13
Hemodynamic effects	PED	OR	30946924	LA	LA	2	1	1	1	1	2	1	1	2	2	1	2	0	1	18
Hemodynamic effects	PED	OR	30946924	LA	ME	2	1	1	1	1	2	1	1	2	2	1	0	0	1	16
Impact of an Extraglot	PED	OR	31738725	LA	MB	2	1	1	1	1	2	1	1	0	2	1	2	0	1	16
Improved outcomes in	PED	OR	31623516	LA	SRK	2	1	1	1	1	2	1	1	0	2	0	2	0	1	15
In-Hospital Cardiac Ar	PED	OR	31545574	LA	MN	2	1	1	1	1	2	1	1	2	2	1	0	0	1	16

Inadequate oxygen deli	PED	OR	31325555	LA	SK	2	1	1	1	1	2	1	1	0	2	1	2	2	1	18
Incidence of peri-opera	PED	OR	30048261	LA	EG	2	0	1	1	1	2	1	1	2	0	1	0	0	1	13
Influence of Cardiopul	PED	OR	30951004	LA	ME	3	1	1	1	1	2	1	1	2	0	1	0	2	1	17
Is Supplemental Oxyg	PED	OR	31824899	LA	ME	3	1	1	1	1	0	1	1	2	2	1	0	0	1	15
Knocking-fingers' ches	PED	OR	29384754	LA	MT	2	1	1	1	1	2	1	1	2	2	1	2	0	1	18
Location of arrest and	PED	OR	31075289	LA	MN	2	1	1	1	1	2	1	1	0	2	1	0	0	1	14
Machine learning base	PED	OR	30264218	LA	MN	2	1	1	1	1	2	1	1	0	2	1	0	0	1	14
Medic One Pediatric (I	PED	OR	31523730	LA	MB	2	1	1	1	1	2	0	1	0	2	1	2	0	1	15
Methods Used to Max	PED	OR	31464818	LA	EG	2	1	1	1	1	2	1	1	2	0	1	2	2	1	18
Multimodal assessmer	PED	OR	30735742	LA	MN	2	1	1	0	1	2	1	1	0	2	1	2	2	1	17
One-year cognitive anc	PED	OR	30818016	LA	LA	3	1	1	1	1	2	1	1	2	2	1	0	0	1	17
One-year cognitive anc	PED	OR	30818016	LA	ME	2	1	1	1	1	2	1	1	0	2	1	0	2	1	16
Outcomes After Extrac	PED	OR	30747771	LA	MB	2	1	1	0	1	2	1	0	0	2	1	0	2	1	14
Outcomes of in-hospit	PED	OR	30714507	LA	SK	2	1	1	1	1	0	0	1	0	0	1	0	0	1	9
Oxygenation and Hem	PED	OR	30987091	LA	MN	3	0	1	1	1	2	1	1	0	2	1	0	0	1	14
Pauses in compression	PED	OR	31421191	LA	MN	2	1	1	1	1	2	1	1	0	2	1	2	0	1	16
Pediatric cardiac arrest	PED	OR	31489021	LA	EG	2	1	1	1	1	2	0	1	2	2	1	0	0	1	15
Pediatric in-hospital C	PED	OR	31734222	LA	ME	2	1	1	1	1	2	1	1	2	2	1	0	0	1	16
Pediatric Out-of-Hospi	PED	OR	30572071	LA	MT	4	1	1	1	1	2	1	1	2	2	1	2	2	1	22
Pediatric Out-of-Hospi	PED	OR	31327568	LA	LA	2	1	1	1	1	2	1	1	2	2	1	2	0	1	18
Pediatric Out-of-Hospi	PED	OR	31327568	LA	MN	2	1	1	1	1	2	1	1	0	2	1	0	0	1	14
Pediatric Out-of-Hospi	PED	OR	31327568	LA	MN	2	1	1	1	1	2	1	1	0	2	1	0	0	1	14
Practice Patterns after t	PED	OR	31093458	LA	EG	1	1	1	1	1	2	0	1	0	0	1	0	0	1	10
Prevalence and Outcon	PED	OR	31688674	LA	SRK	2	1	1	1	1	2	1	1	0	2	1	2	2	1	18
Pulseless electrical acti	PED	OR	29895572	LA	EG	4	1	1	1	0	2	1	1	0	2	1	0	0	1	15
Pulselessness After Ini	PED	OR	31006260	LA	LA	2	1	1	1	1	2	1	1	2	2	1	0	0	1	16
Pulselessness After Ini	PED	OR	31006260	LA	MN	2	1	1	1	1	2	1	1	0	2	1	0	0	1	14
Quantitative EEG pred	PED	OR	30971485	LA	EG	2	1	1	1	1	2	1	1	2	2	1	0	2	1	18
Race/Ethnicity and Ne	PED	OR	31288613	LA	SK	2	1	1	1	1	2	1	1	2	2	1	0	0	1	16
Readmissions to the I(	PED	OR	30867193	LA	MB	2	1	1	1	1	2	1	0	0	2	1	0	2	1	15
Relationship between i	PED	OR	30802557	LA	MT	2	1	1	1	1	2	1	1	2	2	1	0	0	1	16
Relationship Between	PED	OR	30985609	LA	SK	2	1	1	1	1	2	1	1	2	2	1	2	2	1	20
Relationships between	PED	OR	30922934	LA	EG	2	1	1	1	1	2	1	1	2	2	1	0	0	1	16
Rhythm characteristics	PED	OR	30639791	LA	MB	2	1	1	1	1	2	0	1	0	2	1	0	0	1	13
Sex Differences in Rec	PED	OR	30587069	LA	LA	2	1	1	1	1	2	1	1	2	2	1	0	0	1	16
Sex Differences in Rec	PED	OR	30587069	LA	ME	2	1	1	1	1	2	1	1	0	2	1	0	0	1	14
Simulation for Clinica	PED	OR	30608314	LA	MT	2	1	1	1	1	2	0	0	2	2	1	2	2	1	18
Smartwatch feedback c	PED	OR	29369843	LA	MT	4	1	1	1	1	2	1	1	2	2	1	2	0	1	20
Sports activity and pa	PED	OR	30953710	LA	MN	2	1	1	1	1	2	1	1	0	2	1	0	0	1	14
Sports activity and pa	PED	OR	30953710	LA	SK	2	1	1	1	1	2	1	1	0	0	1	0	0	1	12
Survival and Cardiopu	PED	OR	31453988	LA	ME	2	1	1	1	1	2	1	1	2	2	1	2	2	1	20
The aquaporin-4 inhibi	PED	OR	30367162	LA	LA	3	1	1	1	1	2	1	1	0	2	1	0	0	1	15
The aquaporin-4 inhibi	PED	OR	30367162	LA	SRK	1	1	1	1	1	2	1	1	0	2	1	0	0	1	13
The association of earl	PED	OR	31175965	LA	ME	2	1	1	1	1	2	1	1	2	2	1	0	2	1	18
The Association of Ho	PED	OR	30672841	LA	EG	2	1	1	1	1	2	1	1	2	2	1	0	2	1	18
The association of imr	PED	OR	31176666	LA	EG	2	1	1	1	1	2	1	1	2	2	1	0	2	1	18
The impact of a fellow	PED	OR	31331310	LA	MT	1	1	1	1	1	0	0	1	2	2	1	2	0	1	14
The Prognostic Value	PED	OR	31688714	LA	EG	2	1	1	1	1	2	1	0	2	2	1	0	0	1	15
The prognostic value c	PED	OR	30576784	LA	ME	2	1	1	1	1	2	1	1	0	2	1	2	2	1	18
The prognostic value c	PED	OR	30576784	LA	LA	2	1	1	1	1	2	0	1	2	2	1	2	0	1	17
The Prognostic Value	PED	OR	31702709	LA	EG	2	1	1	1	1	2	0	1	2	2	1	2	2	1	19
Time to epinephrine tr	PED	OR	30917838	LA	MT	2	1	1	1	1	2	0	1	2	2	1	2	2	1	19
Trends in Survival Aft	PED	OR	31542952	LA	MT	2	1	1	1	1	2	1	1	2	2	1	2	2	1	20

Trends in Survival Aft	PED	OR	31542952	LA	LA	2	1	1	1	1	2	1	1	2	2	1	0	0	1	16
Two-thumb-encircling	PED	OR	30007942	LA	MN	3	1	1	1	1	2	1	1	0	0	1	0	0	0	12
Use of Sodium Bicarb	PED	OR	31888123	LA	LA	2	1	1	1	1	2	1	1	2	2	1	2	2	1	20
Use of Sodium Bicarb	PED	OR	31888123	LA	MB	2	1	1	1	1	0	1	1	0	2	1	2	2	1	16
Vancomycin Prescribir	PED	OR	30864056	LA	SK	2	1	1	1	1	2	1	1	2	2	1	2	0	1	18
Ventilation Rates and	PED	OR	31369424	LA	SRK	2	1	1	1	1	2	1	1	2	2	1	2	2	1	20
Ventilation Rates and	PED	OR	31369424	LA	LA	2	1	1	1	1	2	1	1	2	2	1	2	2	1	20
Videographic Assessm	PED	OR	30118642	LA	EG	2	1	1	1	1	2	0	1	2	2	0	0	2	1	16
Wearable cardioverter-(	PED	OR	31476408	LA	ME	2	1	1	1	0	0	1	0	0	2	1	2	2	1	14
Wearable cardioverter-(	PED	OR	31476408	LA	LA	3	1	1	1	1	0	0	0	0	2	1	0	0	1	11
What is the best positi	PED	OR	30389590	LA	MT	2	1	1	1	1	2	1	1	2	2	1	2	2	1	20
What is the best positi	PED	OR	30389590	LA	LA	2	1	1	1	1	2	1	1	2	2	1	0	2	1	18
Association of Duratio	PED	OR	31568263	LA	MB	2	1	1	1	1	2	1	1	0	2	1	0	0	1	14
Efficacy and safety of 1	PED	OR	30255451	LA	MB	2	1	1	1	1	2	1	1	0	2	1	0	0	1	14
Prehospital advanced a	PED	OR	31539609	LA	MB	2	1	1	1	1	2	1	1	0	2	1	0	0	1	14
AMethod toDetect Pre	PRE	OR	31144648	DC	KY	3	1	1	1	1	2	1	1	2	2	1	2	2	1	21
A centralized system fi	PRE	OR	31323187	DC	DR	2	1	1	1	1	2	1	1	2	2	1	2	2	1	20
A History and Overvie	PRE	OR	31042339	DC	DR	2	1	1	1	1	0	1	1	2	2	1	2	2	1	18
A Machine Learning S	PRE	OR	30387719	DC	KY	2	1	1	1	1	0	0	0	2	2	1	0	0	1	12
A multistage algorithn	PRE	OR	29993407	DC	TD	3	1	1	1	1	0	1	0	2	2	1	0	0	1	14
A National Survey on	PRE	OR	30395025	DC	DR	2	1	1	1	1	2	1	1	0	2	1	2	2	1	18
A robust machine learn	PRE	OR	31946270	DC	TD	3	1	1	1	0	0	1	0	2	2	1	0	0	1	13
A Robust Machine Lea	PRE	OR	31946270	KH	TD/KH	1	1	1	1	1	0	1	0	2	2	1	0	0	1	12
A simple decision rule	PRE	OR	31228547	DC	CU	2	1	1	1	1	0	0	1	2	2	1	2	2	1	17
A simple decision rule	PRE	OR	31228547	DC	DC	2	1	1	1	1	2	1	1	2	2	1	2	2	1	20
Accuracy of nature of (	PRE	OR	30728188	DC	TD	2	1	1	1	1	0	1	1	0	2	1	0	0	1	12
Accuracy of ultrasounc	PRE	OR	30890289	DC	CU	4	1	1	1	1	2	1	1	2	0	0	2	2	1	19
Activation of extracorp	PRE	OR	31618345	DC	KY	2	1	1	1	1	2	1	1	2	2	1	2	2	1	20
Advanced airway mana	PRE	OR	31756361	DC	TD	2	1	1	1	1	2	1	1	2	2	1	2	0	0	17
Ambulance Density an	PRE	OR	30586753	DC	DR	2	1	1	1	1	2	1	1	2	2	1	2	2	1	20
An Analysis Using Mc	PRE	OR	31198281	DC	RC	2	1	1	1	1	2	1	1	2	2	1	2	2	1	20
An observational study	PRE	OR	31266839	DC	KY	2	1	1	1	1	2	0	1	2	2	1	2	2	1	19
Analyzing the Effectiv	PRE	OR	30709607	DC	DR	3	1	1	1	1	0	1	0	2	2	1	2	2	1	18
Application of a dual-(	PRE	OR	31456379	DC	CU	2	1	1	1	1	2	1	0	2	2	1	2	2	1	19
Application of mechan	PRE	OR	30601816	DC	RC	2	1	1	1	1	2	1	1	2	2	1	2	2	1	20
Assessment of Rapid I	PRE	OR	31355875	DC	DR	2	1	1	1	1	2	1	1	2	2	1	2	2	1	20
Associationofbystande	PRE	OR	31202824	DC	KY	2	1	1	1	1	2	1	1	2	2	1	2	2	1	20
Association between ai	PRE	OR	30795948	DC	TD	2	1	1	1	1	2	0	1	2	2	1	0	0	0	14
Association between l(	PRE	OR	30825552	DC	CU	2	1	1	1	1	0	0	1	0	2	1	2	0	1	13
Association Between N	PRE	OR	31484867	DC	KY	2	1	1	1	1	2	1	1	2	2	1	2	2	1	20
Association between p	PRE	OR	31304028	DC	TD	2	1	1	1	1	2	1	1	2	2	1	2	0	1	18
Association between p	PRE	OR	31645507	DC	TD	2	1	1	1	1	2	1	0	2	2	1	2	2	1	19
Association between sl	PRE	OR	31377392	DC	DR	2	1	1	1	1	2	1	1	2	2	1	2	2	1	20
Association of chest cc	PRE	OR	31369793	DC	TD	2	1	1	1	1	2	1	1	2	2	1	2	0	1	18
Association of Prehosp	PRE	OR	31289342	DC	DR	2	1	1	1	1	2	1	1	2	2	1	2	2	1	20
Association of the tim	PRE	OR	29804789	DC	RC	2	1	1	1	1	2	1	1	2	2	1	2	2	1	20
Association of ventilat	PRE	OR	31112744	DC	KY	2	1	1	1	1	2	0	1	2	2	1	2	2	1	19
Automated external de	PRE	OR	30682401	DC	DR	2	1	1	1	1	2	1	1	2	2	1	2	2	1	20
Availability and Use o	PRE	OR	30572769	DC	TD	1	1	1	1	1	2	1	0	0	2	1	0	0	0	11
Avalanche victims in (	PRE	OR	31185258	DC	CU	2	1	1	1	1	0	0	1	0	2	1	2	2	1	15
Bayesian Analysis of t	PRE	OR	31272823	DC	RC	2	1	1	1	1	2	1	1	2	2	1	2	2	1	20
Benefits of adding a pl	PRE	OR	31772105	DC	KY	2	1	1	1	1	2	1	1	2	2	1	2	2	1	20
Bystander cardiopulmo	PRE	OR	31129226	DC	RC	2	1	1	1	1	2	1	1	2	2	1	2	2	1	20

C-MAC Video Laryng	PRE	OR	31146497	DC	KY	2	1	1	1	1	2	1	1	2	2	1	2	2	1	20
Can pulse check by th	PRE	OR	30782884	DC	CU	2	1	1	1	1	2	1	1	2	2	1	2	0	1	18
Capnography: A supp	PRE	OR	31005583	DC	TD	2	1	1	1	1	0	0	1	2	2	1	2	0	1	15
Cardiac arrest prior to	PRE	OR	31094743	DC	CU	2	1	1	1	0	2	0	0	2	2	1	2	2	1	17
Cardiopulmonary resu	PRE	OR	31483118	DC	DR	2	1	1	1	1	2	1	1	2	2	1	2	2	1	20
Characterising variatio	PRE	OR	30852537	DC	RC	1	1	1	1	1	2	1	1	2	2	1	0	0	1	15
Characteristics Associa	PRE	OR	30968817	DC	RC	1	1	1	1	1	2	1	1	2	2	1	2	2	1	19
Characteristics of aÂ n	PRE	OR	30560444	DC	DR	2	1	1	1	1	2	1	1	2	2	1	2	2	1	20
Chest compression rel	PRE	OR	31560989	DC	KY	2	1	1	1	1	2	1	1	2	2	1	2	2	1	20
Circumstances, outcon	PRE	OR	31351088	DC	CU	2	1	1	1	1	2	1	1	0	2	1	2	2	1	18
Circumstances, outcon	PRE	OR	31351088	DC	CU	2	1	1	1	1	2	0	1	0	0	1	0	0	1	11
Citizen bystander-pati	PRE	OR	31350237	DC	RC	2	1	1	1	1	2	1	1	2	2	1	2	2	1	20
Closed chest compress	PRE	OR	31077754	DC	DR	4	1	1	1	1	2	1	1	2	2	1	2	2	1	22
Closed chest compress	PRE	OR	31077754	DC	DC	4	1	1	1	1	2	1	1	0	2	1	2	0	1	18
Cluster randomised co	PRE	OR	30683057	DC	RC	3	1	1	1	1	2	1	1	2	2	1	2	0	1	19
Coles and woolworths	PRE	OR	31820576	DC	CU	2	1	0	0	0	0	0	1	0	1	1	2	2	1	11
Combination of cardia	PRE	OR	31792731	DC	TD	3	1	1	1	1	0	1	1	0	2	1	0	0	1	13
Community first respo	PRE	OR	31323120	DC	DR	4	1	1	1	1	2	1	0	2	2	1	2	2	1	21
Comparing bysterande	PRE	OR	31005588	DC	CU	4	1	1	1	1	2	0	1	0	2	1	0	0	1	15
Comparison of chest c	PRE	OR	31112576	DC	DR	4	1	1	1	1	2	1	1	2	2	1	2	2	1	22
Comparison of chest c	PRE	OR	31112576	DC	RC	2	1	1	1	1	2	1	0	2	2	1	2	2	1	19
Comparison of EMS p	PRE	OR	31042340	DC	TD	3	1	1	1	1	2	1	1	0	2	1	0	0	1	15
Comparison of extraco	PRE	OR	30691512	DC	TM	2	1	1	1	1	2	1	1	2	2	1	2	0	1	18
Comparison of extraco	PRE	OR	30691512	DC	RC	2	1	1	1	1	2	1	1	0	2	1	2	2	1	18
Comparison of Outcon	PRE	OR	30795712	DC	DR	2	1	1	1	1	2	1	0	2	2	1	2	2	1	19
Comparison of standa	PRE	OR	31771511	DC	RC	3	1	1	1	1	2	1	1	2	2	1	2	2	1	21
Comparison of the eff	PRE	OR	31843537	DC	ky	2	1	1	1	1	2	1	1	2	2	1	2	0	1	18
Comparison of two di	PRE	OR	30760297	DC	TD	2	1	1	1	1	2	1	1	2	2	1	2	0	0	17
Comparison of two di	PRE	OR	30760297	DC	DC	2	1	1	1	1	0	1	1	2	2	1	2	2	1	18
Comparison of video l	PRE	OR	30385385	DC	TD	2	1	1	1	1	2	1	1	2	2	1	2	2	1	20
Confirmed cardiac out	PRE	OR	31171627	DC	RC	2	1	1	1	1	2	1	1	2	2	1	2	2	1	20
Confirmed cardiac out	PRE	OR	31171627	DC	DR	2	1	1	1	1	2	1	1	2	2	1	2	2	1	20
Convolutional Recurre	PRE	OR	31946274	DC	RC	2	1	1	1	1	2	1	0	2	2	1	2	2	1	19
Correlation between e	PRE	OR	31665061	DC	CU	2	1	1	1	1	2	1	1	2	2	1	2	2	1	20
Decreasing time to firs	PRE	OR	31469063	DC	TD	2	1	1	1	1	2	1	1	2	2	1	2	2	1	20
Defibrillator charging l	PRE	OR	31332449	DC	KY	3	1	1	1	1	2	1	1	2	2	1	2	2	1	21
Derivation and interna	PRE	OR	31311608	DC	CU	2	1	1	1	0	2	1	1	2	0	0	0	0	1	12
Determination of the t	PRE	OR	31910501	DC	KY	2	1	1	1	1	2	1	1	2	2	1	2	0	1	18
Developing neural net	PRE	OR	30982559	DC	DR	2	1	1	1	1	2	1	1	2	2	1	2	2	1	20
Developing new ways	PRE	OR	31034193	DC	CU	3	1	1	1	1	2	1	1	2	2	1	2	2	1	21
Direct transport to PC	PRE	OR	31271727	DC	DR	2	1	1	1	1	2	1	1	2	2	1	2	2	1	20
Disparity in Receipt a	PRE	OR	31613657	DC	CU	2	1	1	1	1	0	0	1	2	2	1	2	2	1	17
Dispatcher assisted ca	PRE	OR	31689831	DC	TD	3	1	1	1	1	2	0	1	2	2	1	2	0	0	17
Dispatcher Identificati	PRE	OR	31779716	DC	DR	2	1	1	1	1	2	1	1	2	2	1	2	2	1	20
Dispatcher-Assisted B	PRE	OR	31392232	DC	KY	2	1	1	1	1	2	0	1	2	2	1	2	0	1	17
Dispatcher-assisted ca	PRE	OR	31675978	DC	RC	2	1	1	1	1	2	1	1	0	0	1	2	0	1	14
Dispatcher-assisted ca	PRE	OR	31756360	DC	KY	2	1	1	1	1	2	1	1	2	2	1	0	0	1	16
Does chest compressio	PRE	OR	30761856	DC	KY	2	1	1	1	1	2	1	1	2	2	1	2	2	1	20
Does endotracheal intu	PRE	OR	30753851	DC	TD	2	1	1	1	1	2	1	0	2	2	1	2	0	0	16
Does endotracheal intu	PRE	OR	30753851	DC	CU	2	1	1	1	1	2	1	0	2	2	1	2	0	1	17
Drone delivery of an a	PRE	OR	30961651	DC	CU	2	1	1	1	1	2	1	1	2	2	1	0	0	1	16
Early advanced life su	PRE	OR	30576783	DC	KY	2	1	1	1	1	2	1	1	2	2	1	2	2	1	20
Early initiation of Ext	PRE	OR	30738245	DC	TM	4	1	0	0	0	2	1	0	2	2	1	0	0	0	13

Early initiation of extr	PRE	OR	30738245	DC	RC	3	1	1	1	0	2	1	1	0	0	0	0	0	1	11
ECG pre-hospital telet	PRE	OR	31441838	DC	CU	2	1	1	1	1	2	0	0	0	0	0	0	0	1	9
ECG-based pulse detec	PRE	OR	30215212	DC	TD	3	1	1	1	0	2	0	0	2	2	1	0	0	1	14
Effectofendotrachealint	PRE	OR	31009692	DC	KY	2	1	1	1	1	2	0	1	2	2	1	2	2	1	19
Effect of a feedback sy	PRE	OR	31884870	DC	TD	3	1	1	1	1	2	1	1	2	2	1	2	2	1	21
Effect of awareness tin	PRE	OR	31870923	DC	DR	2	1	1	1	1	2	1	1	2	2	1	2	2	1	20
Effect of bystander CP	PRE	OR	31499101	DC	RC	2	1	1	1	1	2	1	1	2	2	1	2	2	1	20
Effect of endotracheal a	PRE	OR	31009692	DC	CU	3	1	1	1	1	2	1	1	0	2	1	2	2	1	19
Effect of Implementati	PRE	OR	31291129	DC	DR	1	1	1	1	1	2	1	1	2	2	1	2	2	1	19
Effect of initial airway	PRE	OR	30902690	DC	DR	2	1	1	1	1	2	1	1	2	2	1	2	2	1	20
Effect of Intravenous V	PRE	OR	31122576	DC	KY	2	1	1	1	1	2	1	0	2	2	1	2	2	1	19
Effect of Intravenous V	PRE	OR	31122576	DC	DR	2	1	1	1	1	2	1	1	2	2	1	2	2	1	20
Effect of new 9-1-1 sys	PRE	OR	31664875	DC	DR	2	1	1	1	1	2	1	1	2	2	1	2	2	1	20
Effect of team-based ca	PRE	OR	31550494	DC	TD	2	1	1	1	1	2	1	1	0	2	1	2	0	1	16
Effectiveness and safet	Pre	OR	30648550	DC	CU	2	1	1	1	1	2	1	1	2	0	1	2	0	1	16
Effectiveness of a com	PRE	OR	31669756	DC	DR	1	1	1	1	1	2	1	1	2	2	1	2	2	1	19
Effectiveness of a netw	PRE	OR	31088520	DC	KY	2	1	1	1	1	2	0	1	2	0	1	2	0	1	15
Effectiveness of dispat	PRE	OR	31542238	DC	RC	2	1	1	1	1	2	1	1	0	2	1	0	0	1	14
Effectiveness of intuba	PRE	OR	30572066	DC	CU	2	1	1	1	1	2	1	1	2	2	1	2	2	1	20
Effectiveness of Prehos	PRE	OR	30773983	DC	TD	2	1	1	1	1	2	1	1	2	2	1	2	2	1	20
Effectiveness of the du	PRE	OR	31348172	DC	CU	2	1	1	1	1	2	0	0	2	2	1	2	2	1	18
Effects of Chest Comp	PRE	OR	31938777	DC	KY	2	1	1	1	0	2	0	1	2	2	1	2	2	1	18
Effects of dispatcher-in	PRE	OR	31499100	DC	TD	2	1	1	1	1	0	1	1	2	2	1	2	0	0	15
Effects of Shenfu injec	PRE	OR	31770574	DC	DR	4	1	1	1	1	2	1	1	2	2	1	2	0	1	20
Epinephrine administra	PRE	OR	29857945	DC	RC	2	1	1	1	1	2	1	1	2	2	1	2	0	1	18
Epinephrine during res	PRE	OR	31420058	DC	TD	2	1	1	1	1	2	1	1	2	2	1	2	0	1	18
Ethical dilemmas durin	PRE	OR	28558489	DC	CU	1	1	1	1	1	2	1	1	0	0	0	0	0	1	10
Experiences among fir	PRE	OR	31753873	DC	KY	1	1	1	1	1	2	1	1	2	2	1	2	2	1	19
Extracorporeal cardiopu	PRE	OR	31860604	DC	CU	2	1	1	1	1	2	1	1	2	2	1	2	2	1	20
Extracorporeal cardiopu	PRE	OR	31670793	DC	TD	2	1	1	1	1	2	1	1	2	2	1	0	0	0	15
Extracorporeal Life Su	PRE	OR	30365965	DC	DR	2	1	1	1	1	2	1	1	2	2	1	2	2	1	20
Factors Associated wit	PRE	OR	30585772	DC	RC	1	1	1	1	1	2	1	1	0	2	1	0	0	1	13
FEASIBILITY OF A I	PRE	OR	31060845	DC	KY	2	1	1	1	1	2	1	1	2	2	1	2	2	1	20
Feasibility of a modifi	PRE	OR	31060845	DC	TD	3	1	1	1	1	2	1	0	0	2	1	2	2	1	18
Feasibility of a Modifi	PRE	OR	31060845	DC	DC	2	1	1	1	1	2	1	0	2	2	1	0	0	1	15
Feasibility of an augm	PRE	OR	31406943	DC	DR	1	1	1	1	1	2	1	1	2	2	1	2	2	1	19
Feasibility of Out-of-H	PRE	OR	30192687	DC	KY	2	1	1	1	1	2	1	1	2	2	1	2	2	1	20
Feasibility of Pre-Hosp	PRE	OR	31707942	DC	TD	2	1	1	1	1	2	1	1	2	2	1	0	0	1	16
Feasibility study for in	PRE	OR	31867879	DC	DR	2	1	1	1	1	2	1	1	0	2	1	2	2	1	18
Feasilbility and clinica	PRE	OR	31851119	DC	CU	2	1	1	1	1	2	1	1	2	2	1	2	2	1	20
Field termination-of-re	PRE	OR	30580892	DC	DR	2	1	1	1	1	2	1	1	2	2	1	2	2	1	20
First response treatmen	PRE	OR	31842928	DC	CU	1	1	1	1	1	2	1	1	0	2	1	0	0	1	13
Focused cardiac ultraso	PRE	OR	31279947	DC	KY	2	1	1	1	1	2	0	1	2	2	1	2	2	1	19
GPs who volunteer to	PRE	OR	31686571	DC	TD	1	1	0	0	0	2	1	1	0	0	1	0	0	0	7
Health care utilization	PRE	OR	31039392	DC	RC	2	1	1	1	1	2	1	1	2	2	1	0	0	1	16
Healthcare Provider Pe	PRE	OR	31232856	DC	TD	2	1	1	1	1	2	1	1	2	2	1	2	2	1	20
Helicopter Emergency	PRE	OR	31052200	DC	CU	2	1	1	1	1	2	1	1	2	2	1	2	0	1	18
How is quality of card	PRE	OR	31358079	DC	DR	1	1	1	1	1	2	1	1	2	2	1	2	2	1	19
Identification of return	PRE	OR	30498975	DC	RC	3	1	1	1	1	2	1	1	0	2	1	2	0	1	17
Impact of adrenaline de	PRE	OR	30991079	DC	TD	2	0	1	1	1	0	0	1	2	2	1	2	0	0	13
Impact of cardiopulmo	PRE	OR	31767584	DC	CU	3	1	1	1	1	2	1	1	0	2	1	2	2	1	19
Impact of cardiopulmo	PRE	OR	29855670	DC	KY	2	1	1	1	1	2	1	1	2	2	1	2	0	1	18
Impact of prehospital r	PRE	OR	30681557	DC	TD	2	1	1	1	1	2	1	1	2	2	1	2	0	1	18

Impact of prehospital r	PRE	OR	30681557	DC	CU	2	1	1	1	1	2	1	0	2	0	0	0	0	1	12
Impact of prehospital p	PRE	OR	30448503	DC	KY	2	1	1	1	1	2	1	1	2	2	1	2	2	1	20
Impact of synchronous	PRE	OR	31273676	DC	CU	2	1	1	1	1	2	1	1	0	2	1	2	2	1	18
Impact of type of emer	PRE	OR	31592321	DC	TD	2	1	1	1	1	2	1	1	0	2	1	2	0	1	16
Impact of type of emer	PRE	OR	31592321	KH	TD/KH	2	1	1	1	1	2	1	1	2	2	1	2	2	1	20
Impedance Based Auto	PRE	OR	31945835	DC	DR	2	1	1	1	1	2	1	1	2	2	1	2	2	1	20
Implementation of a te	PRE	OR	31687999	DC	TD	2	0	1	0	0	0	0	1	0	0	1	2	0	0	7
Implications of long-te	PRE	OR	30861053	DC	DR	2	1	1	1	1	2	1	1	2	2	1	2	2	1	20
Improving emergency	PRE	OR	31734221	DC	RC	2	1	1	1	1	2	1	1	2	2	1	2	2	1	20
Improving emergency	PRE	OR	31734221	KH	RC/KH	1	1	1	1	1	2	1	1	2	2	1	2	2	1	19
Improving rapid respo	PRE	OR	31144556	DC	KY	1	1	1	1	1	2	1	1	2	2	1	2	2	1	19
Improving rapid respo	PRE	OR	31144556	DC	DC	2	1	1	1	1	2	1	1	0	0	0	0	0	1	11
Influence of prehospita	PRE	OR	29752776	DC	DR	2	1	1	1	1	2	1	1	2	2	1	2	2	1	20
Initial end-tidal carbon	PRE	OR	31742569	KH	DR/KH	2	1	1	1	1	2	1	1	2	2	1	2	2	1	20
Initial end-tidal carbon	PRE	OR	31742569	DC	DR	2	1	1	1	1	2	1	1	2	2	1	2	2	1	20
Intra-Arrest induction	PRE	OR	30427769	DC	CU	2	1	1	1	1	2	1	1	2	2	1	2	2	1	20
Intra-Arrest Induction	PRE	OR	30427769	KH	CU/KH	2	1	1	1	1	2	1	1	2	2	1	2	2	1	20
Intraosseous versus int	PRE	OR	30391366	DC	KY	2	1	1	1	1	2	1	1	2	2	1	2	2	1	20
Intrathoracic Airway C	PRE	OR	30257100	DC	DR	2	1	1	1	1	2	1	1	2	2	1	2	2	1	20
Introduction of an Ext	PRE	OR	31886085	DC	RC	2	1	1	1	1	2	1	1	2	2	1	2	0	1	18
Kinetic analysis of car	PRE	OR	30974314	DC	RC	2	1	1	1	1	2	1	1	2	2	1	2	2	1	20
Kinetics of manual and	PRE	OR	31639462	DC	TD	2	1	1	1	1	2	1	1	0	2	1	2	0	0	15
Levels of Catecholami	PRE	OR	31257337	DC	RC	2	1	1	1	1	2	1	1	2	2	1	2	0	0	17
Load distributing band	PRE	OR	30929972	DC	TD	2	0	1	0	1	2	1	1	0	2	1	2	0	0	13
Location of arrest and	PRE	OR	31326952	DC	TD	2	1	1	1	1	2	1	1	2	2	1	2	0	1	18
Locational effect on au	PRE	OR	31378298	DC	TD	2	1	1	1	1	2	1	1	2	2	1	2	0	1	18
LUCAS versus manua	PRE	OR	30590977	DC	CU	3	1	1	1	1	2	1	1	2	2	1	2	2	1	21
Machine learning as a	PRE	OR	30664917	DC	RC	2	1	1	1	1	2	1	1	2	2	1	2	2	1	20
Mandated 30-minute S	PRE	OR	31472942	DC	DR	2	1	1	1	1	2	1	1	2	2	1	2	2	1	20
Mandated 30-minute S	PRE	OR	31472942	KH	DR/KH	2	1	1	1	1	0	0	0	2	2	0	2	2	1	15
Maximum value of en	PRE	OR	31613696	DC	CU	2	1	1	1	1	2	1	0	2	2	1	2	2	1	19
Medical dispatcher' pe	Pre	OR	30683139	DC	CU	2	1	1	1	1	2	1	1	2	2	1	2	2	1	20
Mobile phone-based al	PRE	OR	31887366	DC	RC	2	1	1	1	1	2	1	1	2	2	1	2	2	1	20
Motivation, challenges	PRE	OR	31399458	DC	CU	3	1	1	1	1	2	1	1	2	2	1	0	0	1	17
Multimedia-aided inst	PRE	OR	31246511	DC	DR	2	1	1	1	1	2	1	1	2	2	1	2	2	1	20
Near-infrared spectrosc	PRE	OR	30446504	DC	TD	2	1	1	1	1	0	0	1	2	2	1	2	0	1	15
North American valida	PRE	OR	30639788	DC	TD	4	1	1	1	1	2	1	1	2	2	1	2	2	1	22
One year experience wi	PRE	OR	31401135	DC	CU	2	1	1	1	1	2	0	1	0	2	1	2	2	1	17
One year experience wi	PRE	OR	31401135	DC	DR	2	1	1	1	1	2	1	1	2	2	1	2	2	1	20
Original articleImpact	PRE	OR	31439715	DC	KY	3	1	1	1	1	0	0	1	2	0	1	0	0	1	12
Out of hospital cardiac	PRE	OR	30597132	DC	CU	2	1	1	1	1	2	1	1	2	2	1	2	2	1	20
Out of hospital cardiac	PRE	OR	31857141	DC	CU	2	1	1	1	1	2	1	1	2	2	1	2	2	1	20
Out of hospital cardiac	PRE	OR	31692808	DC	RC	2	1	1	1	1	0	1	1	0	2	1	2	0	1	14
Out-of-hospital cardiac	PRE	OR	30119989	DC	TD	2	1	1	1	1	2	1	1	2	2	1	2	0	0	17
Outcome after out-of-h	PRE	OR	31401807	DC	KY	3	1	1	1	1	0	0	1	2	2	1	0	2	1	16
Outcomes for Hemodia	PRE	OR	30733235	DC	KY	2	1	1	1	1	2	1	0	2	2	1	2	0	1	17
Paramedic attitudes an	PRE	OR	31791962	DC	RC	1	1	1	1	1	2	1	1	2	2	1	2	2	1	19
Paramedic attitudes an	PRE	OR	31791962	KH	RC/KH	1	1	1	1	1	2	1	1	2	2	1	2	2	1	19
Paramedic equipment l	PRE	OR	31670157	DC	DR	4	1	1	1	1	2	1	1	2	2	1	2	2	1	22
Passivelegraisinginout	PRE	OR	30790693	DC	KY	2	1	1	1	1	2	1	1	2	2	1	2	0	1	18
Phenomenological stu	PRE	OR	31427472	DC	TD	1	1	1	1	1	2	1	1	2	2	1	2	0	1	17
Phenomenological s	PRE	OR	31427472	DC	DC	1	1	1	1	1	2	0	1	0	2	1	0	0	1	12
Pilot Study of the Effe	PRE	OR	31042341	DC	KY	1	1	1	1	1	0	0	0	2	2	1	2	2	1	15

Pre-hospital advanced	PRE	OR	30819685	DC	KY	2	1	1	1	1	2	1	1	2	2	1	2	2	1	20
Predictors of Out of H	PRE	OR	31555766	DC	CU	2	1	1	1	1	2	1	1	2	2	1	2	2	1	20
Prehospital advanced c	PRE	OR	30001817	DC	CU	2	1	1	1	1	2	1	1	2	2	1	2	2	1	20
Prehospital advanced c	PRE	OR	30001817	KH	CU/KH	2	1	1	1	1	2	0	1	2	2	1	2	2	1	19
Prehospital cardiopulm	PRE	OR	31443673	DC	KY	1	1	1	1	1	2	1	1	2	2	1	2	2	1	19
Prehospital cardiopulm	PRE	OR	30883667	DC	KY	2	1	1	1	1	2	1	1	2	2	1	2	2	1	20
Prehospital determinar	PRE	OR	31003991	DC	TD	2	1	1	1	1	2	1	1	2	2	1	0	0	0	15
Prehospital extracorpo	PRE	OR	31264379	DC	TD	2	1	1	1	1	2	1	1	0	0	1	0	0	1	12
Preliminary experience	PRE	OR	30605711	DC	RC	2	1	1	1	1	2	1	1	2	2	1	2	0	1	18
Prevalence and Predict	PRE	OR	31566990	DC	RC	1	1	1	1	1	2	1	1	2	2	1	0	0	1	15
Public access of autom	PRE	OR	31129230	DC	DR	2	1	1	1	1	2	1	1	2	2	1	2	2	1	20
Public Knowledge and	PRE	OR	30375018	DC	CU	2	1	1	1	1	2	1	1	2	2	1	2	2	1	20
Public-access defibrill	PRE	OR	31862250	DC	KY	2	1	1	1	1	2	1	1	2	2	1	2	2	1	20
Quality metrics for the	PRE	OR	31129229	DC	CU	2	1	1	1	0	0	0	1	0	0	0	2	0	0	8
Quality of physician ca	PRE	OR	31785983	DC	KY	2	1	1	1	1	2	1	1	2	0	1	2	2	1	18
Randomised controlled	PRE	OR	31015214	DC	KY	3	1	1	1	1	2	1	1	2	2	1	0	0	1	17
Randomised controlled	PRE	OR	31015214	KH	KY/KH	3	1	1	1	1	2	1	1	2	2	1	2	2	1	21
Real-life time and dist	PRE	OR	31141717	DC	TD	2	1	1	1	1	0	0	1	0	2	1	2	0	0	12
Real-time cardiopulmo	PRE	OR	31708314	DC	TD	2	0	1	1	1	2	1	1	2	0	1	2	0	0	14
Recording Out-of-Hos	PRE	OR	30130413	DC	DR	2	1	1	1	1	2	1	1	2	2	1	2	2	1	20
Regurgitation and pul	PRE	OR	30811470	DC	RC	3	1	1	1	1	2	1	1	0	2	1	2	0	1	17
Relationship between	PRE	OR	31179947	DC	DR	1	1	1	1	1	2	1	1	2	2	1	2	2	1	19
Repeated adrenaline do	PRE	OR	30708076	DC	RC	2	1	1	1	1	2	1	1	2	2	1	2	2	1	20
Rescue transesophagea	PRE	OR	31820822	DC	CU	1	1	0	1	0	0	0	1	0	2	1	2	2	1	12
RESUSCITATIVE EN	PRE	OR	30067564	DC	KY	3	1	1	1	1	2	1	1	2	2	1	2	2	1	21
Resuscitative endovasc	PRE	OR	31076474	DC	RC	2	1	1	1	1	2	1	1	2	2	1	2	2	1	20
Resuscitative Endovas	PRE	OR	30067564	KH	KY/KH	3	1	1	1	1	2	1	1	2	2	1	2	2	1	21
Safety and Effectivene	PRE	OR	30556765	DC	TD	2	1	1	1	1	2	1	1	2	2	1	2	2	1	20
Sample size estimation	PRE	OR	31049459	DC	DR	2	1	1	1	0	2	1	1	2	2	1	2	2	1	19
Should we resuscitate	PRE	OR	31641038	DC	KY	2	1	1	1	1	2	1	1	2	2	1	2	2	1	20
Sign of life is associat	PRE	OR	30737554	DC	TD	2	1	1	1	1	2	0	1	0	2	1	2	0	1	15
Significance of automa	PRE	OR	31297625	DC	DR	2	1	1	1	1	2	1	1	2	2	1	2	2	1	20
Simulating Public Bus	PRE	OR	31124734	DC	TD	2	1	1	1	1	0	1	1	0	2	1	2	0	1	14
Simulation training en	PRE	OR	30862530	DC	DR	2	1	1	1	1	2	1	1	2	2	1	2	2	1	20
Simulation training en	PRE	OR	30862530	DC	RC	2	1	1	1	1	2	1	1	2	2	1	2	0	1	18
Skeletal and soft tissu	PRE	OR	30649242	DC	TD	2	1	1	1	1	2	0	1	2	2	1	2	2	1	19
Survival in Out-of-Ho	PRE	OR	30929457	DC	KY	3	1	1	1	1	0	0	1	2	2	1	2	2	1	18
Survival to hospital di	PRE	OR	30708074	DC	TD	3	1	1	1	1	2	1	1	2	2	1	2	0	0	18
Survival to hospital di	PRE	OR	30708074	KH	TD/KH	2	1	1	1	1	2	1	1	2	2	1	2	2	1	20
Synchronized Chest C	PRE	OR	31697562	DC	RC	4	1	1	1	1	2	1	1	0	2	1	2	0	1	18
Textmessagealertsyster	PRE	OR	30902689	DC	KY	3	1	1	1	1	0	0	1	2	2	1	2	2	1	18
Theeffectofdispatcher-a	PRE	OR	30639789	DC	KY	2	1	1	1	1	2	0	1	2	2	1	2	2	1	19
Theeffectofintravenous	PRE	OR	30708075	DC	KY	3	1	1	1	1	2	0	1	2	2	1	2	2	1	20
The Additive Effect of	PRE	OR	30799340	DC	TD	2	1	1	1	1	2	1	1	2	2	1	2	0	1	18
The assessment of kin	pre	OR	30736850	DC	CU	2	1	1	1	0	2	1	1	2	0	1	2	2	1	17
The Association of the	PRE	OR	31248676	DC	KY	2	1	1	1	1	2	1	1	2	2	1	2	2	1	20
The Case for Drone-as	PRE	OR	31278178	DC	RC	1	1	1	1	1	2	1	1	2	2	1	2	2	1	19
The effect of automatic	PRE	OR	31225932	DC	CU	3	1	1	1	1	0	0	1	2	2	1	2	2	1	18
The effect of operator p	PRE	OR	31813404	DC	TD	3	1	1	1	1	2	1	1	2	2	1	2	2	1	21
The effect of prehospit	PRE	OR	31412291	DC	RC	2	1	1	1	1	2	1	1	2	2	1	2	0	1	18
The Effect of Transpor	PRE	OR	30863269	DC	KY	2	1	1	1	1	0	0	1	2	2	1	2	2	1	17
The effect of ventilatio	PRE	OR	30946921	DC	TD	2	0	1	1	1	2	1	1	2	2	1	0	0	1	15
THE EFFECTS OF T	PRE	OR	30052583	DC	KY	4	1	1	1	1	2	1	1	2	2	1	0	2	1	20

The impact of real-tim	PRE	OR	31446232	DC	DR	2	1	1	1	1	2	1	1	2	2	1	2	2	1	20
The impact of resuscita	PRE	OR	31330198	DC	RC	3	1	1	1	1	2	1	1	2	2	1	2	2	1	21
The incidence of airwa	PRE	OR	31767676	DC	CU	2	1	1	1	1	2	1	1	2	2	1	2	2	1	20
The Maximum Diamet	PRE	OR	31585685	DC	RC	2	1	1	1	1	2	1	1	2	2	1	0	0	1	16
The performance of a r	PRE	OR	31377393	DC	RC	2	1	1	1	1	2	1	1	2	2	1	2	2	1	20
The POCUS pulse che	PRE	OR	30902687	DC	TD	3	1	1	1	1	2	1	1	2	2	1	2	0	1	19
The presence of psycho	PRE	OR	31251894	DC	RC	1	1	1	1	1	2	1	1	2	2	1	0	0	1	15
The use of dispatcher a	PRE	OR	30876922	DC	DR	4	1	1	1	1	2	1	1	2	2	1	2	2	1	22
Time from arrest to ex	PRE	OR	31155852	DC	CU	2	1	1	1	1	2	1	1	2	2	1	2	2	1	20
Timing of advanced ai	PRE	OR	30912467	DC	TD	2	1	1	1	1	2	1	1	2	2	1	2	2	1	20
Towards a non-invasiv	PRE	OR	30365974	DC	TD	3	1	1	1	1	2	1	1	2	2	1	2	0	1	19
Traumatic cardiac arres	PRE	OR	30032348	DC	DR	2	1	1	1	1	2	1	1	2	2	1	2	2	1	20
Trends in survival fror	PRE	OR	31430512	DC	DR	2	1	1	1	1	2	1	1	2	2	1	2	2	1	20
Trends in the use of in	PRE	OR	31197835	DC	TD	2	1	1	1	1	2	1	1	0	0	1	0	0	1	12
Use of Mechanical Car	PRE	OR	31617923	KH	KY/KH	2	1	1	1	1	0	0	1	2	2	1	2	2	1	17
Use of Mechanical Car	PRE	OR	31617923	DC	KY	2	1	1	1	1	2	1	1	2	2	1	2	0	1	18
Use of motorlance to c	PRE	OR	31602431	DC	CU	2	1	1	1	1	2	1	1	0	2	1	2	2	1	18
Use of resuscitative ba	PRE	OR	31121206	DC	CU	3	1	1	1	1	2	0	1	0	2	1	0	2	1	16
Use of selected teleme	PRE	OR	30952832	DC	CU	2	1	1	1	1	2	1	1	2	2	1	2	2	1	20
Usefulness of a stool t	PRE	OR	31395031	DC	DR	4	1	1	1	1	2	1	1	0	0	0	2	0	0	14
Usefulness of cerebral	PRE	OR	31004721	DC	KY	2	1	1	1	1	2	1	1	2	2	1	2	2	1	20
Using a smartwatch wi	PRE	OR	31078650	DC	TD	3	1	1	1	1	2	1	1	0	2	1	2	0	1	17
Using an Immersive V	PRE	OR	30407959	DC	RC	1	1	1	1	1	2	0	1	2	2	1	2	2	1	18
Using an Immersive V	PRE	OR	30407959	KH	RC/KH	1	1	1	1	1	0	0	0	2	2	1	0	0	1	11
Utility of CPR machin	PRE	OR	31239492	DC	CU	3	1	1	1	0	2	1	1	0	2	1	2	0	1	16
Ventilation feedback d	PRE	OR	31640797	DC	CU	3	1	1	1	1	2	1	1	2	2	1	2	2	1	21
Ventilation feedback d	PRE	OR	31640797	KH	CU/KH	3	1	1	1	1	2	1	1	2	2	1	2	2	1	21
Vital sign patterns befo	PRE	OR	30926452	DC	SC	1	1	1	1	1	2	1	1	0	2	1	0	0	1	13
A feasibility study for	PRO	OR	30599180	CM	TK	2	1	1	1	1	2	1	1	0	2	1	0	0	1	14
A machine learning ba	PRO	OR	30885826	SW	CM	2	1	1	1	1	2	0	1	0	2	1	0	0	1	13
Angiographic character	PRO	OR	30280478	CM	JP	2	1	1	1	1	0	0	1	0	2	1	2	0	1	13
Aortic stenosis is an ir	PRO	OR	30818015	CM	SZ	2	1	1	1	0	2	0	1	0	2	1	0	0	0	11
Assessment of serum b	PRO	OR	30781939	CM	SZ	2	1	1	1	1	2	1	1	0	2	1	0	0	1	14
Association between A	PRO	OR	31540352	CM	SW	2	1	1	1	1	2	0	1	0	2	1	0	0	1	13
Association between ac	PRO	OR	31521016	CM	TK	2	1	1	1	1	2	1	1	0	2	1	2	0	0	15
Association between ac	PRO	OR	31521016	CM	CM	2	1	1	1	1	2	1	1	0	2	1	0	0	0	13
Association Between E	PRO	OR	30303836	CM	CC	2	1	1	1	1	2	0	1	2	2	1	2	2	1	19
Association Between E	PRO	OR	30303836	CM	CM	2	1	1	1	1	2	0	1	2	2	1	2	0	1	17
Association between ly	PRO	OR	30651995	CM	TK	2	1	1	1	1	2	0	1	0	2	1	2	0	1	15
Association between se	PRO	OR	31541979	CM	SZ	2	1	1	1	1	2	1	1	0	2	1	2	0	1	16
Associations between c	PRO	OR	31493983	CM	JP	2	1	1	1	1	2	1	1	0	2	1	2	0	1	16
Associations between i	PRO	OR	31786236	CM	SW	2	1	1	1	1	2	1	1	0	2	1	0	0	1	14
Associations between p	PRO	OR	30691510	CM	JP	3	1	1	1	1	2	1	1	2	2	1	0	0	1	17
Beyond dichotomy: pa	PRO	OR	31215475	CM	CC	2	1	1	1	1	2	1	1	0	2	1	2	0	1	16
Brain computed tomog	PRO	OR	31910500	CM	TK	2	1	1	1	1	2	1	1	0	2	1	2	0	0	15
Brain computed tomog	PRO	OR	31910500	CM	CM	2	1	1	1	0	2	1	1	0	2	1	2	0	0	14
Brain Diffusion Imagi	PRO	OR	31107566	CM	SZ	2	1	1	1	1	2	1	1	2	2	1	2	0	1	18
Brain Hypoxia Second	PRO	OR	31789834	CM	CC	2	1	1	1	1	2	0	1	0	2	1	0	0	1	13
Brain Hypoxia Second	PRO	OR	31789834	CM	CM	2	1	1	1	1	2	0	1	0	2	1	0	0	1	13
Cardiac Arrest in Patie	PRO	OR	31272121	SW	CM	2	1	1	1	1	0	0	1	0	0	0	2	0	0	9
Cardiac arrest in takots	PRO	OR	31098611	CM	JP	2	1	1	1	1	2	0	1	0	0	1	0	0	1	11
CaRdiac Arrest Surviv	PRO	OR	31730900	CM	SZ	2	1	1	1	0	2	0	1	0	2	1	2	0	1	14
Cardiac output during	PRO	OR	31362189	CM	CC	2	1	1	1	1	2	1	1	0	0	1	0	0	1	12

Cardiac output, heart r	PRO	OR	31362081	CM	TK	3	1	1	1	1	2	1	1	0	2	1	2	0	1	17
Cardiogenic Shock Cl	PRO	OR	31548097	CM	jp	2	1	1	1	1	2	1	1	2	2	1	2	2	1	20
Cerebrovascular autore	PRO	OR	31282566	CM	SZ	2	1	1	0	0	2	0	1	0	2	1	0	0	1	11
Change in neuron spec	PRO	OR	30935900	CM	JP	2	1	1	1	1	2	0	1	0	2	1	2	2	1	17
Chemokine RANTES	PRO	OR	31043991	CM	TK	3	1	1	1	0	2	1	1	0	2	1	0	0	1	14
Circulating Levels of r	PRO	OR	31275442	SW	CM	3	1	1	1	1	2	1	1	2	2	1	0	0	1	17
Clinical and Angiogra	PRO	OR	29459218	CM	JP	2	1	1	1	1	2	0	1	0	2	1	2	0	1	15
Cognitive Impairment	PRO	OR	31781396	CM	TK	3	1	1	1	1	2	1	1	0	2	1	0	0	1	15
Cognitive Impairment	PRO	OR	31781396	CM	CM	3	1	1	1	1	2	1	1	0	2	1	0	0	1	15
Combination of Early	PRO	OR	31214555	CM	CC	2	1	1	1	1	2	0	0	0	2	1	0	0	1	12
Combination of Early	PRO	OR	31214555	CM	CM	2	1	1	1	1	2	0	0	0	2	1	0	0	1	12
Combination of S100I	PRO	OR	30732223	SW	CM	3	1	1	1	1	2	1	1	0	2	1	0	0	1	15
Combination of S100I	PRO	OR	30732223	CM	SZ	2	1	1	1	0	2	1	1	0	2	1	0	0	1	13
COMMENCE trial (C	PRO	OR	31815641	CM	JP	4	1	1	1	1	2	1	1	0	2	1	0	0	1	16
Complications of venc	PRO	OR	31394965	CM	TK	2	1	1	1	1	2	1	1	0	2	1	0	0	0	13
Contemporary impacts	PRO	OR	31310845	CM	SW	2	1	1	1	1	2	1	1	0	2	1	0	0	1	14
Coronary artery calcifi	PRO	OR	31901459	CM	TK	2	1	1	1	1	2	0	1	0	2	1	2	0	1	15
Coronary artery calcifi	PRO	OR	31901459	CM	CM	2	1	1	1	1	2	0	1	0	2	1	2	0	1	15
Correlation of Patients	PRO	OR	31172117	CM	SZ	2	1	1	1	0	2	0	1	0	0	1	0	0	0	9
Cortical Thickness anc	PRO	OR	30979357	SW	CM	2	1	1	1	1	2	1	1	0	2	1	0	0	1	14
Deep learning based ou	PRO	OR	30978378	CM	CC	2	1	1	1	1	2	1	1	0	2	1	0	0	1	14
Derivation and Validat	PRO	OR	30819521	CM	JP	2	1	1	1	1	2	0	0	0	2	1	2	0	1	14
Derivation and Validat	PRO	OR	30819521	CM	CC	2	1	1	1	1	2	0	1	0	2	1	2	2	1	17
Derivation and Validat	PRO	OR	30819521	CM	CM	2	1	1	1	1	2	0	1	0	2	1	2	0	1	15
Detailed analysis of he	PRO	OR	30385386	CM	SZ	2	1	1	1	1	2	1	1	2	2	1	0	0	1	16
Determination of Cut-	PRO	OR	31431922	SW	CM	2	1	1	1	0	2	1	1	0	2	1	0	0	0	12
Development and Exte	PRO	OR	30001950	CM	JP	2	1	1	1	1	2	0	1	0	2	1	2	0	1	15
Diagnostic and progno	PRO	OR	31201884	CM	SZ	2	1	1	1	1	0	0	1	0	2	1	0	0	1	11
Differential associatior	PRO	OR	30586605	CM	SW	2	1	1	1	1	2	1	1	0	2	1	0	0	1	14
Distinct predictive val	PRO	OR	30951843	SW	CM	2	1	1	1	1	2	1	1	2	2	1	0	0	1	16
Do combined ultrasou	PRO	OR	31566175	CM	TK	2	1	1	1	1	2	0	0	0	2	1	2	0	0	13
Do combined ultrasou	PRO	OR	31566175	CM	CM	2	1	1	1	0	2	0	0	0	2	1	2	0	1	13
Doppler sonography o	PRO	OR	31145934	CM	CC	2	1	1	1	1	2	1	1	2	2	1	2	0	1	18
Early blood transcripto	PRO	OR	30885824	CM	CC	3	1	1	1	1	0	0	1	2	2	1	0	0	1	14
Early hyperoxemia is r	PRO	OR	31039393	CM	JP	2	1	1	1	1	2	0	1	0	2	1	0	0	1	13
Early Lactate Values A	PRO	OR	29608551	CM	CC	2	1	1	1	1	2	0	1	0	0	1	0	0	1	11
Early Lactate Values A	PRO	OR	29608551	CM	CM	2	1	1	1	1	2	0	1	0	0	1	0	0	1	11
Early prognostication	PRO	OR	31623658	CM	SZ	2	1	1	1	1	2	1	1	0	2	1	0	0	1	14
Early recurrent arrhyth	PRO	OR	31185259	CM	CC	2	1	1	1	1	2	1	1	0	2	1	0	0	1	14
Early-SEPs' amplitude	PRO	OR	30230524	CM	JP	2	1	1	1	1	2	1	1	0	2	1	2	0	1	16
EEG based outcome pi	PRO	OR	31322793	CM	CC	2	1	1	1	1	2	1	1	2	2	1	0	0	1	16
Effects of cholesterol l	PRO	OR	31571440	CM	CC	2	1	1	1	1	2	0	1	0	0	1	0	0	0	10
Effects of cholesterol l	PRO	OR	31571440	CM	CM	2	1	1	1	0	2	1	1	0	0	1	0	0	0	10
Electroencephalographi	PRO	OR	31124174	SW	CM	3	1	1	1	1	2	1	1	2	2	1	2	0	1	19
Electroencephalographi	PRO	OR	31124174	CM	TK	2	1	1	1	1	2	1	1	0	2	1	2	0	1	16
Electroencephalographi	PRO	OR	31124174	CM	CM	2	1	1	1	1	2	1	1	0	2	1	2	0	1	16
Electroencephalography	PRO	OR	31136808	SW	CM	2	1	1	1	0	2	1	1	0	2	1	0	0	1	13
Electromyographic rea	PRO	OR	30885825	CM	TK	2	1	1	1	1	2	0	1	0	2	1	2	0	1	15
Electromyographic rea	PRO	OR	30885825	CM	SZ	2	1	1	1	0	2	0	1	0	2	1	2	0	1	14
End tidal CO(2) and c	PRO	OR	30978377	CM	SZ	2	1	1	1	1	2	1	0	0	2	1	0	0	1	13
Endoplasmic reticulum	PRO	OR	31065605	SW	CM	3	1	1	1	1	2	0	1	0	2	1	0	0	1	14
Evaluation of recurrent	PRO	OR	30478809	CM	JP	2	1	1	1	1	2	1	1	0	2	1	0	0	1	14
Evoked potentials imp	PRO	OR	30995538	CM	TK	2	1	1	1	1	2	1	1	2	2	1	2	0	1	18

External validation of	PRO	OR	31153943	CM	SZ	2	1	1	1	1	2	1	1	0	2	1	2	0	1	16
Factors associated witl	PRO	OR	30033165	CM	SW	2	1	1	1	1	2	1	1	0	2	1	0	0	1	14
Frailty and associated	PRO	OR	31785373	CM	TK	2	1	1	1	1	2	0	1	0	2	1	2	0	1	15
Frailty and associated	PRO	OR	31785373	CM	CM	2	1	1	1	1	2	0	1	0	2	1	2	0	1	15
Frailty is associated w	PRO	OR	30411470	CM	CC	2	1	1	1	1	2	1	1	0	2	1	2	0	1	16
Frailty is associated w	PRO	OR	30411470	CM	CM	2	1	1	1	1	2	1	1	0	2	1	2	0	1	16
Gender differences in c	PRO	OR	31664859	CM	SZ	2	1	1	1	1	2	1	1	0	0	1	0	0	1	12
Glycated Hemoglobin	Pro	OR	31187435	CM	JP	2	1	1	1	1	2	0	1	0	2	1	0	0	1	13
Grey-white matter ratic	PRO	OR	30953628	CM	JP	2	1	1	1	1	2	1	1	0	2	1	0	0	0	13
Growth differentiation	PRO	OR	31624933	CM	SZ	3	1	1	1	1	2	1	1	0	2	1	0	0	1	15
How do information so	PRO	OR	31199943	CM	TK	2	1	1	1	1	0	0	1	2	2	1	2	0	0	14
Hypothermia outcome	PRO	OR	30940473	CM	CC	2	1	1	1	1	0	0	1	0	2	1	2	0	1	13
Hypoxic liver injury a	PRO	OR	30831218	CM	CM	2	1	1	1	1	2	1	1	0	2	1	0	0	1	14
Identifying out-of-hosp	PRO	OR	31711916	CM	TK	2	1	1	1	1	2	1	1	0	2	1	2	0	1	16
Identifying out-of-hosp	PRO	OR	31711916	CM	CM	2	1	1	1	1	2	1	1	0	2	1	2	0	1	16
Impact of age on survi	PRO	OR	31622019	CM	SZ	2	1	1	1	0	2	1	1	0	0	1	0	0	0	10
Impact of hypothermia	PRO	OR	31238037	CM	SC	3	1	1	1	1	2	1	1	2	2	1	0	0	1	17
Impact of rapid lactate	PRO	OR	30934011	CM	TK	2	1	1	1	1	2	1	1	0	2	1	2	0	1	16
Impact of transitory R	PRO	OR	31252641	CM	CC	2	1	1	1	1	2	0	1	0	0	1	2	0	0	12
Implications of Initial	PRO	OR	31279406	CM	JP	2	1	1	1	1	2	1	1	0	2	1	2	0	1	16
Implications of Neurol	PRO	OR	31320013	CM	JP	2	1	1	1	1	2	0	1	2	2	1	2	2	1	19
Incidence and Prognos	PRO	OR	30343292	CM	JP	2	1	1	1	1	2	1	1	0	2	1	0	0	1	14
Incorporating baseline	PRO	OR	31310844	CM	CC	2	1	1	1	1	2	1	1	0	2	1	2	0	1	16
Increased Citrullinated	PRO	OR	31581493	CM	SW	3	1	1	1	1	2	1	1	0	2	1	0	0	1	15
Increased Glucose Vari	PRO	OR	31161969	CM	JP	2	1	1	1	1	2	1	1	0	2	1	0	0	1	14
Influence of comorbidi	PRO	OR	31606397	CM	CM	2	1	1	1	0	2	0	1	2	2	1	0	0	0	13
Influence of comorbidi	PRO	OR	31606397	CM	TK	2	1	1	1	0	2	0	1	0	2	1	0	0	0	11
Initial arterial pH as a	PRO	OR	30946922	CM	TK	2	1	1	1	1	2	1	1	0	2	1	2	0	1	16
Initial end-tidal carbon	PRO	OR	30985538	CM	JP	2	1	1	1	1	2	0	1	0	2	1	2	0	1	15
Initial serum cholester	PRO	OR	31711917	CM	CC	2	1	1	1	1	2	1	1	0	0	1	2	0	1	14
Initial serum cholester	PRO	OR	31711917	CM	CM	2	1	1	1	1	2	1	1	0	0	1	2	0	1	14
Inter-predictability of I	PRO	OR	31259107	CM	TK	2	1	0	1	0	2	1	1	0	2	0	2	0	0	12
Ion shift index as a pro	PRO	OR	30807816	CM	SZ	2	1	1	1	1	2	1	1	0	2	1	2	0	1	16
Ion shift index as a pro	PRO	OR	30807816	CM	SW	2	1	1	1	1	2	1	1	0	2	1	2	0	1	16
Lactate Clearance Pred	PRO	OR	30889788	SW	CM	2	1	1	1	1	2	1	1	0	2	1	2	0	1	16
Late Awakening in Su	PRO	OR	30303838	CM	CC	2	1	1	1	1	2	1	1	0	2	1	2	2	1	18
Late Awakening in Su	PRO	OR	30303838	CM	CM	2	1	1	1	1	2	1	1	0	2	1	2	0	1	16
Lipidomics Detection	PRO	OR	30855329	CM	CC	2	1	1	1	1	2	1	1	0	2	1	0	0	1	14
Long term clinical out	PRO	OR	31859025	CM	JP	2	1	1	1	1	2	0	1	0	2	1	0	0	0	12
Long term renal recove	PRO	OR	31271728	SW	CM	2	1	1	1	1	2	0	1	0	2	1	0	0	1	13
Long-Term Outcomes	PRO	OR	30060961	CM	TK	2	1	1	1	1	2	1	1	0	2	1	2	0	1	16
Long-Term Outcomes	PRO	OR	30060961	CM	CM	2	1	1	1	1	2	1	1	0	2	1	2	0	1	16
Long-Term Survival A	PRO	OR	31262547	CM	CC	2	1	1	1	1	2	0	1	0	2	1	2	0	1	15
Long-Term Survival A	PRO	OR	31262547	CM	CM	2	1	1	1	1	2	0	1	0	2	1	0	0	1	13
Long-term Survival an	PRO	OR	31065204	CM	SZ	2	1	1	1	1	2	0	1	0	2	1	2	0	1	15
Long-termsurvival in p	PRO	OR	30603662	CM	JP	2	1	1	1	1	2	1	1	0	2	1	0	0	1	14
Markers of cardiogenic	PRO	OR	30470603	CM	CM	2	1	1	1	1	2	1	1	0	0	1	0	0	1	12
Markers of cardiogenic	PRO	OR	30470603	CM	CC	2	1	1	1	1	2	1	1	0	0	1	0	0	0	11
Mean arterial pressure	PRO	OR	30586655	CM	SZ	2	1	1	1	1	2	1	1	2	2	1	2	0	1	18
Mild hypothermia imp	PRO	OR	31428461	CM	TK	3	1	1	0	0	2	1	1	0	2	1	0	0	1	13
Moderate brain hypoth	PRO	OR	30679007	SW	CM	4	1	1	1	1	2	1	1	0	2	1	0	0	1	16
Modulating effects of i	PRO	OR	31422106	SW	CM	2	1	1	1	1	2	1	1	0	2	1	0	0	1	14
Multimodal approach	PRO	OR	30562594	CM	JP	2	1	1	1	1	2	1	1	0	2	1	0	0	1	14

Neurological outcome	PRO	OR	31300383	CM	CC	2	1	1	1	1	2	0	1	2	2	1	2	0	1	17
Neurological pupil ind	PRO	OR	31069659	CM	CC	2	1	1	0	1	2	1	1	2	2	1	0	0	1	15
Neurological Pupil inc	PRO	OR	31870911	CM	TK	3	1	1	1	1	2	1	1	0	2	1	0	0	1	15
Neurological Pupil inc	PRO	OR	31870911	CM	CM	3	1	1	1	1	2	1	1	0	2	1	0	0	1	15
Neuron-specific enolas	PRO	OR	31306716	CM	JP	2	1	1	1	1	2	1	1	0	2	1	2	0	1	16
Neuron-specific enolas	PRO	OR	30857978	CM	TK	2	1	1	1	1	2	0	1	0	2	1	2	0	1	15
Neurophysiological an	PRO	OR	31763408	CM	SW	2	0	1	1	1	2	1	1	0	2	1	2	0	1	15
Neurophysiology and	PRO	OR	31400398	CM	CC	2	1	1	1	1	2	1	1	2	2	1	2	0	1	18
Neurophysiology for p	PRO	OR	31790754	CM	SZ	2	1	1	1	1	2	1	1	0	2	1	2	0	1	16
Neuroprognostication	PRO	OR	31743225	CM	JP	1	1	1	1	1	2	1	1	2	2	1	0	0	1	15
Neutrophil-lymphocyt	PRO	OR	30815378	CM	JP	2	1	1	1	1	2	1	1	0	2	1	2	0	1	16
Number of Circulating	PRO	OR	31237169	CM	TK	3	1	1	1	1	2	1	1	2	2	1	0	0	1	17
Obesity is associated v	PRO	OR	31526656	CM	SZ	2	1	1	1	1	2	1	1	0	2	1	0	0	1	14
On-the-Scene Hyaluror	PRO	OR	31148947	CM	TK	3	1	1	1	1	2	1	1	0	2	1	0	0	1	15
One-year outcome of p	PRO	OR	31678408	CM	SZ	2	1	1	1	1	2	0	1	2	2	1	0	0	0	14
Optimal Hemodynami	PRO	OR	31633449	CM	CC	2	1	1	1	1	2	1	1	0	0	1	2	0	1	14
Optimal Hemodynami	PRO	OR	31633449	CM	CM	2	1	1	1	1	2	1	1	0	0	1	2	0	1	14
Optimal timing to mea	PRO	OR	31306717	CM	SZ	2	1	1	1	1	2	1	1	0	2	1	0	0	1	14
Out of hospital cardiac	PRO	OR	31423870	CM	CC	2	1	1	1	1	2	1	1	0	0	1	0	0	0	11
Outcome after type A	PRO	OR	31505231	CM	TK	2	1	1	1	1	0	0	1	0	2	1	2	0	1	13
Outcome after type A	PRO	OR	31505231	CM	CM	2	1	1	1	0	0	0	1	0	2	1	2	0	0	11
Outcome prediction of	PRO	OR	31220514	SW	CM	2	1	1	1	1	2	0	1	0	2	1	0	0	1	13
Outcome predictors of	PRO	OR	31714000	CM	SW	2	1	1	1	1	2	0	1	0	2	1	0	0	1	13
Outcome, quality of li	PRO	OR	31771619	CM	CC	2	1	1	1	1	2	1	1	0	2	1	0	0	1	14
Outcome, quality of li	PRO	OR	31771619	CM	CM	2	1	1	1	1	2	1	1	0	2	1	0	0	1	14
Outcomes after out-of-	PRO	OR	31988770	CM	TK	2	1	1	1	1	2	1	1	0	2	1	2	0	1	16
Outcomes after out-of-	PRO	OR	31988770	CM	CM	2	1	1	1	1	2	1	1	0	2	1	2	0	1	16
Outcomes of survivors	PRO	OR	30455410	CM	SZ	2	1	1	1	1	2	0	1	0	0	1	0	0	0	10
Oximetry-Guided norn	PRO	OR	31063840	CM	SZ	4	1	1	1	0	2	1	1	0	2	1	2	0	1	17
Performance of clinical	PRO	OR	30391369	CM	CM	2	1	1	1	1	2	1	1	2	2	1	2	0	1	18
Performance of clinical	PRO	OR	30391369	CM	CC	2	1	1	1	1	2	1	1	0	0	1	2	0	1	14
Plasma Adenylate Lev	PRO	OR	30052576	CM	JP	2	1	1	1	1	2	1	1	0	2	1	0	0	1	14
Post resuscitation prog	PRO	OR	30391368	CM	JP	2	1	1	1	1	2	0	1	0	2	1	0	0	1	13
Prearrest prediction of	PRO	OR	31412292	CM	TK	2	1	1	1	1	2	1	1	0	2	1	0	0	1	14
Predicting Outcome A	PRO	OR	31466497	CM	SZ	2	1	1	1	1	2	0	1	2	2	1	2	0	1	17
Predicting Outcomes c	PRO	OR	31512185	CM	SZ	2	1	1	1	1	2	1	1	0	2	1	2	0	1	16
Predicting survival in	PRO	OR	30994183	CM	SZ	2	1	1	1	0	2	0	1	0	2	1	2	0	1	14
Predicting the probabil	PRO	OR	31821836	CM	CM	2	1	1	1	1	2	0	1	0	2	1	2	0	1	15
Predicting the probabil	PRO	OR	31821836	CM	TK	2	1	1	1	1	2	0	1	0	2	1	2	0	0	14
Prediction of good neu	PRO	OR	31362082	SW	CM	2	1	1	1	1	2	1	1	0	2	1	0	0	1	14
Predictive performance	PRO	OR	31441881	CM	JP	3	1	1	1	1	2	1	1	0	2	1	0	0	1	15
Predictors and their pr	PRO	OR	31628390	CM	SW	2	1	1	1	1	2	1	1	0	2	1	0	0	1	14
Predictors of Neurolog	PRO	OR	31932021	CM	JP	2	1	1	1	1	2	0	1	0	2	1	0	0	1	13
Presepsin As a Bioma	PRO	OR	30985453	CM	SZ	2	1	1	1	0	2	1	1	0	2	1	0	0	1	13
Prognostic Factors for	PRO	OR	31932022	CM	SW	2	1	1	1	1	2	0	1	0	2	1	2	0	1	15
Prognostic impact of c	PRO	OR	30578436	CM	JP	2	1	1	1	1	2	0	1	0	2	1	0	0	1	13
Prognostic impact of t	PRO	OR	31063844	CM	CC	2	1	1	1	1	2	1	1	0	0	1	2	0	1	14
Prognostic performanc	PRO	OR	30797049	CM	JP	2	1	1	1	1	2	1	1	0	2	1	2	0	1	16
Prognostic performanc	PRO	OR	30797049	CM	SW	2	1	1	1	1	2	1	1	0	2	1	2	0	1	16
Prognostic value of blo	PRO	OR	31648431	CM	SZ	2	0	1	0	0	2	1	0	0	0	1	2	0	1	10
Prognostic Value of B	PRO	OR	31276111	CM	JP	2	1	1	1	1	2	1	1	0	2	1	2	0	1	16
Prognostic Value of bl	PRO	OR	31276111	CM	JP	2	1	1	1	1	2	1	1	0	2	1	2	0	1	16
Prognostic value of ph	PRO	OR	31153942	CM	JP	2	1	1	1	1	2	1	1	0	2	1	0	0	1	14

Prognostic Value of S	PRO	OR	31565439	CM	TK	3	1	1	1	1	2	1	1	0	2	1	0	0	1	15
Prognostic Value of S	PRO	OR	31565439	CM	CM	2	1	1	1	0	2	1	1	0	2	1	0	0	1	13
Prognostic Value of th	PRO	OR	31075958	CM	TK	2	1	1	1	1	2	0	1	0	2	1	2	0	1	15
Prognostication after c	PRO	OR	30902688	CM	JP	1	1	1	1	1	2	0	1	2	0	1	0	0	1	12
Prognostication via ea	PRO	OR	31521958	CM	jp	2	1	1	1	1	2	0	1	0	2	1	0	0	1	13
Prospective validation	PRO	OR	31078496	CM	TK	2	1	1	1	1	2	1	1	0	2	1	2	0	0	15
Pulseless electrical acti	PRO	OR	31147183	CM	CC	2	1	1	0	1	2	0	1	0	0	1	2	0	1	12
Quantitative EEG reac	PRO	OR	31419742	CM	SZ	2	1	1	1	1	2	1	1	2	2	1	0	0	1	16
Quantitative Electroen	PRO	OR	31241498	CM	CM	2	1	1	1	1	2	1	1	2	2	1	0	0	1	16
Quantitative Electroen	PRO	OR	31241498	CM	CC	2	1	1	1	1	2	1	1	0	2	1	0	0	1	14
Reasons for death in p	PRO	OR	30710595	CM	CC	2	1	1	1	1	2	0	1	2	2	1	0	0	1	15
Recovery from acute k	PRO	OR	31307504	SW	CM	2	1	1	1	1	2	1	1	0	2	1	0	0	1	14
Reexamination of the	PRO	OR	31150083	SW	CM	2	1	1	1	1	2	1	1	2	2	1	2	2	1	20
Relation of Chronic T	PRO	OR	30967290	CM	TK	2	1	1	1	1	2	1	1	0	2	1	0	0	1	14
Relation of Chronic T	PRO	OR	30967290	CM	TK	2	1	1	1	1	2	1	1	0	2	1	0	0	1	14
Relation of Chronic T	PRO	OR	30967290	CM	CM	2	1	1	1	1	2	1	1	0	2	1	0	0	0	13
Relationship Between	PRO	OR	31821187	CM	CC	2	1	1	0	1	2	1	1	2	2	1	2	0	1	17
Relationship Between	PRO	OR	31821187	CM	CM	2	1	1	0	1	2	1	1	2	2	1	2	0	1	17
Relationship between	PRO	OR	31628979	CM	SW	2	1	1	1	1	2	1	1	0	2	1	0	0	1	14
Relationship Between	PRO	OR	30851940	CM	SZ	2	1	1	1	1	2	0	1	0	0	1	0	0	1	11
Relative tachycardia is	PRO	OR	29936012	CM	SZ	2	1	1	1	1	2	1	1	0	2	1	0	0	1	14
Resistin - Can it be a	PRO	OR	30650128	CM	TK	3	1	1	1	1	2	1	0	0	2	1	2	0	1	16
Resistin - Can it be a	PRO	OR	30650128	CM	CM	3	1	1	1	1	2	1	1	0	2	1	0	0	1	15
Resistin - Can it be a	PRO	OR	30650128	CM	CC	3	1	1	1	0	2	1	1	0	2	1	0	0	1	14
Risk factors for progre	PRO	OR	30963296	CM	CC	2	1	1	1	1	2	1	1	0	2	1	2	0	1	16
Serum neurofilament l	PRO	OR	31375414	CM	JP	2	1	1	1	1	2	1	1	0	2	1	0	0	1	14
Serum Neurofilament	PRO	OR	30383090	CM	CM	3	1	1	1	1	2	1	1	2	2	1	2	0	1	19
Serum Neurofilament	PRO	OR	30383090	CM	CC	2	1	1	1	1	2	1	1	2	2	1	2	0	1	18
Serum potassium level	PRO	OR	31081678	SW	CM	2	1	1	1	1	2	0	1	0	2	1	2	0	1	15
Serum tau fragments a	PRO	OR	31017476	CM	CC	3	1	1	1	1	2	1	1	2	2	1	0	0	1	17
Sources of interrater va	PRO	OR	30847430	CM	TK	2	1	1	1	0	0	0	1	0	2	1	0	0	1	10
Spectral Content of El	PRO	OR	30422916	CM	JP	2	1	1	1	1	2	0	1	0	2	1	0	0	1	13
The association betwe	PRO	OR	31626864	CM	TK	2	1	1	1	1	2	0	1	0	2	1	0	0	1	13
The association betwe	PRO	OR	31626864	CM	CM	2	1	1	1	1	2	0	1	0	2	1	0	0	1	13
The association betwe	PRO	OR	30015516	CM	TK	3	1	1	1	1	2	1	1	0	2	1	0	0	0	14
The association betwe	PRO	OR	30015516	CM	CM	3	1	1	1	1	2	1	1	0	2	1	0	0	0	14
The association of chr	PRO	OR	30794830	SW	CM	2	1	1	1	1	2	0	1	0	2	1	0	0	1	13
The central role of CT	PRO	OR	31892623	CM	CC	1	0	0	1	0	0	1	1	0	0	1	0	0	1	6
The central role of CT	PRO	OR	31892623	CM	CM	1	0	0	1	0	0	1	1	0	0	1	0	0	1	6
The complement lectir	PRO	OR	31538810	CM	SZ	4	1	1	1	1	2	1	1	0	2	1	0	0	1	16
The EXACT protocol:	PRO	OR	31009695	SW	CM	4	1	1	0	0	2	1	1	0	2	1	0	0	0	13
The gradient between a	PRO	OR	29685358	CM	JP	2	1	1	1	1	2	0	1	0	2	1	0	0	1	13
The prognostic signific	PRO	OR	30404678	CM	CC	2	1	1	1	1	2	1	1	0	0	1	2	0	0	13
The prognostic signific	PRO	OR	30404678	CM	CM	2	1	1	1	1	2	1	1	0	0	1	0	0	0	11
The Prognostic Useful	PRO	OR	31306348	CM	TK	2	1	1	1	1	2	0	1	0	2	1	2	0	1	15
The Prognostic Value	PRO	OR	30112686	CM	SW	2	1	1	1	1	2	1	1	0	2	1	2	0	1	16
The usefulness of neur	PRO	OR	31585184	CM	TK	3	1	1	1	1	2	0	1	0	2	1	2	0	1	16
The usefulness of neur	PRO	OR	31585184	CM	CM	3	1	1	1	1	2	0	1	0	2	1	0	0	1	14
Time course of platele	PRO	OR	31132395	CM	JP	2	1	1	1	1	2	1	1	0	2	1	0	0	1	14
Timing of brain comp	PRO	OR	31585185	CM	SZ	2	1	1	1	1	2	0	1	0	2	1	2	0	1	15
Tissue-Specific Metab	PRO	OR	31475603	CM	JP	3	1	1	1	1	2	1	1	0	2	1	0	0	1	15
Turn-to-Shockable Rh	PRO	OR	31411544	CM	JP	2	1	1	1	1	2	1	1	0	2	1	0	0	1	14
Urine Î²-2-Microglobul	PRO	OR	31205786	CM	SZ	2	1	1	1	1	2	1	1	0	2	1	0	0	1	14

Usefulness of a quantit	PRO	OR	30831551	CM	SZ	2	1	1	1	1	2	0	1	0	2	1	0	0	1	13
Usefulness of Intracran	PRO	OR	31526251	CM	CC	2	1	1	1	1	2	1	1	0	0	1	2	0	1	14
Usefulness of Intracran	PRO	OR	31526251	CM	CM	2	1	1	1	1	2	1	1	0	0	1	2	0	1	14
Usefulness of neuron s	PRO	OR	31022497	SW	CM	2	1	1	1	1	2	1	1	0	2	1	2	0	1	16
Usefulness of Trends i	PRO	OR	31405547	CM	TK	2	1	1	1	1	2	0	1	0	2	1	2	0	1	15
Validation of the retur	PRO	OR	30447262	CM	SW	2	1	1	1	1	2	0	1	0	2	1	2	0	1	15
Value of assessment o	PRO	OR	30248375	CM	SW	2	1	1	1	1	2	1	1	0	2	1	0	0	1	14
Value of capnography	PRO	OR	30836170	CM	CC	2	1	1	1	1	0	0	1	0	2	1	0	0	1	11
Value of EEG reactivit	PRO	OR	31211949	CM	JP	2	1	1	1	1	2	1	1	0	2	1	2	0	0	15
Ventricular Fibrillatior	PRO	OR	30626208	CM	SZ	2	1	1	1	1	2	1	1	0	2	1	0	0	1	14
Vitamin D Deficiency	PRO	OR	31524672	CM	SZ	2	1	1	1	1	2	1	1	0	2	1	2	0	1	16
