# Supplementary material for: Cardiac arrest: An interdisciplinary scoping review of the literature from 2019
Source: Resusc Plus. 2020 Nov 4;4:100037. doi: 10.1016/j.resplu.2020.100037 (PMC8244427; doi:10.1016/j.resplu.2020.100037)
Supplement: Supplementary file 5 [file mmc5.pdf]

Article & Reviewer Information						Clarity (5 max)				Design (7 max)				Importance (5 max)			Impact (5 max)			
Citation Title	Category (EPH, PRE, IN, PRO, PED, BSP, GL)	Type: Original Research (OR) or Review (RE)	PubMed ID	Editor Initials	Reviewer Initials	A	B	C	D	A	B	C	D	A	B	C	A	B	C	Total score (22 max)
Disseminated intravasc	BSP	RE	31102491	SG	SC	2	1	1	1	0	0	0	1	2	2	1	2	0	1	14
Sodium-Hydrogen Exc	BSP	RE	31067690	SG	SC	2	1	1	1	0	0	0	0	2	2	1	2	2	1	15
Aspirin to Prevent Suc	EPH	RE	30296406	MPB/MABC	YE	2	1	1	1	0	0	0	0	2	2	1	2	0	1	13
Atrial fibrillation and r	EPH	RE	31353765	MPB/MABC	EM	2	1	1	1	3	2	1	1	0	2	1	2	0	1	18
Autoantibodies for Ca	EPH	RE	30009713	MABC	MABC	2	1	1	1	0	0	0	0	0	0	0	2	2	1	10
Automated external de	EPH	RE	30594573	MPB/MABC	SB	2	1	1	1	0	0	0	0	0	0	0	2	2	1	10
Bystander CPR trainin	EPH	RE	31533000	MPB/MABC	YE	2	1	1	1	3	2	1	1	2	2	1	2	0	1	20
Can cardiac resynchron	EPH	RE	31004607	MPB/MABC	MABC	2	1	0	0	0	0	0	0	0	2	1	2	2	1	11
Canadian Cardiovascul	EPH	RE	31679625	MPB/MABC	SB	2	1	1	1	0	0	0	0	0	2	1	2	2	1	13
Channelopathies That	EPH	RE	30389366	MPB/MABC	SB	2	1	1	1	0	0	0	0	0	0	0	2	2	1	10
Current Device Therap	EPH	RE	30389367	MPB/MABC	EM	2	1	1	1	0	2	0	1	0	2	1	2	0	1	14
Diagnostic Algorithms	EPH	RE	30471046	MPB/MABC	JW	2	1	1	1	0	2	1	1	2	2	1	2	0	1	17
Disparities in Death R	EPH	RE	30594574	MPB/MABC	EM	2	1	1	1	3	2	1	1	0	2	1	2	2	1	20
Do-not-intubate orders	EPH	RE	31659387	MPB/MABC	MPB	2	1	1	1	3	2	1	1	0	2	1	0	0	1	16
Effect of smart devices	EPH	RE	31325556	MPB/MABC	SB	2	1	1	1	3	2	1	1	0	2	1	2	0	1	18
Effectiveness of the 20	EPH	RE	30366935	MABC	MABC	2	1	1	1	3	2	1	1	2	2	1	2	2	1	22
Epidemiology of Sudd	EPH	RE	30482683	MPB/MABC	YE	2	1	1	1	0	0	0	1	2	2	1	2	2	1	16
European Cardiac Arrh	EPH	RE	31482331	MPB/MABC	YE	2	1	1	1	0	0	0	1	2	2	1	2	2	1	16
Evaluation of tools to	EPH	RE	31684872	MPB/MABC	SB	2	1	1	1	3	2	1	1	0	2	1	2	2	1	20
Factors Impacting Pati	EPH	RE	31100851	MPB/MABC	MPB	2	1	1	1	3	2	1	1	0	0	0	0	0	1	13
Family presence during	EPH	RE	31733825	MPB/MABC	MPB	2	1	1	1	0	0	0	0	0	0	1	2	2	1	11
Imaging for sudden ca	EPH	RE	31054859	MPB/MABC	MPB	2	1	1	1	0	0	0	1	2	2	1	0	0	1	12
Impact of Implantable	EPH	RE	30052536	MPB/MABC	EM	2	1	1	1	3	2	1	1	0	2	1	2	0	1	18
Impact of mineralocort	EPH	RE	30264282	MPB/MABC	YE	2	1	1	1	3	0	0	1	2	2	1	2	2	1	19
Implantable cardiovert	EPH	RE	31471226	MPB/MABC	EM	2	1	1	1	3	2	0	1	0	2	1	2	0	1	17
Interventions to impro	EPH	RE	30759140	MPB/MABC	MPB	2	1	1	1	3	2	1	1	2	2	1	2	2	1	22
Is Sauna Bathing Prot	EPH	RE	31102597	MPB/MABC	EM	2	1	1	1	0	2	1	1	0	2	1	0	0	1	13
Is sauna bathing protec	EPH	RE	31102597	MPB/MABC	MABC	2	1	0	1	0	0	0	1	0	2	0	0	2	0	9
Key components of a c	EPH	RE	30858511	MPB/MABC	MPB	2	1	1	1	0	0	0	1	2	2	1	2	0	0	13
Major Adverse Cardiac	EPH	RE	30557212	MPB/MABC	JW	2	1	1	1	0	2	1	1	2	2	1	0	0	0	14
Mechanisms of Arrhyt	EPH	RE	30825952	MPB/MABC	YE	2	1	1	1	0	0	0	1	2	2	1	2	0	1	14
Mitral valve prolapse a	EPH	RE	30242141	MPB/MABC	MPB	2	1	1	1	3	2	1	1	2	2	1	0	0	1	18
Nighttime is associate	EPH	RE	30630680	MPB/MABC	MABC	2	1	1	1	3	2	1	1	2	2	1	2	2	1	22
Nighttime is associate	EPH	RE	30630680	MPB/MABC	YE	2	1	1	1	3	2	1	1	0	2	1	2	2	1	20
Non-arrhythmic causes	EPH	RE	31075277	MPB/MABC	MPB	2	1	1	1	0	0	0	1	0	2	1	0	0	1	10
Predictors of Adverse C	EPH	RE	31180938	MABC	MABC	2	1	1	1	3	2	1	1	0	2	1	2	2	1	20
Prevention of Sudden	EPH	RE	31022955	MPB/MABC	YE	2	1	1	1	0	0	0	1	2	2	1	2	2	1	16
Prevention of Sudden	EPH	RE	31022955	MPB/MABC	MPB	2	1	1	1	0	0	0	1	0	2	1	2	2	1	14
Race, ethnicity, and th	EPH	RE	30029848	MPB/MABC	JW	2	1	1	1	0	2	0	1	2	2	1	2	0	1	16
Relation of Body Mas	EPH	RE	31105141	MPB/MABC	SB	2	1	1	1	3	2	1	1	0	2	1	2	2	1	20
Risk stratification for v	EPH	RE	31422711	MPB/MABC	MPB	2	1	1	1	0	0	0	1	0	2	1	0	0	1	10
Shock Index as a Predi	EPH	RE	30719462	MPB/MABC	YE	2	1	1	1	0	0	0	1	2	2	1	2	0	1	14
Socio-economic differe	EPH	RE	31199944	MPB/MABC	JW	2	1	1	1	3	2	1	1	2	2	1	0	0	1	18
Sudden Cardiac Death	EPH	RE	31118017	MABC	MABC	2	1	1	1	0	0	0	0	0	0	0	2	2	1	10

Sudden Cardiac Death	EPH	RE	30392982	MPB/MABC	JW	2	1	1	1	0	0	0	0	2	2	1	2	0	1	13
Sudden Cardiac Death	EPH	RE	31008774	MPB/MABC	MABC	2	1	1	0	0	0	0	0	0	2	0	2	2	0	10
Sudden cardiac death i	EPH	RE	31538192	MPB/MABC	YE	2	1	1	1	0	0	0	0	2	2	1	2	0	1	13
Sudden Cardiac Death	EPH	RE	31279425	MPB/MABC	YE	2	1	1	1	0	0	0	1	2	2	1	2	0	1	14
Sudden cardiac death i	EPH	RE	31075279	MPB/MABC	MABC	2	1	0	1	0	0	0	0	0	2	1	2	0	1	10
Sudden Cardiac Death	EPH	RE	31199891	MPB/MABC	MABC	2	1	1	0	0	0	0	0	0	2	0	2	2	0	10
Sudden Cardiac Death	EPH	RE	30447717	MABC	MABC	2	1	1	1	0	0	0	0	0	0	0	2	2	1	10
Sudden Cardiac Death:	EPH	RE	31378334	MPB/MABC	JW	2	1	1	1	0	0	0	0	2	0	1	0	0	0	8
Sudden Death and Ver	EPH	RE	30554599	MPB/MABC	SB	2	1	1	1	0	0	0	0	0	0	0	2	2	1	10
Sudden death in heart	EPH	RE	31147814	MPB/MABC	EM	2	1	1	1	0	2	1	1	0	2	1	2	0	1	15
Sudden Death Risk-St	EPH	RE	30482684	MPB/MABC	YE	2	1	1	1	0	0	0	1	2	2	1	2	0	1	14
Sudden unexpected de	EPH	RE	30566897	MPB/MABC	MPB	2	1	1	1	0	0	0	1	2	2	1	2	0	1	14
Systematic Meta-Analy	EPH	RE	31447468	MPB/MABC	SB	2	1	1	1	3	2	1	1	0	2	1	2	2	1	20
Systematic Meta-Analy	EPH	RE	31447468	MPB/MABC	MPB	0	0	1	1	3	2	1	1	0	2	1	2	0	1	15
Systematic review of t	EPH	RE	31740470	MPB/MABC	JW	2	1	1	1	3	2	1	1	2	2	1	2	0	1	20
The Danish in-hospital	EPH	RE	31191032	MPB/MABC	EM	2	1	1	1	0	2	0	1	0	2	1	2	0	1	14
The incidence and outc	EPH	RE	30593842	MPB/MABC	MPB	2	1	1	1	3	2	1	1	2	2	1	2	0	1	20
The incidence of cardia	EPH	RE	31037107	MPB/MABC	YE	2	1	1	1	3	2	1	1	2	2	1	2	0	1	20
The necessity of impla	EPH	RE	30642644	MABC	MABC	2	1	1	1	0	0	0	0	0	0	0	2	2	1	10
The prevention and ma	EPH	RE	31471226	MPB/MABC	EM	2	1	1	1	0	2	0	1	0	2	1	2	2	1	16
The prevention and ma	EPH	RE	31308007	MPB/MABC	SC	2	0	1	1	0	0	0	0	2	2	1	2	0	1	12
The role of cardiorespi	EPH	RE	31075278	MPB/MABC	MPB	2	1	1	1	3	2	0	1	2	2	1	2	0	1	19
Towards cardiac MRI	EPH	RE	30377260	MPB/MABC	YE	2	1	1	1	0	0	0	1	2	2	1	2	0	1	14
Training Adult Laypeo	EPH	RE	30808611	MPB/MABC	MABC	2	1	1	1	3	2	1	1	2	2	1	2	2	1	22
Treatment of ventricula	EPH	RE	30268648	MPB/MABC	SB	2	1	1	1	0	0	0	0	0	0	0	2	2	1	10
What causes sudden de	EPH	RE	31390006	MPB/MABC	MPB	2	0	1	1	0	0	0	1	2	2	1	0	0	1	11
2019 American Heart	GL	RE	31722552	RB	DR	2	1	1	1	3	0	0	1	2	2	1	2	2	1	19
2019 American Heart	GL	RE	31722552	RB	RB	2	1	1	1	3	0	0	1	2	2	1	2	2	1	19
2019 American Heart	GL	RE	31722563	RB	RB	2	1	1	1	3	2	1	1	2	2	1	2	2	1	22
2019 American Heart	GL	RE	31722563	RB	JD	2	1	1	1	3	2	1	1	2	2	1	2	2	1	22
2019 International Co	GL	RE	31734223	RB	RB	2	1	1	1	3	0	0	1	2	2	1	2	2	1	19
2019 International Co	GL	RE	31734223	RB	DR	2	1	1	1	0	2	0	1	2	2	1	2	2	1	18
A Complete Review o	GL	RE	30971592	RB	JD	2	0	1	1	3	2	0	1	2	2	1	2	0	1	18
A Systematic Literatu	GL	RE	31695740	RB	JD	2	1	1	1	3	2	1	1	2	2	1	2	2	1	22
A systematic literature	GL	RE	31695740	RB	RB	2	1	1	1	3	2	1	1	2	2	1	2	0	1	20
Advanced Airway Ma	GL	RE	30981882	RB	JD	2	1	1	1	3	2	1	1	2	2	1	2	2	1	22
Advanced airway man	GL	RE	30981882	RB	RB	2	1	1	1	3	2	1	1	2	2	1	2	2	1	22
Advances in the Risk	GL	RE	31255188	RB	JD	2	1	1	1	0	0	0	0	0	2	1	2	0	1	11
Advances in the Risk	GL	RE	31255188	RB	RB	2	1	1	1	0	0	0	0	0	2	1	0	0	1	9
An International, Cons	GL	RE	30925524	RB	JD	2	1	1	1	3	2	0	1	2	2	1	2	2	1	21
An international, cons	GL	RE	30925524	RB	RB	2	1	1	1	0	0	0	0	2	2	1	2	0	0	12
Cadaver models for ca	GL	RE	31412293	RB	DR	2	1	1	1	0	2	1	1	2	2	1	2	2	1	19
Cadaver models for ca	GL	RE	31412293	RB	RB	2	1	1	1	0	2	1	1	2	2	1	2	0	1	17
Canadian Cardiovascul	GL	RE	30595170	RB	DR	2	1	1	1	0	0	0	0	0	2	1	2	0	0	10
Canadian Cardiovascul	GL	RE	30595170	RB	RB	2	1	1	1	0	0	0	1	0	2	1	2	0	0	11
Cardiac Surgical Resu	GL	RE	31351560	RB	DR	2	1	0	1	0	0	0	0	2	2	1	2	2	0	13
COACHRED: A Proto	GL	RE	31456338	RB	JD	2	1	1	1	0	0	0	0	2	2	1	2	2	1	15
European Resuscitatio	GL	RE	30496838	RB	RB	2	1	1	1	0	0	0	0	2	2	1	2	0	0	12
European Resuscitatio	GL	RE	30496838	RB	DR	2	1	1	1	0	0	0	0	2	2	1	2	0	1	13
In-flight Cardiac Arres	GL	RE	30232737	RB	JD	2	0	1	1	0	0	0	0	0	0	1	0	0	1	6
Invasive Management	GL	RE	31510774	RB	JD	2	1	1	1	0	0	0	0	0	2	1	2	0	1	11
Meta-Analysis Compa	GL	RE	31858970	RB	JD	2	1	1	1	3	2	1	1	2	2	1	2	2	1	22

New Approaches in th	GL	RE	31100908	RB	RB	2	1	1	1	0	0	0	0	0	2	1	2	0	1	11
New Approaches in th	GL	RE	31100908	RB	JD	2	1	1	1	0	0	0	1	0	2	0	0	0	1	9
New Concepts in Sudo	GL	RE	30621954	RB	JD	2	1	1	1	0	0	0	1	2	2	1	2	0	1	14
New Concepts in Sudo	GL	RE	30621954	RB	RB	2	1	1	1	0	0	0	1	2	2	1	2	0	1	14
Optimizing the Early I	GL	RE	31512559	RB	DR	2	1	0	0	0	0	0	0	2	2	1	2	0	1	11
Optimizing the Early I	GL	RE	31512559	RB	RB	2	1	1	0	0	0	0	0	2	2	1	2	0	1	12
Personalized physiolog	GL	RE	30741366	RB	DR	2	1	0	1	0	0	0	0	0	2	1	2	0	0	9
Recent advances in per	GL	RE	31249674	RB	DR	2	1	0	1	0	0	0	0	2	2	1	2	0	1	12
Recent advances in per	GL	RE	31249674	RB	RB	2	1	1	1	0	0	0	0	2	2	1	2	0	1	13
Recommendations for	GL	RE	30361819	RB	DR	2	1	1	1	0	0	0	0	2	0	1	2	2	1	13
Resuscitation of the Pı	GL	RE	30940377	RB	DR	2	1	0	1	0	0	0	0	0	2	1	2	2	0	11
Resuscitation of the Pı	GL	RE	30940377	RB	RB	2	1	1	1	0	0	0	0	0	2	1	2	2	0	12
The changes in cardiop	GL	RE	31938692	RB	DR	2	1	1	1	0	0	0	0	2	0	1	2	2	1	13
The changes in cardiop	GL	RE	31938692	RB	RB	2	1	1	1	0	0	0	0	2	2	1	2	0	1	13
The Critical Care Liter	GL	RE	31831348	RB	JD	2	1	1	1	3	0	0	1	2	2	1	2	2	1	19
Update on Prevention	GL	RE	30449537	RB	JD	2	1	1	1	0	0	0	0	2	2	1	2	0	1	13
Update on prevention a	GL	RE	30449537	RB	RB	0	1	1	1	0	0	0	0	2	2	1	2	0	1	11
Vasopressors during A	GL	RE	30980877	RB	JD	2	1	1	1	3	2	1	1	2	2	1	2	2	1	22
Vasopressors during ad	GL	RE	30980877	RB	RB	2	1	1	1	3	2	1	1	2	2	1	2	2	1	22
Ventilation During Ca	GL	RE	31138729	RB	DR	2	1	0	1	0	0	0	0	2	2	1	2	0	0	11
Adult post-cardiac arre	IN	RE	31846693	CH	GS	2	1	1	1	3	2	1	1	2	2	1	0	0	0	17
Beta-blockade for the t	IN	RE	31790759	DC	DR/KH	2	1	1	1	3	2	1	1	2	2	1	2	2	1	22
Can Systemic Thromb	IN	RE	31594741	CH	KD	2	1	1	1	3	2	1	1	2	2	1	2	0	1	20
Cardiac arrest after card	IN	RE	30710033	CH	DB	2	1	1	1	0	0	0	1	0	2	1	2	2	0	13
Cardiac Arrest and Car	IN	RE	31522544	CH	DC	2	1	1	1	0	0	0	0	2	2	1	0	0	1	11
Cardiac Arrest and Car	IN	RE	31536777	CH	CH	2	1	1	1	0	0	0	0	2	2	1	0	0	1	11
Cardiac Arrest and Car	IN	RE	31536777	CH	RL	2	1	0	1	0	0	0	0	2	2	1	0	0	1	10
Clinical Efficacy of Ex	IN	RE	31360719	CH	GS	2	1	1	1	3	2	1	1	2	2	1	2	2	1	22
Clinical Pearls of Ven	IN	RE	31364329	CH	GS	2	1	1	1	0	0	0	0	2	2	1	2	0	0	12
Contemporary manage	IN	RE	31867056	CH	DB	2	1	1	1	3	0	0	1	2	2	1	2	2	1	19
Coronary angiography	IN	RE	31442472	CH	DB	2	1	1	1	0	2	1	1	0	2	1	2	0	1	15
Diagnostic yield of no	IN	RE	30201536	CH	GS	2	1	1	1	0	2	1	1	2	2	1	2	0	1	17
Does care at a cardiac a	IN	RE	30779976	CH	RL	2	1	1	1	3	2	1	1	2	2	1	2	0	1	20
Effect of different metl	IN	RE	31443696	CH	DC	2	1	1	1	3	2	1	1	2	2	1	2	2	1	22
Extended Indications f	IN	RE	31014165	CH	GS	2	1	1	1	0	0	0	0	0	2	1	2	0	0	10
Extra corporeal membı	IN	RE	30817400	CH	RL	2	1	1	1	0	0	0	1	2	2	1	2	2	1	16
Extracorporeal membra	IN	RE	31618362	CH	DB	2	1	1	1	3	2	0	1	2	2	1	2	2	0	20
Gender differences in t	IN	RE	31409393	CH	KD	2	1	1	1	3	2	1	1	2	2	1	2	0	1	20
Hospital management	IN	RE	30747004	CH	DC	2	1	1	1	0	0	0	0	2	2	1	2	0	0	12
Hypothermia and cardi	IN	RE	30544147	CH	DC	2	1	1	1	0	0	0	1	2	2	1	2	2	1	16
Impact of Structured P	IN	RE	31306259	CH	RL	0	1	1	1	3	2	1	1	2	2	1	2	2	1	20
In the ICU - delirium p	IN	RE	30985357	CH	RL	2	1	1	1	0	0	0	0	2	2	1	2	2	1	15
In-Hospital Cardiac Ar	IN	RE	30912843	CH	DC	2	1	1	1	3	2	0	1	2	2	1	2	2	1	21
Management of Out-of	IN	RE	31758275	CH	GS	2	1	1	1	0	0	0	0	0	2	1	2	0	0	10
Management of post-ca	IN	RE	31723926	CH	KD	0	1	1	0	0	0	0	0	2	2	1	2	2	1	12
Neuroprotective Prope	IN	RE	31758401	CH	CH	2	1	1	1	0	0	0	1	2	2	1	2	0	1	14
Neuroprotective Prope	IN	RE	31758401	CH	RL	2	1	1	1	0	0	0	0	2	2	1	2	0	1	13
Optimal timing of cor	IN	RE	31580909	CH	KD	2	1	1	1	3	2	1	1	2	2	1	2	2	1	22
Out-of-hospital cardiac	IN	RE	31022086	CH	DC	2	1	1	1	0	0	0	0	2	2	1	2	0	1	13
Outcomes of extracorp	IN	RE	31790756	CH	DB	2	1	1	1	0	2	1	1	0	2	1	2	2	1	17
Perfusion parameters a	IN	RE	31473622	CH	RL	2	1	1	0	3	2	1	1	2	2	1	2	2	1	21
Postresuscitation care a	IN	RE	31021845	CH	DB	0	1	1	1	0	0	0	1	2	2	1	2	2	1	14

Postresuscitation Care	IN	RE	31021845	CH	CH	2	1	1	1	0	0	0	1	2	2	1	2	2	1	16
Prophylactic antibiotic	IN	RE	31085216	CH	DC	2	1	1	1	3	2	1	1	2	2	1	2	2	1	22
Racial Differences in tl	IN	RE	31845289	CH	RL	2	1	1	1	0	2	0	1	2	2	1	2	2	1	18
Rapid response systen	IN	RE	31723915	CH	DB	2	1	1	1	3	0	0	0	2	2	1	2	2	0	17
Short-term outcomes c	IN	RE	30485466	CH	GS	2	1	1	1	0	2	1	1	0	2	1	2	0	1	15
Sinus Bradycardia Dur	IN	RE	31660784	CH	CH	2	1	1	1	3	2	1	1	2	2	1	2	2	1	22
Sinus Bradycardia Dur	IN	RE	31660784	CH	KD	2	1	1	1	3	2	1	1	2	2	1	2	2	1	22
Strategies for Left Ven	IN	RE	31530454	CH	DC	2	1	1	1	0	2	0	1	2	2	1	2	2	1	18
Structured review of p	IN	RE	31522913	CH	GS	2	1	0	1	0	0	0	0	0	2	1	2	0	1	10
Systematic review and	IN	RE	31730898	CH	DC	2	1	1	1	3	2	1	1	2	2	1	2	2	1	22
Targeted temperature n	IN	RE	31384664	CH	DB	2	1	1	1	3	2	1	1	2	2	1	2	2	0	21
Targeted Temperature I	IN	RE	31262410	CH	KD	2	1	1	1	0	0	0	0	2	2	1	2	0	1	13
Targeted temperature n	IN	RE	31078561	CH	CH	2	1	1	1	0	0	0	1	2	2	1	2	2	1	16
Targeted temperature n	IN	RE	31078561	CH	RL	2	1	1	1	0	0	0	1	2	2	1	2	2	1	16
The necessity of conve	IN	RE	30342787	CH	DB	2	1	1	1	0	0	0	0	2	2	1	2	0	0	12
The Patient Selection (	IN	RE	31720177	CH	GS	2	1	1	1	0	0	0	0	2	2	1	2	2	1	15
The Use of Transesopl	IN	RE	30280396	DC	RC/KH	2	1	1	1	3	2	0	0	2	2	1	2	2	1	20
Therapeutic hypotherm	IN	RE	31778636	CH	GS	2	1	1	1	3	2	1	1	2	2	1	2	0	1	20
Utility and Challenges	IN	RE	31000007	CH	KD	2	1	1	1	0	0	0	0	2	2	1	2	0	1	13
Utility and Challenges	IN	RE	31000007	CH	CH	2	1	1	1	0	0	0	1	2	2	1	2	2	1	16
Venoarterial extracorpo	IN	RE	31116109	CH	KD	2	1	1	0	0	0	0	0	0	2	1	2	0	1	10
Vive la rÃ©sistance! T	IN	RE	30340016	CH	RL	2	1	1	1	0	2	0	1	2	2	1	0	0	1	14
2019 American Heart A	PED	RE	31727859	LA	MT	2	1	1	1	3	2	1	1	2	2	1	2	2	1	22
2019 American Heart A	PED	RE	31727859	LA	LA	2	1	1	1	3	2	0	1	2	2	1	2	2	1	21
2019 American Heart A	PED	RE	31722551	LA	MN	2	1	1	1	3	2	0	1	2	2	1	2	0	1	19
2019 American Heart A	PED	RE	31722551	LA	LA	2	1	1	1	3	2	0	1	2	2	1	2	2	1	21
2019 American Heart A	PED	RE	31722546	LA	SRK	2	1	1	1	3	2	0	1	2	2	1	2	0	1	19
2019 International Co	PED	RE	31722543	LA	EG	2	1	1	1	3	2	0	1	2	2	1	2	2	1	21
Advanced airway inter	PED	RE	30862528	LA	ME	2	1	1	1	3	0	0	1	2	2	1	2	0	1	17
Basic life support for t	PED	RE	31556731	LA	ME	2	0	1	1	0	0	0	1	2	0	1	2	0	0	10
Cardiovascular genomi	PED	RE	31256468	LA	MB	2	1	1	1	0	0	0	0	0	2	1	0	0	0	8
Chest Compressions fo	PED	RE	31671867	LA	MT	2	1	1	1	0	2	0	1	2	2	1	2	2	1	18
Chest-compression-onl	PED	RE	30391520	LA	MN	2	1	1	1	3	2	1	1	0	2	1	0	0	1	16
Comparison of two-thu	PED	RE	31702646	LA	MB	2	1	1	1	0	2	0	1	0	2	1	2	0	1	14
Defibrillation energy d	PED	RE	31029714	LA	SK	2	1	1	1	3	2	1	1	2	2	1	2	2	1	22
Defibrillation energy d	PED	RE	31029714	LA	LA	2	1	1	1	0	2	1	1	2	2	1	2	0	1	17
Defibrillation energy d	PED	RE	31029714	LA	SRK	2	1	1	1	3	2	1	0	2	2	1	2	0	1	19
Electrocardiographic A	PED	RE	30210005	LA	EG	0	1	0	1	0	0	0	0	0	2	1	0	0	0	5
Epidemiology of pedia	PED	RE	31580845	LA	ME	2	1	1	1	0	0	0	1	2	2	1	2	0	1	14
Epinephrine in Neonat	PED	RE	30987062	LA	ME	2	1	1	1	0	0	0	1	0	2	1	2	0	1	12
Epinephrine in Neonat	PED	RE	30987062	LA	MB	2	1	1	1	0	0	0	0	0	2	1	2	0	1	11
Extracorporeal membra	PED	RE	30854319	LA	EG	2	1	1	1	0	0	0	1	2	2	1	0	0	0	11
Paediatric targeted tem	PED	RE	30951842	LA	EG	2	1	1	1	3	2	1	1	2	2	1	2	0	1	20
Pediatric Out-of-Hospi	PED	RE	31042343	LA	SK	2	0	1	1	0	0	0	0	0	0	1	0	0	1	6
Pediatric Post–Cardiac	PED	RE	31242751	LA	MT	2	1	1	1	0	2	0	1	2	2	1	2	2	1	18
SUDEP and Grief: Ov	PED	RE	30338420	LA	MT	2	1	1	1	0	2	0	1	2	2	1	2	2	1	18
Therapeutic hypotherm	PED	RE	31278022	LA	MN	2	1	1	1	0	0	0	0	2	2	1	0	0	1	11
Therapeutic hypotherm	PED	RE	31278022	LA	LA	2	1	1	1	0	0	0	1	2	2	1	2	2	1	16
There is No Reason to	PED	RE	31286811	LA	EG	2	1	1	1	0	0	0	1	0	2	1	2	2	1	14
A meta-analysis of the	PRE	RE	30917840	DC	TD	2	1	1	1	3	2	1	1	2	2	1	2	2	0	21
A systematic review ar	PRE	RE	30853623	DC	KY	2	1	1	1	3	2	0	1	2	2	1	2	0	1	19
Adrenaline and vasopr	PRE	RE	30653257	DC	DC	2	1	1	1	3	2	1	1	2	2	1	2	0	1	20

Advancing emergency	PRE	RE	31592072	DC	KY	2	1	1	1	0	0	0	0	2	2	1	2	0	1	13
Bedside ultrasound in	PRE	RE	31889224	DC	TD	2	1	0	0	0	0	0	0	2	2	1	2	0	1	11
Beta-blockade for the t	PRE	RE	31790759	DC	DR	2	1	1	1	3	2	1	1	2	2	1	2	2	1	22
Can epinephrine therap	PRE	RE	30234557	DC	RC	2	1	1	1	0	2	1	1	2	2	1	2	0	1	17
Chest compression cor	PRE	RE	31536776	DC	CU	2	1	1	1	0	2	1	1	2	2	1	2	2	1	19
Chest compression cor	PRE	RE	31536776	DC	CU/KH	2	1	1	1	0	1	1	1	2	2	1	2	2	1	18
Clinical characteristics	PRE	RE	30771451	DC	TD	2	1	0	1	0	0	1	1	2	2	1	2	0	1	14
Comparing the efficacy	PRE	RE	31355224	DC	DC	2	1	1	1	0	2	1	1	2	2	1	2	0	1	17
Data Utilization in Em	PRE	RE	31042338	DC	DC	2	1	1	1	0	0	0	0	0	2	1	0	0	1	9
Data Utilization in Em	PRE	RE	31042338	DC	DR	2	1	1	1	3	2	0	0	2	2	1	2	2	1	20
Diagnostic performanc	PRE	RE	31887367	DC	DR	2	1	1	1	3	2	1	1	2	2	1	2	2	1	22
Double sequential defi	PRE	RE	31937443	DC	CU	2	1	1	1	3	2	1	1	2	2	1	2	0	1	20
Double sequential exte	PRE	RE	30612966	DC	DC	2	1	1	1	0	2	1	1	2	2	1	2	0	1	17
Double sequential exte	PRE	RE	30612966	DC	DR	2	1	1	1	3	2	1	1	2	2	1	2	2	1	22
Effects of epinephrine	PRE	RE	31702610	DC	KY	2	1	1	1	0	2	0	1	2	2	1	2	2	1	18
Efficacy and safety of a	PRE	RE	31561009	DC	TD	2	1	1	1	0	2	1	1	2	2	1	2	0	1	17
Emergency medical ser	PRE	RE	30973523	DC	KY	2	1	1	1	3	2	0	1	2	2	1	0	0	1	17
Emergent endotracheal	PRE	RE	30900042	DC	DR	2	1	1	1	3	2	0	1	2	2	1	2	2	1	21
Epinephrine for out of	PRE	RE	30685547	DC	DC	2	1	1	1	3	2	0	1	2	2	1	2	2	1	21
Epinephrine for Out-of	PRE	RE	31789700	DC	RC	2	0	1	1	3	2	1	1	2	2	1	2	0	1	19
How effective are epine	PRE	RE	31080 029	DC	CU	2	0	1	1	0	2	1	1	2	2	1	2	2	1	18
How effective are epine	PRE	RE	31080029	DC	CU	2	0	1	1	0	2	1	1	2	2	1	2	2	1	18
Is point of care ultraso	PRE	RE	30974189	DC	CU	2	1	1	1	3	2	1	1	2	2	1	2	2	1	22
Is point of care ultraso	PRE	RE	30974189	DC	CU	2	1	1	1	3	2	1	1	2	2	1	2	2	1	22
Mechanical chest comp	PRE	RE	31689757	DC	CU	2	1	1	1	3	2	1	1	2	2	1	2	2	1	22
Meta-analysis of the ad	PRE	RE	31142552	DC	SC	2	1	1	1	3	2	1	1	2	2	1	2	0	1	20
New physiological ins	PRE	RE	30531537	DC	KY	2	1	1	1	0	0	0	0	2	2	1	2	0	1	13
New physiological ins	PRE	RE	30531537	DC	KY/KH	2	1	1	1	0	0	0	1	2	2	1	2	2	1	16
Open-chest cardiopulm	PRE	RE	31881900	DC	KY	2	1	1	1	0	2	0	1	2	2	1	2	2	1	18
Standard dose epineph	PRE	RE	30658877	DC	KY	2	1	1	1	3	2	0	1	2	2	1	2	2	1	21
The effect of prehospit	PRE	RE	31455 452	DC	CU	2	1	1	1	3	2	1	1	2	2	1	2	2	1	22
The effect of prehospit	PRE	RE	31455452	DC	CU	2	1	1	1	3	2	1	1	2	2	1	2	2	1	22
The effect of prehospit	PRE	RE	31572937	DC	TD	2	0	1	0	3	0	0	0	0	2	1	2	0	0	11
The effect of prehospit	PRE	RE	31572937	DC	TD/KH	2	1	1	1	3	2	0	0	2	2	1	2	2	1	20
The effects of adrenalin	PRE	RE	31116964	DC	DR	2	1	1	1	3	2	1	1	2	2	1	2	2	1	22
The evolving role of n	PRE	RE	31836341	DC	DR	2	1	1	1	3	2	1	1	2	2	1	2	2	1	22
Understanding the adv	PRE	RE	31849717	DC	CU	2	1	1	1	0	2	0	1	2	2	1	2	0	1	16
Assessing brain injury	PRO	RE	31022084	CM	SZ	0	1	1	0	0	0	0	0	2	2	1	2	0	1	10
Assessing brain injury	PRO	RE	31022084	CM	CM	0	1	1	0	0	0	0	0	2	2	1	2	0	1	10
Assessment of neurocc	PRO	RE	31022085	CM	CC	2	1	1	1	0	0	0	0	2	2	1	2	0	1	13
Diagnostic evaluation	PRO	RE	31411341	CM	SW	0	1	1	1	0	0	0	0	2	2	1	0	0	0	8
Diagnostic evaluation	PRO	RE	31411341	CM	CM	0	1	1	1	0	0	0	0	2	2	1	0	0	0	8
Diagnostic performanc	PRO	RE	30878375	CM	SZ	2	1	1	1	0	2	0	1	2	2	1	2	0	1	16
Diagnostic performanc	PRO	RE	30878375	CM	CM	2	1	1	1	0	2	0	1	2	2	1	0	0	1	14
Effect of Palliative Ca	PRO	RE	31579159	CM	SW	0	1	1	0	0	0	0	1	2	2	1	0	0	1	9
Gap Analysis Regardin	PRO	RE	31368059	CM	JP	2	1	1	0	0	0	0	0	0	0	1	0	0	1	6
Imaging for Neuroprog	PRO	RE	31549351	CM	TK	2	1	1	1	0	1	0	1	2	2	1	2	0	1	15
Imaging for Neuroprog	PRO	RE	31549351	CM	CM	2	1	1	0	3	1	0	1	2	2	1	2	0	1	17
Neurological Prognost	PRO	RE	30739213	CM	SW	2	1	1	0	0	0	0	0	2	2	1	0	0	0	9
Neurological Prognost	PRO	RE	30739213	CM	CM	2	1	1	0	0	0	0	0	2	2	1	0	0	0	9
Neurological Prognost	PRO	RE	30739213	CM	TK	2	1	1	0	0	0	0	0	2	2	1	2	0	1	12
Neurological Prognost	PRO	RE	30739213	CM	CM	2	1	1	0	0	0	0	0	2	2	1	2	0	1	12

Pre-arrest and intra-arre	PRO	RE	31801749	CM	SZ	2	1	1	1	0	2	0	1	2	2	1	2	0	1	16
Procalcitonin as a prog	PRO	RE	30872069	CM	CC	2	1	1	1	3	2	1	1	2	0	1	0	0	1	16
Shock Index as a Predi	PRO	RE	30719462	CM	TK	2	1	1	0	0	0	0	0	2	2	1	0	0	1	10
SSEP retains its value	PRO	RE	31647028	CM	SW	0	1	1	1	0	0	0	0	2	2	1	0	0	0	8
Standards for Studies	PRO	RE	31291775	CM	TK	2	1	1	1	0	0	0	0	2	2	1	2	2	1	15
The Full Outline of Ur	PRO	RE	30411302	CM	JP	2	1	1	1	0	2	0	1	0	0	1	0	0	1	10
The Influence of Thera	PRO	RE	31768661	CM	SZ	2	1	1	1	0	0	0	0	2	2	1	2	0	1	13
