# Supplementary material for: Cardiac arrest: An interdisciplinary scoping review of the literature from 2019
Source: Resusc Plus. 2020 Nov 4;4:100037. doi: 10.1016/j.resplu.2020.100037 (PMC8244427; doi:10.1016/j.resplu.2020.100037)
Supplement: Supplementary file 6 [file mmc6.docx]

**Supplement #6 Editor and Reviewer Score Distributions**

Median and interquartile range of scoring by reviewer and editors for each category

| **Initials** | **Category** | **Role** | **OR Articles** | **RE Articles** |
| --- | --- | --- | --- | --- |
| CA | BSP | Reviewer | 17 (15 - 19) | --- |
| JL | BSP | Reviewer | 16 (15 - 18) | --- |
| MC | BSP | Reviewer | 18 (16.8 - 19.3) | --- |
| SC | BSP/EPH/IN | Reviewer | 15 (14 - 16) | 14.5 (13.5 - 16.3) |
| TM | BSP | Editor | 16 (15 - 18) | --- |
| EM | EPH | Reviewer | 14 (13 - 15.8) | 16 (14 - 18) |
| JW | EPH | Reviewer | 16 (14 - 17) | 16 (13.5 - 17.5) |
| SB | EPH | Reviewer | 16 (15 - 17) | 13 (10 - 20) |
| YE | EPH | Reviewer | 15 (14 - 17) | 15 (14 - 18.3) |
| MABC | EPH | Editor | 18 (17 - 18) | 10 (10 - 20) |
| MPB | EPH | Editor | 15 (13.5 - 16) | 14 (11.5 - 17) |
| DR | GL | Reviewer | --- | 13 (11 - 13) |
| JD | GL | Reviewer | --- | 18 (12 - 22) |
| RB | GL | Editor | --- | 13 (12 - 19) |
| DB | IN | Reviewer | 17 (14 - 18) | 17 (14 - 19) |
| DC | IN | Reviewer | 16 (15 - 18) | 18 (13 - 22) |
| GS | IN | Reviewer | 17 (14 - 20) | 15 (10.5 - 17) |
| KD | IN | Reviewer | 17 (14 - 18) | 16.5 (12.8 - 20.5) |
| RL | IN | Reviewer | 18 (15 - 20) | 16 (14.3 - 19.5) |
| CH | IN | Editor | 17 (15.5 - 18) | 16 (14.5 - 16) |
| EG | PED | Reviewer | 16 (14.3 - 17.8) | 14 (11 - 20) |
| MB | PED | Reviewer | 14 (13 - 15) | 11 (9.5 - 12.5) |
| ME | PED | Reviewer | 16 (15 - 17.5) | 13 (11.5 - 14.8) |
| MN | PED | Reviewer | 14 (14 - 16) | 16 (13.5 - 17.5) |
| MT | PED | Reviewer | 19 (18 - 20) | 18 (18 - 19) |
| SK | PED | Reviewer | 15 (13.8 - 18) | 19 (15.8 - 19.8) |
| LA | PED | Editor | 17 (16 - 18) | 19 (16.8 - 21) |
| CU | PRE | Reviewer | 18 (16 - 20) | 21 (18.3 - 22) |
| DR | PRE | Reviewer | 20 (20 - 20) | 22 (21.5 - 22) |
| KH | PRE | Reviewer | 19.5 (17.5 - 20) | 20 (18 - 20) |
| KY | PRE | Reviewer | 19 (18 - 20) | 18 (15 - 18.5) |
| RC | PRE | Reviewer | 19 (17 - 20) | 18 (17.5 - 18.5) |
| TD | PRE | Reviewer | 16 (14 - 18) | 14 (11 - 17) |
| DC | PRE | Editor | 16.5 (12.8 - 18) | 17 (17 - 20) |
| CC | PRO | Reviewer | 14 (13 - 16) | 14.5 (13.8 - 15.3) |
| JP | PRO | Reviewer | 14 (13 - 16) | 8 (7 - 9) |
| SW | PRO | Reviewer | 14 (14 - 15) | 8.5 (8 - 9) |
| SZ | PRO | Reviewer | 14 (13 - 16) | 14.5 (12.3 - 16) |
| TK | PRO | Reviewer | 15 (14 - 15.8) | 13.5 (11.5 - 15) |
| CM | PRO | Editor | 14 (13 - 16) | 11 (9.3 - 13.5) |
