# Supplementary material for: Cardiac arrest: An interdisciplinary scoping review of the literature from 2019
Source: Resusc Plus. 2020 Nov 4;4:100037. doi: 10.1016/j.resplu.2020.100037 (PMC8244427; doi:10.1016/j.resplu.2020.100037)
Supplement: Supplementary file 8 [file mmc8.pdf]

Name	Email	Role	Group	Affiliation
Torben K Becker	t.becker@ufl.edu	Editor-in-Chief		University of Florida, College of Medicine, Department of Emergency Medicine, Division of Critical Care
Travis W Murphy	travis.murphy@surgery.ufl.edu	Managing Editor		University of Florida, College of Medicine, Department of Emergency Medicine, Division of Critical Care
Scott Cohen, MPH	scohen211@ufl.edu	Technical Editor		University of Florida, College of Medicine, Department of Emergency Medicine
Francis Han	omarhan2011@ufl.edu	Assistant Technical Editor		University of Florida, College of Medicine, Department of Emergency Medicine
Carolina B Maciel	carolina.maciel@neurology.ufl.edu	Editor	PRO	University of Florida, College of Medicine, Department of Neurology, Division of Neurocritical Care
Charles W Hwang	c7places@ufl.edu	Editor	IN	University of Florida, College of Medicine, Department of Emergency Medicine
David B. Crabb	dcrabb@ufl.edu	Editor	PRE	University of Florida, College of Medicine, Department of Emergency Medicine
K. Leslie Avery	avery4@ufl.edu	Editor	PED	University of Florida, College of Medicine, Department of Pediatrics
Karl Huesgen	karlhuegen@ufl.edu	Editor	PRE	University of Florida, College of Medicine, Department of Emergency Medicine
Meenakshi P. Balakrishnan	meenakshipb@ufl.edu	Editor	EPH	University of Florida, College of Medicine, Department of Emergency Medicine
Muhammad Abdul Baker Chowdhury	chowdhurym@ufl.edu	Editor	EPH	University of Florida, College of Medicine, Department of Emergency Medicine
Ramani Balu	ramani.balu@pennmedicine.upenn.edu	Editor	GL	University of Pennsylvania, Department of Neurology, Division of Neurocritical Care
Sarah Gul	Sarah.Gul@neurosurgery.ufl.edu	Editor	BSP	Yale University, School of Medicine, Department of Surgery
Casey Carr	caseytercarr@gmail.com	New Reviewer	PRO	University of Florida, College of Medicine, Department of Emergency Medicine
Chanteil Ulatowski	c.ulatowski@ufl.edu	New Reviewer	PRE	University of Florida, College of Medicine, Department of Emergency Medicine
Colton B Amaral	colton.amaral@gmail.com	New Reviewer	BSP	University of Florida, Alachua County Fire Rescue - Critical Care Division
Daniel Buchalter	dbuchalter372351@student.wmcarey.edu	Senior Reviewer	IN	William Carey University, College of Osteopathic Medicine
Daniel Ralston	dralston@ufl.edu	New Reviewer	GL	University of Florida, College of Medicine, Department of Emergency Medicine
Danielle Roberts	danirobertsmd@gmail.com	New Reviewer	PRE	University of Florida, College of Medicine, Department of Emergency Medicine
Dru Curtis	drucurtis@ufl.edu	New Reviewer	IN	University of Florida
Ellen George	emcngeorge@gmail.com	Senior Reviewer	PED	University of Florida, College of Medicine, Department of Pediatrics
Emmett Martin	emmett1986@ufl.edu	New Reviewer	EPH	University of Florida, College of Medicine, Department of Emergency Medicine
Garrett Snipes	snipesg@ufl.edu	Senior Reviewer	IN	University of Florida, College of Medicine, Department of Emergency Medicine
Jeremy Latimer	jeremy.latimer@neurology.ufl.edu	New Reviewer	BSP	University of Florida, College of Medicine, Department of Neurocritical Care
A. Jessica Pinto	ajessica.pinto@ufl.edu	New Reviewer	PRO	University of Florida, College of Medicine, Department of Emergency Medicine
John Dollerschell	jdollerschell@anest.ufl.edu	Senior Reviewer	GL	Univeristy of Florida, College of Medicine, Department of Anesthesiology
Jon Wiese	jonwiese@ufl.edu	New Reviewer	EPH	University of Florida
Kathryn Dasburg	kdasburg@ufl.edu	New Reviewer	IN	University of Florida, College of Medicine, Department of Emergency Medicine
Kayvon Yazdanbakhsh	kayvonyazdan@gmail.com	Senior Reviewer	PRE	Melrose Fire Rescue Department
Manjiri Tule	mtule@ufl.edu	Senior Reviewer	PED	University of Florida, College of Medicine, Department of Pediatrics
Maria Echavarria	mechavarria@ufl.edu	Senior Reviewer	PED	University of Florida, College of Medicine, Department of Pediatrics
Marie Nader	mnader@ufl.edu	New Reviewer	PED	Yale Univeristy, School of Medicine, Section of Pediatric Critical Care Medicine, Department of Pediatrics,
Meghan Bowser	mbowser1@ufl.edu	New Reviewer	PED	University of Florida, College of Medicine, Department of Pediatrics
Morgan William Carson-Marino	mwcmarino@ufl.edu	Senior Reviewer	BSP	University of Florida, College of Pharmacy, Department of Pharmacotherapy and Translational Research
Robert Cueto	cuetorobertj@ufl.edu	New Reviewer	PRE	University of Florida, College of Medicine, Department of Emergency Medicine
Robert Leverence	robert.leverence@mybcom.org	New Reviewer	IN	New Mexico State University, Burrell College of Osteopathic Medicine
Sabuj Chandra Bhowmick	Sabuj606@gmail.com	Senior Reviewer	EPH	University of Oslo, Department of Mathematics and Statistics
Sara Kim	sara.kim@ufl.edu	New Reviewer	PED	University of Florida, College of Medicine, Department of Pediatrics
Shannon Williams	sjwilliams@ufl.edu	New Reviewer	PRO	University of Florida, College of Medicine, Department of Emergency Medicine
Sonya E Zhou	sonya.zhou@yale.edu	Senior Reviewer	PRO	Yale University, School of Medicine, Department of Neurology
Terri Davis	davist@ufl.edu	Senior Reviewer	PRE	University of Florida, College of Medicine, Department of Emergency Medicine
Thomas King	thomascking@ufl.edu	New Reviewer	PRO	University of Florida
Yasmeen O. Elmelige	yelmelige@ufl.edu	Senior Reviewer	EPH	University of Florida, College of Medicine, Department of Emergency Medicine
